# Organic Dye Photocatalyzed Synthesis of Functionalized Lactones and Lactams via a Cyclization–Alkynylation Cascade

**DOI:** 10.1021/acs.orglett.4c01078

**Published:** 2024-05-13

**Authors:** Diana Cavalli, Jerome Waser

**Affiliations:** Laboratory of Catalysis and Organic Synthesis, Institute of Chemical Sciences and Engineering, Ecole Polytechnique Fédérale de Lausanne, CH-1015 Lausanne Switzerland

## Abstract

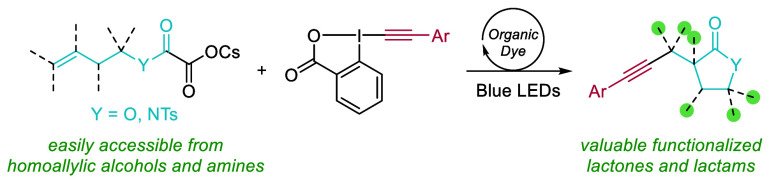

An organic dye photocatalyzed lactonization–alkynylation of easily accessible homoallylic cesium oxalates using ethynylbenziodoxolone (EBX) reagents has been developed. The reaction gave access to valuable functionalized lactones and lactams in up to 88% yield via the formation of two new C–C bonds. The transformation was carried out on primary, secondary, and tertiary homoallylic alcohols and primary homoallylic amines and could be applied to the synthesis of spirocyclic compounds as well as fused and bridged bicyclic lactones.

γ-Lactones and -lactams are found in numerous agrochemicals, pharmaceuticals, and bioactive natural products (Scheme 1A). For example, spironolactone (**1a**) is used to treat heart failure,^1^ santonin (**1b**) is used as an anthelmintic drug,^2^ and nirmatrelvir (**1c**) is used for the treatment of COVID-19.^3^ Hence, various synthetic approaches have been developed over the years to construct γ-lactones, mainly focusing on the formation of C–O bonds (Scheme 1B). Esterification of 1,4-hydroxy acids, Baeyer–Villiger oxidation, and cyclization of carboxylic acids onto olefins are among the most frequently used methods.^4^

**Scheme 1:**
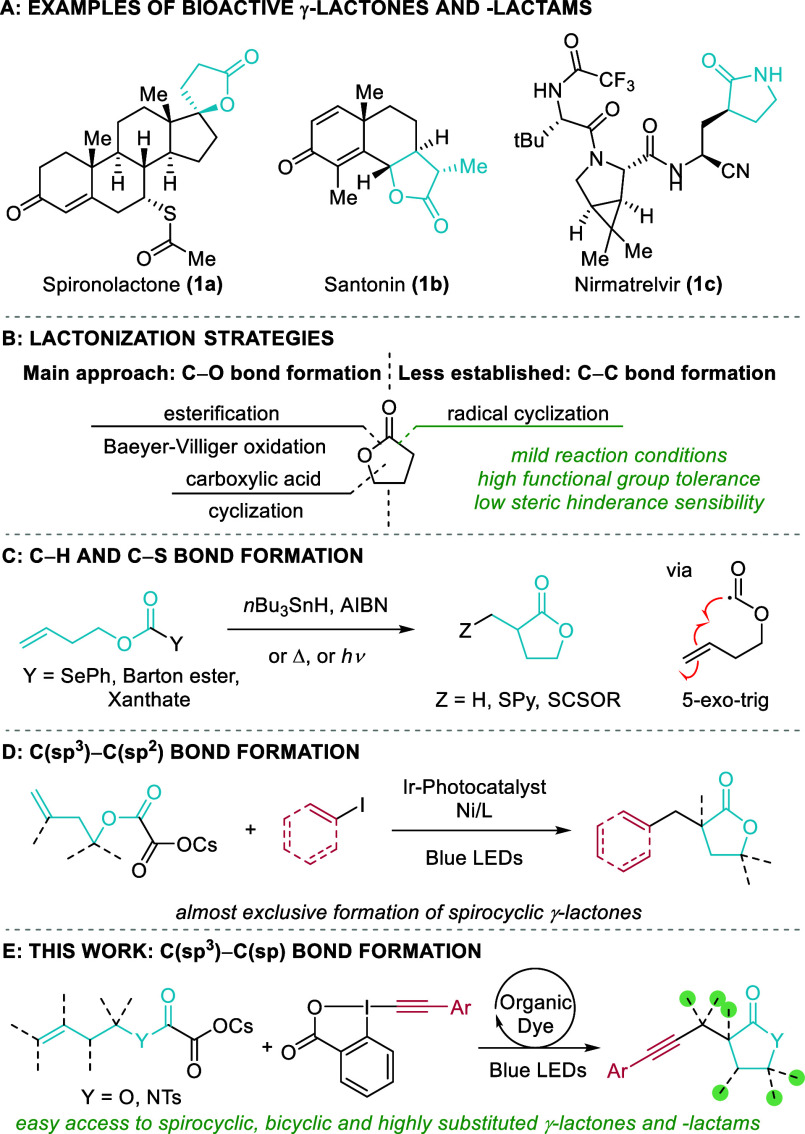
γ-Lactones and γ-Lactams: Examples of Bioactive Compounds (A) and Synthetic Strategies (B–E)

In contrast, the synthesis of γ-lactones and -lactams via C–C bond formation has been less investigated, although it has the potential for a more convergent construction of the carbon backbone of the molecules. Radical-based approaches are especially attractive due to their high functional group tolerance, mild reaction conditions, and lower sensibility toward steric hindrance. In particular, the 5-*exo*-*trig* cyclization of an alkoxycarbonyl radical onto an olefin is an attractive strategy as the radical precursor can be generated from easily accessible homoallylic alcohols and the radical generated after cyclization can be trapped by a SOMOphile, hence allowing multifunctionalization reactions (Scheme 1C).^5,6^ In this regard, lactonization has been reported using selenocarbonates,^7,8^*N*-alkoxyoxalyloxy-2-thiopyridones,^9,10^ and *S*-alkoxycarbonyl xanthates^11^ under classical conditions for the generation of radicals. The formed alkyl radicals were then trapped by a hydrogen atom, a thiopyridine, or a xanthate moiety, with only one example of double C–C bond formation in a 5-*exo*-*trig*–Giese addition sequence.^9^

More recently, Overman and co-workers reported the synthesis of arylated and vinylated spirolactones via a photoredox-catalyzed alkoxycarbonyl radical cyclization–Ni-catalyzed cross-coupling cascade using easily accessible homoallylic cesium oxalates as substrates and an iridium photocatalyst (Scheme 1D).^12^

This work was focused on the synthesis of spirolactones, with only one example each of a lactone/lactam derived from a primary alcohol or amine, respectively, indicating that the Thorpe–Ingold effect is not necessary for the cyclization.

The reported examples allowed the formation of a second C–C bond including a C(sp^3^) or C(sp^2^) carbon. Nevertheless, there is no example of C(sp^3^)–C(sp) bond formation. Alkynes are versatile functional groups: they can either be used as a rigid linker or converted into other functionalities such as carboxylic acids. Thus, they find numerous applications in various fields such as medicinal chemistry, chemical biology, and materials science;^13,14^ hence, the development of new methods for their installation is highly appealing. Along the various radical approaches developed over the years to access alkynylated compounds, ethynylbenziodoxolones (EBXs) have been shown to be efficient radical traps.^15−19^ Furthermore, Leonori and co-workers have shown that EBXs can be used to terminate photoredox-catalyzed radical cyclization–alkynylation cascade reactions.^20^ However, this process was based on subsequent C–N and C–C bond formation rather than two C–C bond formations as envisaged here.

In our previous work on the alkynylative deoxygenation of cesium oxalates, we had observed a single example of cyclization–alkynylation on a preorganized rigid substrate.^21^ Herein, we introduce a general method for the synthesis of highly functionalized γ-lactones and -lactams starting from easily accessible homoallylic cesium oxalates using an organic dye and EBXs as radical traps through an efficient lactonization–alkynylation cascade (Scheme 1E).

Based on previous reports on photomediated alkynylation,^21,22^ we started our investigation using homoallylic cesium oxalate **2a** as starting material, 1.5 equiv of PhEBX (**3a**) as SOMOphile, and 5 mol % 4CzIPN (**4a**) as photocatalyst (Table 1). After irradiation (44 W, 440 nm) in DCM overnight, the desired spirolactone **5a** was obtained in 53% yield (entry 1). Changing the photocatalyst for the more oxidizing 4ClCzIPN (**4b**)^23^ provided **5a** in 64% yield (entry 2). The chlorinated solvent could be replaced with DMSO without a change in yield (entry 3). In this case, full conversion was obtained and **5a** was the only product that could be isolated, indicating most probably background polymerization of alkene **2a**. To slow down this competing pathway, the concentration of **2a** was lowered to 0.05 M and the catalyst loading was decreased to 2%, while keeping the concentration of PhEBX (**3a**) to 1.5 M, which indeed provided spirolactone **5a** in increased 75% yield (entry 4). Additionally, the yield could be further improved to 80% by lowering the power of the light source to 22 W and stirring the reaction mixture for 42 h to ensure full conversion of **2a** (entry 5). It could be speculated that the concentration of free radicals is lower under these conditions, preventing polymerization. Finally, in absence of any photocatalyst, **5a** was obtained in 14% yield due to the innate photoactivity of PhEBX (**3a**)^21^ (entry 6). In the absence of light, no product was observed (entry 7).

**Table 1:**
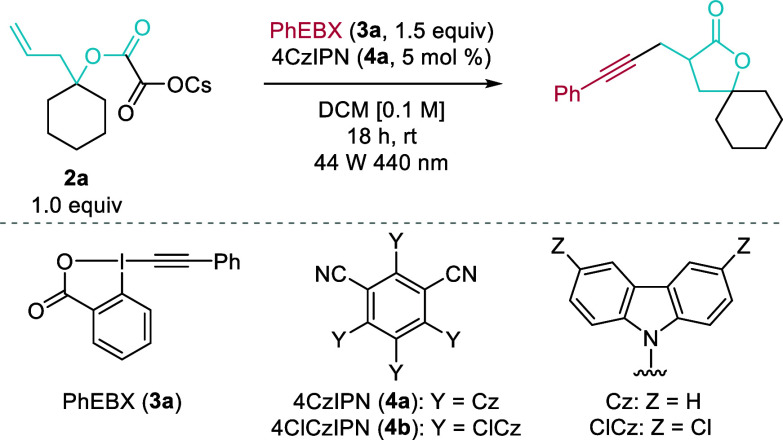
Optimization of the Reaction Conditions^a^

Entry	Modification from standard conditions	Yield (%)^b^
1	None	53
2	PC: 4ClCzIPN (**4b**)	64
3	As above, solvent: DMSO	62
	3.0 equiv of PhEBX (**3a**)	
4	PC: 4ClCzIPN (**4b**, 2 mol %)	75
	Solvent: DMSO [0.05 M]	
5	As above, 22 W light source, 42 h	80
6	As above, no PC	14
7	No light source	0

a Reaction conditions: 0.1 mmol of **2a**, 1.5 equiv of PhEBX (**3a**), 5 mol % of 4CzIPN (**4a**), DCM [0.1 M], 44 W 440 nm LEDs, rt, 18 h.

b Yields determined by ^1^H NMR spectroscopy using mesitylene as internal standard.

With the optimized conditions in hand, we first investigated the scope of the lactonization reaction on tertiary alcohols to obtain spirolactones (Scheme 2). The model substrate **2a** cyclized smoothly on a 0.3 mmol scale, giving **5a** in 76% isolated yield. Furthermore, performing the same reaction on a 3.0 mmol scale provided **5a** in 69% yield. Spiroheterocycles **5b** and **5c** were obtained in 86% and 70% yield, respectively. The size of the second ring in the spirocycle could be varied broadly to give compounds **5d**, **5e**, and **5f** in 77%, 81%, and 74% yield, respectively. The dehydroepiandrosterone-derived spirolactone **5g** was obtained in 50% yield with 3:1 dr. This compound is the analogue of an intermediate reported in the synthesis of spironolactone (**1a**), requiring only four additional steps to potentially access the alkynylated derivative of **1a**.^24^

**Scheme 2:**
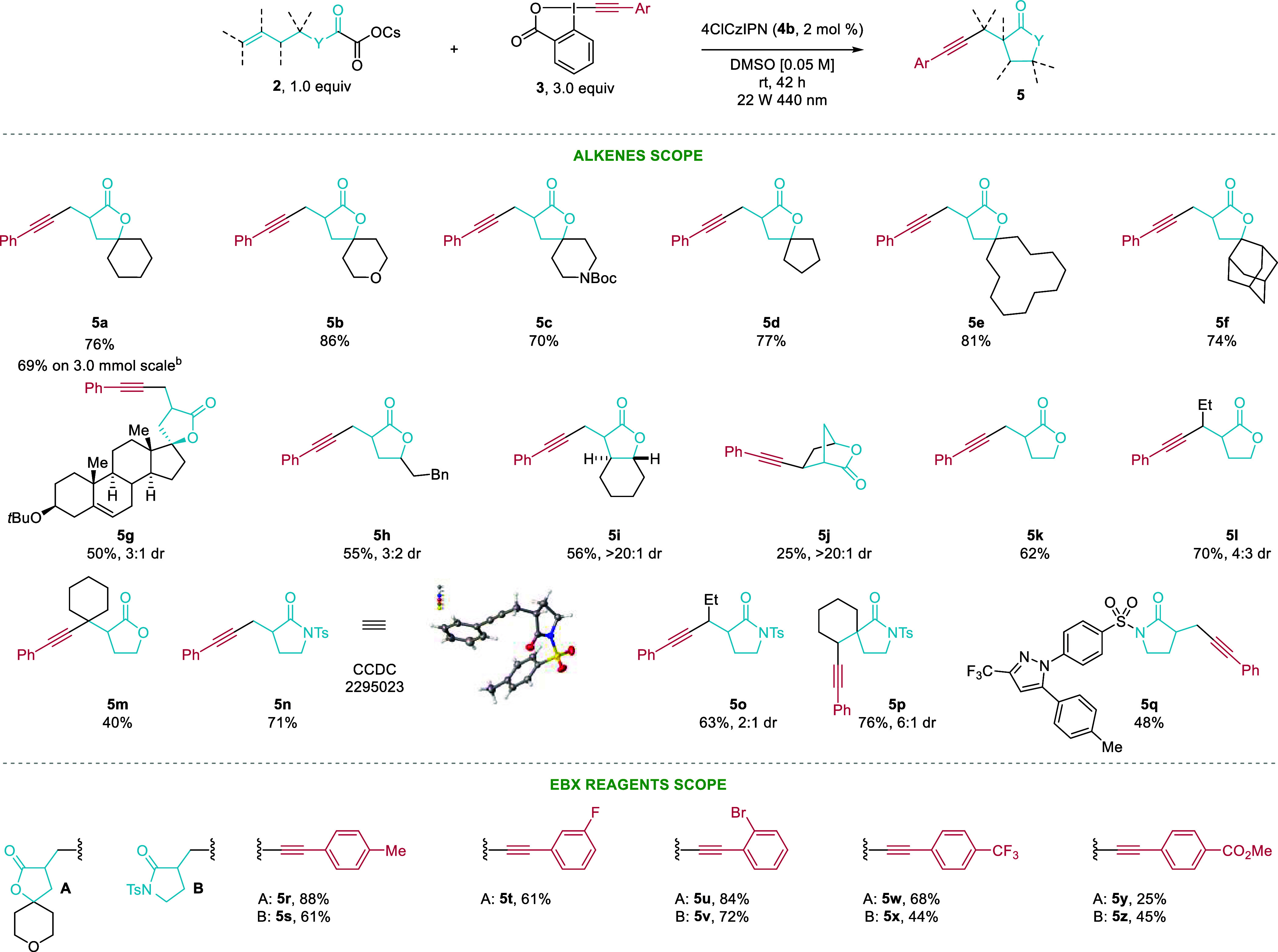
Scope of the Cyclization–Alkynylation Cascade Reaction conditions: 0.3 mmol of cesium oxalate or oxamate (**2**), 3.0 equiv of ArEBX (**3**), 2 mol % of 4ClCzIPN (**4b**), DMSO [0.05 M], 22 W 440 nm LEDs, rt, 42 h. Modifications: 44 W LEDs, 18 h.

Having established the scope of tertiary alcohols, we moved our attention to secondary homoallylc cesium oxalates. This class of substrates had not been investigated by Overman and co-workers.^12^ Lactone **5h** having a pendent alkyl chain could be isolated in 55% yield with 3:2 dr. Furthermore, performing the reaction from *trans*-1,2-vinylcyclohexanol provided *trans* 6/5 fused lactone **5i** in 56% yield and perfect diastereoselectivity, which is found in the natural product santonin (**1b**). Additionally, we were pleased to obtain bridged compound **5j** in 25% yield as a single diastereoisomer.

Finally, the alkynylation–lactonization reaction was performed on primary homoallylic cesium oxalates. We used this class of substrates to study the difference in the reactivity of the primary, secondary, or tertiary radical intermediate formed after cyclization in the alkynylation step. In the case of C(sp^3^)–C(sp^2^) bond formation, only primary intermediates had been reported.^12^ Lactone **5k** resulting from alkynylation of a primary radical intermediate was obtained in 62% yield, while a *cis*-hexenol-derived cesium oxalate was cyclized in 70% yield and 4:3 dr through the alkynylation of a secondary radical to give **5l**. Lactone **5m**, which arose from a tertiary radical intermediate, was isolated in 40% yield. We speculate that the trapping of the radical with PhEBX (**3a**) was less efficient in this case due to steric congestion.

In addition to lactones, we were interested in applying the same reaction conditions on homoallylic cesium oxamates to obtain alkynylated γ-lactams. To our delight, a simple primary tosyl-protected amide provided lactam **5n** in 71% yield. The structure of **5n** was confirmed by X-ray crystallography. Also, a *cis*- hexenamine derivative cyclized successfully to give **5o** in 63% yield with 2:1 dr. Despite several attempts, tertiary homoallylic cesium oxamates only provided the product resulting from alkynylation of the aminocarbonyl radical prior to cyclization. We speculate that the 5-*exo*-*trig* cyclization is not occurring due to a nonfavorable conformation of the aminocarbonyl radical. Nevertheless, spirolactam **5p** with a different connectivity can still be obtained in 76% yield and 6:1 dr starting from a cyclohexenylethylamine oxamate as a radical precursor. Finally, the lactamization–alkynylation reaction was performed by using a Celecoxib derivative to afford lactam **5q** in 48% yield, demonstrating that more complex functionalized sulfonimides were tolerated in the reaction.

The scope of the Ar-EBX reagents was then explored on both lactonization and lactamization reactions using either cesium oxalate **2a** or cesium oxamate **2n**. *p*TolEBX (**3b**) provided compounds **5r** and **5s** in 88% and 61% yield, respectively. Halogenated Ar-EBX reagents **3c** and **3d** were tolerated under the reaction conditions: the fluorinated compound **5t**, was isolated in 61% yield, while brominated EBX reagent **3d** provided cyclized products **5u** and **5v** in 84% and 72% yield, respectively. Finally, aryl alkynes containing electron-withdrawing groups such as trifluoromethyl (**5w** and **5x**) and methyl ester (**5y** and **5z**) were obtained in 68%, 44%, 25%, and 45% yield, respectively. Unfortunately, silyl- and alkyl-substituted EBX reagents as well as other hypervalent iodine reagents, such as cyanobenziodoxolone and vinylbenziodoxol(on)es, gave only traces of the desired product (see the Supporting Information for details).

Additionally, the δ-spirolactone **7** was obtained in 45% isolated yield *via* 6-*exo-trig* cyclization from homologous cesium oxalate **6** (eq 1).
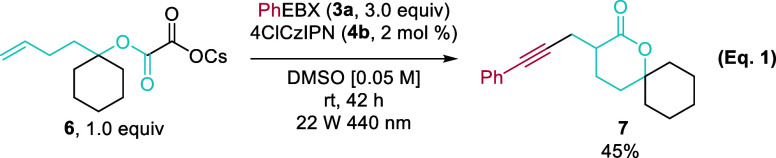
1

In order to demonstrate the synthetic utility of the obtained alkynylated spirolactones, post-functionalization reactions were carried out from **5a** (Scheme 3A). First, we envisaged introducing a different synthetic handle than the alkyne. Therefore, spirolactone **5a** was subjected to a *one-pot* Ru-catalyzed 1,2-diketone formation–Baeyer–Villiger oxidation procedure, giving carboxylic acid derivative **8** in 73% yield.^25^

**Scheme 3:**
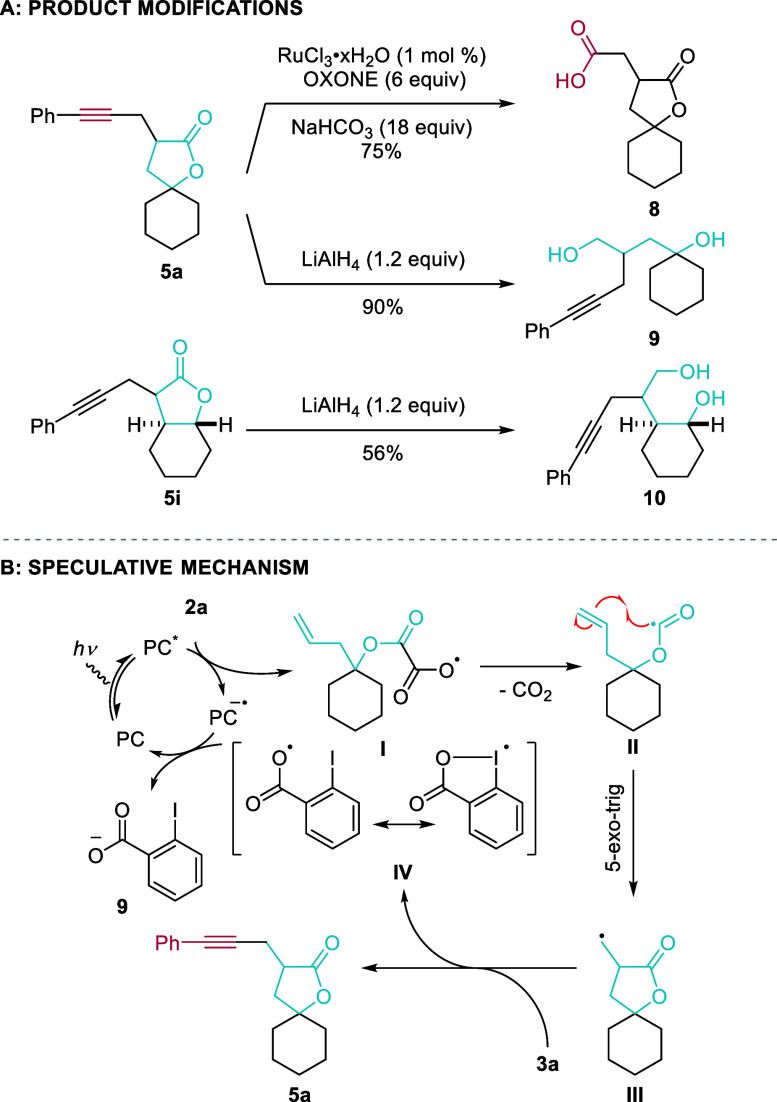
(A) Product Modifications. (B) Proposed Mechanism for the Cyclization–Alkynylation Cascade

Second, the lactone was opened reductively by treatment with LiAlH_4_ leading to 1,4-diol **9** having a pendent alkynyl group in 90% yield. Finally, the same reaction conditions were applied to lactone **5i**, which had been obtained as a single diastereoisomer, to provide diol **10** in 56% yield. This sequence therefore allowed installation of three contiguous stereocenters with high relative stereocontrol.

Scheme 3B details a speculative mechanism for the lactonization–alkynylation reaction of homoallylic oxalates **2** based on literature precedence.^23,26−28^ Light irradiation would result in the excitation of 4ClCzIPN (**4b**, PC) to its excited state, 4ClCzIPN* (PC*). PC* (*E*_1/2_(PC*/PC^•–^) = +1.71 V vs SCE in MeCN)^23^ would then undergo single electron transfer (SET) from oxalate salt **2a** (*E*_*p*_(**2a**^–^/**2a**^•^) = +1.71 V vs SCE in MeCN) generating radical **I**. Intermediate **I** would then undergo fast decarboxylation, generating alkoxycarbonyl radical **II**. This intermediate, instead of losing a second equivalent of CO_2_, would undergo 5-*exo*-*trig* cyclization with the alkene forming radical intermediate **III**.^27^ The latter is then trapped by PhEBX (**3a**) generating the desired alkynyl-γ-lactone **5a** and iodanyl radical **IV** (*E*_1/2_(**IV**^•^/**IV**^–^) ≈ −0.25 V,^28^ which would then turn over the photocatalyst (*E*_1/2_(PC/PC^•–^) = −0.97 V vs SCE in MeCN) and generate iodobenzoate (**11**).

In conclusion, a metal-free photocatalyzed lactonization–alkynylation reaction of homoallylic cesium oxalates has been developed. In addition to valuable spirolactones, bicyclic compounds and substituted lactones and lactams were also obtained. Moreover, various halogenated- and electron-donating or -withdrawing groups containing aryl EBX reagents were successfully used in the reaction. Finally, the synthetic utility of the obtained compounds was demonstrated by transforming the alkyne into a carboxylic acid and reducing the lactones to 1,4-diols.^29^

## Data Availability

The data underlying this study are available in the published article and its Supporting Information. Raw data for NMR and MS is available free of charge from zenodo.org at https://doi.org/10.5281/zenodo.11186147.

## Supporting Information Available

Experimental procedures and analytical data for all new compounds; copy of NMR spectra (PDF)
