# Supplementary material for: Organic Dye Photocatalyzed Synthesis of Functionalized Lactones and Lactams via a Cyclization–Alkynylation Cascade
Source: Org Lett. 2024 May 13;26(20):4235–9. doi: 10.1021/acs.orglett.4c01078 (PMC11129301; doi:10.1021/acs.orglett.4c01078)

Organic Dye Photocatalyzed Synthesis of Functionalized Lactones and Lactams via a Cyclization-Alkynylation Cascade

Diana Cavalli, and Jerome Waser*

Laboratory of Catalysis and Organic Synthesis, Institute of Chemical Sciences and Engineering, Ecole Polytechnique Fédérale de Lausanne, CH-1015 Lausanne, Switzerland.

Table of Contents

Table of Contents 2
1. General Methods 3
2. Photochemical Experimental Set-up 4
3. Reaction Optimization 4
4. Unsuccessful Substrates 5
5. Experimental part 6
5.1. Starting Material Synthesis 8
5.1.1. Synthesis of Ethynylbenziodoxolones 8
5.1.2. Synthesis of Photocatalysts 12
5.1.3. Synthesis of Homoallylic alcohols and amides 14
5.1.3.1. Tertiary Alcohols 14
5.1.3.2. Secondary Alcohols 20
5.1.3.3. Primary Alcohols 21
5.1.3.4. Primary Amides 23
5.1.4. Synthesis of Homoallylic Cesium Oxalates and Oxamates 28
5.2. Photoredox Catalyzed Lactonization/Lactamization Reaction 43
5.3. Product Modifications 59
6. X-Ray crystallography data 62
7. References 67
8. NMR Spectra of New Compounds 68

1. General Methods

All reactions were carried out in oven-dried glassware and under an atmosphere of nitrogen unless stated otherwise. For flash chromatography, distilled technical grade solvents were used. THF, CH_3CN, toluene, Et_2O and DCM were dried by passage over activated alumina under nitrogen atmosphere (H_2O content < 10 ppm, Karl-Fischer titration). The solvents were degassed by Freeze-Pump-Thaw method or Ar-bubbling when mentioned. All chemicals were purchased from Acros, Aldrich, Fluka, VWR, TCI, Merck and used as such unless stated otherwise. Chromatographic purification was performed as flash chromatography using Macherey-Nagel silica 40-63, 60 Å, using the solvents indicated as eluent with 0.1-0.5 bar pressure. TLC was performed on Macherey-Nagel pre-coated TLC sheets ALUGRAM® Xtra SIL G/UV_{254} and visualized with UV light and *p*-anisaldehyde stain (EtOH:H_2SO_4:AcOH:*p*-anisaldehyde 135:5:1.5:3.7, V:V:V:V).

^1H NMR spectra were recorded on a Brucker DPX-400 400 MHz spectrometer in $CDCl_3$, CD_3CN, MeOD or DMSO-d_6. All signals are reported in ppm with the internal chloroform signal at 7.26 ppm, the internal acetonitrile signal at 1.94 ppm, the internal methanol signal at 3.30 ppm or the internal DMSO signal at 2.50 ppm as standard. The data is reported as (s = singlet, d = doublet, t= triplet, q = quadruplet, qi = quintet, m = multiplet or unresolved, br = broad signal, coupling constant(s) in Hz, integration, interpretation). ^{13}C {^1H} NMR spectra were recorded with ^1H-decoupling on a Brucker DPX-400 101 MHz spectrometer in $CDCl_3$, CD_3CN, MeOD or DMSO-d_6. All signals are reported in ppm with the internal chloroform signal at 77.0 ppm, the internal acetonitrile signal at 1.3 ppm the internal methanol signal at 49.0 ppm or the internal DMSO signal at 39.5 ppm as standard. Diastereomeric ratios has been determined by ^1H NMR spectroscopy of the crude reaction.

High resolution mass spectrometric measurements were performed by the mass spectrometry service of ISIC at the EPFL on a MICROMASS (ESI) Q-TOF Ultima API.

All photocatalyzed reactions were carried out in oven dried glassware and under inert atmosphere (degassed solvent stored on molecular sieves and under nitrogen for maximum one month) unless specified otherwise. They were performed in screw cap dram vials (0.5 – 7,5 mL) which were stuck to a glass plate that was placed on a stirring plate with 1 Kessil lamp (440 nm, 22 W) irradiating from one side (the hood was free and the reaction was covered with an orange Plexiglas cover). The distance between the Kessil lamps and the vials was approximatively 7 cm. Long irradiation resulted in temperature increasing up to 50 °C during overnight reactions unless a fan was used in which case the temperature raised to 25 °C.

2. Photochemical Experimental Set-up

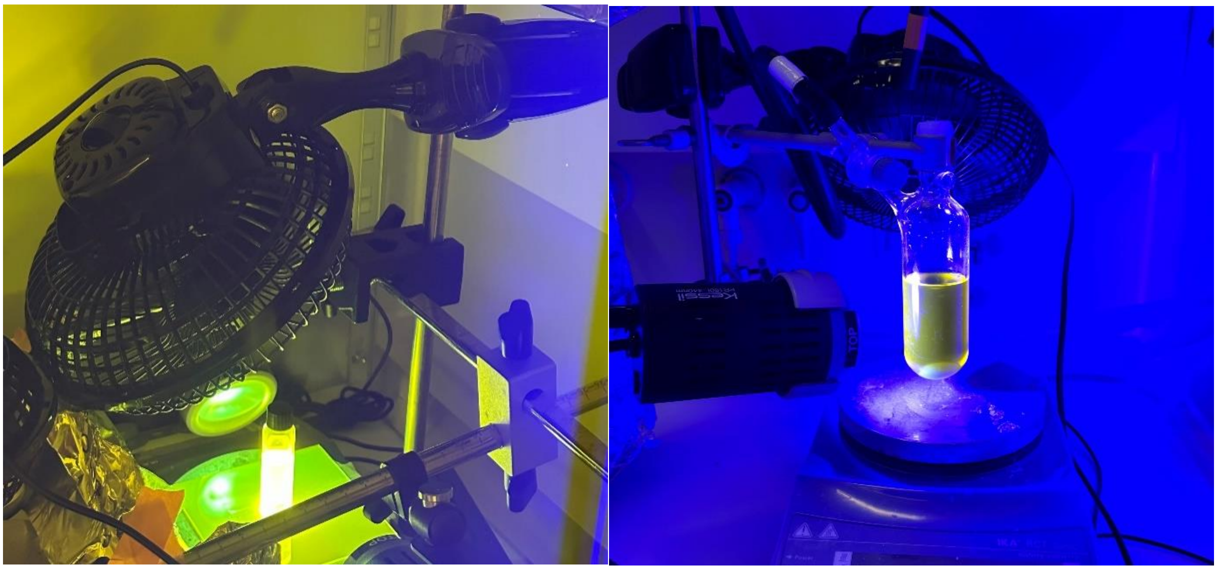

Figure S1: Left: Scope scale reaction. Right: 3.0 mmol scale reaction.

3. Reaction Optimization

Table S1: Reaction optimization.

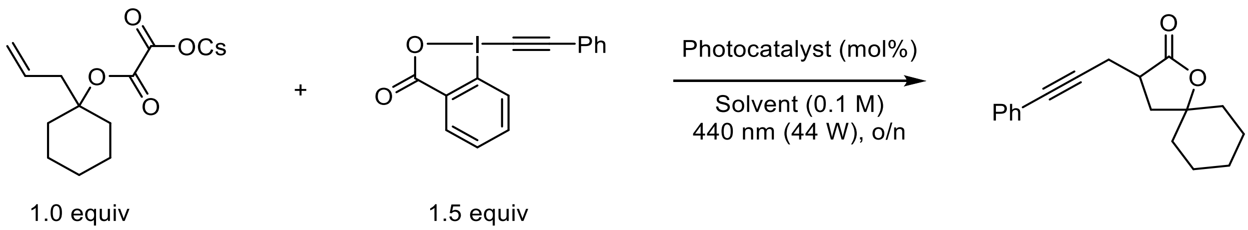

Entry	Photocatalyst (mol%)	Solvent	Yield[a]
**1**	4ClCzIPN (5)	MeCN	35
**2**	4ClCzIPN (5)	MeOH	18
**3**	4ClCzIPN (5)	THF	27
**4**	4ClCzIPN (5)	Toluene	10
**5**	4ClCzIPN (5)	DCE	63
**6**	4DPAIPN (5)	DCE	15
**7**	MesAcr•ClO_4 (5)	DCE	18
**8**	[Ir(dFCF$_3$ppy)dtbbpy]PF_6 (2)	DCM	58
**9**	4ClCzIPN (5)	DCM + H_2O (10 equiv)	57
**10[b]**	4ClCzIPN (5)	DMSO-d$_6$	55

a) Yield determined by ^{1}H NMR spectroscopy using Mesitylene as internal standard. b) The reaction was not performed under inert atmosphere.

Experimental procedure: An oven-dried 2.5 mL screw-cap vial equipped with a magnetic stirring bar was charged with PhEBX (**3a**) and photocatalyst. The vial was brought to a glove box where cesium oxalate **2a** was added. The vial was closed with a septum, removed from the glove box and an Ar balloon was added. Degassed solvent (freeze-pump-thaw degassing for volatile solvents, Ar bubbling for 1 h for high boiling point solvents) was added and the septum was replaced with a screw-cap under a flux of Ar. The vial was irradiated (440 nm, 44 W) while cooling with a fan overnight. The solvents were removed under reduced pressure, mesitylene (0.33 equiv) was added and the crude was analyzed by ^{1}H NMR spectroscopy.

4. Unsuccessful Substrates

Hypervalent iodine reagents:

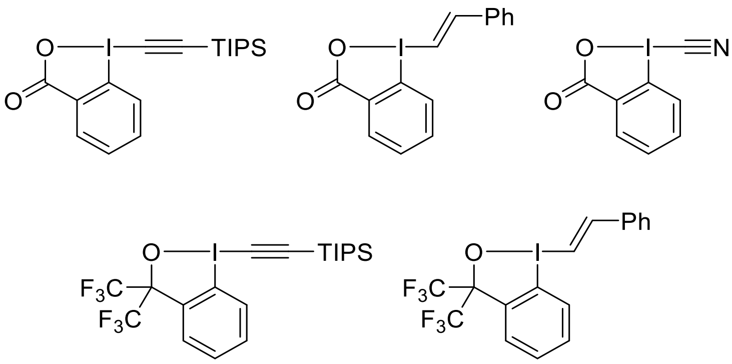

Olefins:

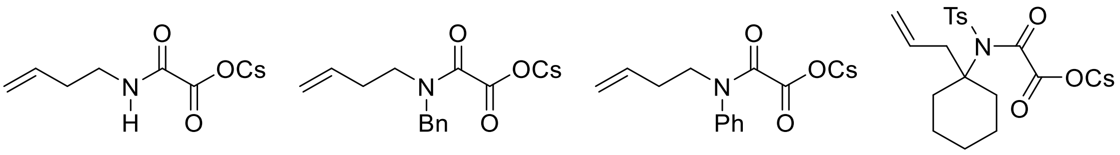

5. Mechanistic Investigation

5.1. Cyclic Voltammetry Data for Cesium 2-((1-allylcyclohexyl)oxy)-2-oxoacetate (2a)

An Autolab potentiostat with a three-electrode cell configuration: surface Pt (working electrode), Pt (control electrode), and Ag/AgCl (NaCl, 3M aq., reference electrode) was used for the measures. Tetrabutyl ammonium hexafluorophosphhate (TBAP, 0.1 M in MeCN) was used as an electrolyte. The sample (approx.. 20 mg) was dissolved in a stock solution of TBAP (0.1 M, 14 mL in MeCN) and was degassed by bubbling Nitrogen directly before measure. The voltammogram was recorded at 1V/s. In absence of reversible behavior, the formal oxidation potential was determined at the $E_{p,max}$ and converted from $E_{Ag/AgCl}$ to E_{SCE}.

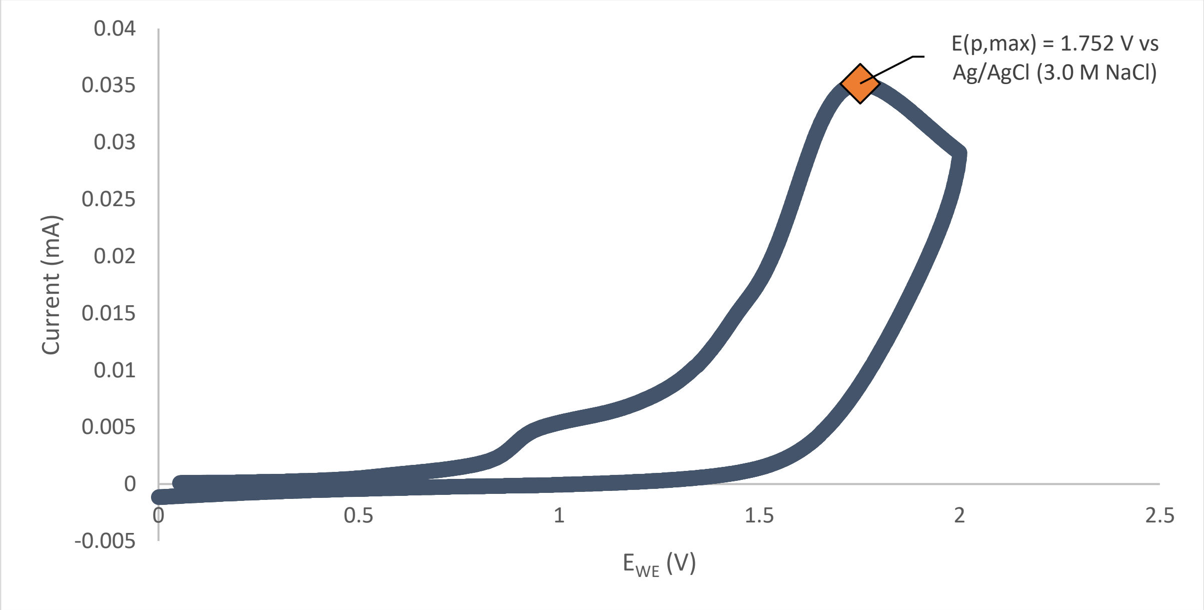

Graph 1 Cyclic voltammogram of **2a**.

$$E_{SCE} = E_{Ag/AgCl} + E^{sat}_{Ag/AgCl} - E^{sat}_{SCE}$$
$$E_{SCE} = 1.752 + 0.197 - 0.241$$
$$E_{SCE} = 1.708\,V$$

$E_{p,max}$ = 1.71 V vs SCE

5.2. Stern Volmer Photoluminescence Quenching

The Photoluminescence quenching study was carried out with an Agilent Cary Eclipse fluorescence spectrometer.

A solution of **2a** (0.01 M in DMSO, degassed) and a solution of 4ClCzIPN (**4b**, 10 nM in DMSO, degassed) were prepared. 2.0 mL of the photocatalyst solution was added to a 3.0 mL fluorimeter cuvette and its emission was measured. Successively, the quencher was added progressively using a 100 µL Hamilton syringe as following:

Table S2: mL of **2a** solution added before each fluorescence measurement.

eq **2a**	0	10	25	50	75	100	150	200
μL added	0	20	30	50	50	50	100	100

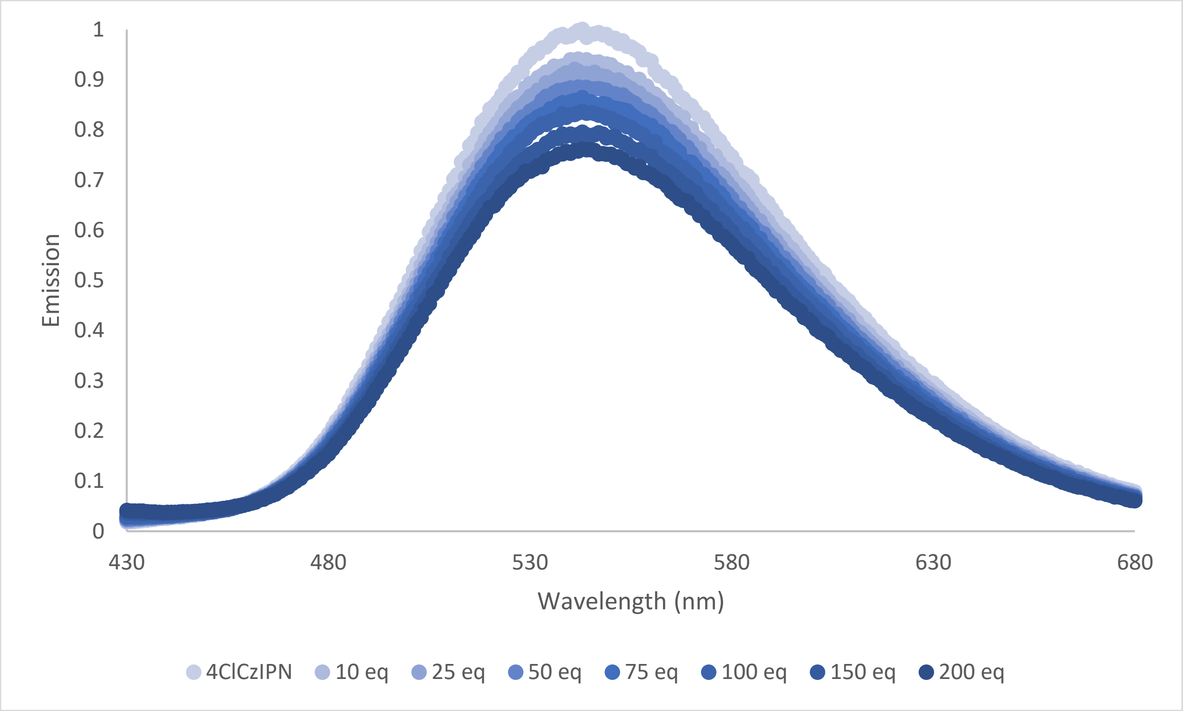

Graph 2: Normalized fluorescence emission spectra of **4b** at different quencher (**2a**) equivalents.

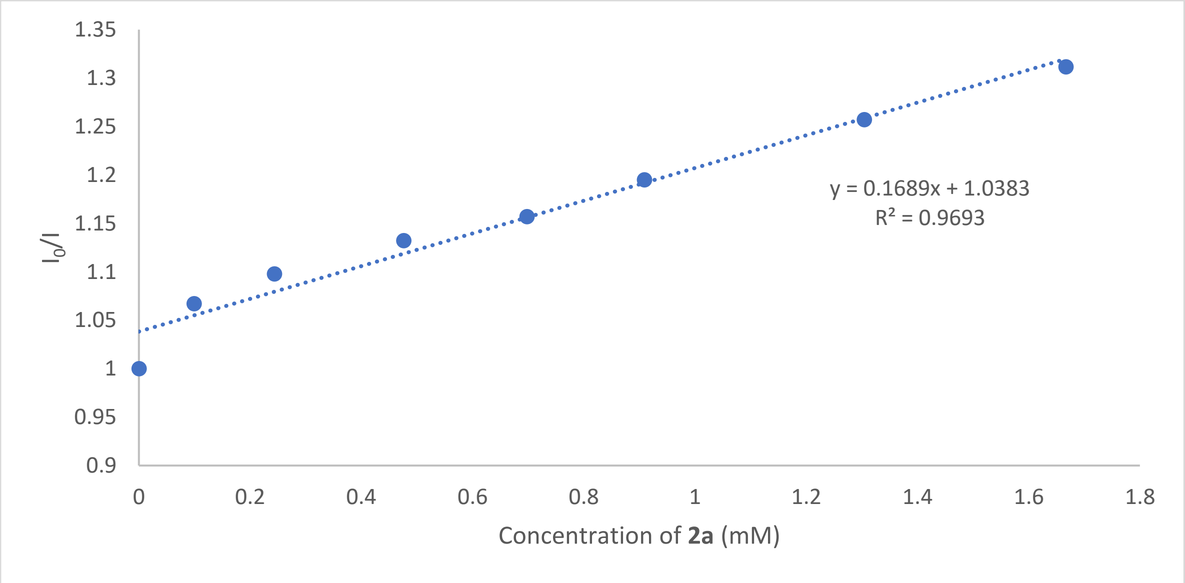

Graps 3: Stern-Volmer plot for **2a**.

6. Experimental part

6.1. Starting Material Synthesis

6.1.1. Synthesis of Ethynylbenziodoxolones

The synthesis of reagents **3a**, **3b**, **3c**, **3d** and **3e** had already been described before by our group. The procedures are taken from the indicated publications.

1-Hydroxy-1,2-benziodoxol-3-(1*H*)-one (S2)

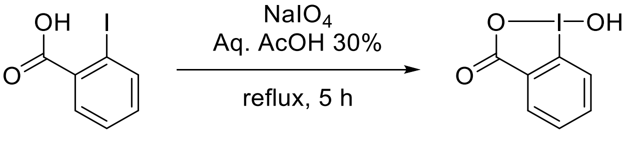

S1 **S2**

Following a reported procedure,[1] $NaIO_4$ (40.5 g, 189 mmol, 1.05 equiv) and 2-iodobenzoic acid (**S1**, 44.8 g, 180 mmol, 1.0 equiv) were suspended in 30% (v:v) aq. AcOH (350 mL). The mixture was vigorously stirred and refluxed for 5 h. The reaction mixture was then diluted with cold water (250 mL) and allowed to cool to rt, protecting it from light. After 1 h, the crude product was collected by filtration, washed on the filter with ice water (3 x 150 mL) and acetone (3 x 150 mL), and air-dried in the dark overnight to afford 1-Hydroxy-1,2-benziodoxol-3-(1*H*)-one (**S2**, 44.3 g, 168 mmol, 93% yield) as a white solid.

Analytical data is consistent with literature values.[1]

^{1}H NMR (400 MHz, $CDCl_3$) δ/ppm: 8.02 (dd, *J* = 7.7, 1.4 Hz, 1H, Ar*H*), 7.97 (m, 1H, Ar*H*), 7.85 (dd, *J* = 8.2, 0.7 Hz, 1H, Ar*H*), 7.71 (td, *J* = 7.6, 1.2 Hz, 1H, Ar*H*).

^{13}C {^{1}H} NMR (101 MHz, $CDCl_3$) δ/ppm: 167.7, 134.5, 131.5, 131.1, 130.4, 126.3, 120.4.

1-[Phenylethynyl]-1,2-benziodoxol-3(1*H*)-one (PhEBX, 3a)

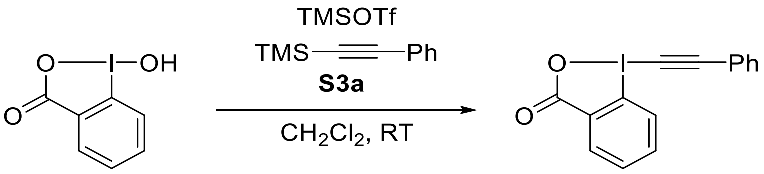

S2 **3a**

Following a reported procedure,[1] trimethylsilyltriflate (9.1 mL, 50 mmol, 1.1 equiv) was added dropwise to a suspension of 2-iodosylbenzoic acid (**S2**, 12.1 g, 45.8 mmol, 1.0 equiv) in DCM (120 mL) at 0 °C. The mixture was stirred for 1 h, followed by the dropwise addition of trimethyl(phenylethynyl)silane (**S3a**, 8.8 mL, 50 mmol, 1.1 equiv) (slightly exothermic). The resulting suspension was stirred for 6 h at RT, during this time a white solid was formed. A saturated solution of $NaHCO_3$ (120 mL) was added and the mixture

[1] Amos, S. G. E.; Cavalli, D.; Le Vaillant, F.; Waser, J. Direct Photoexcitation of Ethynylbenziodoxolones: An Alternative to Photocatalysis for Alkynylation Reactions. *Angew. Chem. Int. Ed.* **2021**, *60* (44), 23827–23834.

was stirred vigorously for 30 min. The two layers of the mother liquors were separated and the organic layer was washed with sat. $NaHCO_3$ (2x50 mL), dried over $MgSO_4$, filtered and evaporated under reduced pressure. The resulting solid was recrystalized in EtOAc:MeOH (7:3 v:v) (ca. 20 mL). The solution was left to cool to RT then in the freezer ovenight, filtered and dried under high vacuum to afford PhEBX (**3a**, 6.8 g, 25 mmol, 43% yield) as colorless crystals.

Analytical data is consistent with literature values.[1]

^{1}H NMR (400 MHz, $CDCl_3$) δ/ppm: 8.46 (m, 1H, Ar*H*), 8.28 (m, 1H, Ar*H*), 7.80 (m, 2H, Ar*H*), 7.63 (m, 2H, Ar*H*), 7.48 (m, 3H, Ar*H*).

^{13}C {^{1}H} NMR (101 MHz, $CDCl_3$) δ/ppm: 163.9, 134.9, 132.9, 132.5, 131.6, 131.3. 130.8, 128.8, 126.2, 120.5, 116.2, 106.6, 50.2.

1-(p-Tolylethynyl)-1,2-benziodoxol-3(1*H*)-one (3b)

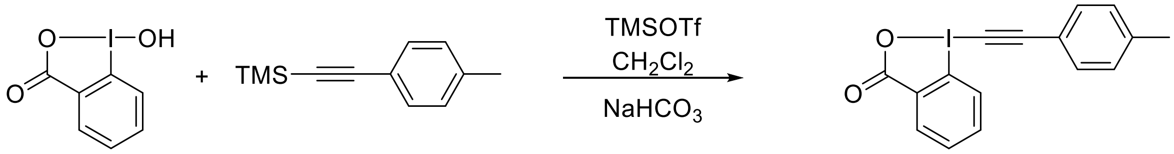

S2 **S3b** **3b**

Following a reported procedure,[1] trimethylsilyl triflate (1.0 mL, 5.5 mmol, 1.1 equiv) was added to a suspension of 2-iodosylbenzoic acid (**S2**) (1.32 g, 5.00 mmol, 1.00 equiv) in DCM (15 mL) at room temperature. The resulting suspension was stirred for 3 h, followed by the drop wise addition of trimethyl(p-tolylethynyl)silane (**S3b**) (1.04 g, 5.50 mmol, 1.10 equiv). The resulting suspension was stirred for 6 h at room temperature. A saturated solution of $NaHCO_3$ (20 mL) was then added and the mixture was stirred vigorously for 30 minutes, the two layers were separated and the organic layer was washed with saturated solution of $NaHCO_3$ (20 mL), dried over Na_2SO_4, filtered and evaporated under reduced pressure. The resulting solid was recrystallized from EtOAc:MeOH 7:3 (ca 20 mL). The mixture was cooled down, filtered and dried under high vacuum to afford **3b** (0.620 g, 1.71 mmol, 45%) as white crystals.

Analytical data is consistent with literature values.[1]

^{1}H NMR (400 MHz, $CDCl_3$) δ/ppm: 8.43 (dd, J = 6.1, 2.9 Hz, 1H, Ar*H*), 8.30– 8.14 (m, 1H, Ar*H*), 7.77 (dd, J = 6.9, 3.1 Hz, 2H, Ar*H*), 7.50 (d, J = 7.8 Hz, 2H, Ar*H*), 7.25 (d, J = 7.6 Hz, 2H, Ar*H*), 2.43 (s, 3H, ArC*H*$_3$).

^{13}C {^{1}H} NMR (101 MHz, $CDCl_3$) δ/ppm: 166.6, 141.5, 134.9, 132.8, 132.5, 131.6, 131.3, 129.5, 126.2, 117.4, 116.2, 107.3, 49.1, 21.7.

1-[3-Fluorophenylethynyl]-1,2-benziodoxol-3(1*H*)-one (3c)

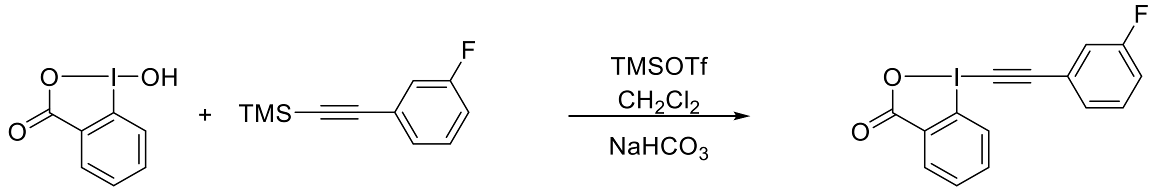

S2 **S3c** **3c**

Following a slightly modified reported procedure,[1] trimethylsilyl triflate (0.44 mL, 2.5 mmol, 1.1 equiv) was added to a suspension of 2-iodosylbenzoic acid (**S2**, 0.589 g, 2.23 mmol, 1.00 equiv) in DCM (6.8 mL) at RT The resulting suspension was stirred for 1 h, followed by the dropwise addition of ((3-fluorophenyl)ethynyl)trimethylsilane (**S3c**, 0.50 mL, 2.5 mmol, 1.1 equiv). The resulting suspension was stirred for 6 h at RT. A saturated solution of $NaHCO_3$ (10 mL) was then added and the mixture was stirred vigorously for 30 minutes, resulting in a suspension. The mixture was diluted with chloroform (10 mL), water (5 mL) and MeOH (ca. 0.5 mL) resulting in two clear layers. The two layers were separated, and the organic layer was washed with sat. $NaHCO_3$ (7 mL), dried over Na_2SO_4, filtered, and evaporated under reduced pressure. The resulting solid was recrystallized in EtOAc:MeOH (7:3 v:v) (ca. 20 mL). The solution was left to cool to RT then was placed in the freezer (-20 °C) overnight. The crystals were filtered and washed with Et_2O to afford **3c** (787 mg, 2.15 mmol, 43% yield) as colorless crystals.

Analytical data is consistent with literature values.[1]

^{1}H NMR (400 MHz, DMSO-*d*$_6$) δ/ppm: 8.33 (dd, *J* = 8.2, 0.8 Hz, 1H, Ar*H*), 8.13 (dd, *J* = 7.4, 1.7 Hz, 1H, Ar*H*), 7.91 (ddd, *J* = 8.2, 7.2, 1.7 Hz, 1H, Ar*H*), 7.81 (td, *J* = 7.3, 0.9 Hz, 1H, Ar*H*), 7.64 – 7.59 (m, 1H, Ar*H*), 7.58 – 7.53 (m, 2H, Ar*H*), 7.47 – 7.37 (m, 1H, Ar*H*).

^{13}C {^{1}H} NMR (101 MHz, DMSO-*d*$_6$) δ/ppm: 166.3, 161.8 (d, *J* = 245.6 Hz), 135.3, 131.9, 131.3, 131.2 (d, *J* = 8.7 Hz), 129.0 (d, *J* = 2.9 Hz), 127.7, 122.4 (d, *J* = 9.6 Hz), 119.2 (d, *J* = 23.4 Hz), 118.1 (d, *J* = 21.1 Hz), 116.4, 102.5 (d, *J* = 3.3 Hz), 53.8.

^{19}F NMR (376 MHz, DMSO-*d*$_6$) δ/ppm: –111.7.

1-[2-Bromophenylethynyl]-1,2-benziodoxol-3(1*H*)-one (3d)

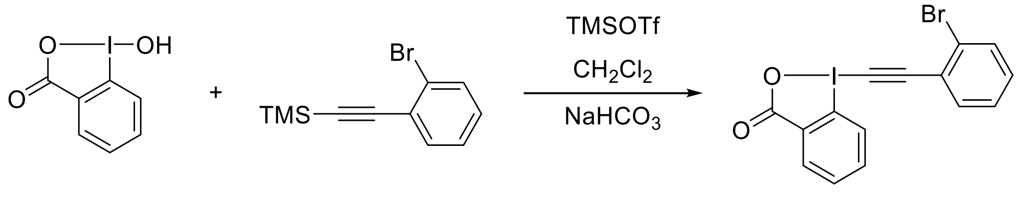

S2 **S3d** **3d**

Following a slightly modified reported procedure,[1] trimethylsilyl triflate (0.42 mL, 2.4 mmol, 1.1 equiv) was added to a suspension of 2-iodosylbenzoic acid (**S2**, 0.562 g, 2.13 mmol, 1.00 equiv) in DCM (6 mL) at RT The resulting suspension was stirred for 1 h, followed by the drop wise addition of ((2-bromophenyl)ethynyl)trimethylsilane (**S3d**, 0.50 mL, 2.4 mmol, 1.1 equiv). The resulting suspension was stirred for 6 h at RT. A saturated solution of $NaHCO_3$ (10 mL) was then added and the mixture was stirred vigorously for 1 h resulting in a persistent emulsion/suspension. The mixture was diluted with $CHCl_3$ (10

mL), water (5 mL) and MeOH (ca. 2 mL) to afford 2 distinct layers. The two layers were separated, and the organic layer was washed with sat. $NaHCO_3$ (5 mL), dried over Na_2SO_4, filtered, and evaporated under reduced pressure. The resulting solid was recrystallized in EtOAc:MeOH (7:3 v:v) (ca. 20 mL). The solution was left to cool to RT then was placed in the freezer (-20 °C) overnight. The crystals were filtered and washed with Et_2O afford **3d** (1.50 g, 3.51 mmol, 70% yield) as colorless crystals.

Analytical data is consistent with literature values.[1]

^{1}H NMR (400 MHz, $CDCl_3$) δ/ppm: 8.44 (td, *J* = 7.3, 2.1 Hz, 2 H, Ar*H*), 7.84 – 7.74 (m, 2 H, Ar*H*), 7.68 (d, *J* = 1.1 Hz, 1 H, Ar*H*), 7.61 (dd, *J* = 7.6, 1.7 Hz, 1 H,*ArH*), 7.36 (m, 2 H, Ar*H*).

^{13}C {^{1}H} NMR (101 MHz, $CDCl_3$) δ/ppm: 166.6, 135.2, 134.7, 133.0, 132.7 , 131.8, 131.3, 127.6, 126.8, 126.4, 123.2, 116.5, 104.3, 55.4.

1-[4-Trifluoromethylphenylethynyl]-1,2-benziodoxol-3(1*H*)-one (3e)

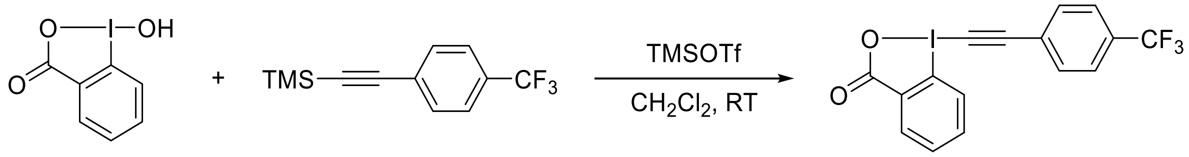

S2 **S3e** **3e**

Following a reported procedure,[1] trimethylsilyl triflate (1.0 mL, 5.5 mmol, 1.1 equiv) was added to a suspension of 2-iodosylbenzoic acid (**S2**, 1.3 g, 5.0 mmol, 1.0 equiv) in DCM (15 mL) at RT The resulting suspension was stirred for 1 h, followed by the dropwise addition of trimethyl((4-(trifluoromethyl)phenyl)ethynyl)silane (**S3e**, 1.3 mL, 5.5 mmol, 1.1 equiv), which was dissolved in DCM (1 mL). The resulting suspension was stirred for 6 h at RT A saturated solution of $NaHCO_3$ (20 mL) was then added and the mixture was stirred vigorously for 30 min, the two layers were separated and the organic layer was washed with sat. $NaHCO_3$ (20 mL), dried over $MgSO_4$, filtered and evaporated under reduced pressure. The resulting solid was boiled in CH_3CN (20 mL). The mixture was cooled down, filtered and dried under high vacuum to afford **3e** (1.3 g, 3.2 mmol, 64% yield) as a pale-yellow solid.

Analytical data is consistent with literature values.[1]

^{1}H NMR (400 MHz, $CDCl_3$) δ/ppm: 8.46 – 8.38 (m, 1H, Ar*H*), 8.28 – 8.19 (m, 1H, Ar*H*), 7.84 – 7.74 (m, 2H, Ar*H*), 7.74 – 7.65 (m, 4H, Ar*H*).

^{13}C {^{1}H} NMR (101 MHz, $CDCl_3$) δ/ppm: 166.6, 135.0, 133.0, 132.6, 132.2 (q, *J* = 33.0 Hz), 131.7, 131.2, 126.3, 125.7 (q, *J* = 3.6 Hz), 124.4, 123.4 (q, *J* = 272.6 Hz), 116.1, 104.2, 53.7.

1-[4-Methoxycarbonylphenylethynyl]-1,2-benziodoxol-3(1*H*)-one (3f)

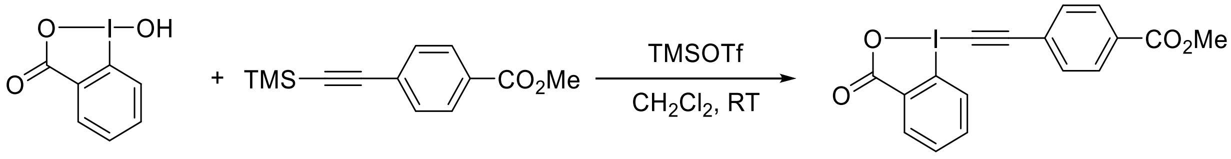

S2 **S3f** **3f**

Following a modified reported procedure,[1] trimethylsilyl triflate (0.80 mL, 4.4 mmol, 1.1 equiv) was added to a suspension of 2-iodosylbenzoic acid (**S2**, 1.06 g, 4.0 mmol, 1.0 equiv) in DCM (11 mL) at RT The resulting suspension was stirred for 1 h, followed by the dropwise addition of methyl 4(2-trimethylsilylethynyl)benzoate (**S3f**, 1.02 g, 4.4 mmol, 1.1 equiv), which was dissolved in DCM (1 mL). The resulting suspension was stirred for 6 h at RT A saturated solution of $NaHCO_3$ (20 mL) was then added and the mixture was stirred vigorously for 30 min, the two layers were separated and the organic layer was washed with sat. $NaHCO_3$ (20 mL), dried over $MgSO_4$, filtered and evaporated under reduced pressure. The resulting solid was boiled in CH_3CN (20 mL). The mixture was cooled down, filtered and dried under high vacuum to afford **3f** (1.54 g, 3.79 mmol, 95% yield) as a white solid.

Analytical data is consistent with literature values.[2]

^{1}H NMR (400 MHz, $CDCl_3$) δ/ppm: 8.48 – 8.39 (m, 1H, Ar*H*), 8.29 – 8.18 (m, 1H, Ar*H*), 8.15 – 8.05 (m, 2H, Ar*H*), 7.88 – 7.72 (m, 2H, Ar*H*), 7.72 – 7.62 (m, 2H, Ar*H*), 3.96 (s, 3H, C*H*$_3$).

^{13}C {^{1}H} NMR (101 MHz, $CDCl_3$) δ/ppm: 166.7, 166.1, 135.2, 132.8, 132.7, 131.9, 131.4, 129.9, 126.4, 125.1, 116.2, 105.2, 53.9, 52.7.

6.1.2. Synthesis of Photocatalysts

The synthesis of photocatalysts **4a** and **4b** had already been described before by our group. The procedures are taken from the indicated publications.

2,4,5,6-Tetra(9*H*-carbazol-9-yl)isophthalonitrile (4CzIPN, 4a)

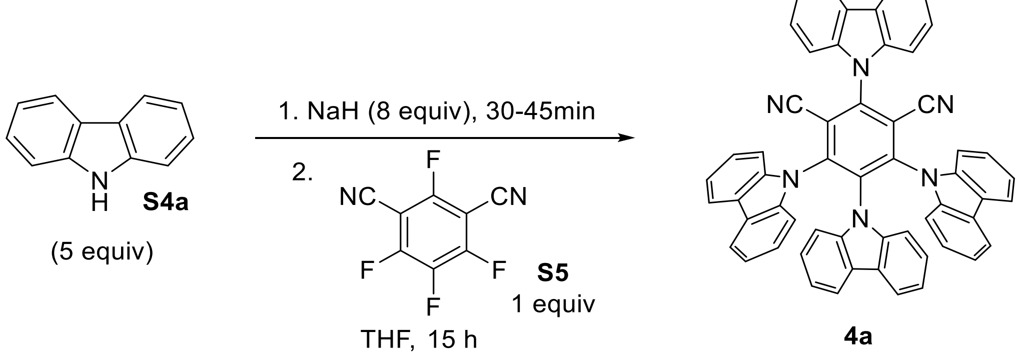

4a

Sodium hydride (60% suspension in mineral oil, 0.60 g, 15 mmol, 7.5 equiv) was added slowly to a stirred solution of 9H-carbazole **S4a** (1.67 g, 10.0 mmol, 5.00 equiv) in dry THF (40 mL) under a nitrogen

[2] Liu, B.; Lim, C.-H.; Miyake, G. M. Light-Driven Intermolecular Charge Transfer Induced Reactivity of Ethynylbenziodoxol(on)e and Phenols. *J. Am. Chem. Soc.* **2018**, *140* (40), 12829–12835.

atmosphere at RT After 30 min, 2,4,5,6-tetrafluoroisophthalonitrile **S5** (0.40 g, 2.0 mmol, 1.0 equiv) was added. After stirring at RT for 15 h, 2 mL water was added to the reaction mixture to quench the excess of NaH. The resulting mixture was then concentrated under reduced pressure. The crude product was purified by recrystallization from hexane:DCM (1:1, 90 mL) then filtered. The brown liquid filtrate was concentrated and recrystallized as before. The combined solids were then purified by column chromatography on silica gel with DCM:Hexane to obtain 2,4,5,6-tetra(9H-carbazol-9-yl)isophthalonitrile (**4a**, 1.14 g, 1.45 mmol, 73 % yield) as a bright yellow crystalline solid.

Analytical data is consistent with literature values. [1]

Rf (Hexane;DCM 1:1) = 0.29.

^{1}H NMR (400 MHz, CDCl$_3$) δ/ppm: 8.22 (d, *J* = 7.7 Hz, 2H, Ar*H*), 7.76 – 7.65 (m, 8H, Ar*H*), 7.49 (t, *J* = 7.2 Hz, 2H, Ar*H*), 7.33 (d, *J* = 7.7 Hz, 2H, Ar*H*), 7.22 (d, *J* = 7.5 Hz, 4H, Ar*H*), 7.16 – 7.03 (m, 8H, Ar*H*), 6.82 (t, *J* = 7.9 Hz, 4H, Ar*H*), 6.63 (t, *J* = 7.6 Hz, 2H, Ar*H*).

^{13}C {^{1}H} NMR (101 MHz, CDCl$_3$) δ/ppm: 145.2, 144.6, 140.0, 138.2, 136.9, 134.7, 127.0, 125.8, 124.9, 124.7, 124.5, 123.8, 122.4, 121.9, 121.4, 121.0, 120.4, 119.6, 116.3, 111.6, 109.9, 109.5, 109.4.

2,4,5,6-Tetrakis(3,6-dichloro-9H-carbazol-9-yl)isophthalonitrile (4ClCzIPN, 4b)

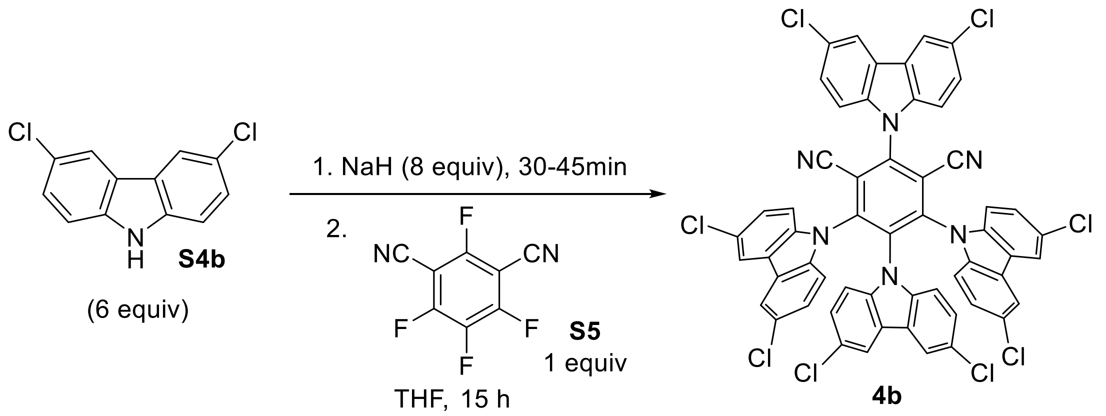

Sodium hydride (60% suspension in mineral oil, 0.320 g, 8.00 mmol, 8.0 equiv) was added slowly to a stirred solution of 3,6-dichloro-9H-carbazole **S4b** (1.96 g, 6.00 mmol, 6.0 equiv) in dry THF (20 mL) under a nitrogen atmosphere at RT After 30 min, 2,4,5,6-tetrafluoroisophthalonitrile **S5** (200 mg, 1.00 mmol) was added. After stirring at RT for 15 h, 1 mL water was added to the reaction mixture to quench the excess of NaH. The resulting mixture was then concentrated under reduced pressure. The crude product was purified by recrystallization from hexane:DCM (1:2, 80 mL) then filtered. The brown liquid filtrate was concentrated and recrystallized as before. The combined solids were then purified by column chromatography on silica gel with DCM:Hexane to obtain 2,4,5,6-tetrakis(3,6-*d*ichloro-9H-carbazol-9-yl)isophthalonitrile (**4b**, 830 mg, 0.780 mmol, 87 % yield) as a bright yellow crystalline solid.

Analytical data is consistent with literature values. [1]

Rf (Hexane;DCM 1:1) = 0.25.

^{1}H NMR (400 MHz, CDCl$_3$) δ/ppm: 8.60 (d, *J* = 2.1 Hz, 2H, Ar*H*), 8.15 (d, *J* = 2.1 Hz, 4H, Ar*H*), 8.08 (d, *J* = 8.8 Hz, 2H, Ar*H*), 7.87 (dd, *J* = 8.8, 2.1 Hz, 2H, Ar*H*), 7.80 (d, *J* = 2.2 Hz, 2H, Ar*H*), 7.69 (d, *J* = 8.8 Hz,

4H, Ar*H*), 7.46 (d, *J* = 8.8 Hz, 2H, Ar*H*), 7.32 (dd, *J* = 8.8, 2.2 Hz, 4H, Ar*H*), 6.93 (dd, *J* = 8.8, 2.2 Hz, 2H, Ar*H*).

^{13}C {^{1}H} NMR (101 MHz, $CDCl_3$) δ/ppm: 145.0, 144.5, 138.5, 137.4, 136.5, 135.8, 134.5, 127.8, 127.0, 126.4, 125.7, 125.3, 124.2, 123.8, 123.3, 121.6, 120.9, 120.3, 116.8, 112.6, 112.5, 112.3, 111.7.

6.1.3. Synthesis of Homoallylic alcohols and amides

6.1.3.1. Tertiary Alcohols

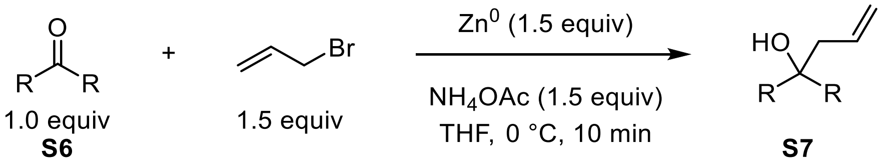

General Procedure 1

Following a reported procedure,[3] a round-bottomed flask was charged with zinc powder (1.5 equiv), NH_4OAc (1.5 equiv) and ketone (1.0 equiv), if solid. The flask was evacuated and backfilled with N_2 three times. THF (0.25 M) and ketone (1.0 equiv), if liquid, were added and the suspension was cooled with an ice bath. Allyl bromide (1.5 equiv) was then added dropwise and the reaction mixture was stirred at this temperature for 10 min. The reaction was then quenched by sat. aq. $NaHCO_3$ (1 volume), allowed to warm to room temperature and stirred for 30 min. EtOAc (1 volume) and H_2O (1 volume) were added and the layers were separated. The aqueous layer was extracted with EtOAc (3 x 1 volume) and the combined organic layers were dried over $MgSO_4$, filtered and the solvents were removed under reduced pressure. The crude compound was used as is in the next step.

1-Allylcyclohexan-1-ol (S7a)

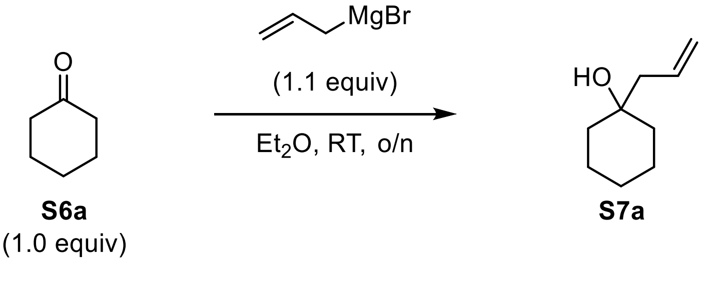

A three-necked 500 mL round-bottomed flask equipped with a magnetic stirring bar and a dropping funnel was evacuated and backfilled with N_2 three times. Cyclohexanone (**S6a**, 3.93 g, 4.20 mL, 40.0 mmol, 1.0 equiv) was added, followed by Et_2O (200 mL). The solution was cooled with an ice bath. Allylmagnesium bromide (1.0 M in Et_2O, 44 mL, 44 mmol, 1.1 equiv) was added dropwise *via* the dropping funnel at this temperature. The suspension was allowed to warm to RT and stirred overnight.

---

[3] Weires, N. A.; Slutskyy, Y.; Overman, L. E. Facile Preparation of Spirolactones by an Alkoxycarbonyl Radical Cyclization–Cross-coupling Cascade. *Angew. Chem. Int. Ed.* **2019**, *58* (25), 8561–8565.

The reaction mixture was cooled with an ice bath and quenched with NH_4Cl (100 mL) and H_2O (50 mL) was added to dissolve the salts formed. The layers were separated and the aqueous layer was extracted with Et_2O (3x100 mL). The combined organic layers were washed with H_2O (200 mL) and brine (200 mL), dried over $MgSO_4$, filtered and the solvents were removed under reduced pressure.

The crude was purified by flash chromatography (10% Et_2O in Pentane) obtaining 1-allylcyclohexan-1-ol (**S7a**, 4.36 g, 31.1 mmol, 78% yield) as a colorless liquid.

Analytical data is consistent with literature values. [3]

Rf (1:9 Et_2O:Pentane) = 0.4

^{1}H NMR (400 MHz, $CDCl_3$) δ/ppm: 5.89 (ddt, *J* = 17.6, 10.2, 7.5 Hz, 1H, C*H*=CH_2), 5.18 – 5.04 (m, 2H, CH=C*H_2*), 2.21 (dt, *J* = 7.5, 1.1 Hz, 2H, C*H_2*), 1.68 – 1.35 (m, 10H, C*H_2*), 1.33 – 1.20 (m, 1H, C*H_2*).

^{13}C {^{1}H} NMR (101 MHz, $CDCl_3$) δ/ppm: 133.9, 118.8, 71.1, 46.9, 37.5, 25.9, 22.3.

4-Allyltetrahydro-2*H*-pyran-4-ol (S7b)

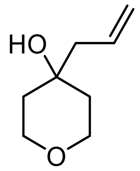

S7b was prepared according to *General Procedure 1* from tetrahydropyran-4-one (**S6b**, 0.46 mL, 5.0 mmol, 1.0 equiv) using Zinc powder (490 mg, 7.50 mmol, 1.5 equiv), NH_4OAc (578 mg, 7.50 mmol, 1.5 equiv) and 3-bromopropen (0.65 mL, 7.5 mmol, 1.5 equiv); obtaining 4-allyltetrahydro-2H-pyran-4-ol (**S7b**, 640 mg, 4.50 mmol, 90%) as a colorless oil.

Analytical data is consistent with literature values. [3]

^{1}H NMR (400 MHz, $CDCl_3$) δ/ppm: 5.87 (ddt, *J* = 17.0, 10.2, 7.5 Hz, 1H, C*H*=CH_2), 5.27 – 5.09 (m, 2H, CH=C*H_2*), 3.84 – 3.67 (m, 4H, C*H_2*), 2.24 (dt, *J* = 7.5, 1.1 Hz, 2H, C*H_2*), 1.70 (ddd, *J* = 13.7, 10.4, 5.9 Hz, 2H, C*H_2*), 1.57 – 1.41 (m, 3H, C*H_2*).

^{13}C {^{1}H} NMR (101 MHz, $CDCl_3$) δ/ppm: 132.6, 120.0, 68.5, 64.0, 47.6, 37.7.

***Tert*-butyl 4-allyl-4-hydroxypiperidine-1-carboxylate (S7c)**

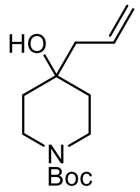

S7c was prepared according to *General Procedure 1* from *N*-Boc-4-piperidone (**S6c**, 1.92 g, 10.0 mmol, 1.0 equv) using Zinc powder (981 mg, 15.0 mmol, 1.5 equiv), NH_4OAc (1.16 g, 15.0 mmol, 1.5 equiv) and 3-bromopropen (1.3 mL, 15 mmol, 1.5 equiv); obtaining *tert*-butyl 4-allyl-4-hydroxypiperidine-1-carboxylate (**S7c**, 2.41 g, 9.99 mmol, 100%) as a colorless oil.

Analytical data is consistent with literature values[3]

^{1}H NMR (400 MHz, $CDCl_3$) δ/ppm: 5.85 (ddt, *J* = 17.0, 10.2, 7.5 Hz, 1H, C*H*=CH_2), 5.30 – 5.02 (m, 2H, CH=CH_2), 3.89 – 3.61 (s, 1H, CH_2), 3.30 – 3.06 (m, 2H, CH_2), 2.22 (dt, *J* = 7.6, 1.1 Hz, 2H, CH_2), 1.62 – 1.48 (m, 5H, CH_2), 1.44 (s, 9H, CH_3).

^{13}C {^{1}H} NMR (101 MHz, $CDCl_3$) δ/ppm: 155.0, 132.8, 119.9, 79.5, 69.3, 47.4, 39.8, 36.8, 28.6.

1-Allylcyclopentan-1-ol (S7d)

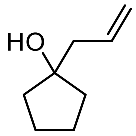

S7d was prepared according to *General Procedure 1* from cyclopentanone (**S6d**, 0.44 mL, 5.0 mmol, 1.0 equv) using Zinc powder (490 mg, 7.50 mmol, 1.5 equiv), NH_4OAc (578 mg, 7.50 mmol, 1.5 equiv) and 3-bromopropen (0.65 mL, 7.5 mmol, 1.5 equiv); obtaining allylcyclopentanol (**S7d**, 422 mg, 3.34 mmol, 67%) as a colorless oil.

Analytical data is consistent with literature values. [3]

^{1}H NMR (400 MHz, $CDCl_3$) δ/ppm: 5.97 – 5.82 (m, 1H, C*H*=CH_2), 5.21 – 5.06 (m, 2H, CH=CH_2), 2.34 (dt, *J* = 7.4, 1.2 Hz, 2H, CH_2), 1.89 – 1.71 (m, 2H, CH_2), 1.71 – 1.53 (m, 6H, CH_2).

^{13}C {^{1}H} NMR (101 MHz, $CDCl_3$) δ/ppm: 134.7, 118.7, 81.5, 46.0, 39.6, 24.0.

1-Allylcyclododecan-1-ol (S7e)

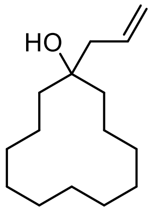

S7e was prepared according to *General Procedure 1* from cyclododecanone (**S6e**, 912 mg, 5.00 mmol, 1.0 equv) using Zinc powder (490 mg, 7.50 mmol, 1.5 equiv), NH_4OAc (578 mg, 7.50 mmol, 1.5 equiv) and 3-bromopropen (0.65 mL, 7.5 mmol, 1.5 equiv); obtaining allylcyclododecanol (**S7e**, 1.11 g, 4.95 mmol, 99%) as a white solid.

Analytical data is consistent with literature values. [3]

^{1}H NMR (400 MHz, $CDCl_3$) δ/ppm: 5.91 (ddt, *J* = 17.6, 10.2, 7.4 Hz, 1H, C*H*=CH_2), 5.25 – 5.04 (m, 2H, CH=CH_2), 2.17 (dt, *J* = 7.5, 1.2 Hz, 2H, CH_2), 1.48 – 1.24 (m, 22H, CH_2).

^{13}C {^{1}H} NMR (101 MHz, $CDCl_3$) δ/ppm: 134.0, 118.9, 74.8, 45.4, 34.5, 26.6, 26.2, 22.7, 22.2, 19.6.

2-Allyladamantan-2-ol (S7f)

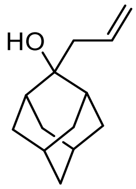

S7f was prepared according to *General Procedure 1* from adamantan-2-one (**S6f**, 751 mg, 5.00 mmol, 1.00 equiv) using Zinc powder (490 mg, 7.50 mmol, 1.5 equiv), NH_4OAc (578 mg, 7.50 mmol, 1.5 equiv) and 3-bromopropen (0.65 mL, 7.5 mmol, 1.5 equiv); obtaining 2-allyladamantan-2-ol (**S7f**, 874 mg, 4.55 mmol, 91%) as a white solid.

Analytical data is consistent with literature values. [3]

^{1}H NMR (400 MHz, $CDCl_3$) δ/ppm: 5.90 (ddt, *J* = 16.9, 10.3, 7.5 Hz, 1H, C*H*=CH$_2$), 5.21 – 5.09 (m, 2H, CH=C*H*$_2$), 2.49 – 2.41 (m, 2H, C*H*$_2$), 2.20 (dd, *J* = 12.7, 3.0 Hz, 2H, Ad*H*), 1.94 – 1.77 (m, 4H, Ad*H*), 1.75 – 1.67 (m, 6H, Ad*H*), 1.64 (s, 1H, O*H*), 1.58 – 1.51 (m, 2H, Ad*H*).

^{13}C {^{1}H} NMR (101 MHz, $CDCl_3$) δ/ppm: 133.9, 119.0, 74.6, 42.9, 38.5, 37.2 (2C), 34.6 (2C), 33.1 (2C), 27.6, 27.5.

***tert*-Butoxy dehydroepiandrosterone (S6g)**

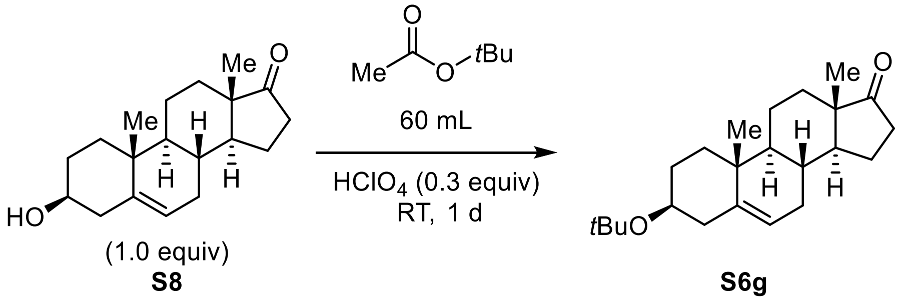

The reaction was carried out in an open flask.

To a solution of dehydroepiandrosterone (**S8**, 865 mg, 3.00 mmol, 1.0 equiv) in *tert*-butyl acetate (60 mL) perchloric acid (129 mg, 0.0800 mL, 0.900 mmol, 0.3 equiv) was added. The reaction mixture was stirred for one day, then quenched with Na_2CO_3 (636 mg, 6.00 mmol, 2.0 equiv) and stirred for 40 min. The solids were filtered off and the solvent was removed under reduced pressure. The crude was purified by flash chromatography (5 to 10% Et_2O in Pentane) obtaining *tert*-butoxy dehydroepiandrosterone (**S6g**, 539 mg, 1.56 mmol, 52% yield) as a white solid.

Analytical data is consistent with literature values.[4]

[4] Freerksen, R. W.; Pabst, W. E.; Raggio, M. L.; Sherman, S. A.; Wroble, R. R.; Watt, D. S. Photolysis of α-Peracetoxynitriles. 2. A Comparison of Two Synthetic Approaches to 18-Cyano-20-Ketosteroids. *J. Am. Chem. Soc.* **1977**, *99* (5), 1536–1542.

Rf (2:8 Et_2O:Pentane) = 0.6.

^{1}H NMR (400 MHz, $CDCl_3$) δ/ppm: 5.34 (dt, *J* = 5.1, 1.9 Hz, 1H, C=C*H*), 3.31 (tt, *J* = 11.2, 4.7 Hz, 1H, *t*BuO-C*H*), 2.52 – 2.39 (m, 1H), 2.29 (tq, *J* = 11.3, 2.7 Hz, 1H), 2.20 – 2.01 (m, 3H), 2.00 – 1.89 (m, 1H), 1.88 – 1.79 (m, 2H), 1.75 – 1.40 (m, 8H), 1.34 – 1.23 (m, 2H), 1.19 (s, 9H, C(CH_3)$_3$), 1.09 (td, *J* = 13.6, 3.9 Hz, 1H), 1.02 (s, 3H, CH_3), 0.88 (s, 3H, CH_3).

^{13}C {^{1}H} NMR (101 MHz, $CDCl_3$) δ/ppm: 221.3, 142.4, 120.3, 73.6, 71.4, 52.0, 50.6, 47.7, 42.2, 37.9, 36.9, 36.0, 31.7, 31.6, 31.4, 31.0, 28.6, 22.0, 20.5, 19.5, 13.7.

3β-*tert*-butoxy-17α-allyl-dehydroepiandrostane-17β-ol (S7g)

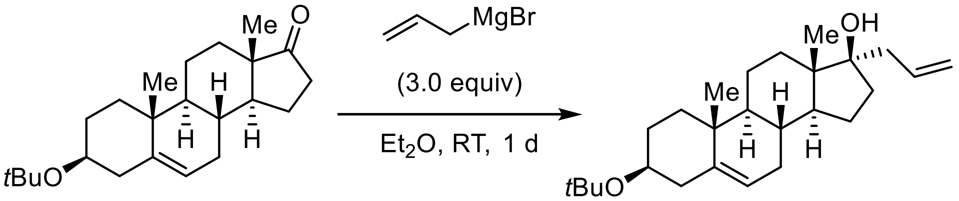

(1.0 equiv)
S6g **S7g**

A two-necked 50 mL round-bottomed flask equipped with a stirring bar was charged with *tert*-butoxydehydroepiandrosterone (**S6g**, 413 mg, 1.20 mmol, 1.0 equiv). The flask was evacuated and backfilled with N_2 three times. Et_2O (10 mL) was added, followed by allylmagnesium bromide (1.0 M in Et_2O, 3.6 mL, 3.60 mmol, 3.0 equiv). The suspension was stirred at room temperature for one day, then cooled with an ice bucket and quenched with NH_4Cl (10 mL). The layers were separated and the aqueous layer was extracted with Et_2O (3 x 15 mL). The combined organic layers were washed with brine, dried over $MgSO_4$, filtered and the solvents were removed under reduced pressure. The crude was purified by flash chromatography (2 to 10% EtOAc in Pentane) to obtain 3β-*tert*-butoxy-17α-allyl-dehydroepiandrostane-17β-ol (**S7g**, 342 mg, 0.885 mmol, 74% yield) as a white amorphous solid.

Rf (1:9 EtOAc:Pentane) = 0.3.

^{1}H NMR (400 MHz, $CDCl_3$) δ/ppm: 5.99 (ddt, *J* = 17.3, 10.2, 7.1 Hz, 1H, C*H*=CH_2), 5.31 (dt, *J* = 5.0, 1.8 Hz, 1H, C=C*H*), 5.25 – 5.10 (m, 2H, CH=CH_2), 3.30 (tt, *J* = 11.2, 4.7 Hz, 1H, *t*BuO-C*H*), 2.38 – 2.23 (m, 2H), 2.20 (dd, *J* = 13.7, 7.3 Hz, 1H), 2.14 (ddd, *J* = 13.5, 5.0, 2.3 Hz, 1H), 2.07 – 1.89 (m, 2H), 1.83 (dt, *J* = 13.2, 3.6 Hz, 1H), 1.73 – 1.41 (m, 12H), 1.38 – 1.22 (m, 2H), 1.19 (s, 9H, C(CH_3)$_3$), 1.07 (td, *J* = 13.5, 3.9 Hz, 1H), 1.01 (s, 3H, CH_3), 0.90 (s, 3H, CH_3).

^{13}C {^{1}H} NMR (101 MHz, $CDCl_3$) δ/ppm: 142.2, 135.1, 120.8, 119.3, 82.6, 73.5, 71.6, 51.2, 50.4, 46.1, 42.2, 41.9, 38.0, 36.9, 35.2, 32.9, 32.0, 31.9, 31.5, 28.6, 24.0, 20.9, 19.5, 14.4.

HRMS (Sicrit plasma/LTQ-Orbitrap) m/z: $[M + H]^+$ Calcd for $C_{26}H_{43}O_2^+$ 387.3258; Found 387.3258.

1-(But-3-en-1-yl)cyclohexanol (S9)

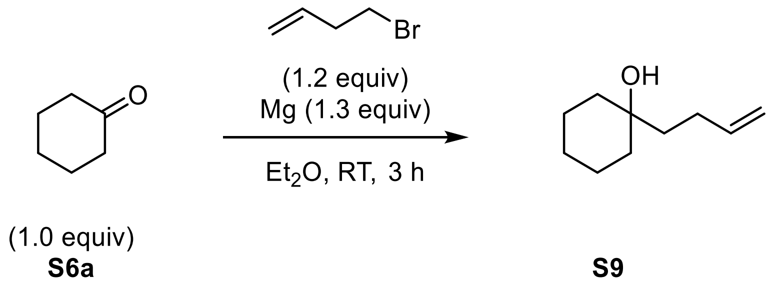

In a sealed 20 mL round-bottomed vial, magnesium turnings (0.305 g, 12.5 mmol, 1.3 equiv.) were suspended in diethyl ether (dry; 8.6 mL) together with a crystal of iodine. To the resulting yellow suspension, 4-bromobut-1-ene (1.2 mL, 1.6 mmol, 1.2 equiv.) was added via syringe at room temperature. During the addition, the mixture was not stirred until it was completely decolored. After that, stirring was commenced, with visible reflux. When the addition was complete, stirring was continued at 40 °C for another hour. To the resulting pale yellow solution, a solution of cyclohexanone (**S6a**, 1.0 mL, 9.6 mmol, 1.0 equiv.) in Et_2O (4.3 mL) was added dropwise at room temperature. The resulting turbid mixture was stirred for 3 hours and then quenched by pouring it into cold sat. aq. NH_4Cl. The water layer was then extracted with ether (3 x 40 mL). The combined organic extracts were dried over $MgSO_4$, filtered, and concentrated under vacuum. Column chromatography of the obtained pale orange crude oil (SiO_2; EtOAc in pentane, 5%) afforded the desired 1-(but-3-en-1-yl)cyclohexanol (**S9**, 0.798 g, 5.17 mmol, 54% yield) as a colorless oil.

Analytical data is consistent with literature values.[5]

[5] Nicolai, S.; Waser, J. Pd(0)-Catalyzed Oxy- and Aminoalkynylation of Olefins for the Synthesis of Tetrahydrofurans and Pyrrolidines. *Org. Lett.* **2011**, *13* (23), 6324–6327.

6.1.3.2. Secondary Alcohols

3-Cyclopenten-1-ol (**S7j**) was purchased from Fluorochem and used as received.

1-Phenylhex-5-en-3-ol (S7h)

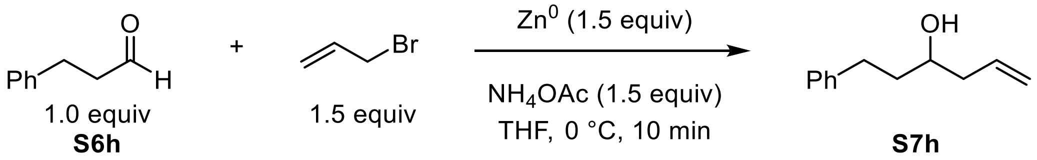

Following a reported procedure,[3] a round-bottomed flask was charged with zinc powder (490 mg, 7.50 mmol, 1.5 equiv) and NH_4OAc (578 mg, 7.50 mmol, 1.5 equiv). The flask was evacuated and backfilled with N_2 three times. THF (20 mL) and hydrocynnamaldehyde (**S6h**, 0.67 g, 0.70 mL, 5.0 mmol, 1.0 equiv) were added and the suspension was cooled with an ice bath. Allyl bromide (0.91 g, 0.70 mL, 7.5 mmol, 1.5 equiv) was then added dropwise and the reaction mixture was stirred at this temperature for 10 min. The reaction was then quenched by sat. aq. $NaHCO_3$ (20 mL), allowed to warm to room temperature and stirred for 30 min. EtOAc (20 mL) and H_2O (20 mL) were added and the layers were separated. The aqueous layer was extracted with EtOAc (3 x 20 mL) and the combined organic layers were dried over $MgSO_4$, filtered and the solvents were removed under reduced pressure to obtain 1-phenylhex-5-en-3-ol (**S7h**, 847 mg, 4.81 mmol, 96% yield) as a pale-yellow oil. The crude compound was used as is in the next step.

Analytical data is consistent with literature values.[6]

^{1}H NMR (400 MHz, $CDCl_3$) δ/ppm: 7.32 – 7.26 (m, 2H, Ar*H*), 7.24 – 7.15 (m, 3H, Ar*H*), 5.92 – 5.75 (m, 1H, C*H*=CH$_2$), 5.15 (dtd, *J* = 13.1, 2.6, 1.1 Hz, 2H, CH=C*H*$_2$), 3.68 (ddt, *J* = 12.1, 7.7, 4.4 Hz, 1H, C*H*), 2.82 (ddd, *J* = 13.7, 8.7, 6.8 Hz, 1H, C*H*$_2$), 2.75 – 2.65 (m, 1H, C*H*$_2$), 2.33 (dddt, *J* = 13.6, 6.8, 4.3, 1.3 Hz, 1H, C*H*$_2$), 2.19 (dtt, *J* = 13.9, 7.9, 1.1 Hz, 1H, C*H*$_2$), 1.85 – 1.75 (m, 2H, C*H*$_2$), 1.62 (d, *J* = 4.2 Hz, 1H, O*H*).

^{13}C {^{1}H} NMR (101 MHz, $CDCl_3$) δ/ppm: 142.2, 134.7, 128.6, 128.5, 125.9, 118.4, 70.0, 42.2, 38.6, 32.2.

[6] Wang, T.; Hao, X.-Q.; Huang, J.-J.; Niu, J.-L.; Gong, J.-F.; Song, M.-P. Chiral Bis(Imidazolinyl)Phenyl NCN Pincer Rhodium(III) Catalysts for Enantioselective Allylation of Aldehydes and Carbonyl–Ene Reaction of Trifluoropyruvates. *J. Org. Chem.* **2013**, *78* (17), 8712–8721.

***trans*-Vinylcyclohexanol (S7i)**

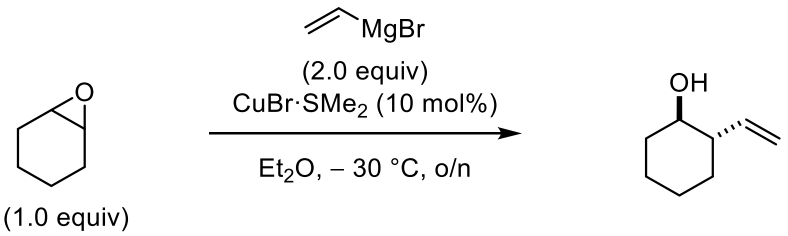

Following a reported procedure,[7] an oven-dried 250 mL three-necked round bottomed flask equipped with a magnetic stirring bar and a 50 mL dropping funnel was evacuated and backfilled three times with N_2. CuBr·Me_2S (206 mg, 1.00 mmol, 10 mol%) was added, followed by Et_2O (5.0 mL). The suspension was then cooled to – 40 °C. Cyclohexene oxide (**S10**, 1.0 mL, 10 mmol, 1.0 equiv) was added to the suspension, followed by the dropwise addition of vinylmagnesium bromide (1.0 M in THF, 20 mL, 20.0 mmol, 2.0 equiv). The reaction mixture was then allowed to warm to – 30 °C and stirred overnight. The mixture was ten quenched with sat. aq. NH_4Cl (basified to pH = 8 with ammonia, 30 mL) and the layers were separated. The aqueous layer was extracted with Et_2O (3 x 30 mL), the combined organic layer was washed with brine (30 mL), dried over $MgSO_4$ and the solvents were removed under reduced pressure to obtain trans-2-vinylcyclohexanol (**S7i**, 1.26 g, 9.98 mmol, 100%) as a yellow oil. The crude was used in the next step without further purification.

Analytical data is consistent with literature values.[8]

^{1}H NMR (400 MHz, $CDCl_3$) δ/ppm: 5.68 (ddd, *J* = 17.2, 10.2, 8.7 Hz, 1H, C*H*=CH2), 5.27 – 5.05 (m, 2H, CH=C*H*2), 3.29 – 3.19 (m, 1H, C*H*OH), 2.09 – 2.01 (m, 1H, C*H*), 1.93 – 1.87 (m, 1H, C*H*2), 1.79 – 1.71 (m, 2H, C*H*2), 1.70 – 1.62 (m, 1H, C*H*2), 1.31 – 1.16 (m, 4H, C*H*2).

^{13}C {^{1}H} NMR (101 MHz, $CDCl_3$) δ/ppm: 140.9, 116.7, 72.9, 51.3, 34.0, 31.2, 25.2, 24.9.

6.1.3.3. Primary Alcohols

3-Butenol (**S7k**) was purchased from Acros Organics and used as received. *cis*-3-Hexenol (**S7l**) was purchased from Sigma Aldrich and used as received.

[7] Tobia, D.; Rickborn, B. Kinetics and Stereochemistry of LiNR2-Induced 1,2-Elimination of Homoallylic Ethers. *J. Org. Chem.* **1989**, *54* (4), 777–782.
[8] Launay, G. G.; Slawin, A. M. Z.; O'Hagan, D. Prins Fluorination Cyclisations: Preparation of 4-Fluoro-Pyran and - Piperidine Heterocycles. *Beilstein J. Org. Chem.* **2010**, *6*. https://doi.org/10.3762/bjoc.6.41.

3-Cyclohexylidenepropanoic acid (S11)

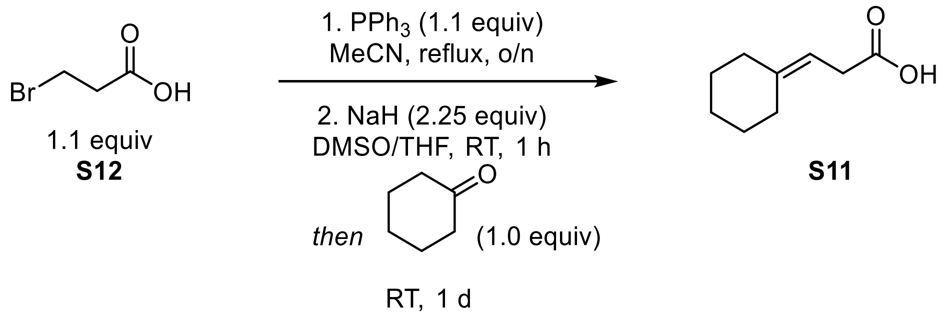

A 25 mL round-bottomed flask equipped with a magnetic stirring bar and a reflux condenser was charged with 3-bromopropionic acid (**S12**, 1.68 g, 11.0 mmol, 1.1 equiv), triphenylphosphine (2.89 g, 11.0 mmol, 1.1 equiv) and acetonitrile (11 mL). The mixture was refluxed overnight, allowed to cool to room temperature and then the solvents were removed under reduced pressure. The crude phosphonium bromide was dried at high vacuum for 2 to 3 h.

In a 100 mL round-bottomed flask, the crude phosphonium bromide was dissolved in DMSO (10 mL). THF was added and the solution was homogenized for 5 min. NaH (60% in mineral oil, 900 mg, 22.5 mmol, 2.25 equiv) was added portionswise and the suspension was stirred for 1 h. Cyclohexanone (981 mg, 1.04 mL, 10.0 mmol, 1.0 equiv) was added and the reaction mixture was stirred for 1 day. The reaction was then quenched with aq. HCl (1.0 M, 30 mL), the layers were removed and the aqueous layer was extracted with EtOAc (3 x 50 mL). The combined organic layers were dried over $MgSO_4$, filtered and the solvents were removed under reduced pressure.

The crude was purified by flash chromatography (1:3 EtOAc in Pentane) obtaining 3-cyclohexylidenepropanoic acid (**S11**, 888 mg, 5.76 mmol, 58% yield) as a yellow oil.

Analytical data is consistent with literature values.[9]

Rf (1:3 EtOAc:Pentane) = 0.2

^{1}H NMR (400 MHz, $CDCl_3$) δ/ppm: 5.24 (ddt, *J* = 7.2, 5.9, 1.3 Hz, 1H, C*H*=C), 3.09 (d, *J* = 7.2 Hz, 2H, CH_2), 2.19 – 2.08 (m, 4H, CH_2), 1.61 – 1.46 (m, 6H, CH_2).

^{13}C {^{1}H} NMR (101 MHz, $CDCl_3$) δ/ppm: 178.4, 144.3, 111.7, 37.1, 32.7, 29.1, 28.5, 27.6, 26.8.

[9] Schelwies, M.; Paciello, R.; Pelzer, R.; Siegel, W.; Breuer, M. Palladium‐catalyzed Low Pressure Carbonylation of Allylic Alcohols by Catalytic Anhydride Activation. *Chemistry* **2021**, *27* (36), 9263–9266.

3-Cyclohexylidenepropan-1-ol (S7m)

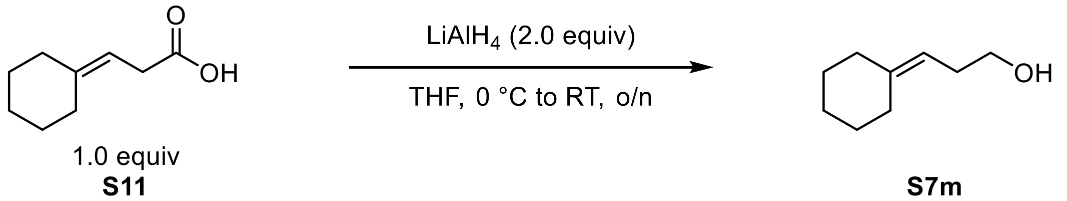

In an inert 25 mL pear-shaped flask, 3-cyclohexylidenepropanoic acid (**S11**, 617 mg, 4.00 mmol, 1.0 equiv) was diluted in THF (10 mL). A 50 mL two-necked flask equipped with a magnetic stirring bar was evacuated and backfilled with N_2 three times. $LiAlH_4$ (304 mg, 8.00 mmol, 2.0 equiv) was added against the N_2 flow, followed by THF (10 mL). The suspension was cooled to 0 °C and then the carboxylic acid solution was added dropwise. The reaction mixture was allowed to warm to RT and stirred overnight.

The suspension was diluted with Et_2O (10 mL) and cooled with an ice bath. At this temperature, 0.3 mL of H_2O were added, followed by 0.3 mL of 15% aq. NaOH, followed by 0.9 mL of H_2O. The reaction was then allowed to warm to RT and it was stirred for 15 min. $MgSO_4$ was added and the mixture was stirred for 15 min, filtered and the solvents were removed under reduced pressure. The crude was purified by flash chromatography (10% EtOAc in Pentane) obtaining 3-cyclohexylidenepropan-1-ol (**S7m**, 167 mg, 1.19 mmol, 30% yield) as a colorless oil.

Analytical data is consistent with literature values.[10]

Rf (1:9 EtOAc:Pentane) = 0.5.

^{1}H NMR (400 MHz, $CDCl_3$) δ/ppm: 5.07 (tt, *J* = 7.5, 1.3 Hz, 1H, C*H*=C), 3.60 (t, *J* = 6.5 Hz, 2H, C*H*$_2$), 2.28 (q, *J* = 6.8 Hz, 2H, C*H*$_2$), 2.20 – 2.04 (m, 4H, C*H*$_2$), 1.61 – 1.44 (m, 6H, C*H*$_2$).

^{13}C {^{1}H} NMR (101 MHz, $CDCl_3$) δ/ppm: 143.8, 116.6, 62.7, 37.4, 30.8, 29.0, 28.9, 28.1, 27.0.

6.1.3.4. Primary Amides

***N*-(but-3-en-1-yl)-4-methylbenzenesulfonamide (S7n)**

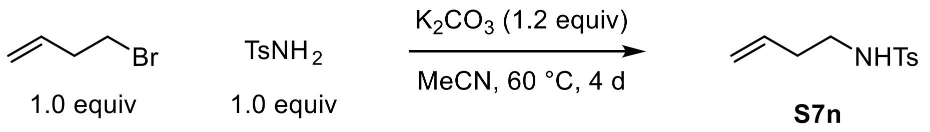

The reaction was performed in an open flask.

Following a reported procedure,[11] a 250 mL round-bottomed flask equipped with a magnetic stirring bar and a reflux condenser was charged with 4-methylbenzenesulfonamide (3.42 g, 20.0 mmol, 1.0 equiv) and potassium carbonate (3.32 g, 24.0 mmol, 1.2 equiv). MeCN (80 mL) and 4-bromobut-1-ene (2.7 g, 2.0 mL,

---

[10] Fanourakis, A.; Hodson, N. J.; Lit, A. R.; Phipps, R. J. Substrate-Directed Enantioselective Aziridination of Alkenyl Alcohols Controlled by a Chiral Cation. *J. Am. Chem. Soc*. **2023**, *145* (13), 7516–7527.
[11] Lucas, E. L.; Hewitt, K. A.; Chen, P.-P.; Castro, A. J.; Hong, X.; Jarvo, E. R. Engaging Sulfonamides: Intramolecular Cross-Electrophile Coupling Reaction of Sulfonamides with Alkyl Chlorides. *J. Org. Chem.* **2020**, *85* (4), 1775–1793.

20 mmol, 1.0 equiv) were added and the reaction mixture was stirred at 60 °C for 4 days. The reaction was allowed to cool to RT and it was quenched with sat. aq. NH_4Cl (80 mL). EtOAc (40 mL) was added and the layers were separated. The aqueous layer was extracted with EtOAc (3 x 80 mL), the combined organic layers were washed with brine (50 mL), dried over $MgSO_4$, filtered and the solvents were removed under reduced pressure. The crude was purified by flash chromatography (20% EtOAc in Pentane) obtaining N-(but-3-en-1-yl)-4-methylbenzenesulfonamide (**S7n**, 2.24 g, 9.94 mmol, 50% yield) as a clear oil.

Analytical data is consistent with literature values.[11]

Rf (2:8 EtOAc:Pentane) = 0.5

^{1}H NMR (400 MHz, $CDCl_3$) δ/ppm: 7.82 – 7.69 (m, 2H, Ar*H*), 7.34 – 7.28 (m, 2H, Ar*H*), 5.62 (ddt, *J* = 17.1, 10.3, 6.9 Hz, 1H, C*H*=CH_2), 5.12 – 4.95 (m, 2H, C*H*=CH_2), 4.49 (t, *J* = 6.1 Hz, 1H, C*H*2), 3.02 (q, *J* = 6.5 Hz, 2H, C*H*2), 2.43 (s, 3H, C*H*3), 2.20 (qt, *J* = 6.7, 1.3 Hz, 2H, C*H*2).

^{13}C {^{1}H} NMR (101 MHz, $CDCl_3$) δ/ppm: 143.6, 137.1, 134.3, 129.8, 127.3, 118.3, 42.2, 33.7, 21.7.

(*Z*)-2-(hex-3-en-1-yl)isoindoline-1,3-dione (S13)

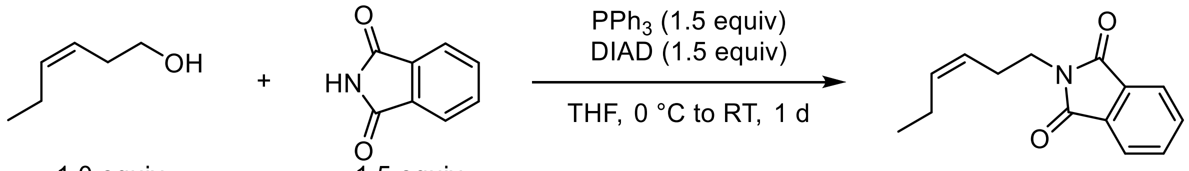

1.0 equiv
S7l

1.5 equiv

S13

A 50 mL two-necked round bottomed flask equipped with a magnetic stirring bar was charged with phthalimide (662 mg, 4.50 mmol, 1.5 equiv) and triphenylphosphine (1.18 g, 4.50 mmol, 1.5 equiv). The flask was evacuated and backfilled with N_2 three times. THF (12 mL) and (*Z*)-hex-3-en-1-ol (**S7l**, 501 mg, 0.600 mL, 5.00 mmol, 1.0 equiv) were added and the suspension was cooled with an ice bath. DIAD (910 mg, 0.900 mL, 4.50 mmol, 1.5 equiv) was added dropwise. The reaction mixture was then allowed to warm to RT and it was stirred for 1 day. The solvents were then removed and the crude was purified by flash chromatography (1 to 10% Et_2O in pentane) obtaining 2-[(*Z*)-hex-3-enyl]isoindole-1,3-dione (**S13**, 900 mg, 3.93 mmol, 79% yield) as a colorless oil.

Analytical data is consistent with literature values.[12]

Rf (5% Et_2O in Pentane) = 0.4

^{1}H NMR (400 MHz, $CDCl_3$) δ/ppm: 7.83 (dd, *J* = 5.4, 3.1 Hz, 2H, Ar*H*), 7.70 (dd, *J* = 5.5, 3.0 Hz, 2H, Ar*H*), 5.53 – 5.40 (m, 1H, C=C*H*), 5.33 (dtt, *J* = 10.6, 7.4, 1.5 Hz, 1H, C=C*H*), 3.72 (t, *J* = 7.2 Hz, 2H, C*H*2), 2.54 – 2.28 (m, 2H, C*H*2), 1.99 (pd, *J* = 7.5, 1.5 Hz, 2H, C*H*2), 0.86 (t, *J* = 7.5 Hz, 3H, C*H*3).

^{13}C {^{1}H} NMR (101 MHz, $CDCl_3$) δ/ppm: 168.5, 135.0, 134.0, 132.3, 124.3, 123.3, 37.8, 26.5, 20.6, 14.3.

[12] Wang, J.-W.; Liu, D.-G.; Chang, Z.; Li, Z.; Fu, Y.; Lu, X. Nickel‐catalyzed Switchable Site‐selective Alkene Hydroalkylation by Temperature Regulation. *Angew. Chem. Int. Ed.* **2022**, *61* (31), e202205537.

(*Z*)-Hex-3-en-1-amine (S14)

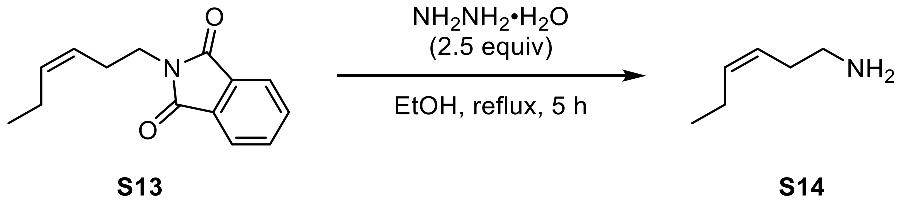

The reaction was performed in an open flask.

A 25 mL round-bottomed flask equipped with a magnetic stirring bar and a reflux condenser was charged with 2-[(*Z*)-hex-3-enyl]isoindole-1,3-dione (**S13**, 688 mg, 3.00 mmol, 1.0 equiv) and ethanol (10 mL). Hydrazine hydrate (0.37 g, 0.40 mL, 7.5 mmol, 2.5 equiv) was added and the reaction mixture was refluxed for 5 h (a white precipitate formed). The reaction was then allowed to cool to room temperature, the precipitate was filtered off and the solvent was removed under reduced pressure. The residue was then diluted with DCM (15 mL), H_2O (15 mL) was added and the layers were separated. The aqueous layer was extracted with DCM (3 x 20 mL) and the combined organic layers were dried over $MgSO_4$, filtered and the solvents removed under reduced pressure, obtaining (*Z*)-hex-3-en-1-amine (**S14**, 148 mg, 1.49 mmol, 50% yield) as a yellow oil. The crude was used in the next step without any further purification.

Analytical data is consistent with literature values.[13]

^{1}H NMR (400 MHz, $CDCl_3$) δ/ppm: 5.56 – 5.44 (m, 1H, C=C*H*), 5.37 – 5.26 (m, 1H, C=C*H*), 2.71 (t, *J* = 6.7 Hz, 2H, CH_2), 2.23 – 2.14 (m, 2H, CH_2), 2.12 – 2.01 (m, 2H, CH_2), 1.26 (bs, 2H, NH_2), 0.96 (t, *J* = 7.5 Hz, 3H, CH_3).

^{13}C {^{1}H} NMR (101 MHz, $CDCl_3$) δ/ppm: 134.0, 126.3, 42.2, 31.6, 20.8, 14.5.

(*Z*)-*N*-(hex-3-en-1-yl)-4-methylbenzenesulfonamide (S7o)

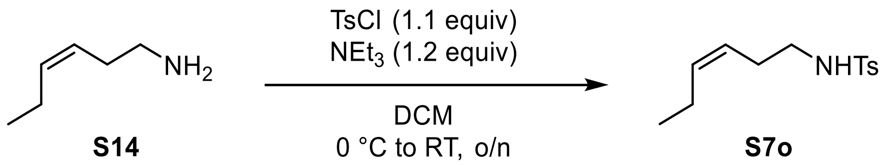

A 25 mL two-necked round-bottomed flask equipped with a magnetic stirring bar was evacuated and backfilled with N_2 three times. (*Z*)-Hex-3-en-1-amine (**S14**, 99.2 mg, 1.00 mmol, 1.0 equiv) was added, followed by DCM (5.0 mL) and triethylamine (0.12 g, 0.20 mL, 1.2 mmol, 1.2 equiv) were added and the solution was cooled with an ice bath. A solution of tosyl chloride (210 mg, 1.10 mmol, 1.1 equiv) in DCM (2.5 mL) was added dropwise to the reaction mixture and the latter was allowed to warm to room temperature and it was stirred overnight. The reaction was then quenched with $NaHCO_3$ (5 mL) and the layers were separated. The aqueous layer was extracted with DCM (3 x 10 mL) and the combined organic

[13] Asensio, G.; Mello, R.; Boix-Bernardini, C.; Gonzalez-Nunez, M. E.; Castellano, G. Epoxidation of Primary and Secondary Alkenylammonium Salts with Dimethyldioxirane, Methyl(Trifluoromethyl)Dioxirane, and m-Chloroperbenzoic Acid. A General Synthetic Route to Epoxyalkylamines. *J. Org. Chem.* **1995**, *60* (12), 3692–3699.

layers were washed with brine (10 mL), dried over $MgSO_4$, filtered and the solvents removed under reduced pressure.

The crude was purified by flash chromatography (10% EtOAc in Pentane) obtaining (*Z*)-*N*-(hex-3-en-1-yl)-4-methylbenzenesulfonamide (**S7o**, 124 mg, 65.3 mmol, 49% yield) as a colorless oil.

Analytical data is consistent with literature values.[14]

Rf (1:9 EtOAc:Pentane) = 0.3

^{1}H NMR (400 MHz, $CDCl_3$) δ/ppm: 7.80 – 7.68 (m, 2H, Ar*H*), 7.31 (d, *J* = 8.1 Hz, 2H, Ar*H*), 5.62 – 5.41 (m, 1H, C=C*H*), 5.22 – 5.06 (m, 1H, C=C*H*), 4.38 (t, *J* = 6.1 Hz, 1H, C*H*2), 2.97 (q, *J* = 6.6 Hz, 2H, C*H*2), 2.43 (s, 3H, C*H*3), 2.20 (qd, *J* = 7.0, 1.5 Hz, 2H, C*H*2), 1.97 (pd, *J* = 7.5, 1.6 Hz, 2H, C*H*2), 1.00 – 0.86 (m, 3H, C*H*3).

^{13}C {^{1}H} NMR (101 MHz, $CDCl_3$) δ/ppm: 143.5, 137.1, 135.7, 129.8, 127.3, 124.1, 42.9, 27.4, 21.7, 20.7, 14.3.

N-(2-(cyclohex-1-en-1-yl)ethyl)-4-methylbenzenesulfonamide (S7p)

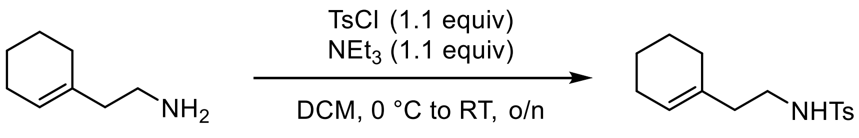

A 100 mL two-necked round-bottomed flask equipped with a magnetic stirring bar was evacuated and backfilled with N_2 three times. 2-(Cyclohex-1-en-1-yl)ethan-1-amine (**S15**, 1.25 g, 1.40 mL, 10.0 mmol, 1.0 equiv) was added, followed by DCM (20 mL). The solution was cooled with an ice bath. Triethylamine (1.11 g, 1.50 mL, 11.0 mmol, 1.1 equiv) was added, followed by a solution of tosylchloride (2.10 g, 11.0 mmol, 1.1 equiv) in DCM (10 mL). The reaction mixture was allowed to warm to room temperature and stirred overnight. The reaction was then quenched with sat. aq. $NaHCO_3$ (20 mL) and the layers were separated. The aqueous layer was extracted with DCM (3 x 20 mL), the combined organic layers were washed with brine (20 mL), dried over $MgSO_4$, filtered and the solvents were removed under reduced pressure. The crude was purified by flash chromatography (10% EtOAc in Pentane) obtaining *N*-(2-(cyclohex-1-en-1-yl)ethyl)-4-methylbenzenesulfonamide (**S7p**, 2.24 g, 8.02 mmol, 80% yield) as a pale yellow solid.

Analytical data is consistent with literature values.[15]

Rf (15% EtOAc in Pentane) = 0.3

^{1}H NMR (400 MHz, $CDCl_3$) δ/ppm: 7.80 – 7.70 (m, 2H, Ar*H*), 7.35 – 7.28 (m, 2H, Ar*H*), 5.41 – 5.36 (m, 1H, C=C*H*), 4.30 (t, *J* = 5.8 Hz, 1H, C*H*2), 3.00 (td, *J* = 6.5, 5.6 Hz, 2H, C*H*2), 2.43 (s, 3H, C*H*3), 2.05 (td, *J* = 6.7, 1.5 Hz, 2H, C*H*2), 2.01 – 1.90 (m, 2H, C*H*2), 1.74 – 1.65 (m, 2H, C*H*2), 1.61 – 1.45 (m, 4H, C*H*2).

---

[14] Jones, A. D.; Knight, D. W.; Hibbs, D. E. A Stereochemically Flexible Approach to Pyrrolidines Based on 5-Endo-Trig Iodocyclisations of Homoallylic Sulfonamides. *J Chem Soc Perkin Trans 1* **2001**, No. 10, 1182–1203.

[15] Nguyen, T. M.; Nicewicz, D. A. Anti-Markovnikov Hydroamination of Alkenes Catalyzed by an Organic Photoredox System. *J. Am. Chem. Soc.* **2013**, *135* (26), 9588–9591.

$^{13}C\ \{^1H\}$ NMR (101 MHz, $CDCl_3$) δ/ppm: 143.5, 137.0, 133.6, 129.8, 127.3, 125.0, 40.5, 37.5, 27.6, 25.3, 22.8, 22.3, 21.7.

_N_-(But-3-en-1-yl)-4-(5-(p-tolyl)-3-(trifluoromethyl)-1H-pyrazol-1-yl)benzenesulfonamide (S7q)

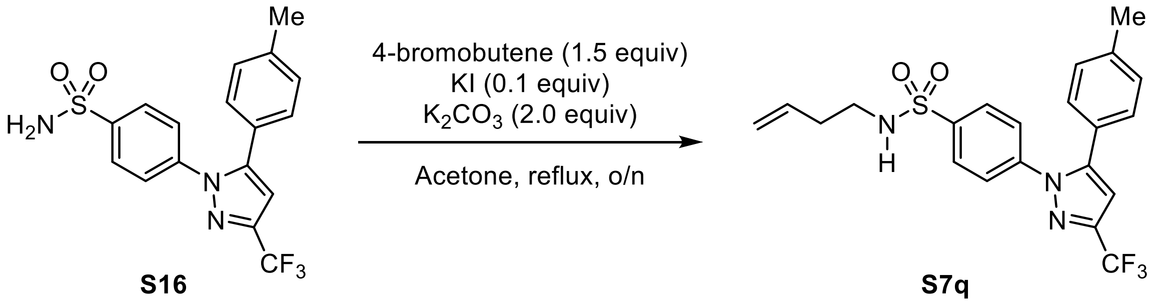

S16 **S7q**

A 2-5 mL Biotage microwave vial equipped with a magnetic stirring bar was charged with Celecoxib (**S16**, 458 mg, 1.20 mmol, 1.0 equiv), KI (20 mg, 0.12 mmol, 0.1 equiv), potassium carbonate (332 mg, 2.40 mmol, 2.0 equiv) and acetone (3.0 mL). The vial as sealed and 4-bromobutene (243 mg, 0.180 mL, 1.5 equiv) was added and the reaction mixture was refluxed overnight. The suspension was allowed to cool to RT, the solids were filtered off and the solvents were removed under reduced pressure. The crude was purified by flash chromatography (7 to 30% EtOAc in Pentane) obtaining _N_-(but-3-en-1-yl)-4-(5-(p-tolyl)-3-(trifluoromethyl)-1H-pyrazol-1-yl)benzenesulfonamide (**S7q**, 391 mg, 0.900 mmol, 75% yield) as a pale yellow amorphous solid.

Rf (15% EtOAc in Pentane) = 0.3

^1H NMR (400 MHz, $CDCl_3$) δ/ppm: 7.88 – 7.80 (m, 2H, Ar*H*), 7.52 – 7.43 (m, 2H, Ar*H*), 7.17 (d, *J* = 7.8 Hz, 2H, Ar*H*), 7.13 – 7.06 (m, 2H, Ar*H*), 6.74 (s, 1H, Ar*H*), 5.61 (ddt, *J* = 17.1, 10.2, 6.9 Hz, 1H, C*H*=CH$_2$), 5.12 – 5.07 (m, 1H, CH=C*H*$_2$), 5.07 – 5.01 (m, 1H, CH=C*H*$_2$), 4.45 (t, *J* = 6.1 Hz, 1H, N*H*), 3.04 (q, *J* = 6.4 Hz, 2H, C*H*$_2$), 2.38 (s, 3H, C*H*$_3$), 2.27 – 2.14 (m, 2H, C*H*$_2$).

$^{13}C\ \{^1H\}$ NMR (101 MHz, $CDCl_3$) δ/ppm: 145.4, 144.1 (q, *J* = 38.7 Hz), 142.7, 140.0, 139.6, 134.0, 129.9, 128.9, 128.3, 125.8, 125.8, 121.2 (q, *J* = 269.2 Hz), 118.7, 106.4 (d, *J* = 2.0 Hz), 42.2, 33.7, 21.5.

^{19}F NMR (376 MHz, $CDCl_3$) δ/ppm: – 62.5

HRMS (ESI/QTOF) m/z: $[M + H]^+$ Calcd for $C_{21}H_{21}F_3N_3O_2S^+$ 436.1301; Found 436.1310.

6.1.4. Synthesis of Homoallylic Cesium Oxalates and Oxamates

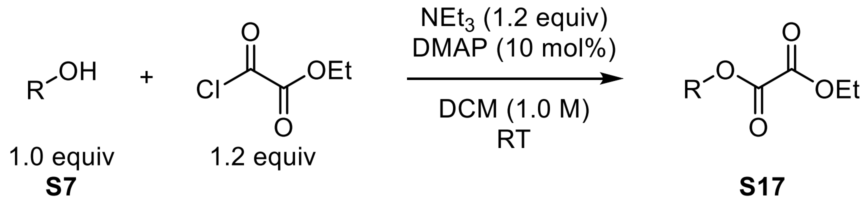

General Procedure 2

Following a modified reported procedure,[1] a two-necked round bottom flask equipped with a magnetic stirring bar was charged with DMAP (10 mol%). The flask was evacuated and backfilled with N_2 three times. Dichloromethane (1.0 M), triethylamine (1.20 equiv) and the alcohol (1.0 equiv) were then added, followed by the dropwise addition of ethyl 2-chloro-2-oxoacetate (1.20 equiv). The mixture was then stirred at room temperature until TLC showed full conversion. The reaction was quenched with sat. aq. NH_4Cl (1 volume). The layers were then separated and the aqueous layer was extracted with DCM (3 x 1 volume). The combined organic layer was then washed with brine (1 volume). The organic layer was then dried over $MgSO_4$, filtered and the solvents were removed under reduced pressure. The crude compound was then purified by flash chromatography.

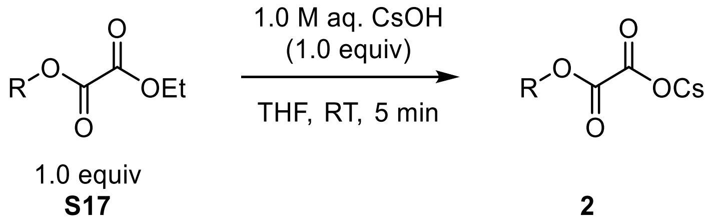

General Procedure 3

The reaction was performed in an open flask.

Following a modified reported procedure,[1] a round-bottomed flask equipped with a magnetic stirring bar was charged with the ethyl oxalate (1.0 equiv). THF (1.0 M) was added, followed by CsOH (1.0 M in H_2O, 1.0 equiv). The reaction mixture was vigorously stirred for 5 min and then the solvents were removed under reduced pressure (bath = 60 °C), azeotroping the water with toluene and then dried at high vacuum overnight obtaining the cesium oxalates as hygroscopic solids. The obtained compounds were not further purified.

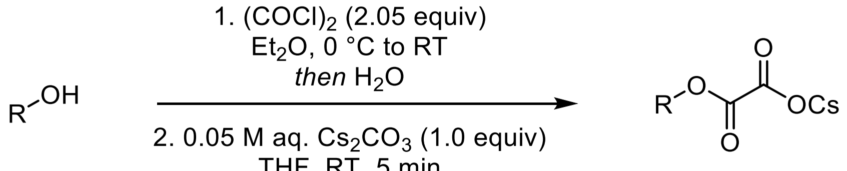

S7 **2**

General Procedure 4

Following a modified reported procedure,[16] a solution of the alcohol (1.0 equiv) in diethyl ether (0.5 M) was added dropwise to a stirring solution of oxalyl chloride in diethyl ether (0.4 M) at 0 °C. The suspension was allowed to warm to room temperature and stirred until TLC showed full conversion (approximately 2 h). The reaction mixture was then cooled to 0 °C, quenched with water and stirred at open air for 15 min. The two layers were separated and the aqueous layer was extracted with diethyl ether. The combined organic layer was dried over $MgSO_4$, filtered and the solvents were removed under reduced pressure (water bath = 35 °C).

The crude was then dissolved in THF (1.0 M) and aq. Cs_2CO_3 (0.5 M, 0.5 equiv) was added dropwise. The reaction was stirred for 5 min and then the solvents were removed under reduced pressure (water bath = 60 °C) azeotroping the water with toluene, and then dried at high vacuum overnight obtaining the cesium oxalates as hygroscopic solids. The compounds were used without any purification.

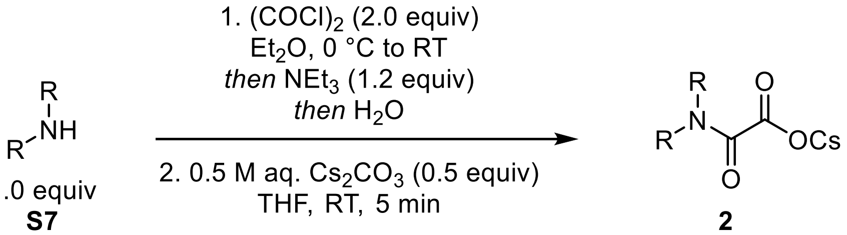

1.0 equiv
S7 **2**

General Procedure 5

Following a modified reported procedure,[3] a round-bottomed flask equipped with a magnetic stirring bar was charged with the sulfonamide (1.0 equiv). The flask was evacuated and backfilled with N_2 three times, Et_2O (0.05 M) was added and the solution was cooled with an ice bucket. Oxalyl chloride (2.0 equiv) was added dropwise, the reaction mixture was allowed to warm to room temperature and stirred for 30 min. Triethylamine (1.2 equiv) was added, a white precipitate was formed, and the suspension was stirred for 30 min. The reaction mixture was then cooled with an ice bucket and quenched with H_2O (1 volume). The two layers were separated and the aqueous layer was extracted with Et_2O. The combined organic layers were washed with brine, dried over $MgSO_4$ and the solvent removed under reduced pressure.

The crude was then dissolved in THF (1.0 M) and aq. Cs_2CO_3 (0.5 M, 0.5 equiv) was added dropwise. The reaction was stirred for 5 min and then the solvents were removed under reduced pressure (water bath = 60 °C) azeotroping the water with toluene, and then dried at high vacuum overnight obtaining the cesium oxamates as hygroscopic solids. The compounds were used without any purification.

[16] Su, J. Y.; Grünenfelder, D. C.; Takeuchi, K.; Reisman, S. E. Radical Deoxychlorination of Cesium Oxalates for the Synthesis of Alkyl Chlorides. *Org. Lett.* **2018**, *20* (16), 4912–4916.

1-Allylcyclohexyl ethyl oxalate (S17a)

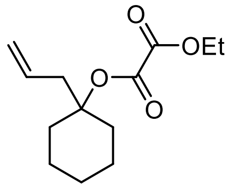

S17a was prepared according to *General Procedure 2* from 1-allylcyclohexenol (**S7a**, 3.51 g, 25.0 mmol, 1.0 equiv). The crude was purified by flash chromatography (SiO_2, 4% Et2O in Pentane, Rf = 0.35) obtaining 1-allylcyclohexyl ethyl oxalate (**S17a**, 5.18 g, 21.6 mmol, 86% yield) as a colorless oil.

Rf (4% Et2O in Pentane) = 0.35.

^{1}H NMR (400 MHz, $CDCl_3$) δ/ppm: 5.75 (ddt, *J* = 15.9, 11.1, 7.4 Hz, 1H, C*H*=CH$_2$), 5.13 – 5.09 (m, 1H, CH=C*H*$_2$), 5.09 – 5.05 (m, 1H, CH=C*H*$_2$), 4.31 (q, *J* = 7.1 Hz, 2H, C*H*$_2$), 2.70 (dt, *J* = 7.4, 1.2 Hz, 2H, C*H*$_2$), 2.42 – 2.18 (m, 2H, C*H*$_2$), 1.70 – 1.40 (m, 6H, C*H*$_2$), 1.36 (t, *J* = 7.1 Hz, 3H, C*H*$_2$), 1.26 (m, 2H, C*H*$_2$).

^{13}C {^{1}H} NMR (101 MHz, $CDCl_3$) δ/ppm: 158.7, 157.1, 132.2, 119.0, 88.2, 62.8, 41.9, 34.2, 25.4, 21.8, 14.1.

HRMS (ESI/QTOF) m/z: [M + Na]$^+$ Calcd for $C_{13}H_{20}NaO_4^+$ 263.1254; Found 263.1253.

Cesium 2-((1-allylcyclohexyl)oxy)-2-oxoacetate (2a)

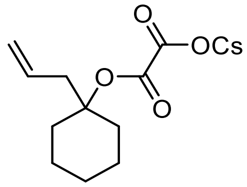

2a was prepared according to *General Procedure 3* from 1-allylcyclohexyl ethyl oxalate (**S17a**, 3.60 g, 15.0 mmol, 1.0 equiv), obtaining cesium 2-((1-allylcyclohexyl)oxy)-2-oxoacetate (**2a**, 5.14 g, 14.9 mmol, 100% yield) as a hygroscopic solid.

Analytical data is consistent with literature values.[3]

^{1}H NMR (400 MHz, DMSO-d_6) δ/ppm: 5.74 (ddt, *J* = 15.8, 11.4, 7.3 Hz, 1H, C*H*=CH$_2$), 5.08 – 4.98 (m, 2H, CH=C*H*$_2$), 2.59 (dt, *J* = 7.2, 1.3 Hz, 2H, C*H*$_2$), 2.04 (dt, *J* = 13.0, 3.9 Hz, 2H, C*H*$_2$), 1.61 – 1.11 (m, 8H, C*H*$_2$).

^{13}C {^{1}H} NMR (101 MHz, DMSO-d_6) δ/ppm: 167.6, 163.3, 133.6, 117.7, 80.8, 41.6, 34.0, 25.0, 21.1.

4-Allyltetrahydro-2H-pyran-4-yl ethyl oxalate (S17b)

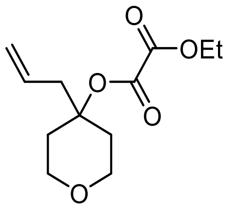

S17b was prepared according to *General Procedure 2* from 4-allyltetrahydro-2H-pyran-4-ol (**S7b**, 1.28 g, 9.00 mmol, 1.00 equiv), ethyl chlorooxoacetate (1.48 g, 1.20 mL, 10.8 mmol, 1.2 equiv), triethylamine (1.09 g, 1.50 mL, 10.8 mmol, 1.2 equiv) and DMAP (110 mg, 0.900 mmol, 10 mol%) in THF (90 mL). The crude was purified by flash chromatography (SiO_2, 10% EtOAc in Pentane, Rf = 0.3) obtaining 4-allyltetrahydro-2H-pyran-4-yl ethyl oxalate (**S17b**, 1.28 g, 5.29 mmol, 59% yield) as a colorless oil.

Rf (10% EtOAc in Pentane) = 0.3.

^{1}H NMR (400 MHz, $CDCl_3$) δ/ppm: 5.73 (ddt, *J* = 16.7, 10.3, 7.3 Hz, 1H, CH=CH_2), 5.20 – 5.04 (m, 2H, CH=CH_2), 4.32 (q, *J* = 7.1 Hz, 2H, CH_2), 3.78 (ddd, *J* = 11.9, 4.8, 2.8 Hz, 2H, CH_2), 3.66 (td, *J* = 11.5, 2.3 Hz, 2H), 2.75 (dt, *J* = 7.4, 1.2 Hz, 2H, CH_2), 2.23 (dq, *J* = 14.6, 2.7 Hz, 2H, CH_2), 1.77 (ddd, *J* = 14.2, 11.3, 5.0 Hz, 2H, CH_2), 1.36 (t, *J* = 7.1 Hz, 3H, CH_2).

^{13}C {^{1}H} NMR (101 MHz, $CDCl_3$) δ/ppm: 158.3, 157.0, 131.0, 119.9, 84.8, 63.5, 63.1, 41.7, 34.5, 14.0.

HRMS (ESI/QTOF) m/z: $[M + Na]^+$ Calcd for $C_{12}H_{18}NaO_5^+$ 265.1046; Found 265.1053.

Cesium 2-((4-allyltetrahydro-2H-pyran-4-yl)oxy)-2-oxoacetate (2b)

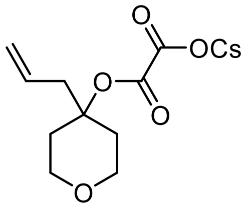

2b was prepared according to *General Procedure 3* from 4-allyltetrahydro-2H-pyran-4-yl ethyl oxalate (969 mg, 4.00 mmol, 1.0 equiv). Obtaining cesium 2-((4-allyltetrahydro-2H-pyran-4-yl)oxy)-2-oxoacetate (**2b**, 1.38 g, 3.99 mmol, 100% yield) as a hygroscopic solid.

Analytical data is consistent with literature values.[3]

^{1}H NMR (400 MHz, DMSO-d_6) δ/ppm: 5.84 – 5.59 (m, 1H, C*H*=CH_2), 5.25 – 5.08 (m, 1H, CH=CH_2), 5.08 – 4.98 (m, 1H, CH=CH_2), 3.62 (ddd, *J* = 11.5, 4.6, 3.2 Hz, 2H, CH_2), 3.53 (td, *J* = 11.1, 2.4 Hz, 2H, CH_2), 2.65 (dt, *J* = 7.4, 1.2 Hz, 2H, CH_2), 2.00 (dq, *J* = 14.2, 2.7 Hz, 2H, CH_2), 1.58 (ddd, *J* = 14.1, 10.8, 4.8 Hz, 2H, CH_2).

^{13}C {^{1}H} NMR (101 MHz, DMSO-d_6) δ/ppm: 167.4, 163.0, 132.8, 118.4, 78.2, 62.6, 41.3, 34.4.

4-Allyl-1-(tert-butoxycarbonyl)piperidin-4-yl ethyl oxalate (S17c)

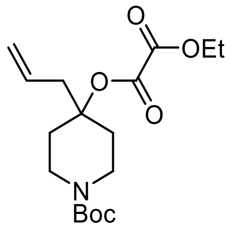

S17c was prepared according to *General Procedure 2* from tert-butyl 4-allyl-4-hydroxypiperidine-1-carboxylate (**S7c**, 7.24 mg, 3.00 mmol, 1.00 equiv). The crude was purified by flash chromatography (SiO_2, 10% EtOAc in Pentane, Rf = 0.45) obtaining 4-allyl-1-(tert-butoxycarbonyl)piperidin-4-yl ethyl oxalate (**S17c**, 605 mg, 1.77 mmol, 59% yield) as a colorless oil.

Rf (5% EtOAc in Pentane) = 0.5.

^{1}H NMR (400 MHz, $CDCl_3$) δ/ppm: 5.72 (ddt, *J* = 16.9, 10.3, 7.4 Hz, 1H, C*H*=CH$_2$), 5.21 – 5.01 (m, 2H, CH=C*H*$_2$), 4.32 (q, *J* = 7.1 Hz, 2H, C*H*$_2$), 3.89 (s, 2H, C*H*$_2$), 3.03 (t, *J* = 12.7 Hz, 2H, C*H*$_2$), 2.74 (dt, *J* = 7.5, 1.2 Hz, 2H, C*H*$_2$), 2.29 (dq, *J* = 14.6, 2.8 Hz, 2H, C*H*$_2$), 1.60 (ddd, *J* = 14.1, 11.9, 4.8 Hz, 2H, C*H*$_2$), 1.45 (s, 9H, C*H*$_3$), 1.36 (t, *J* = 7.1 Hz, 3H, C*H*$_3$).

^{13}C {^{1}H} NMR (101 MHz, $CDCl_3$) δ/ppm: 158.2, 157.0, 154.8, 131.1, 119.9, 85.5, 79.9, 63.1, 41.6, 39.5, 33.7, 28.5, 14.1.

HRMS (ESI/QTOF) m/z: $[M + Na]^+$ Calcd for $C_{17}H_{27}NNaO_6^+$ 364.1731; Found 364.1737.

Cesium 2-((4-allyl-1-(tert-butoxycarbonyl)piperidin-4-yl)oxy)-2-oxoacetate (2c)

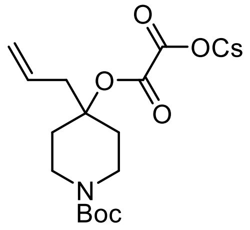

2c was prepared according to *General Procedure 3* from 4-allyl-1-(tert-butoxycarbonyl)piperidin-4-yl ethyl oxalate (**S17c**, 512 mg, 1.50 mmol, 1.0 equiv). The resulting solid was washed with pentane and Et_2O, obtaining cesium 2-((4-allyl-1-(tert-butoxycarbonyl)piperidin-4-yl)oxy)-2-oxoacetate (**2c**, 300 mg, 0.670 mmol, 45% yield) as a hygroscopic solid.

Analytical data is consistent with literature values. [3]

^{1}H NMR (400 MHz, DMSO-*d*$_6$) δ/ppm: 5.82 – 5.62 (m, 1H, C*H*=CH$_2$), 5.15 – 4.91 (m, 2H, CH=C*H*$_2$), 3.67 (d, *J* = 13.1 Hz, 2H, C*H*$_2$), 3.07 – 2.85 (m, 2H, C*H*$_2$), 2.63 (d, *J* = 7.3 Hz, 2H, C*H*$_2$), 2.05 (d, *J* = 13.6 Hz, 2H, C*H*$_2$), 1.57 – 1.41 (m, 2H C*H*$_2$), 1.39 (s, 9H, C*H*$_3$).

^{13}C {^{1}H} NMR (101 MHz, DMSO-*d*$_6$) δ/ppm: 167.4, 162.9, 154.0, 132.8, 118.5, 78.7, 78.6, 41.1, 38.6, 33.3, 28.1.

1-Allylcyclopentyl ethyl oxalate (S17d)

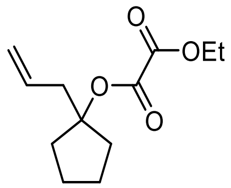

S17d was prepared according to *General Procedure 2* from 1-allylcyclopentenol (**S7d**, 350 mg, 2.77 mmol, 1.00 equiv). The crude was purified by flash chromatography (SiO_2, 5% Et_2O in Pentane, Rf = 0.3) obtaining 1-allylcyclopentyl ethyl oxalate (**S17d**, 521 mg, 2.30 mmol, 83% yield) as a colorless oil.

Rf (5% Et2O in Pentane) = 0.3.

^{1}H NMR (400 MHz, $CDCl_3$) δ/ppm: 5.87 – 5.65 (m, 1H, C*H*=CH_2), 5.16 – 5.09 (m, 1H, CH=C*H*$_2$), 5.08 (d, *J* = 1.1 Hz, 1H, CH=C*H*$_2$), 4.31 (q, *J* = 7.1 Hz, 2H, C*H*$_2$), 2.77 (dt, *J* = 7.2, 1.2 Hz, 2H, C*H*$_2$), 2.28 – 2.08 (m, 2H, C*H*$_2$), 1.93 – 1.71 (m, 4H, C*H*$_2$), 1.69 – 1.58 (m, 2H, C*H*$_2$), 1.36 (t, *J* = 7.1 Hz, 3H, C*H*$_3$).

^{13}C {^{1}H} NMR (101 MHz, $CDCl_3$) δ/ppm: 158.7, 157.4, 133.0, 118.8, 96.1, 62.9, 41.1, 37.1, 24.0, 14.1.

HRMS (ESI/QTOF) m/z: $[M + Na]^+$ Calcd for $C_{12}H_{18}NaO_4^+$ 249.1097; Found 249.1092.

Cesium 2-((1-allylcyclopentyl)oxy)-2-oxoacetate (2d)

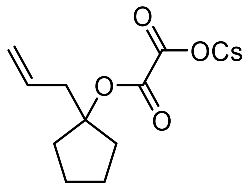

2d was prepared according to *General Procedure 3* from 1-allylcyclopentyl ethyl oxalate (**S7d**, 453 mg, 2.00 mmol, 1.0 equiv), obtaining cesium 2-((1-allylcyclopentyl)oxy)-2-oxoacetate (**2d**, 631 mg, 1.91 mmol, 96% yield) as a hygroscopic solid.

Analytical data is consistent with literature values. [3]

^{1}H NMR (400 MHz, DMSO-*d*$_6$) δ/ppm: 5.76 (ddt, *J* = 17.4, 10.3, 7.2 Hz, 1H, C*H*=CH_2), 5.10 – 5.04 (m, 1H, CH=C*H*$_2$), 5.04 – 5.01 (m, 1H, CH=C*H*$_2$), 2.64 (dt, *J* = 7.2, 1.3 Hz, 2H, C*H*$_2$), 2.08 – 1.84 (m, 2H, C*H*$_2$), 1.75 – 1.43 (m, 6H, C*H*$_2$).

^{13}C {^{1}H} NMR (101 MHz, DMSO-*d*$_6$) δ/ppm: 167.5, 163.3, 134.2, 117.6, 89.4, 40.9, 36.7, 23.5.

1-Allylcyclododecyl ethyl oxalate (S17e)

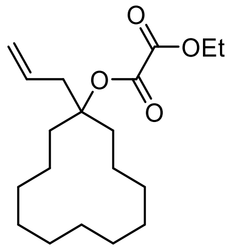

S17e was prepared according to *General Procedure 2* from 1-allylcyclododecanol (**S7e**, 673 mg, 3.00 mmol, 1.00 equiv). The crude was purified by flash chromatography (SiO_2, 2% to 5% Et_2O in Pentane, Rf (2%) = 0.18) obtaining 1-allylcyclododecyl ethyl oxalate (**S17e**, 175 mg, 0.539 mmol, 18% yield) as a white solid.

Rf (2% Et2Oin Pentane) = 0.18.

^{1}H NMR (400 MHz, $CDCl_3$) δ/ppm: 5.84 – 5.69 (m, 1H, C*H*=CH$_2$), 5.11 (d, *J* = 1.4 Hz, 1H, CH=C*H*$_2$), 5.10 – 5.04 (m, 1H, CH=C*H*$_2$), 4.30 (q, *J* = 7.1 Hz, 2H, C*H*$_2$), 2.70 (d, *J* = 7.2 Hz, 2H, C*H*$_2$), 2.02 (ddd, *J* = 14.1, 11.5, 4.5 Hz, 2H, C*H*$_2$), 1.68 (ddd, *J* = 14.1, 11.6, 4.6 Hz, 2H, C*H*$_2$), 1.53 – 1.16 (m, 21H, C*H*$_2$).

^{13}C {^{1}H} NMR (101 MHz, $CDCl_3$) δ/ppm: 158.7, 157.0, 132.2, 118.8, 92.0, 62.9, 39.5, 31.1, 26.2, 22.4, 22.0, 19.1, 14.1.

HRMS (ESI/QTOF) m/z: $[M + Na]^+$ Calcd for $C_{19}H_{32}NaO_4^+$ 347.2193; Found 347.2204.

Cesium 2-((1-allylcyclododecyl)oxy)-2-oxoacetate (2e)

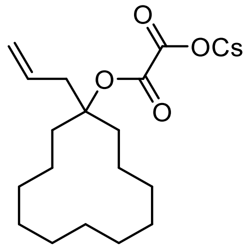

2e was prepared according to *General Procedure 3* from 1-allylcyclododecyl ethyl oxalate (**S17e**, 130 mg, 0.400 mmol, 1.0 equiv). Obtaining cesium 2-((1-allylcyclododecyl)oxy)-2-oxoacetate (**2e**, 169 mg, 0.400 mmol, 100% yield) as a hygroscopic solid.

^{1}H NMR (400 MHz, DMSO-*d*$_6$) δ/ppm: 5.76 (ddt, *J* = 16.4, 11.5, 7.1 Hz, 1H, C*H*=CH$_2$), 5.11 – 5.05 (m, 1H, CH=C*H*$_2$), 5.05 – 5.01 (m, 1H, CH=C*H*$_2$), 2.59 (d, *J* = 7.2 Hz, 2H, C*H*$_2$), 1.88 – 1.72 (m, 2H, C*H*$_2$), 1.58 – 1.43 (m, 2H, C*H*$_2$), 1.41 – 1.12 (m, 18H, C*H*$_2$).

^{13}C {^{1}H} NMR (101 MHz, DMSO-*d*$_6$) δ/ppm: 167.5, 163.3, 133.6, 117.4, 84.6, 39.2, 30.9, 25.8, 25.7, 21.8, 21.5, 18.2.

HRMS (ESI/QTOF) m/z: $[M]^-$ Calcd for $C_{17}H_{27}O_4^-$ 295.1915; Found 295.1903.

2-Allyladamantan-2-yl ethyl oxalate (S17f)

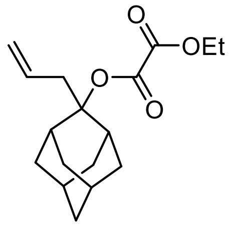

S17f was prepared according to *General Procedure 2* from 2-allyladamantan-2-ol (**S7f**, 573 mg, 3.00 mmol, 1.00 equiv). The crude was purified by flash chromatography (SiO_2, 2% Et_2O in Pentane, Rf = 0.4) obtaining 2-allyladamantan-2-yl ethyl oxalate (**S17f**, 224 mg, 0.766 mmol, 26% yield) as a white solid.

Rf (2% Et_2O in Pentane) = 0.4.

^{1}H NMR (400 MHz, $CDCl_3$) δ/ppm: 5.85 – 5.60 (m, 1H, C*H*=CH$_2$), 5.09 (dtd, *J* = 13.2, 2.4, 1.1 Hz, 2H, CH=C*H*$_2$), 4.32 (q, *J* = 7.1 Hz, 2H, C*H*$_2$), 2.97 (dt, *J* = 7.4, 1.3 Hz, 2H, C*H*$_2$), 2.45 (t, *J* = 3.1 Hz, 2H, Ad*H*), 2.13 – 2.00 (m, 2H, Ad*H*), 1.97 – 1.83 (m, 4H, Ad*H*), 1.81 – 1.68 (m, 4H, Ad*H*), 1.65 – 1.58 (m, 2H, Ad*H*), 1.36 (t, *J* = 7.1 Hz, 3H, C*H*$_3$).

^{13}C {^{1}H} NMR (101 MHz, $CDCl_3$) δ/ppm: 158.8, 156.8, 132.2, 118.6, 93.1, 62.8, 38.2, 36.9, 34.3, 34.0, 33.1, 27.1, 26.9, 14.1.

HRMS (ESI/QTOF) m/z: [M + Na]$^+$ Calcd for $C_{17}H_{24}NaO_4^+$ 315.1567; Found 315.1570.

Cesium 2-((-2-allyladamantan-2-yl)oxy)-2-oxoacetate (2f)

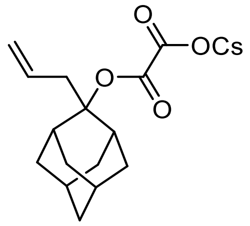

2f was prepared according to *General Procedure 3* from 2-allyladamantan-2-yl ethyl oxalate (**S17f**, 146 mg, 0.500 mmol, 1.0 equiv), obtaining cesium 2-((-2-allyladamantan-2-yl)oxy)-2-oxoacetate (**2f**, 195 mg, 0.490 mmol, 98% yield) as a hygroscopic solid.

Analytical data is consistent with literature values. [3]

^{1}H NMR (400 MHz, DMSO-d_6) δ/ppm: 5.90 – 5.62 (m, 1H, C*H*=CH$_2$), 5.18 – 4.97 (m, 2H, CH=C*H*$_2$), 2.86 (dt, *J* = 7.2, 1.3 Hz, 2H, C*H*$_2$), 2.22 (d, *J* = 3.7 Hz, 2H, Ad*H*), 2.05 (d, *J* = 12.4 Hz, 2H, Ad*H*), 1.85 (d, *J* = 13.1 Hz, 2H, Ad*H*), 1.75 (dq, *J* = 7.5, 3.0 Hz, 2H, Ad*H*), 1.70 – 1.57 (m, 4H, Ad*H*), 1.45 (dd, *J* = 11.9, 3.1 Hz, 2H, Ad*H*).

^{13}C {^{1}H} NMR (101 MHz, DMSO-d_6) δ/ppm: 167.4, 163.3, 133.6, 117.4, 85.2, 37.8, 36.5, 33.5, 33.2, 32.1, 26.6, 26.4.

(3S,8R,9S,10R,13S,14S,17R)-17-allyl-3-(tert-butoxy)-10,13-dimethyl-2,3,4,7,8,9,10,11,12,13,14,15,16,17-tetradecahydro-1H-cyclopenta[a]phenanthren-17-yl ethyl oxalate (S17g)

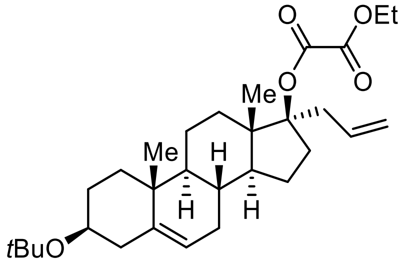

S17g was prepared according to *General Procedure 2* from 3β-*tert*-butoxy-17α-allyl-dehydroepiandrostane-17β-ol (**S7g**, 290 mg, 0.75 mmol, 1.0 equiv). The crude was purified by flash chromatography (SiO_2, 5% EtOAc in Pentane) obtaining (3S,8R,9S,10R,13S,14S,17R)-17-allyl-3-(tert-butoxy)-10,13-dimethyl-2,3,4,7,8,9,10,11,12,13,14,15,16,17-tetradecahydro-1*H*-cyclopenta[α]phenanthren-17-yl ethyl oxalate (**S17g**, 166 mg, 0.340 mmol, 45% yield) as colorless oil.

Rf (5% EtOAc in Pentane) = 0.4.

^{1}H NMR (400 MHz, $CDCl_3$) δ/ppm: 5.79 (dddd, *J* = 17.0, 10.5, 8.1, 6.8 Hz, 1H, C*H*=CH_2), 5.30 (dt, *J* = 5.1, 1.8 Hz, 1H, C=C*H*), 5.14 – 4.99 (m, 2H, CH=CH_2), 4.30 (q, *J* = 7.1 Hz, 2H, $COOCH_2$), 3.30 (tt, *J* = 11.2, 4.7 Hz, 1H, *t*BuOC*H*), 3.13 (dd, *J* = 14.8, 6.6 Hz, 1H), 2.41 (ddt, *J* = 14.8, 7.5, 1.3 Hz, 1H), 2.33 – 2.22 (m, 1H), 2.19 – 2.09 (m, 3H), 2.09 – 1.96 (m, 2H), 1.83 (dt, *J* = 13.2, 3.5 Hz, 1H), 1.76 – 1.47 (m, 6H), 1.35 (t, *J* = 7.1 Hz, 3H, CH_2CH_3), 1.26 (td, *J* = 6.2, 2.2 Hz, 1H), 1.19 (s, 9H, $(CH_3)_3$), 1.07 (td, *J* = 13.4, 3.8 Hz, 1H), 1.00 (s, 3H, CH_3), 0.97 – 0.89 (m, 1H), 0.89 (s, 3H, CH_3).

^{13}C {^{1}H} NMR (101 MHz, $CDCl_3$) δ/ppm: 158.8, 157.2, 142.3, 133.2, 120.5, 118.8, 97.6, 73.5, 71.5, 62.8, 51.6, 50.1, 47.5, 42.1, 38.2, 37.9, 36.8, 35.2, 33.3, 32.8, 31.9, 31.4, 28.6, 23.9, 20.9, 19.5, 14.5, 14.1.

HRMS (ESI/QTOF) m/z: $[M + Na]^+$ Calcd for $C_{30}H_{46}NaO_5^+$ 509.3237; Found 509.3248.

Cesium 2-(((3S,8R,9S,10R,13S,14S,17R)-17-allyl-3-(tert-butoxy)-10,13-dimethyl-2,3,4,7,8,9,10,11,12,13,14,15,16,17-tetradecahydro-1H-cyclopenta[a]phenanthren-17-yl)oxy)-2-oxoacetate (2g)

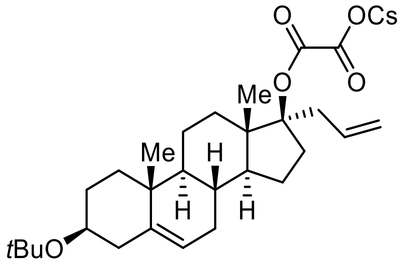

2g was prepared according to *General Procedure 3* from (3S,8R,9S,10R,13S,14S,17R)-17-allyl-3-(tert-butoxy)-10,13-dimethyl-2,3,4,7,8,9,10,11,12,13,14,15,16,17-tetradecahydro-1*H*-cyclopenta[α] phenanthren-17-yl ethyl oxalate (**S17g**, 219 mg, 0.450 mmol, 1.0 equiv). Obtaining Cesium 2-(((3S,8R,9S,10R,13S,14S,17R)-17-allyl-3-(tert-butoxy)-10,13-dimethyl-2,3,4,7,8,9,10,11,12,13,14,15,16,

17-tetradecahydro-1H-cyclopenta[a]phenanthren-17-yl)oxy)-2-oxoacetate (**2g**, 264 mg, 0.450 mmol, 100% yield) as a hygroscopic solid.

^{1}H NMR (400 MHz, DMSO-*d*$_6$) δ/ppm: 5.77 (dddd, *J* = 17.1, 10.2, 8.3, 5.3 Hz, 1H, C*H*=CH$_2$), 5.31 – 5.17 (m, 1H, C=C*H*), 5.15 – 4.96 (m, 2H, CH=C*H*$_2$), 3.26 (dq, *J* = 10.5, 5.2 Hz, 1H), 3.11 (dd, *J* = 14.3, 5.5 Hz, 1H), 2.20 (dd, *J* = 14.4, 8.5 Hz, 1H), 2.14 – 2.03 (m, 2H), 2.01 – 1.86 (m, 3H), 1.83 – 1.70 (m, 2H), 1.64 – 1.32 (m, 8H), 1.32 – 1.16 (m, 2H), 1.11 (s, 9H (C*H*$_3$)$_3$), 1.03 (td, *J* = 13.6, 3.7 Hz, 1H), 0.94 (s, 3H, C*H*$_3$), 0.92 – 0.83 (m, 1H), 0.77 (s, 3H, C*H*$_3$).

^{13}C {^{1}H} NMR (101 MHz, DMSO-*d*$_6$) δ/ppm: 167.3, 163.4, 141.4, 134.8, 120.4, 117.3, 91.3, 72.6, 70.7, 50.9, 49.4, 46.6, 41.8, 37.4, 37.1, 36.0, 34.6, 32.7, 32.2, 31.2, 31.1, 28.3, 23.3, 20.4, 19.0, 13.9.

HRMS (nanochip-ESI/LTQ-Orbitrap) m/z: [M]$^-$ Calcd for $C_{28}H_{41}O_5^-$ 457.2959; Found 457.2938.

Cesium 2-oxo-2-((1-phenylhex-5-en-3-yl)oxy)acetate (2h)

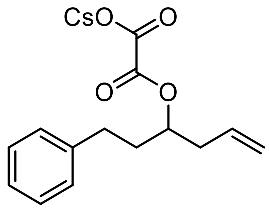

2h was prepared according to *General Procedure 4* from 1-phenylhex-5-en-3-ol (**S7h**, 529 mg, 3.00 mmol, 1.0 equiv). Obtaining cesium 2-oxo-2-((1-phenylhex-5-en-3-yl)oxy)acetate (**2h**, 1.04 g, 2.74 mmol, 91% yield) as a hygroscopic solid.

^{1}H NMR (400 MHz, DMSO-*d*$_6$) δ/ppm: 7.32 – 7.22 (m, 2H, Ar*H*), 7.22 – 7.11 (m, 3H, Ar*H*), 5.73 (ddt, *J* = 17.2, 10.2, 7.0 Hz, 1H, C*H*=CH$_2$), 5.12 – 4.94 (m, 2H, CH=C*H*$_2$), 4.73 (dq, *J* = 7.4, 6.0 Hz, 1H, CH), 2.69 – 2.51 (m, 2H, C*H*$_2$), 2.29 (ddt, *J* = 6.2, 4.7, 1.5 Hz, 2H, C*H*$_2$), 1.84 – 1.62 (m, 2H, C*H*$_2$).

^{13}C {^{1}H} NMR (101 MHz, DMSO-*d*$_6$) δ/ppm: 167.3, 162.8, 141.6, 134.1, 128.3, 128.2, 125.7, 117.5, 70.2, 38.0, 35.0, 30.8.

HRMS (nanochip-ESI/LTQ-Orbitrap) m/z: [M + Cu]$^+$ Calcd for $C_{14}H_{15}CuO_4^+$ 310.0261; Found 310.0258.

***trans-* Cesium 2-oxo-2-((2-vinylcyclohexyl)oxy)acetate (2i)**

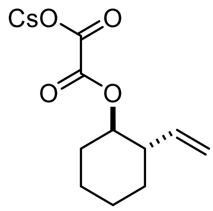

2i was prepared according to *General Procedure 4* from *trans*-vinylcyclohexanol (**S7i**, 252 mg, 2.00 mmol, 1.0 equiv). Obtaining *trans-* cesium 2-oxo-2-((2-vinylcyclohexyl)oxy)acetate (**2i**, 326 mg, 1.90 mmol, 95% yield) as a hygroscopic solid.

^{1}H NMR (400 MHz, DMSO-d_6) δ/ppm: 5.70 (ddd, J = 17.4, 10.5, 6.9 Hz, 1H, CH=CH$_2$), 5.00 (dt, J = 17.4, 1.6 Hz, 1H, CH=CH_2), 4.94 (dt, J = 10.5, 1.4 Hz, 1H, CH=CH_2), 4.41 (td, J = 9.9, 4.6 Hz, 1H, OCH), 2.20 – 2.03 (m, 1H, CH), 1.92 – 1.80 (m, 1H, CH_2), 1.77 – 1.54 (m, 3H, CH_2), 1.42 – 1.03 (m, 4H, CH_2).

^{13}C {^{1}H} NMR (101 MHz, DMSO-d_6) δ/ppm: 166.9, 162.7, 140.1, 114.7, 73.4, 45.6, 31.2, 30.3, 24.3, 24.0.

Cesium 2-(cyclopent-3-en-1-yloxy)-2-oxoacetate (2j)

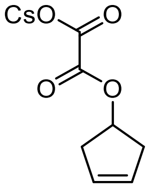

2j was prepared according to *General Procedure 4* from cyclopent-3-en-1-ol (**S7j**, 505 mg, 0.500 mL 6.00 mmol, 1.0 equiv). Obtaining cesium 2-(cyclopent-3-en-1-yloxy)-2-oxoacetate (**2j**, 1.46 g, 5.07 mmol, 84% yield) as a hygroscopic solid.

^{1}H NMR (400 MHz, DMSO-d_6) δ/ppm: 5.77 – 5.64 (m, 2H, C=CH), 5.17 (tt, J = 7.0, 2.4 Hz, 1H, OCH), 2.79 – 2.59 (m, 2H, CH_2), 2.34 – 2.15 (m, 2H, CH_2).

^{13}C {^{1}H} NMR (101 MHz, DMSO-d_6) δ/ppm: 167.4, 162.7, 128.3, 71.8, 39.4.

HRMS (ESI/QTOF) m/z: $[M]^-$ Calcd for $C_7H_7O_4^-$ 155.0350; Found 155.0348.

Cesium 2-(but-3-en-1-yloxy)-2-oxoacetate (2k)

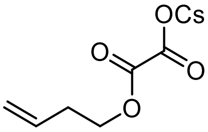

2k was prepared according to *General Procedure 4* from but-3-enol (**S7k**, 433 mg, 0.500 mL, 6.00 mmol, 1.0 equiv). The solid was washed with pentane and Et_2O obtaining cesium 2-(but-3-en-1-yloxy)-2-oxoacetate (**2k**, 1.22 g, 4.42 mmol, 74% yield) as a hygroscopic solid.

Analytical data is consistent with literature values. [3]

^{1}H NMR (400 MHz, DMSO-d_6) δ/ppm: 5.78 (ddt, J = 17.1, 10.3, 6.7 Hz, 1H, CH=CH$_2$), 5.16 – 5.00 (m, 2H, CH=CH_2), 3.95 (t, J = 6.9 Hz, 2H, CH_2), 2.31 (qt, J = 6.8, 1.4 Hz, 2H, CH_2).

^{13}C {^{1}H} NMR (101 MHz, DMSO-d_6) δ/ppm: 167.4, 162.5, 134.7, 117.0, 61.5, 32.6.

Cesium (*Z*)-2-(hex-3-en-1-yloxy)-2-oxoacetate (2l)

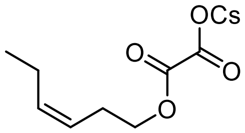

2l was prepared according to *General Procedure 4* from (*Z*)-Hex-3-en-1-ol (**S7l**, 601 mg, 0.700 mL, 6.00 mmol, 1.0 equiv), obtaining cesium (*Z*)-2-(hex-3-en-1-yloxy)-2-oxoacetate (**2l**, 1.78 g, 5.85 mmol, 98% yield) as a hygroscopic solid.

^{1}H NMR (400 MHz, DMSO-*d*$_6$) δ/ppm: 5.54 – 5.38 (m, 1H, C=C*H*), 5.37 – 5.20 (m, 1H, C=C*H*), 3.89 (t, *J* = 7.1 Hz, 2H, CH_2O), 2.39 – 2.19 (m, 2H, CH_2), 2.12 – 1.96 (m, 2H, CH_2), 0.92 (t, *J* = 7.5 Hz, 3H, CH_2CH_3).

^{13}C {^{1}H} NMR (101 MHz, DMSO-*d*$_6$) δ/ppm: 167.5, 162.5, 133.6, 124.4, 61.9, 26.4, 20.1, 14.1.

HRMS (ESI/QTOF) m/z: [M]$^-$ Calcd for $C_8H_{11}O_4^-$ 171.0663; Found 171.0664.

Cesium 2-(3-cyclohexylidenepropoxy)-2-oxoacetate (2m)

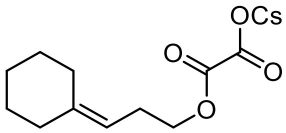

2m was prepared according to *General Procedure 4* from 3-cyclohexylidenepropanol (**S7m**, 140 mg, 1.00 mmol, 1.0 equiv). Obtaining cesium 2-(3-cyclohexylidenepropoxy)-2-oxoacetate (**2m**, 326 mg, 0.950 mmol, 95% yield) as a hygroscopic solid.

^{1}H NMR (400 MHz, DMSO-*d*$_6$) δ/ppm: 5.05 (tt, *J* = 7.2, 1.3 Hz, 1H, C=C*H*), 3.83 (t, *J* = 7.1 Hz, 2H, CH_2), 2.24 (q, *J* = 7.2 Hz, 2H, CH_2), 2.13 – 1.98 (m, 4H, CH_2), 1.55 – 1.41 (m, 6H, CH_2).

^{13}C {^{1}H} NMR (101 MHz, DMSO-*d*$_6$) δ/ppm: 167.6, 162.6, 141.4, 116.5, 62.2, 36.5, 28.2, 28.0, 27.3, 26.3, 26.3.

HRMS (nanochip-ESI/LTQ-Orbitrap) m/z: [M]$^-$ Calcd for $C_{11}H_{15}O_4^-$ 211.0976; Found 211.0969.

Cesium 2-((N-(but-3-en-1-yl)-4-methylphenyl)sulfonamido)-2-oxoacetate (5n)

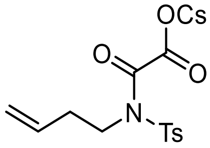

5n was prepared according to *General Procedure 5* from *N*-(but-3-en-1-yl)-4-methylbenzenesulfonamide (**S7n**, 1.80 g, 8.00 mmol, 1.0 equiv). The solid was washed with pentane and Et_2O obtaining cesium 2-((N-(but-3-en-1-yl)-4-methylphenyl)sulfonamido)-2-oxoacetate (**5n**, 3.11 g, 7.25 mmol, 91% yield) as a hygroscopic solid.

Analytical data is consistent with literature values. [3]

^{1}H NMR (400 MHz, DMSO-*d*$_6$) δ/ppm: 7.92 – 7.84 (m, 2H, Ar*H*), 7.39 (d, *J* = 8.1 Hz, 2H, Ar*H*), 5.74 (ddt, *J* = 17.0, 10.3, 6.6 Hz, 1H, C*H*=CH$_2$), 5.10 – 4.96 (m, 2H, CH=C*H*$_2$), 3.72 – 3.63 (m, 2H, C*H*$_2$), 2.48 – 2.36 (m, 5H, C*H*$_2$ and C*H*$_3$).

^{13}C {^{1}H} NMR (101 MHz, DMSO-*d*$_6$) δ/ppm: 169.0, 163.3, 144.0, 136.8, 134.8, 129.2, 128.1, 116.8, 45.2, 34.5, 21.1.

Cesium (Z)-2-((N-(hex-3-en-1-yl)-4-methylphenyl)sulfonamido)-2-oxoacetate (2o)

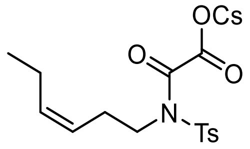

2o was prepared according to *General Procedure 5* from (*Z*)-*N*-(hex-3-en-1-yl)-4-methylbenzenesulfonamide (**S7o**, 111 mg, 0.440 mmol, 1.0 equiv), obtaining cesium (Z)-2-((N-(hex-3-en-1-yl)-4-methylphenyl)sulfonamido)-2-oxoacetate (**2o**, 185 mg, 0.410 mmol, 92% yield) as a hygroscopic solid.

^{1}H NMR (400 MHz, DMSO-*d*$_6$) δ/ppm: 7.93 – 7.81 (m, 2H, Ar*H*), 7.44 – 7.34 (m, 2H, Ar*H*), 5.56 – 5.38 (m, 1H, C=C*H*), 5.30 – 5.17 (m, 1H, C=C*H*), 3.64 – 3.52 (m, 2H, C*H*$_2$), 2.45 – 2.36 (m, 5H, C*H*$_2$ and C*H*$_3$), 2.02 (pd, *J* = 7.5, 1.5 Hz, 2H, C*H*$_2$), 0.92 (t, *J* = 7.5 Hz, 3H, C*H*$_3$).

^{13}C {^{1}H} NMR (101 MHz, DMSO-*d*$_6$) δ/ppm: 169.0, 163.4, 144.0, 136.9, 134.0, 129.2, 128.0, 124.4, 45.5, 28.4, 21.0, 20.1, 14.2.

HRMS (nanochip-ESI/LTQ-Orbitrap) m/z: [M]$^-$ Calcd for $C_{15}H_{18}NO_5S^-$ 324.0911; Found 324.0896.

Cesium 2-((N-(2-(cyclohex-1-en-1-yl)ethyl)-4-methylphenyl)sulfonamido)-2-oxoacetate (2p)

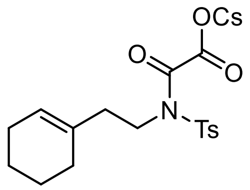

2p was prepared according to *General Procedure 5* from (*Z*)-*N*-(hex-3-en-1-yl)-4-methylbenzenesulfonamide (**S7p**, 1.40 g, 5.00 mmol, 1.0 equiv). The crude was washed with pentane and Et$_2$O obtaining cesium 2-((N-(2-(cyclohex-1-en-1-yl)ethyl)-4-methylphenyl)sulfonamido)-2-oxoacetate (**2p**, 2.11 g, 4.37 mmol, 87% yield) as a hygroscopic solid.

^{1}H NMR (400 MHz, DMSO-*d*$_6$) δ/ppm: 7.95 – 7.77 (m, 2H, Ar*H*), 7.44 – 7.34 (m, 2H, Ar*H*), 5.43 – 5.31 (m, 1H, C=C*H*), 3.74 – 3.62 (m, 2H, C*H*$_2$), 2.39 (s, 3H, C*H*$_3$), 2.35 – 2.20 (m, 2H, C*H*$_2$), 2.00 – 1.83 (m, 4H, C*H*$_2$), 1.61 – 1.43 (m, 4H, C*H*$_2$).

^{13}C {^{1}H} NMR (101 MHz, DMSO-*d*$_6$) δ/ppm: 169.0, 163.4, 143.9, 136.9, 134.5, 129.2, 128.0, 122.2, 45.0, 38.4, 27.9, 24.6, 22.4, 21.9, 21.1.

HRMS (nanochip-ESI/LTQ-Orbitrap) m/z: [M]$^-$ Calcd for $C_{17}H_{20}NO_5S^-$ 350.1068; Found 350.1051.

Cesium 2-((N-(but-3-en-1-yl)-4-(5-(p-tolyl)-3-(trifluoromethyl)-1H-pyrazol-1-yl)phenyl)sulfonamido)-2-oxoacetate (2q)

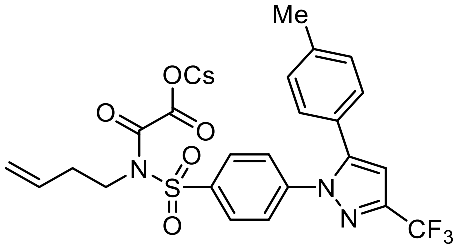

2q was prepared according to *General Procedure 5* from *N*-(but-3-en-1-yl)-4-(5-(p-tolyl)-3-(trifluoromethyl)-1H-pyrazol-1-yl)benzenesulfonamide (**S7q**, 261 mg, 0.600 mmol, 1.0 equiv). Obtaining cesium 2-((N-(but-3-en-1-yl)-4-(5-(p-tolyl)-3-(trifluoromethyl)-1H-pyrazol-1-yl)phenyl)sulfonamido)-2-oxoacetate (**2q**, 364 mg, 0.570 mmol, 65% yield) as a hygroscopic solid.

^{1}H NMR (400 MHz, DMSO-*d*$_6$) δ/ppm: 8.18 – 8.05 (m, 2H, Ar*H*), 7.60 – 7.53 (m, 2H, Ar*H*), 7.37 – 7.14 (m, 5H, Ar*H*), 5.73 (ddt, *J* = 17.0, 10.3, 6.6 Hz, 1H, C=C*H*), 5.11 – 4.98 (m, 2H, C=C*H$_2$*), 3.75 – 3.67 (m, 2H, C*H$_2$*), 2.40 (dt, *J* = 9.7, 6.6 Hz, 2H, C*H$_2$*), 2.32 (s, 3H, C*H$_3$*).

^{13}C {^{1}H} NMR (101 MHz, DMSO-*d*$_6$) δ/ppm: 169.0, 163.0, 145.4, 142.4, 139.4, 139.2, 134.7, 129.5, 129.3, 128.8, 125.7, 117.0, 106.3, 45.2, 34.2, 20.8. 3 carbons are not resolved.

^{19}F NMR (376 MHz, CDCl$_3$) δ/ppm: – 60.9.

HRMS (nanochip-ESI/LTQ-Orbitrap) m/z: [M]$^-$ Calcd for $C_{23}H_{19}F_3N_3O_5S^-$ 506.1003; Found 506.0983.

1-(But-3-en-1-yl)cyclohexyl methyl oxalate (S18)

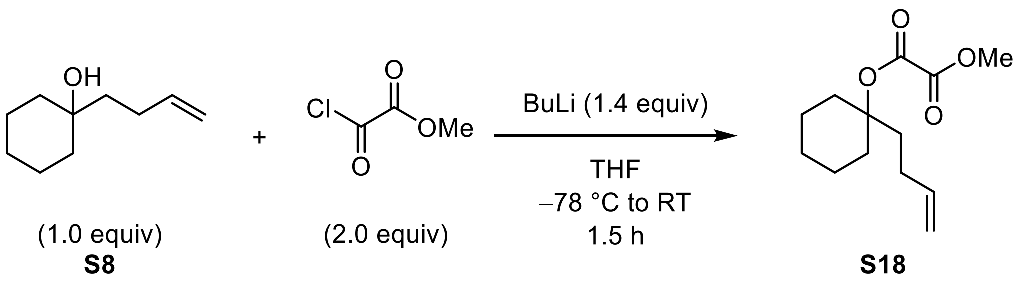

In a 25 mL, two-necked, round-bottomed flask, a solution of 1-(but-3-en-1-yl)cyclohexanol (**S8**, 0.300 g, 1.94 mmol, 1.0 equiv.) in THF (dry; 7.8 mL) was cooled to -78 °C (dry ice-acetone bath). n-BuLi (2.5 M in hexanes; 1.1 mL, 2.7 mmol, 1.4 equiv.) was added drop-wise and the resulting orange mixture was stirred at the same temperature for 0.5 hour. After this time, methyl 2-chloro-2-oxoacetate (0.36 mL, 3.9 mmol, 2.0 equiv.) was added drop-wise and the mixture, turned to off-white, was stirred at -78 °C for 1 hour, and then warmed to room temperature for another 0.5 hours. The reaction was then quenched by pouring the mixture into sat. aq. NaHCO3 (20 mL). The aqueous layer was extracted with EtOAc (3 x 20 mL). The combined organic layers were washed with brine, dried over $MgSO_4$, filtered and concentrated under vacuum. The resulting yellow crude oil was submitted to column chromatography (Biotage, 25 g SiO_2; EtOAc in pentane,

2 to 18%) obtaining 1-(but-3-en-1-yl)cyclohexyl methyl oxalate (**S18**, 0.326 g, 1.36 mmol, 70% yield) as a colorless oil.

Rf (1:9 EtOAc:Pentane) = 0.7.

^{1}H NMR (400 MHz, $CDCl_3$) δ/ppm: 5.79 (dddd, *J* = 17.1, 10.9, 8.0, 4.8 Hz, 1H, alkene CH), 5.02 (dd, *J* = 17.2, 1.7 Hz, 1H, alkene CH_2), 4.98 - 4.91 (m, 1H, alkene CH_2), 3.88 (s, 3H, CO_2Me), 2.28 (dd, *J* = 12.6, 4.8 Hz, 2H, CH_2), 2.12 - 1.97 (m, 4H, CH_2), 1.67 - 1.41 (m, 7H, CH_2), 1.38 - 1.19 (m, 1H, CH_2).

^{13}C {^{1}H} NMR (101 MHz, $CDCl_3$) δ/ppm: 159.0, 156.6, 137.9, 114.8, 88.8, 53.2, 36.4, 34.2, 27.3, 25.3, 21.7.

1-(But-3-en-1-yl)cyclohexyl methyl oxalate (6)

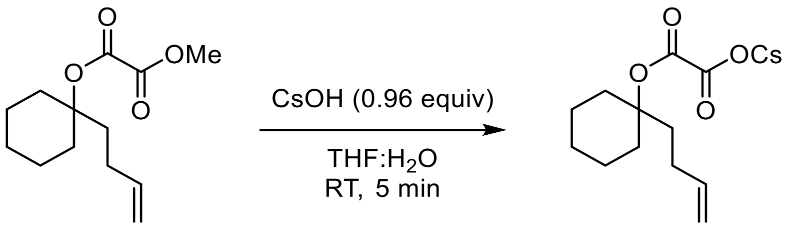

S18 (1.0 equiv) **6**

In a 25 mL round-bottomed, single-necked flask, 1-(but-3-en-1-yl)cyclohexyl methyl oxalate (**S18**, 0.326 g, 1.36 mmol, 1.0 equiv.) was dissolved in a mixture of THF (8.2 mL) and water (0.82 mL). Under vigorous stirring, aq. CsOH (1.0 M; 1.3 mL, 1.3 mmol, 0.96 equiv.) was added drop-wise. The resulting mixture was stirred for additional 5 minutes, and it was then concentrated under reduced pressure in order to remove the THF. The resulting pale yellow aqueous residue was washed with a 1:1 mixture of pentane/diethyl ether (4 x 10 mL). It was then concentrated under vacuum. The resulting colorless sticky oil was triturated with hexane to furnish a colorless solid, which was azeotropically concentrated from toluene, and dried under high vacuum overnight. Cesium 2-((1-(but-3-en-1-yl)cyclohexyl)oxy)-2-oxoacetate (**6**, 0.393 g, 1.10 mmol, 84% yield) was obtained as a colorless, hygroscopic solid.

^{1}H NMR (400 MHz, CD_3OD) δ/ppm: δ 5.81 (ddt, *J* = 16.8, 10.3, 6.4 Hz, 1H, alkene CH), 5.01 (dd, *J* = 17.3, 2.0 Hz, 1H, alkene CH_2), 4.91 (dd, *J* = 10.1, 2.0 Hz, 1H, alkene CH_2), 2.28 (d, *J* = 12.7 Hz, 1H, CH_2), 2.16 - 2.05 (m, 2H, CH_2), 2.04 - 1.95 (m, 2H, CH_2), 1.61 (ddt, *J* = 11.3, 7.4, 4.1 Hz, 4H, CH_2), 1.56 - 1.40 (m, 4H, CH_2), 1.39 - 1.21 (m, 1H, CH_2).

^{13}C {^{1}H} NMR (101 MHz, CD_3OD) δ/ppm: 166.7, 166.2, 139.8, 114.8, 86.3, 38.0, 35.6, 28.5, 26.7, 22.9.

HRMS (ESI/QTOF) m/z: $[M]^+$ Calcd for $C_{12}H_{17}O_4^-$ 225.1132; Found 225.1129.

6.2. Photoredox Catalyzed Lactonization/Lactamization Reaction

General Procedure 6

An oven-dried 7 mL dram vial equipped with a magnetic stirring bar was charged with the cesium oxalate/oxamate (300 μmol, 1.0 equiv), ArEBX (900 μmol, 3.0 equiv) and 4ClCzIPN (6.4 mg, 6.00 μmol, 2 mol%). The vial was capped with a septum and evacuated and backfilled with N_2 three times. An Ar balloon was connected to the vial and dry DMSO (degassed by Ar bubbling, 6.0 mL) was added. The septum was then replaced by a screw cap against Ar flow and then the reaction mixture was stirred under blue light irradiation (440 nm Kessil, 22 W) while cooling with a fan for 42 h.

The reaction mixture was then transferred to a separating funnel, diluted with Et_2O (15 mL) and 1:1 Brine:H_2O (20 mL) and the two layers were separated. The aqueous layer was extracted with Et_2O (3x15 mL) and the combined organic layers were washed with H_2O (20 mL) and brine (20 mL). The organic layer was then dried over $MgSO_4$, filtered and the solvents were removed under reduced pressure. The crude was then purified by flash chromatography obtaining the desired cyclized product.

3-(3-Phenylprop-2-yn-1-yl)-1-oxaspiro[4.5]decan-2-one (5a)

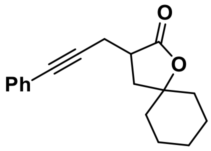

5a was prepared according to *General Procedure 6* from cesium 2-((1-allylcyclohexyl)oxy)-2-oxoacetate (**2a**, 103 mg, 300 μmol, 1.00 equiv) and PhEBX (**3a**, 313 mg, 900 μmol, 3.0 equiv). The crude was purified by flash chromatography (SiO_2, 6:5 DCM:Pentane to DCM) obtaining 3-(3-phenylprop-2-yn-1-yl)-1-oxaspiro[4.5]decan-2-one (**5a**, 61.0 mg, 227 μmol, 76% yield) as a yellow oil.

3 mmol scale:

An oven-dried 100 mL Schlenk flask equipped with a magnetic stirring bar was charged with cesium 2-((1-allylcyclohexyl)oxy)-2-oxoacetate (**2a**, 1.03 g, 3.00 mmol, 1.0 equiv), PhEBX (**3a**, 3.13 g, 9.00 mmol, 3.0 equiv) and 4ClCzIPN (**4b**, 64 mg, 60 μmol, 2.0 mol%). The flask was evacuated and backfilled with N_2 three times. DMSO (60 mL) was added and the reaction mixture was irradiated while cooling with a fan for 18 h. The reaction mixture was poured into 1:1 Brine:H_2O (200 mL) and the aqueous layer was extracted with Et_2O (3 x 150 mL). The combined organic layer was washed with water (100 mL) and brine (100 mL), dried over $MgSO_4$ and the solvents were removed under reduced pressure. The crude was purified by flash chromatography (SiO_2, 5 to 15% 1:1 DCM:Et_2O in pentane) obtaining 3-(3-phenylprop-2-yn-1-yl)-1-oxaspiro[4.5]decan-2-one (**5a**, 552 mg, 2.06 mmol, 69%) as a yellow oil.

Rf (6:5 DCM:Pentane) = 0.39.

^{1}H NMR (400 MHz, $CDCl_3$) δ/ppm: 7.41 – 7.36 (m, 2H, Ar*H*), 7.31 – 7.27 (m, 3H, Ar*H*), 3.02 (dddd, *J* = 11.0, 9.3, 7.9, 4.4 Hz, 1H, C*H*), 2.90 (dd, *J* = 17.0, 4.5 Hz, 1H, CH_2), 2.74 (dd, *J* = 17.0, 7.9 Hz, 1H, CH_2), 2.42 (dd, *J* = 12.9, 9.3 Hz, 1H, CH_2), 1.99 (dd, *J* = 12.9, 11.0 Hz, 1H, CH_2), 1.88 – 1.35 (m, 10H, CH_2).

^{13}C {^{1}H} NMR (101 MHz, $CDCl_3$) δ/ppm: 177.0, 131.8, 128.4, 128.2, 123.4, 86.1, 84.6, 82.8, 39.7, 38.8, 38.5, 36.5, 25.1, 22.8, 22.8, 21.1.

HRMS (ESI/QTOF) m/z: $[M + Na]^+$ Calcd for $C_{18}H_{20}NaO_2^+$ 291.1356; Found 291.1354.

3-(3-Phenylprop-2-yn-1-yl)-1,8-dioxaspiro[4.5]decan-2-one (2b)

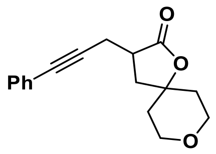

5b was prepared according to *General Procedure 6* from cesium 2-oxo-2-(4-prop-2-enyloxan-4-yl)oxyacetate (**2b**, 104 mg, 300 μmol, 1.0 equiv) and PhEBX (**3a**, 313 mg, 900 μmol, 3.0 equiv). The crude was purified by flash chromatography (SiO_2, 1:1 Et_2O:Pentane to Et_2O) obtaining 3-(3-phenylprop-2-yn-1-yl)-1,8-dioxaspiro[4.5]decan-2-one (**5b**, 70.0 mg, 259 μmol, 86% yield) as a yellow oil with residual grease.

Rf (1:1 Et_2O:Pentane) = 0.2.

^{1}H NMR (400 MHz, $CDCl_3$) δ/ppm: 7.43 – 7.35 (m, 2H, Ar*H*), 7.32 – 7.27 (m, 3H. Ar*H*), 3.94 – 3.70 (m, 4H, CH_2), 3.05 (dddd, *J* = 11.1, 9.2, 7.6, 4.4 Hz, 1H, C*H*), 2.91 (dd, *J* = 17.1, 4.5 Hz, 1H, CH_2), 2.79 (dd, *J* = 17.1, 7.6 Hz, 1H, CH_2), 2.45 (dd, *J* = 13.0, 9.3 Hz, 1H, CH_2), 2.09 (dd, *J* = 13.0, 11.1 Hz, 1H, CH_2), 2.00 – 1.73 (m, 4H, CH_2).

^{13}C {^{1}H} NMR (101 MHz, $CDCl_3$) δ/ppm: 176.4, 131.8, 128.4, 128.3, 123.2, 85.7, 83.1, 81.2, 64.5, 64.3, 39.3, 39.1, 38.1, 37.0, 21.0.

HRMS (ESI/QTOF) m/z: $[M + Na]^+$ Calcd for $C_{17}H_{18}NaO_3^+$ 293.1148; Found 293.1154.

***tert*-Butyl 2-oxo-3-(3-phenylprop-2-yn-1-yl)-1-oxa-8-azaspiro[4.5]decane-8-carboxylate (5c)**

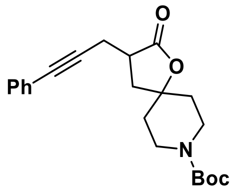

5c was prepared according to *General Procedure 6* from cesium 2-((4-allyl-1-(*tert*-butoxycarbonyl)piperidin-4-yl)oxy)-2-oxoacetate (**2c**, 114 mg, 300 μmol, 1.0 equiv) and PhEBX (**3a**, 313 mg, 900 μmol, 3.0 equiv). The crude was purified by flash chromatography (SiO_2, 5 to 40% EtOAc in Pentane) obtaining *tert*-Butyl 2-oxo-3-(3-phenylprop-2-yn-1-yl)-1-oxa-8-azaspiro[4.5]decane-8-carboxylate (**5c**, 78.0 mg, 211 μmol, 70% yield) as a yellow oil.

Rf (1:4 EtOAc:Pentane) = 0.3.

^{1}H NMR (400 MHz, $CDCl_3$) δ/ppm: 7.45 – 7.35 (m, 2H), 7.32 – 7.27 (m, 3H), 3.92 – 3.72 (m, 2H), 3.44 – 3.17 (m, 2H), 3.05 (dddd, *J* = 11.0, 9.3, 7.5, 4.5 Hz, 1H), 2.90 (dd, *J* = 17.1, 4.5 Hz, 1H), 2.79 (dd, *J* = 17.1, 7.5 Hz, 1H), 2.39 (dd, *J* = 13.0, 9.3 Hz, 1H), 2.09 (dd, *J* = 13.0, 11.1 Hz, 1H), 1.89 – 1.73 (m, 3H), 1.73 – 1.59 (m, 1H), 1.46 (s, 9H).

^{13}C {^1H} NMR (101 MHz, $CDCl_3$) δ/ppm: 176.4, 154.7, 131.8, 128.4, 128.3, 123.1, 85.6, 83.1, 81.9, 80.0, 40.4, 39.4, 38.8, 37.4, 36.2, 29.8, 28.5, 21.0.

HRMS (ESI/QTOF) m/z: [M + Na]$^+$ Calcd for $C_{22}H_{27}NNaO_4^+$ 392.1832; Found 392.1828.

3-(3-Phenylprop-2-yn-1-yl)-1-oxaspiro[4.4]nonan-2-one (5d)

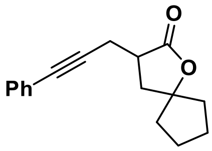

5d was prepared according to *General Procedure 6* from cesium 2-((1-allylcyclopentyl)oxy)-2-oxoacetate (**2d**, 99.0 mg, 300 μmol, 1.00 equiv) and PhEBX (**3a**, 313 mg, 900 μmol, 3.0 equiv). The crude was purified by flash chromatography (SiO_2, 1:3 DCM:Pentane to DCM) obtaining 3-(3-phenylprop-2-yn-1-yl)-1-oxaspiro[4.4]nonan-2-one (**5d**, 59.0 mg, 232 μmol, 77% yield) as a yellow oil.

Rf (DCM) = 0.6.

^1H NMR (400 MHz, $CDCl_3$) δ/ppm: 7.36 – 7.26 (m, 2H, Ar*H*), 7.25 – 7.20 (m, 3H, Ar*H*), 2.95 (dtd, *J* = 11.3, 8.4, 4.4 Hz, 1H, C*H*), 2.86 (dd, *J* = 17.0, 4.4 Hz, 1H, CH_2), 2.65 (dd, *J* = 17.0, 8.2 Hz, 1H, CH_2), 2.39 (dd, *J* = 12.8, 8.6 Hz, 1H, CH_2), 2.23 (dd, *J* = 12.8, 11.3 Hz, 1H, CH_2), 2.06 – 1.57 (m, 8H).

^{13}C {^1H} NMR (101 MHz, $CDCl_3$) δ/ppm: 177.0, 131.8, 128.4, 128.2, 123.4, 93.1, 86.1, 82.7, 40.8, 39.1, 38.7, 38.2, 24.2, 23.6, 20.8.

HRMS (ESI/QTOF) m/z: [M + Na]$^+$ Calcd for $C_{17}H_{18}NaO_2^+$ 277.1199; Found 277.1198.

3-(3-Phenylprop-2-yn-1-yl)-1-oxaspiro[4.11]hexadecan-2-one (5e)

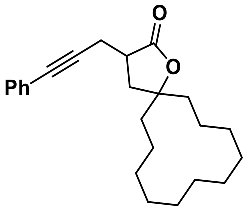

5e was prepared according to *General Procedure 6* from cesium 2-((1-allylcyclododecyl)oxy)-2-oxoacetate (**2e**, 129 mg, 300 μmol, 1.0 equiv) and PhEBX (**3a**, 313 mg, 900 μmol, 3.0 equiv). The crude was purified by flash chromatography (SiO_2, 3:1 Pentane:DCM to DCM) obtaining 3-(3-phenylprop-2-yn-1-yl)-1-oxaspiro[4.11]hexadecan-2-one (**5e**, 86.0 mg, 244 μmol, 81% yield) as a white amorphous solid.

Rf (DCM) = 0.5.

^1H NMR (400 MHz, $CDCl_3$) δ/ppm: 7.42 – 7.35 (m, 2H, Ar*H*), 7.32 – 7.27 (m, 3H, Ar*H*), 3.08 – 2.95 (m, 1H, C*H*), 2.90 (dd, *J* = 17.0, 4.4 Hz, 1H, CH_2), 2.72 (dd, *J* = 17.0, 8.0 Hz, 1H, CH_2), 2.37 (dd, *J* = 12.9, 9.2 Hz, 1H, CH_2), 2.14 – 1.88 (m, 2H, CH_2), 1.80 – 1.22 (m, 21H, CH_2).

^{13}C {^{1}H} NMR (101 MHz, $CDCl_3$) δ/ppm: 177.1, 131.8, 128.4, 128.2, 123.4, 88.0, 86.2, 82.8, 39.8, 38.5, 35.2, 32.8, 26.3, 26.3, 26.0, 22.6, 22.6, 22.3, 22.2, 21.1, 19.7, 19.4.

HRMS (ESI/QTOF) m/z: $[M + Na]^+$ Calcd for $C_{24}H_{32}NaO_2^+$ 375.2295; Found 375.2292.

4'-(3-Phenylprop-2-yn-1-yl)dihydro-5'H-spiro[adamantane-2,2'-furan]-5'-one (5f)

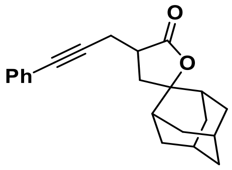

5f was prepared according to *General Procedure 6* from cesium 2-(((1r,3r)-2-allyladamantan-2-yl)oxy)-2-oxoacetate (**2f**, 119 mg, 300 µmol, 1.00 equiv) and PhEBX (**3a**, 313 mg, 900 µmol, 3.0 equiv). The crude was purified by flash chromatography (SiO_2, 1:3 to 3:1 DCM:Pentane) obtaining 4'-(3-phenylprop-2-yn-1-yl)dihydro-5'H-spiro[adamantane-2,2'-furan]-5'-one (**5f**, 71.0 mg, 222 µmol, 74% yield) as a white amorphous solid.

Rf (DCM) = 0.56.

^{1}H NMR (400 MHz, $CDCl_3$) δ/ppm: 7.42 – 7.35 (m, 2H, Ar*H*), 7.32 – 7.27 (m, 3H, Ar*H*), 3.00 (dddd, *J* = 10.8, 9.2, 7.9, 4.4 Hz, 1H, C*H*), 2.91 (dd, *J* = 17.0, 4.4 Hz, 1H, CH_2), 2.78 – 2.68 (m, 2H, CH_2), 2.31 – 2.14 (m, 2H, Ad*H*), 1.94 (dd, *J* = 13.1, 10.9 Hz, 1H, CH_2), 1.90 – 1.71 (m, 10H, Ad*H*), 1.68 – 1.59 (m, 2H, Ad*H*).

^{13}C {^{1}H} NMR (101 MHz, $CDCl_3$) δ/ppm: 176.9, 131.8, 128.4, 128.2, 123.4, 88.9, 86.2, 82.8, 39.9, 39.4, 37.6, 37.2, 36.2, 35.5, 34.0, 33.8, 33.0, 26.9, 26.8, 21.2.

HRMS (ESI/QTOF) m/z: $[M + Na]^+$ Calcd for $C_{22}H_{24}NaO_2^+$ 343.1669; Found 343.1661.

(3S,9S,14S,17R)-3-(*Tert*-butoxy)-10,13-dimethyl-4'-(3-phenylprop-2-yn-1-yl)-1,2,3,3',4,4',7,8,9,10,11,12,13,14,15,16-hexadecahydro-5'H-spiro[cyclopenta[a]phenanthrene-17,2'-furan]-5'-one (5g)

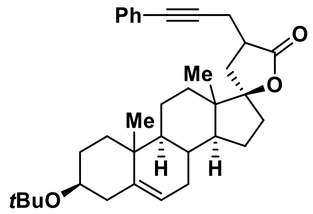

5g was prepared according to *General Procedure 6* from (3S,8R,9S,10R,13S,14S,17R)-17-allyl-3-(tert-butoxy)-10,13-dimethyl-2,3,4,7,8,9,10,11,12,13,14,15,16,17-tetradecahydro-1*H*-cyclopenta[α]phenanthren-17-yl ethyl oxalate (**2g**, 177 mg, 300 µmol, 1.0 equiv) and PhEBX (**3a**, 313 mg, 900 µmol, 3.0 equiv). The crude was purified by flash chromatography (SiO_2, 2 to 13% EtOAc in Pentane) obtaining (3S,9S,14S,17R)-3-(tert-butoxy)-10,13-dimethyl-4'-(3-phenylprop-2-yn-1-yl)-1,2,3,3',4,4',7,8,9, 10,11,12,13,14,15,16-hexadecahydro-5'H-spiro[cyclopenta[a]phenanthrene-17,2'-furan]-5'-one (**5g**, Major: 58.0 mg, 113 µmol, 38% yield; Minor: 18.0 mg, 34.9 µmol, 12% yield, minor impurities) as white amorphous solids. The diastereomeric ratio was determined to be 3:1 by ^{1}H NMR spectroscopy of the crude mixture before column purification.

Major

Rf (10% EtOAc in Pentane) = 0.4.

^{1}H NMR (400 MHz, CDCl$_3$) δ/ppm: 7.42 – 7.35 (m, 2H, Ar*H*), 7.33 – 7.27 (m, 3H, Ar*H*), 5.30 (d, *J* = 5.1 Hz, 1H, C=C*H*), 3.30 (tt, *J* = 10.8, 4.7 Hz, 1H, *t*BuOC*H*), 3.04 (dtd, *J* = 12.2, 8.1, 4.3 Hz, 1H, COC*H*), 2.93 (dd, *J* = 17.1, 4.3 Hz, 1H), 2.71 (dd, *J* = 17.1, 8.1 Hz, 1H), 2.37 (t, *J* = 12.1 Hz, 1H), 2.27 (ddd, *J* = 13.6, 11.1, 2.5 Hz, 1H), 2.18 – 2.08 (m, 2H), 2.08 – 1.97 (m, 2H), 1.92 (ddd, *J* = 14.1, 9.5, 5.8 Hz, 1H), 1.83 (dt, *J* = 13.2, 3.5 Hz, 1H), 1.77 – 1.39 (m, 9H), 1.56 (s, 3H), 1.19 (s, 9H, C(CH_3)$_3$), 1.11 – 1.02 (m, 1H), 1.01 (s, 3H, CH_3), 0.99 (s, 3H, CH_3).

^{13}C {^{1}H} NMR (101 MHz, CDCl$_3$) δ/ppm: 177.0, 142.3, 131.8, 128.4, 128.2, 123.4, 120.4, 94.1, 86.2, 82.8, 73.5, 71.5, 50.5, 50.1, 44.8, 42.1, 39.4, 38.0, 36.8, 35.1, 34.4, 32.6, 32.1, 31.7, 31.4, 28.6, 23.5, 20.7, 20.5, 19.5, 14.8.

HRMS (ESI/QTOF) m/z: [M + Na]$^+$ Calcd for $C_{35}H_{46}NaO_3^+$ 537.3339; Found 537.3336.

Minor

Rf (10% EtOAc in Pentane) = 0.3.

^{1}H NMR (400 MHz, CDCl$_3$) δ/ppm: 7.44 – 7.37 (m, 2H, Ar*H*), 7.34 – 7.27 (m, 3H, Ar*H*), 5.34 – 5.28 (m, 1H, C=C*H*), 3.30 (tt, *J* = 11.0, 4.9 Hz, 1H, *t*BuOC*H*), 3.01 – 2.82 (m, 1H, COC*H*), 2.72 (tt, *J* = 17.4, 8.6 Hz, 1H), 2.54 – 2.33 (m, 1H), 2.28 (ddd, *J* = 13.7, 11.2, 2.5 Hz, 1H), 2.14 (ddd, *J* = 13.6, 5.0, 2.1 Hz, 1H), 2.10 – 1.98 (m, 2H), 1.89 – 1.78 (m, 2H), 1.73 – 1.44 (m, 9H), 1.19 (d, *J* = 1.0 Hz, 9H, C(CH_3)$_3$), 1.10 – 1.04 (m, 1H), 1.00 (s, 3H, CH_3), 0.93 (s, 3H, CH_3).

^{13}C {^{1}H} NMR (101 MHz, CDCl$_3$) δ/ppm: 177.2, 142.1, 131.8, 129.0, 128.4, 128.0, 123.4, 120.4, 94.5, 86.1, 82.8, 73.5, 71.4, 50.4, 50.0, 46.3, 42.1, 40.6, 38.8, 37.9, 37.3, 36.8, 32.4, 31.6, 31.4, 28.6, 23.1, 21.3, 20.6, 19.5, 14.5.

5-Phenethyl-3-(3-phenylprop-2-yn-1-yl)dihydrofuran-2(3*H*)-one (5h)

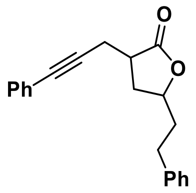

5h was prepared according to *General Procedure 6* from cesium 2-oxo-2-((1-phenylhex-5-en-3-yl)oxy)acetate (**2h**, 114 mg, 300 µmol, 1.0 equiv) and PhEBX (**3a**, 313 mg, 900 µmol, 3.0 equiv). The crude was purified by flash chromatography (SiO_2, 5 to 15% 1:1 DCM:Et_2O in pentane) obtaining 5-phenethyl-3-(3-phenylprop-2-yn-1-yl)dihydrofuran-2(3*H*)-one (**5h**, 50.0 mg, 164 µmol, 55% yield, 3:2 dr) as a yellow oil with residual grease.

The diastereomeric ratio was determined by ^{1}H NMR spectroscopy of the crude mixture by integrating the peaks at 4.40 and 4.61 ppm, respectively.

The diastereomers were then separated by preparatory TLC (1:1 Et_2O:Pentane).

Major

Rf (1:1:8 DCM:Et_2O:Pentane) = 0.3.

^{1}H NMR (400 MHz, $CDCl_3$) δ/ppm: 7.43 – 7.36 (m, 2H, Ar*H*), 7.34 – 7.25 (m, 5H, Ar*H*), 7.24 – 7.16 (m, 3H, Ar*H*), 4.40 (ddt, *J* = 10.3, 8.0, 5.2 Hz, 1H, OC*H*), 3.00 – 2.81 (m, 3H, CH_2 and COC*H*), 2.81 – 2.69 (m, 2H, CH_2), 2.58 (ddd, *J* = 12.8, 8.4, 5.7 Hz, 1H, CH_2), 2.10 (dtd, *J* = 14.0, 8.6, 5.6 Hz, 1H, CH_2), 2.04 – 1.86 (m, 2H, CH_2).

^{13}C {^{1}H} NMR (101 MHz, $CDCl_3$) δ/ppm: 177.2, 140.8, 131.8, 128.7, 128.6, 128.4, 128.2, 126.3, 123.3, 85.9, 82.9, 78.0, 40.6, 37.4, 34.2, 31.7, 20.7.

HRMS (ESI/QTOF) m/z: $[M + Na]^+$ Calcd for $C_{21}H_{20}NaO_2^+$ 327.1356; Found 327.1356.

Minor

Rf (1:1:8 DCM:Et_2O:Pentane) = 0.3.

^{1}H NMR (400 MHz, $CDCl_3$) δ/ppm: 7.39 – 7.32 (m, 2H, Ar*H*), 7.31 – 7.26 (m, 5H, Ar*H*), 7.24 – 7.15 (m, 3H, Ar*H*), 4.61 (tt, *J* = 8.8, 4.9 Hz, 1H, OC*H*), 3.01 – 2.89 (m, 1H, COC*H*), 2.88 – 2.68 (m, 4H, CH_2), 2.43 (dt, *J* = 13.3, 7.6 Hz, 1H, CH_2), 2.19 (ddd, *J* = 13.7, 9.5, 4.9 Hz, 1H, CH_2), 2.10 – 1.97 (m, 1H, CH_2), 1.97 – 1.81 (m, 1H, CH_2).

^{13}C {^{1}H} NMR (101 MHz, $CDCl_3$) δ/ppm: 177.8, 140.8, 131.8, 128.7, 128.6 128.4, 128.3, 126.4, 123.2, 85.7, 82.8, 78.2, 39.0, 37.7, 32.8, 31.8, 21.4.

3-(3-Phenylprop-2-yn-1-yl)hexahydrobenzofuran-2(3H)-one (5i)

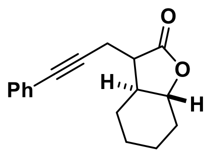

5i was prepared according to *General Procedure 6* from cesium 2-oxo-2-(((1R,2S)-2-vinylcyclohexyl)oxy)acetate (**2i**, 99.0 mg, 300 µmol, 1.0 equiv) and PhEBX (**3a**, 313 mg, 900 µmol, 3.0 equiv). The crude was purified by flash chromatography (SiO_2, 5 to 15% 1:1 DCM:Et_2O in pentane) obtaining 3-(3-phenylprop-2-yn-1-yl)hexahydrobenzofuran-2(3H)-one (**5i**, 43.0 mg, 169 µmol, 56% yield) as an amorphous white solid.

Rf (1:1:8 DCM:Et_2O:Pentane) = 0.5.

^{1}H NMR (400 MHz, $CDCl_3$) δ/ppm: 7.44 – 7.33 (m, 2H, Ar*H*), 7.32 – 7.27 (m, 3H, Ar*H*), 3.80 (ddd, *J* = 11.5, 10.4, 3.8 Hz, 1H, OC*H*), 2.97 (dd, *J* = 17.3, 4.2 Hz, 1H, COC*H*), 2.69 (dd, *J* = 17.3, 8.0 Hz, 1H, C*H*$_2$), 2.52 (ddd, *J* = 12.3, 8.0, 4.2 Hz, 1H, C*H*), 2.35 – 2.21 (m, 2H, C*H*$_2$), 2.02 – 1.74 (m, 3H, C*H*$_2$), 1.47 – 1.29 (m, 4H, C*H*$_2$).

^{13}C {^{1}H} NMR (101 MHz, $CDCl_3$) δ/ppm: 177.0, 131.7, 128.4, 128.1, 123.4, 86.3, 83.0, 82.7, 49.9, 45.7, 30.3, 28.1, 25.4, 24.1, 19.0.

HRMS (ESI/QTOF) m/z: [M + Na]$^+$ Calcd for $C_{17}H_{18}NaO_2^+$ 277.1199; Found 277.1200.

5-(Phenylethynyl)-2-oxabicyclo[2.2.1]heptan-3-one (5j)

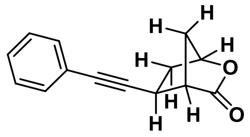

5j was prepared according to *General Procedure 6* from cesium 2-(cyclopent-3-en-1-yloxy)-2-oxoacetate (**2j**, 86.0 mg, 300 µmol, 1.0 equiv) and PhEBX (**3a**, 313 mg, 900 µmol, 3.0 equiv). The crude was purified by flash chromatography (SiO_2, 5 to 15% 1:1 DCM:Et_2O in pentane) obtaining 5-(phenylethynyl)-2-oxabicyclo[2.2.1]heptan-3-one (**5j**, 16.0 mg, 75.3 µmol, 25% yield) as an amorphous white solid.

Rf (15% 1:1 DCM:Et_2O in Pentane) = 0.22.

^{1}H NMR (400 MHz, $CDCl_3$) δ/ppm: 7.42 – 7.35 (m, 2H, Ar*H*), 7.30 (dd, *J* = 5.1, 1.9 Hz, 3H, Ar*H*), 5.03 – 4.95 (m, 1H, OC*H*), 3.14 (ddd, *J* = 8.8, 4.3, 1.3 Hz, 1H, alkyne-C*H*), 3.07 (s, 1H, COC*H*), 2.42 (ddd, *J* = 13.7, 8.5, 2.4 Hz, 1H, ax C*H*), 2.35 – 2.26 (m, 1H, C*H*$_2$), 2.20 (dd, *J* = 10.8, 1.3 Hz, 1H, C*H*$_2$), 2.06 (ddd, *J* = 13.6, 4.1, 2.0 Hz, 1H, eq. C*H*).

^{13}C {^{1}H} NMR (101 MHz, $CDCl_3$) δ/ppm: 176.5, 131.7, 128.5, 128.5, 122.9, 89.8, 82.6, 80.6, 48.6, 38.6, 38.3, 28.0.

HRMS (APCI/QTOF) m/z: [M + H]$^+$ Calcd for $C_{14}H_{13}O_2^+$ 213.0910; Found 213.0905.

3-(3-Phenylprop-2-yn-1-yl)dihydrofuran-2(3*H*)-one (5k)

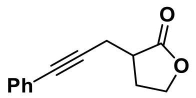

5k was prepared according to *General Procedure 6* from cesium 2-(but-3-en-1-yloxy)-2-oxoacetate (**2k**, 83.0 mg, 300 µmol, 1.0 equiv) and PhEBX (**3a**, 313 mg, 900 µmol, 3.0 equiv). The crude was purified by flash chromatography (SiO_2, 5 to 15% 1:1 DCM:Et_2O in pentane) obtaining 3-(3-phenylprop-2-yn-1-yl)dihydrofuran-2(3*H*)-one (**5k**, 37.0 mg, 185 µmol, 62% yield) as a yellow oil.

Rf (1:1:8 DCM:Et_2O:Pentane) = 0.4.

^{1}H NMR (400 MHz, $CDCl_3$) δ/ppm: 7.43 – 7.36 (m, 2H, Ar*H*), 7.33 – 7.27 (m, 3H, Ar*H*), 4.44 (td, *J* = 8.8, 2.9 Hz, 1H, OC*H*$_2$), 4.26 (ddd, *J* = 9.6, 9.0, 6.9 Hz, 1H, OC*H*$_2$), 2.93 (dd, *J* = 16.2, 4.2 Hz, 1H, C*H*$_2$), 2.90 – 2.81 (m, 1H, C*H*$_2$), 2.76 (dd, *J* = 16.3, 7.3 Hz, 1H, C*H*$_2$), 2.54 (dddd, *J* = 12.7, 8.6, 6.9, 2.9 Hz, 1H, C*H*), 2.33 (dtd, *J* = 12.7, 9.8, 8.7 Hz, 1H, C*H*$_2$).

^{13}C {^{1}H} NMR (101 MHz, $CDCl_3$) δ/ppm: 177.9, 131.8, 128.4, 128.2, 123.2, 85.7, 82.8, 66.8, 39.0, 28.0, 20.8.

HRMS (ESI/QTOF) m/z: $[M + H]^+$ Calcd for $C_{13}H_{13}O_2^+$ 201.0910; Found 201.0913.

3-(1-Phenylpent-1-yn-3-yl)dihydrofuran-2(3*H*)-one (5l)

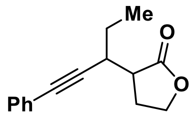

5l was prepared according to *General Procedure 6* from cesium (Z)-2-(hex-3-en-1-yloxy)-2-oxoacetate (**2l**, 91.0 mg, 300 µmol, 1.0 equiv) and PhEBX (**3a**, 313 mg, 900 µmol, 3.0 equiv). The crude was purified by flash chromatography (SiO_2, 5 to 40% 1:1 DCM:Et_2O in pentane) obtaining 3-(1-phenylpent-1-yn-3-yl)dihydrofuran-2(3*H*)-one (**5l**, 48.0 mg, 210 µmol, 70% yield, 4:3 dr) as a yellow oil.

The diastereomeric ratio was determined by ^{1}H NMR spectroscopy of the crude mixture.

The diastereomers were then separated by preparatory TLC (1:1 Et_2O:Pentane).

<u>Major</u>

Rf (1:1:8 DCM:Et_2O:Pentane) = 0.2.

^{1}H NMR (400 MHz, $CDCl_3$) δ/ppm: 7.43 – 7.33 (m, 2H, Ar*H*), 7.32 – 7.26 (m, 3H, Ar*H*), 4.44 (td, *J* = 8.8, 3.4 Hz, 1H, OC*H*$_2$), 4.25 (td, *J* = 9.0, 7.3 Hz, 1H, OC*H*$_2$), 3.17 (ddd, *J* = 8.1, 7.1, 4.2 Hz, 1H, C*H*), 2.72 (td, *J* = 9.4, 4.2 Hz, 1H, C*H*$_2$), 2.47 (dq, *J* = 12.7, 9.1 Hz, 1H, C*H*$_2$), 2.40 – 2.29 (m, 1H, C*H*), 1.74 – 1.64 (m, 2H, C*H*$_2$CH$_3$), 1.12 (t, *J* = 7.4 Hz, 3H, C*H*$_3$).

^{13}C {^{1}H} NMR (101 MHz, $CDCl_3$) δ/ppm: 178.1, 131.9, 128.4, 128.2, 123.3, 88.5, 84.2, 67.0, 43.4, 35.1, 27.3, 24.8, 12.3.

HRMS (ESI/QTOF) m/z: $[M + Na]^+$ Calcd for $C_{15}H_{16}NaO_2^+$ 251.1043; Found 251.1049.

<u>Minor</u>

Rf (1:1:8 DCM:Et_2O:Pentane) = 0.2.

^{1}H NMR (400 MHz, CDCl$_3$) δ/ppm: 7.44 – 7.36 (m, 2H, Ar*H*), 7.32 – 7.27 (m, 3H, Ar*H*), 4.42 (td, *J* = 8.8, 4.0 Hz, 1H, OC*H$_2$*), 4.25 (td, *J* = 8.7, 7.6 Hz, 1H, OC*H$_2$*), 3.05 – 2.86 (m, 2H, C*H*$_2$), 2.47 (dddd, *J* = 13.0, 9.1, 7.5, 4.0 Hz, 1H, C*H*$_2$), 2.39 – 2.28 (m, 1H, C*H*), 1.79 (ddq, *J* = 12.9, 10.3, 7.4 Hz, 1H, C*H*$_2$), 1.69 – 1.59 (m, 1H, C*H*$_2$), 1.12 (t, *J* = 7.3 Hz, 3H, C*H*$_3$).

^{13}C {^{1}H} NMR (101 MHz, CDCl$_3$) δ/ppm: 177.1, 131.8, 128.4, 128.1, 123.4, 89.7, 83.2, 66.8, 43.4, 35.1, 26.1, 24.7, 12.7.

3-(1-(Phenylethynyl)cyclohexyl)dihydrofuran-2(3*H*)-one (5m)

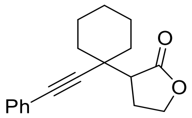

5m was prepared according to *General Procedure 6* from cesium 2-(3-cyclohexylidenepropoxy)-2-oxoacetate (**2m**, 103 mg, 300 µmol, 1.00 equiv) and PhEBX (**3a**, 313 mg, 900 µmol, 3.0 equiv). The crude was purified by flash chromatography (SiO$_2$, 2:3 DCM:Pentane) obtaining 3-(1-(phenylethynyl)cyclohexyl)dihydrofuran-2(3H)-one (**5m**, 32.0 mg, 119 µmol, 40% yield) as a yellow oil with residual grease.

Rf (2:3 DCM:Pentane) = 0.3.

^{1}H NMR (400 MHz, CDCl$_3$) δ/ppm: 7.43 – 7.37 (m, 2H, Ar*H*), 7.32 – 7.27 (m, 3H, Ar*H*), 4.39 (td, *J* = 8.5, 4.1 Hz, 1H, OC*H$_2$*), 4.20 (td, *J* = 8.6, 7.6 Hz, 1H, OC*H$_2$*), 2.62 – 2.45 (m, 3H, C*H*$_2$), 2.44 – 2.35 (m, 1H, C*H*), 1.87 – 1.51 (m, 8H, C*H*$_2$), 1.49 – 1.37 (m, 1H, C*H*$_2$).

^{13}C {^{1}H} NMR (101 MHz, CDCl$_3$) δ/ppm: 176.5, 131.9, 128.4, 128.0, 123.5, 91.7, 84.5, 66.4, 49.0, 39.4, 36.8, 34.8, 25.9, 25.8, 23.3, 22.9.

HRMS (ESI/QTOF) m/z: $[M + Na]^+$ Calcd for $C_{18}H_{20}NaO_2^+$ 291.1356; Found 291.1350.

3-(3-Phenylprop-2-yn-1-yl)-1-tosylpyrrolidin-2-one (5n)

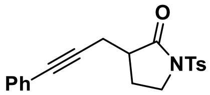

5n was prepared according to *General Procedure 6* from cesium 2-((*N*-(but-3-en-1-yl)-4-methylphenyl)sulfonamido)-2-oxoacetate (**2n**, 129 mg, 300 µmol, 1.0 equiv) and PhEBX (**3a**, 313 mg, 900 µmol, 3.0 equiv). The crude was purified by flash chromatography (SiO$_2$, 5 to 40% 1:1 DCM:Et$_2$O in pentane) obtaining 3-(3-phenylprop-2-yn-1-yl)-1-tosylpyrrolidin-2-one (**5n**, 75.0 mg, 212 µmol, 71% yield) as a white solid.

The compound was recrystallized from CDCl$_3$ for X-Ray Crystallography.

Rf (1:1:8 DCM:Et$_2$O:Pentane) = 0.2.

^{1}H NMR (400 MHz, CDCl$_3$) δ/ppm: 7.94 – 7.86 (m, 2H, Ar*H*), 7.32 – 7.21 (m, 7H, Ar*H*), 4.01 (ddd, *J* = 9.8, 8.8, 3.1 Hz, 1H, NC*H*$_2$), 3.80 (ddd, *J* = 9.9, 8.7, 7.4 Hz, 1H, NC*H*$_2$), 2.82 – 2.70 (m, 2H, C*H*$_2$), 2.64 (dd, *J* = 17.7, 8.2 Hz, 1H, C*H*$_2$), 2.42 – 2.31 (m, 4H, C*H*$_2$ and C*H*$_3$), 2.10 (dq, *J* = 12.9, 8.9 Hz, 1H, C*H*).

^{13}C {^1H} NMR (101 MHz, $CDCl_3$) δ/ppm: 173.7, 145.2, 135.3, 131.7, 129.8, 128.3, 128.1, 128.1, 123.2, 85.5, 82.6, 45.6, 42.4, 24.1, 21.8, 20.8.

HRMS (APCI/QTOF) m/z: $[M + H]^+$ Calcd for $C_{20}H_{20}NO_3S^+$ 354.1158; Found 354.1155.

3-(1-Phenylpent-1-yn-3-yl)-1-tosylpyrrolidin-2-one (5o)

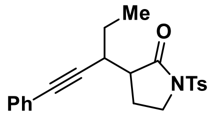

5o was prepared according to *General Procedure 6* from cesium cesium (Z)-2-((N-(hex-3-en-1-yl)-4-methylphenyl)sulfonamido)-2-oxoacetate (**2o**, 137 mg, 300 µmol, 1.0 equiv) and PhEBX (**3a**, 313 mg, 900 µmol, 3.0 equiv). The crude was purified by flash chromatography (SiO_2, 5 to 20% EtOAc in pentane) obtaining 3-(1-phenylpent-1-yn-3-yl)-1-tosylpyrrolidin-2-one (**5o**, major: 46.0 mg, 121 µmol, 40% yield; minor: 26.0 mg, 68.1 µmol, 23% yield, 2:1 dr) as a white amorphous solid.

The diastereomeric ratio was determined by ^1H NMR spectroscopy of the crude mixture by integrating the peaks at 2.33 and 2.41 ppm, respectively.

<u>Major</u>

Rf (2:8 EtOAc:Pentane) = 0.6.

^1H NMR (400 MHz, $CDCl_3$) δ/ppm: 7.41 – 7.21 (m, 5H, Ar*H*), 7.21 – 7.12 (m, 4H, Ar*H*), 3.99 (ddd, *J* = 9.8, 8.5, 4.3 Hz, 1H, NCH_2), 3.85 (dt, *J* = 9.7, 7.9 Hz, 1H, NCH_2), 3.04 (ddd, *J* = 8.9, 6.3, 3.9 Hz, 1H, C*H*), 2.33 (s, 3H, CH_3), 2.30 – 2.14 (m, 2H, C*H*), 1.65 – 1.46 (m, 2H, CH_2), 1.03 (t, *J* = 7.3 Hz, 3H, CH_3).

^{13}C {^1H} NMR (101 MHz, $CDCl_3$) δ/ppm: 173.9, 145.0, 135.4, 131.8, 129.7, 128.3, 128.1, 128.1, 123.2, 88.4, 83.8, 46.7, 45.9, 35.1, 26.8, 21.8, 20.9, 12.2.

HRMS (ESI/QTOF) m/z: $[M + H]^+$ Calcd for $C_{22}H_{24}NO_3S^+$ 382.1471; Found 382.1484.

<u>Minor</u>

Rf (2:8 EtOAc:Pentane) = 0.7.

^1H NMR (400 MHz, $CDCl_3$) δ/ppm: 7.96 – 7.89 (m, 2H, Ar*H*), 7.34 – 7.22 (m, 7H, Ar*H*), 4.09 – 3.95 (m, 1H, NCH_2), 3.91 – 3.75 (m, 1H, NCH_2), 2.93 – 2.73 (m, 2H, C*H*), 2.41 (s, 3H, CH_3), 2.38 – 2.25 (m, 1H, CH_2), 2.19 – 2.09 (m, 1H, CH_2), 1.80 – 1.62 (m, 1H, CH_2), 1.50 – 1.40 (m, 1H, CH_2), 1.03 (t, *J* = 7.3 Hz, 3H, CH_3).

^{13}C {^1H} NMR (101 MHz, $CDCl_3$) δ/ppm: 173.2, 145.2, 135.3, 131.8, 129.7, 128.3, 128.2, 128.1, 123.3, 89.4, 83.0, 46.7, 45.7, 35.7, 24.5, 22.3, 21.8, 12.6.

HRMS (ESI/QTOF) m/z: $[M + H]^+$ Calcd for $C_{22}H_{24}NO_3S^+$ 382.1471; Found 382.1475.

6-(Phenylethynyl)-2-tosyl-2-azaspiro[4.5]decan-1-one (5p)

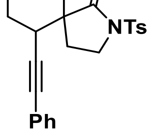

5p was prepared according to *General Procedure 6* from cesium 2-((N-(2-(cyclohex-1-en-1-yl)ethyl)-4-methylphenyl)sulfonamido)-2-oxoacetate (**2p**, 145 mg, 300 µmol, 1.0 equiv) and PhEBX (**3a**, 313 mg, 900 µmol, 3.0 equiv). The crude was purified by flash chromatography (SiO_2, 5 to 40% 1:1 DCM:Et_2O in pentane) obtaining 6-(phenylethynyl)-2-tosyl-2-azaspiro[4.5]decan-1-one (**5p**, 93.0 mg, 228 µmol, 76% yield, 6:1 dr) as a white amorphous solid.

The diastereomeric ratio was determined by ^{1}H NMR spectroscopy of the crude mixture by integrating the CH_3 peaks.

Rf (1:1:8 DCM:Et_2O:Pentane) = 0.2.

<u>Major:</u>

^{1}H NMR (400 MHz, $CDCl_3$) δ/ppm: 7.96 – 7.76 (m, 2H, Ar*H*), 7.35 – 7.21 (m, 3H, Ar*H*), 7.14 – 7.09 (m, 2H, Ar*H*), 7.04 (d, *J* = 8.1 Hz, 2H, Ar*H*), 3.92 (ddt, *J* = 9.7, 6.0, 3.0 Hz, 2H, NC*H*$_2$), 2.78 (dd, *J* = 12.0, 3.7 Hz, 1H, C*H*), 2.43 (ddd, *J* = 12.9, 8.5, 6.4 Hz, 1H, C*H*$_2$), 2.28 (s, 3H, C*H*$_3$), 2.10 – 1.88 (m, 2H, C*H*$_2$), 1.70 (dddd, *J* = 14.0, 10.0, 4.7, 2.5 Hz, 2H, C*H*$_2$), 1.62 – 1.50 (m, 2H, C*H*$_2$), 1.46 – 1.33 (m, 3H, C*H*$_2$).

^{13}C {^{1}H} NMR (101 MHz, $CDCl_3$) δ/ppm: 176.8, 144.9, 135.3, 131.7, 129.6, 128.2, 128.1, 128.0, 123.2, 89.6, 82.6, 50.4, 44.9, 35.4, 33.1, 29.1, 25.5, 24.8, 21.8, 21.2.

HRMS (ESI/QTOF) m/z: $[M + Na]^+$ Calcd for $C_{24}H_{25}NNaO_3S^+$ 430.1447; Found 430.1442.

3-(3-Phenylprop-2-yn-1-yl)-1-((4-(5-(*p*-tolyl)-3-(trifluoromethyl)-1*H*-pyrazol-1-yl)phenyl)sulfonyl)pyrrolidin-2-one (5q)

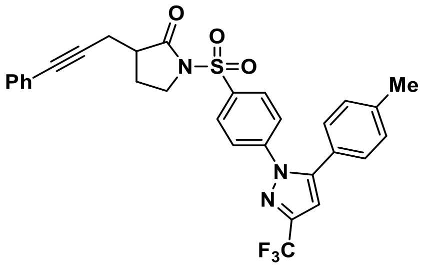

5q was prepared according to *General Procedure 6* from cesium 2-((*N*-(but-3-en-1-yl)-4-(5-(*p*-tolyl)-3-(trifluoromethyl)-1*H*-pyrazol-1-yl)phenyl)sulfonamido)-2-oxoacetate (**2q**, 192 mg, 300 µmol, 1.0 equiv) and PhEBX (**3a**, 313 mg, 900 µmol, 3.0 equiv). The crude was purified by flash chromatography (SiO_2, 5 to 20% EtOAc in pentane) obtaining 3-(3-phenylprop-2-yn-1-yl)-1-((4-(5-(*p*-tolyl)-3-(trifluoromethyl)-1*H*-pyrazol-1-yl)phenyl)sulfonyl)pyrrolidin-2-one (**5q**, 81.0 mg, 144 µmol, 48% yield) as a yellow amorphous solid.

Rf (2:8 EtOAc:pentane) = 0.25.

^{1}H NMR (400 MHz, $CDCl_3$) δ/ppm: 8.03 – 7.94 (m, 2H, Ar*H*), 7.40 – 7.33 (m, 2H, Ar*H*), 7.25 – 7.20 (m, 5H, Ar*H*), 7.19 – 7.13 (m, 2H, Ar*H*), 7.10 – 7.02 (m, 2H, Ar*H*), 6.73 (s, 1H), 3.99 (ddd, *J* = 9.9, 8.8, 3.4 Hz, 1H, NC*H*$_2$), 3.81 (dt, *J* = 9.8, 7.9 Hz, 1H, NC*H*$_2$), 2.82 – 2.61 (m, 3H, C*H*$_2$), 2.38 (s, 3H, C*H*$_3$), 2.22 – 2.13 (s, 1H, C*H*$_2$), 2.17 – 2.07 (m, 1H, C*H*$_2$).

^{13}C {^{1}H} NMR (101 MHz, $CDCl_3$) δ/ppm: 173.7, 145.4, 143.7, 140.1, 137.3, 131.7, 129.9, 129.2, 128.8, 128.4, 128.2, 125.8, 125.3, 123.0, 106.6, 85.2, 82.8, 45.8, 42.3, 24.1, 21.5, 20.9. Two carbons are not resolved.

^{19}F NMR (376 MHz, $CDCl_3$) δ/ppm: – 62.5.

HRMS (ESI/QTOF) m/z: $[M + Na]^+$ Calcd for $C_{30}H_{24}F_3N_3NaO_3S^+$ 586.1383; Found 586.1385.

3-(3-(*p*-Tolyl)prop-2-yn-1-yl)-1,8-dioxaspiro[4.5]decan-2-one (5r)

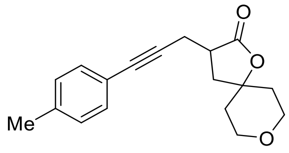

5r was prepared according to *General Procedure 6* from cesium 2-oxo-2-(4-prop-2-enyloxan-4-yl)oxyacetate (**2b**, 104 mg, 300 µmol, 1.0 equiv) and 1-[4-methylphenylethynyl]-1,2-benziodoxol-3-(1*H*)-one (**3b**, 326 mg, 900 µmol, 3.0 equiv). The crude was purified by flash chromatography (SiO_2, 3:3:14 to 1:1:2 DCM:Et_2O:Pentane) obtaining 3-(3-(p-tolyl)prop-2-yn-1-yl)-1,8-dioxaspiro[4.5]decan-2-one (**5r**, 75.0 mg, 264 µmol, 88% yield) as a yellow oil with residual grease. The compound couldn't be purified further.

Rf (1:1:2 DCM:Et_2O:Pentane) = 0.4.

^1H NMR (400 MHz, $CDCl_3$) δ/ppm: 7.32 – 7.23 (m, 2H, Ar*H*), 7.09 (d, *J* = 7.9 Hz, 2H, Ar*H*), 4.04 – 3.66 (m, 4H, CH_2) 3.04 (dddd, *J* = 11.1, 9.3, 7.6, 4.5 Hz, 1H, C*H*), 2.90 (dd, *J* = 17.0, 4.5 Hz, 1H, CH_2), 2.77 (dd, *J* = 17.1, 7.6 Hz, 1H, CH_2), 2.44 (dd, *J* = 13.0, 9.3 Hz, 1H, CH_2), 2.34 (s, 3H, CH_3), 2.09 (dd, *J* = 13.0, 11.1 Hz, 1H, CH_2), 2.00 – 1.73 (m, 4H, CH_2).

^{13}C {^1H} NMR (101 MHz, $CDCl_3$) δ/ppm: 176.5, 138.4, 131.7, 129.2, 120.1, 84.9, 83.2, 81.2, 64.6, 64.3, 39.4, 39.1, 38.1, 37.1, 21.6, 21.0.

HRMS (ESI/QTOF) m/z: $[M + Na]^+$ Calcd for $C_{18}H_{20}NaO_3^+$ 307.1305; Found 307.1308.

3-(3-(*p*-Tolyl)prop-2-yn-1-yl)-1-tosylpyrrolidin-2-one (5s)

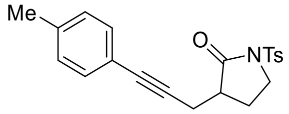

5s was prepared according to *General Procedure 6* from cesium 2-((*N*-(but-3-en-1-yl)-4-methylphenyl)sulfonamido)-2-oxoacetate (**2n**, 129 mg, 300 µmol, 1.0 equiv) and 1-[4-methylphenylethynyl]-1,2-benziodoxol-3-(1*H*)-one (**3b**, 326 mg, 900 µmol, 3.0 equiv). The crude was purified by flash chromatography (SiO_2, 10 to 30% Et_2O in pentane) obtaining 3-(3-(*p*-tolyl)prop-2-yn-1-yl)-1-tosylpyrrolidin-2-one (**5s**, 67.0 mg, 182 µmol, 61% yield) as a yellow amorphous solid.

Rf (2:8 Et_2O:Pentane) = 0.3.

^1H NMR (400 MHz, $CDCl_3$) δ/ppm: 7.99 – 7.80 (m, 2H, Ar*H*), 7.25 – 7.21 (m, 2H, Ar*H*), 7.15 (d, *J* = 8.1 Hz, 2H, Ar*H*), 7.07 (d, *J* = 7.9 Hz, 2H, Ar*H*), 4.00 (td, *J* = 9.3, 3.2 Hz, 1H, NCH_2), 3.91 – 3.74 (m, 1H, NCH_2), 2.79 – 2.68 (m, 2H, CH_2), 2.62 (dd, *J* = 17.7, 8.3 Hz, 1H, CH_2), 2.48 – 2.35 (m, 1H, CH_2), 2.39 (s, 3H, CH_3), 2.34 (s, 3H, CH_3), 2.10 (dq, *J* = 12.8, 8.9 Hz, 1H, C*H*).

^{13}C {^1H} NMR (101 MHz, $CDCl_3$) δ/ppm: 173.7, 145.2, 138.2, 135.3, 131.6, 129.8, 129.1, 128.2, 120.1, 84.7, 82.7, 45.7, 42.6, 24.2, 21.8, 21.6, 20.8.

HRMS (ESI/QTOF) m/z: [M + Na]$^+$ Calcd for $C_{21}H_{21}NNaO_3S^+$ 390.1134; Found 390.1131.

3-(3-(3-Fluorophenyl)prop-2-yn-1-yl)-1,8-dioxaspiro[4.5]decan-2-one (5t)

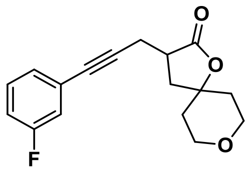

5t was prepared according to *General Procedure 6* from cesium 2-oxo-2-(4-prop-2-enyloxan-4-yl)oxyacetate (**2b**, 104 mg, 300 µmol, 1.0 equiv) and 1-[3-fluorophenylethynyl]-1,2-benziodoxol-3-(1*H*)-one (**3c**, 330 mg, 900 µmol, 3.0 equiv). The crude was purified by flash chromatography (SiO_2, 15% 1:1 DCM:Et_2O in pentane to 1:1 DCM:Et_2O) obtaining 3-(3-(3-fluorophenyl)prop-2-yn-1-yl)-1,8-dioxaspiro[4.5]decan-2-one (**5t**, 53.0 mg, 184 µmol, 61% yieldc) as a yellow oil.

Rf (66% 1:1 DCM:Et_2O in pentane) = 0.5.

^{1}H NMR (400 MHz, $CDCl_3$) δ/ppm: 7.35 – 7.25 (m, 1H, Ar*H*), 7.19 (dt, *J* = 7.8, 1.3 Hz, 1H, Ar*H*), 7.11 (ddd, *J* = 9.5, 2.6, 1.4 Hz, 1H, Ar*H*), 7.04 (tdd, *J* = 8.4, 2.7, 1.0 Hz, 1H, Ar*H*), 3.99 – 3.75 (m, 4H, C*H*$_2$), 3.23 – 3.00 (m, 1H, C*H*), 2.94 (dd, *J* = 17.1, 4.5 Hz, 1H, C*H*$_2$), 2.81 (dd, *J* = 17.1, 7.6 Hz, 1H, C*H*$_2$), 2.48 (dd, *J* = 13.0, 9.3 Hz, 1H, C*H*$_2$), 2.09 (dd, *J* = 13.0, 11.2 Hz, 1H, C*H*$_2$), 2.02 – 1.79 (m, 4H, C*H*$_2$).

^{13}C {^{1}H} NMR (101 MHz, $CDCl_3$) δ/ppm: 176.3, 162.4 (d, *J* = 246.4 Hz), 130.0 (d, *J* = 8.7 Hz), 127.6 (d, *J* = 3.0 Hz), 125.0 (d, *J* = 9.5 Hz), 118.6 (d, *J* = 22.6 Hz), 115.6 (d, *J* = 21.2 Hz), 86.8, 81.9 (d, *J* = 3.4 Hz), 81.2, 64.5, 64.2, 39.2, 39.1, 38.1, 36.9, 20.9.

^{19}F NMR (376 MHz, $CDCl_3$) δ/ppm: – 113.0.

ESI/QTOF) m/z: [M + Na]$^+$ Calcd for $C_{17}H_{17}FNaO_3^+$ 311.1054; Found 311.1051.

3-(3-(2-Bromophenyl)prop-2-yn-1-yl)-1,8-dioxaspiro[4.5]decan-2-one (5u)

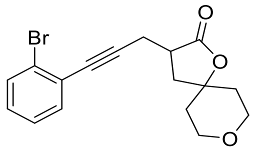

5u was prepared according to *General Procedure 6* from cesium 2-oxo-2-(4-prop-2-enyloxan-4-yl)oxyacetate (**2b**, 104 mg, 300 µmol, 1.0 equiv) and 1-[2-bromophenylethynyl]-1,2-benziodoxol-3-(1*H*)-one (**3d**, 384 mg, 900 µmol, 3.0 equiv). The crude was purified by flash chromatography (SiO_2, 1:1:8 to 1:1:2 DCM:Et_2O:pentane) 3-(3-(2-bromophenyl)prop-2-yn-1-yl)-1,8-dioxaspiro[4.5]decan-2-one (**5u**, 88.0 mg, 252 µmol, 84% yield) as a yellow amorphous solid.

Rf (1:1:2 DCM:Et_2O:pentane) = 0.4.

^{1}H NMR (400 MHz, $CDCl_3$) δ/ppm: 7.55 (dd, *J* = 8.0, 1.2 Hz, 1H, Ar*H*), 7.41 (dd, *J* = 7.7, 1.7 Hz, 1H, Ar*H*), 7.28 – 7.20 (m, 1H, Ar*H*), 7.14 (td, *J* = 7.7, 1.8 Hz, 1H, Ar*H*), 3.95 – 3.69 (m, 4H, CH_2), 3.07 (dddd, *J* = 11.5, 9.2, 7.3, 4.5 Hz, 1H, C*H*), 2.90 (qd, *J* = 17.2, 5.9 Hz, 2H, CH_2), 2.47 (dd, *J* = 13.0, 9.2 Hz, 1H, CH_2), 2.19 (dd, *J* = 12.9, 11.3 Hz, 1H, CH_2), 2.01 – 1.75 (m, 4H, CH_2).

^{13}C {^{1}H} NMR (101 MHz, $CDCl_3$) δ/ppm: 176.3, 133.6, 132.4, 129.4, 127.2, 125.6, 125.2, 90.6, 81.7, 81.2, 64.5, 64.2, 39.2, 39.0, 38.0, 36.9, 21.0.

HRMS (ESI/QTOF) m/z: $[M + Na]^+$ Calcd for $C_{17}H_{17}BrNaO_3^+$ 371.0253; Found 371.0258.

3-(3-(2-Bromophenyl)prop-2-yn-1-yl)-1-tosylpyrrolidin-2-one (5v)

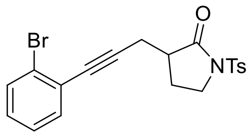

5v was prepared according to *General Procedure 6* from cesium 2-((*N*-(but-3-en-1-yl)-4-methylphenyl)sulfonamido)-2-oxoacetate (**2n**, 129 mg, 300 µmol, 1.0 equiv) and 1-[2-bromophenylethynyl]-1,2-benziodoxol-3-(1*H*)-one (**3d**, 384 mg, 900 µmol, 3.0 equiv). The crude was purified by flash chromatography (SiO_2, 1:1:8 to 1:1:2 DCM:Et_2O:pentane) obtaining 3-(3-(2-bromophenyl)prop-2-yn-1-yl)-1-tosylpyrrolidin-2-one (**5v**, 93.0 mg, 215 µmol, 72% yield) as a yellow amorphous solid.

Rf (1:1:8 DCM:Et_2O:pentane) = 0.1.

^{1}H NMR (400 MHz, $CDCl_3$) δ/ppm: 7.98 – 7.82 (m, 2H, Ar*H*), 7.54 (dd, *J* = 7.9, 1.3 Hz, 1H, Ar*H*), 7.30 (dd, *J* = 7.6, 1.8 Hz, 1H, Ar*H*), 7.24 – 7.18 (m, 3H, Ar*H*), 7.14 (td, *J* = 7.7, 1.8 Hz, 1H, Ar*H*), 4.04 (ddd, *J* = 10.0, 8.9, 2.8 Hz, 1H, NCH_2), 3.81 (ddd, *J* = 10.0, 9.0, 7.4 Hz, 1H, NCH_2), 2.90 – 2.64 (m, 3H, CH_2), 2.49 – 2.39 (m, 1H, CH_2), 2.36 (s, 3H, CH_3), 2.21 (dq, *J* = 12.9, 9.1 Hz, 1H, C*H*).

^{13}C {^{1}H} NMR (101 MHz, $CDCl_3$) δ/ppm: 173.5, 145.2, 135.3, 133.5, 132.4, 129.7, 129.3, 128.2, 127.1, 125.6, 125.3, 90.6, 81.3, 45.6, 42.4, 24.1, 21.8, 20.9.

HRMS (ESI/QTOF) m/z: $[M + Na]^+$ Calcd for $C_{20}H_{18}BrNNaO_3S^+$ 454.0083; Found 454.0082.

3-(3-(4-(Trifluoromethyl)phenyl)prop-2-yn-1-yl)-1,8-dioxaspiro[4.5]decan-2-one (5w)

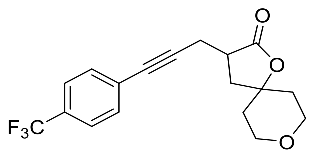

5w was prepared according to *General Procedure 6* from cesium 2-oxo-2-(4-prop-2-enyloxan-4-yl)oxyacetate (**2b**, 104 mg, 300 µmol, 1.0 equiv) and 1-[4-trifluoromethylphenylethynyl]-1,2-benziodoxol-3-(1*H*)-one (**3e**, 375 mg, 900 µmol, 3.0 equiv). The crude was purified by flash chromatography (SiO_2, 1:1:11 to 1:1:0 DCM:Et_2O:Pentane) obtaining 3-(3-(4-(trifluoromethyl)phenyl)prop-2-yn-1-yl)-1,8-dioxaspiro[4.5]decan-2-one (**5w**, 69.0 mg, 204 µmol, 68% yield) as a yellow oil.

Rf (3:3:4 DCM:Et_2O:Pentane) = 0.4.

^{1}H NMR (400 MHz, $CDCl_3$) δ/ppm: 7.55 (d, *J* = 8.7 Hz, 2H, Ar*H*), 7.52 – 7.44 (m, 2H, Ar*H*), 4.03 – 3.66 (m, 4H, CH_2), 3.06 (dddd, *J* = 11.2, 9.2, 7.5, 4.5 Hz, 1H, C*H*), 2.93 (dd, *J* = 17.2, 4.5 Hz, 1H, CH_2), 2.87 – 2.78 (m, 1H, CH_2), 2.46 (dd, *J* = 13.0, 9.3 Hz, 1H, CH_2), 2.06 (dd, *J* = 13.0, 11.2 Hz, 1H, CH_2), 1.97 – 1.73 (m, 4H, CH_2).

^{13}C {^{1}H} NMR (101 MHz, $CDCl_3$) δ/ppm: 176.2, 132.0, 127.0, 125.4 (q, *J* = 3.9 Hz), 88.4, 81.9, 81.2, 64.5, 64.2, 39.2, 38.1, 37.0, 21.0. 3 carbons are not resolved.

^{19}F NMR (376 MHz, $CDCl_3$) δ/ppm: – 62.8.

HRMS (ESI/QTOF) m/z: $[M + Na]^+$ Calcd for $C_{18}H_{17}F_3NaO_3^+$ 361.1022; Found 361.1028.

1-Tosyl-3-(3-(4-(trifluoromethyl)phenyl)prop-2-yn-1-yl)pyrrolidin-2-one (5x)

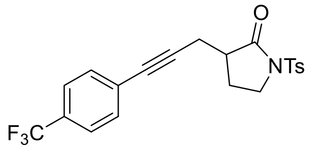

5x was prepared according to *General Procedure 6* from cesium 2-((*N*-(but-3-en-1-yl)-4-methylphenyl)sulfonamido)-2-oxoacetate (**2n**, 129 mg, 300 µmol, 1.0 equiv) and 1-[4-trifluoromethylphenylethynyl]-1,2-benziodoxol-3-(1*H*)-one (**3e**, 375 mg, 900 µmol, 3.0 equiv). The crude was purified by flash chromatography (SiO_2, 2:8 to 8:2 Et_2O:Pentane) obtaining 3-(3-(4-(trifluoromethyl)phenyl)prop-2-yn-1-yl)-1,8-dioxaspiro[4.5]decan-2-one (**5x**, 56.0 mg, 133 µmol, 44% yield) as a yellow oil. The compound couldn't be purified further.

Rf (1:1 Et_2O:Pentane) = 0.4.

^{1}H NMR (400 MHz, $CDCl_3$) δ/ppm: 7.97 – 7.88 (m, 2H, Ar*H*), 7.52 (d, *J* = 8.1 Hz, 2H, Ar*H*), 7.36 (d, *J* = 8.1 Hz, 2H, Ar*H*), 7.29 – 7.20 (m, 2H, Ar*H*), 4.02 (ddd, *J* = 9.9, 8.8, 2.8 Hz, 1H, NCH_2), 3.79 (ddd, *J* = 9.9, 8.9, 7.3 Hz, 1H, NCH_2), 2.84 – 2.60 (m, 3H, CH_2), 2.41 – 2.32 (m, 4H, CH_2 and CH_3), 2.17 – 2.00 (m, 1H, C*H*).

^{13}C {^{1}H} NMR (101 MHz, $CDCl_3$) δ/ppm: 173.4, 145.4, 135.3, 132.0, 129.9, 129.8, 128.2, 125.3 (q, *J* = 3.6 Hz), 88.3, 81.5, 45.5, 42.3, 24.2, 21.8, 20.8. Two carbons are not resolved.

^{19}F NMR (376 MHz, $CDCl_3$) δ/ppm: – 62.8.

HRMS (ESI/QTOF) m/z: $[M + Na]^+$ Calcd for $C_{21}H_{18}F_3NNaO_3S^+$ 444.0852; Found 444.0843.

Methyl 4-(3-(2-oxo-1,8-dioxaspiro[4.5]decan-3-yl)prop-1-yn-1-yl)benzoate (5y)

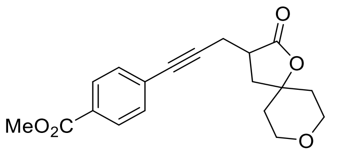

5y was prepared according to *General Procedure 6* from cesium 2-oxo-2-(4-prop-2-enyloxan-4-yl)oxyacetate (**2b**, 104 mg, 300 µmol, 1.0 equiv) and methyl 4-(2-oxo-1λ^3-benzo[d][1,3]iodaoxol-1(2H)-yl)benzoate (**3f**, 366 mg, 900 µmol, 3.0 equiv). The crude was purified by flash chromatography (SiO_2, 30 to 60% EtOAc in pentane) obtaining methyl 4-(3-(2-oxo-1,8-dioxaspiro[4.5]decan-3-yl)prop-1-yn-1-yl)benzoate (**5y**, 25.0 mg, 76.1 µmol, 25% yield) as a yellow oil with residual grease.

Rf (1:1 EtOAc:pentane) = 0.4.

^{1}H NMR (400 MHz, $CDCl_3$) δ/ppm: 8.00 – 7.89 (m, 2H, Ar*H*), 7.47 – 7.39 (m, 2H, Ar*H*), 3.91 (s, 3H, C*H*$_3$), 3.89 – 3.75 (m, 4H, C*H*$_2$), 3.06 (dddd, *J* = 11.5, 9.2, 7.6, 4.5 Hz, 1H, C*H*), 2.94 (dd, *J* = 17.1, 4.5 Hz, 1H, C*H*$_2$), 2.81 (dd, *J* = 17.2, 7.6 Hz, 1H, C*H*$_2$), 2.46 (dd, *J* = 13.0, 9.2 Hz, 1H, C*H*$_2$), 2.14 – 2.03 (m, 1H, C*H*$_2$), 1.91 (ddd, *J* = 14.2, 9.6, 4.9 Hz, 1H, C*H*$_2$), 1.86 – 1.76 (m, 3H, C*H*$_2$).

^{13}C {^{1}H} NMR (101 MHz, $CDCl_3$) δ/ppm: 176.2, 166.6, 131.7, 129.7, 129.6, 127.9, 89.0, 82.5, 81.2, 64.5, 64.2, 52.4, 39.2, 38.1, 38.1, 37.0, 21.1.

HRMS (ESI/QTOF) m/z: $[M + H]^+$ Calcd for $C_{19}H_{21}O_5^+$ 329.1384; Found 329.1384.

Methyl 4-(3-(2-oxo-1-tosylpyrrolidin-3-yl)prop-1-yn-1-yl)benzoate (5z)

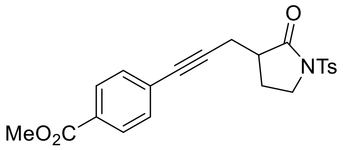

5z was prepared according to *General Procedure 6* from cesium 2-((*N*-(but-3-en-1-yl)-4-methylphenyl)sulfonamido)-2-oxoacetate (**2n**, 129 mg, 300 µmol, 1.0 equiv) and methyl 4-(2-oxo-1λ^3-benzo[d][1,3]iodaoxol-1(2H)-yl)benzoate (**3f**, 366 mg, 900 µmol, 3.0 equiv). The crude was purified by flash chromatography (SiO_2, 10 to 30% EtOAc in pentane) obtaining Methyl 4-(3-(2-oxo-1-tosylpyrrolidin-3-yl)prop-1-yn-1-yl)benzoate (**5z**, 55.0 mg, 134 µmol, 45% yield) as a yellow amorphous solid.

Rf (2:8 EtOAc:Pentane) = 0.3.

^{1}H NMR (400 MHz, CD_3CN) δ/ppm: 7.97 – 7.89 (m, 2H, Ar*H*), 7.88 – 7.81 (m, 2H, Ar*H*), 7.36 – 7.31 (m, 2H, Ar*H*), 7.31 – 7.25 (m, 2H, Ar*H*), 3.97 (ddd, *J* = 9.7, 9.0, 2.7 Hz, 1H, NC*H*), 3.88 (s, 3H, C*H*$_3$), 3.81 (ddd, *J* = 9.7, 9.0, 7.4 Hz, 1H, NC*H*), 2.83 (ddt, *J* = 10.1, 8.8, 5.3 Hz, 1H, C*H*$_2$), 2.75 – 2.62 (m, 2H, C*H*$_2$), 2.34 (s, 3H, C*H*$_3$), 2.33 – 2.27 (m, 1H, C*H*$_2$), 2.12 – 2.02 (m, 1H, C*H*).

^{13}C {^{1}H} NMR (101 MHz, CD_3CN) δ/ppm: 174.9, 167.1, 146.4, 136.6, 132.5, 130.6, 130.6, 130.3, 128.8, 128.7, 90.5, 82.2, 52.9, 46.6, 42.7, 24.3, 21.6, 20.8.

HRMS (ESI/QTOF) m/z: $[M + Na]^+$ Calcd for $C_{22}H_{21}NNaO_5S^+$ 434.1033; Found 434.1034.

3-(3-Phenylprop-2-yn-1-yl)-1-oxaspiro[5.5]undecan-2-one (7)

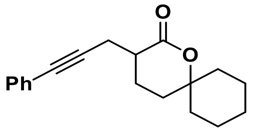

7 was prepared according to *General Procedure 6* from cesium 2-((1-(but-3-en-1-yl)cyclohexyl)oxy)-2-oxoacetate (**6**, 54 mg, 0.15 mmol, 1.0 equiv) and PhEBX (**3a**, 157 mg, 450 µmol, 3.0 equiv). The crude was purified by flash chromatography (SiO_2, 2 to 50% EtOAc in Pentane) obtaining 3-(3-phenylprop-2-yn-1-yl)-1-oxaspiro[5.5]undecan-2-one (**7**, 19.0 mg, 67.0 µmol, 45% yield) as a pale yellow oil with residual grease.

Rf (1:4 EtOAc:Pentane) = 0.2.

^{1}H NMR (400 MHz, $CDCl_3$) δ/ppm: 7.42 – 7.32 (m, 2H, Ar*H*), 7.28 (dt, *J* = 4.8, 1.8 Hz, 3H, Ar*H*), 2.98 – 2.79 (m, 2H, CH_2), 2.63 (dtd, *J* = 10.7, 7.2, 4.7 Hz, 1H, C*H*), 2.19 – 1.88 (m, 4H, CH_2), 1.87 – 1.66 (m, 4H, CH_2), 1.62 – 1.40 (m, 5H, CH_2), 1.40 – 1.27 (m, 1H, CH_2).

^{13}C {^{1}H} NMR (101 MHz, $CDCl_3$) δ/ppm: 172.6, 131.7, 128.4, 128.0, 123.6, 87.1, 83.6, 82.7, 39.8, 38.8, 36.6, 25.5, 22.5, 21.9, 21.8.

HRMS (ESI/QTOF) m/z: $[M + Na]^+$ Calcd for $C_{19}H_{22}NaO_2^+$ 305.1512; Found 305.1504.

6.3. Product Modifications

2-(2-Oxo-1-oxaspiro[4.5]decan-3-yl)acetic acid (8)

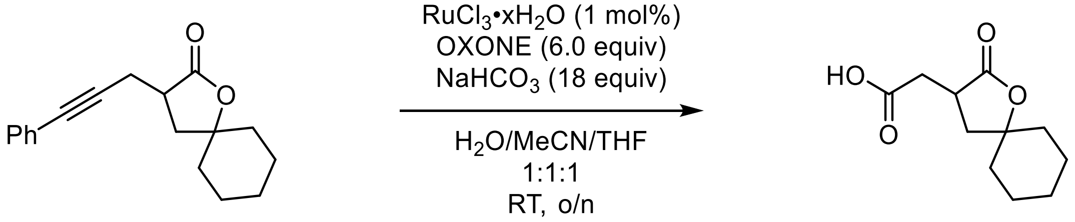

The reaction was not carried out under inert conditions.

Following a modified reported procedure,[17] a 2-5 mL Biotage microwave vial equipped with a magnetic stirring bar was charged with 3-(3-phenylprop-2-yn-1-yl)-1-oxaspiro[4.5]decan-2-one (**5a**, 54 mg, 0.20 mmol, 1.0 equiv), THF (1.0 mL), MeCN (1.0 mL) and water (1.0 mL). Na_2CO_3 (302 mg, 3.60 mmol, 18 equiv) and OXONE (738 mg, 1.20 mmol, 6.0 equiv) were added. The suspension was stirred for 3 min and then $RuCl_3$•xH_2O (0.4 mg, 0.002 mmol, 1 mol%) was added. The reaction was stirred overnight and then it was quenched with aq. $NaHSO_3$ (10%, 5.0 mL), acidified to pH < 2 with aq. HCl (1.0 M) and extracted with EtOAc (3 x 5 mL). The combined organic layer was dried over $MgSO_3$, filtered and the solvent were

[17] Yang, D.; Chen, F.; Dong, Z.-M.; Zhang, D.-W. Ruthenium-Catalyzed Oxidative Cleavage of Alkynes to Carboxylic Acids. *J. Org. Chem*. **2004**, *69* (6), 2221–2223.

removed under reduced pressure. Mesitylene (9.2 μL, 0.066 mmol, 0.33 equiv) was added and the crude was analyzed by ^{1}H NMR spectroscopy, obtaining 2-(2-oxo-1-oxaspiro[4.5]decan-3-yl)acetic acid (**8**) in 75% ^{1}H NMR yield.

Analytical data is consistent with literature values.[18]

^{1}H NMR (400 MHz, MeOD) δ/ppm: 3.14 (dddd, *J* = 11.5, 9.4, 7.5, 4.2 Hz, 1H, C*H*), 2.75 (dd, *J* = 17.3, 4.3 Hz, 1H, C*H*$_2$), 2.60 (dd, *J* = 17.4, 7.6 Hz, 1H, C*H*$_2$), 2.42 (dd, *J* = 12.6, 9.5 Hz, 1H, C*H*$_2$), 1.88 – 1.40 (m, 11H, C*H*$_2$).

^{13}C {^{1}H} NMR (101 MHz, MeOD) δ/ppm: 176.1, 174.6, 86.2, 39.0, 37.8, 36.8, 36.7, 35.0, 34.3, 23.8, 23.1.

HRMS (ESI/QTOF) m/z: $[M + H_{-1}]^-$ Calcd for $C_{11}H_{15}O_4^-$ 211.0976; Found 211.0976.

1-(2-Hydroxy-5-phenylpent-4-yn-1-yl)cyclohexan-1-ol (9)

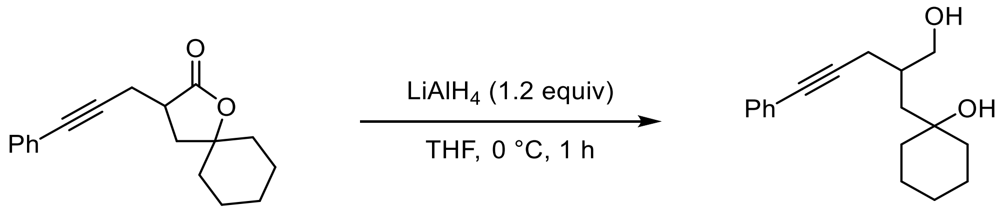

To a solution of 3-(3-phenylprop-2-yn-1-yl)-1-oxaspiro[4.5]decan-2-one (**5a**, 54 mg, 0.20 mmol, 1.0 equiv) in THF (2.0 mL) at 0 °C, $LiAlH_4$ (2.4 M in THF, 0.1 mL, 0.24 mmol, 1.2 equiv) was added. The reaction mixture was stirred at this temperature for 1 h. The reaction was acidified to pH < 3 with aq. HCl (1.0 M) and the aqueous layer was extracted with DCM (3 x 5 mL). The combined organic layer was washed with $NaHCO_3$ and brine, dried over $MgSO_4$ and the solvents removed under reduced pressure obtaining 1-(2-hydroxy-5-phenylpent-4-yn-1-yl)cyclohexan-1-ol (**9**, 49 mg, 0.18 mmol, 90% yield) as a colorless oil.

^{1}H NMR (400 MHz, $CDCl_3$) δ/ppm: 7.43 – 7.33 (m, 2H, Ar*H*), 7.33 – 7.23 (m, 3H, Ar*H*), 3.82 (dd, *J* = 10.8, 4.2 Hz, 1H, C*H*$_2$), 3.57 (dd, *J* = 10.8, 7.3 Hz, 1H, C*H*$_2$), 2.70 (bs, 2H, O*H*), 2.52 – 2.29 (m, 2H, C*H*$_2$), 2.20 – 2.06 (m, 1H, C*H*), 1.86 (dd, *J* = 14.9, 3.4 Hz, 1H, C*H*$_2$), 1.73 – 1.44 (m, 10H, C*H*$_2$), 1.37 – 1.27 (m, 1H, C*H*$_2$).

^{13}C {^{1}H} NMR (101 MHz, $CDCl_3$) δ/ppm: 131.7, 128.4, 127.8, 123.9, 88.5, 82.0, 71.8, 67.1, 44.5, 40.0, 36.5, 36.0, 25.8, 24.1, 22.7, 22.4.

HRMS (ESI/QTOF) m/z: $[M + Na]^+$ Calcd for $C_{18}H_{24}NaO_2^+$ 295.1669; Found 295.1671.

2-(1-Hydroxy-5-phenylpent-4-yn-2-yl)cyclohexan-1-ol (10)

[18] Gérardy, R.; Winter, M.; Horn, C. R.; Vizza, A.; Van Hecke, K.; Monbaliu, J.-C. M. Continuous-Flow Preparation of γ-Butyrolactone Scaffolds from Renewable Fumaric and Itaconic Acids under Photosensitized Conditions. *Org. Process Res. Dev.* **2017**, *21* (12), 2012–2017.

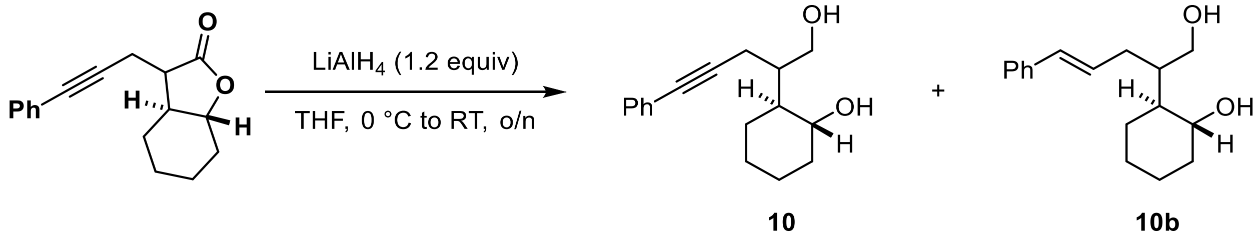

To a solution of 3-(3-phenylprop-2-yn-1-yl)hexahydrobenzofuran-2(3H)-one (**5i**, 19 mg, 0.075 mmol, 1.0 equiv) in THF (0.75 mL) at 0 °C, $LiAlH_4$ (2.4 M in THF, 0.04 mL, 0.09 mmol, 1.2 equiv) was added. The reaction mixture was allowed to warm to room temperature and it was stirred overnight. The reaction was acidified to pH < 3 with aq. HCl (1.0 M) and the aqueous layer was extracted with DCM (3 x 5 mL). The combined organic layer was washed with $NaHCO_3$ and brine, dried over $MgSO_4$ and the solvents removed under reduced pressure. The crude was purified by flash chromatography (50% EtOAc:Pentane) obtaining 2-(1-hydroxy-5-phenylpent-4-yn-2-yl)cyclohexan-1-ol (**10**, 14 mg of a mixture of 77% **10** and 23% **10b**, calculated for **10**: 41.6 μmol, 56% yield) as a white amorphous solid.

The mixture was determined by ^{1}H NMR integrating the peaks at 6.43 (**10b**) and 3.90 (**10**).

Rf (1:1 EtOAc:Pentane) = 0.5.

^{1}H NMR (400 MHz, $CDCl_3$) δ/ppm: 7.45 – 7.36 (m, 2H, Ar*H*), 7.30 – 7.26 (m, 3H, Ar*H*), 3.90 (dd, *J* = 11.0, 4.8 Hz, 1H, OCH_2), 3.85 – 3.77 (m, 1H, OCH_2), 3.71 – 3.37 (m, 2H, C*H*), 2.57 (d, *J* = 7.1 Hz, 2H, CH_2), 2.17 – 2.07 (m, 1H, CH_2), 2.07 – 1.88 (m, 4H, CH_2), 1.80 – 1.64 (m, 4H, CH_2).

^{13}C {^{1}H} NMR (101 MHz, $CDCl_3$) δ/ppm: 131.7, 128.7, 128.4, 127.9, 89.4, 81.8, 72.5, 64.2, 46.6, 42.6, 36.4, 28.3, 25.9, 25.0, 19.6.

HRMS (ESI/QTOF) m/z: $[M + Na]^+$ Calcd for $C_{17}H_{22}NaO_2^+$ 281.1512; Found 281.1518.

7. X-Ray crystallography data

Crystals were grown by preparing a solution of **5n** in CD_3Cl, leaving the solution slowly evaporate over 2-3 days at 4° C.

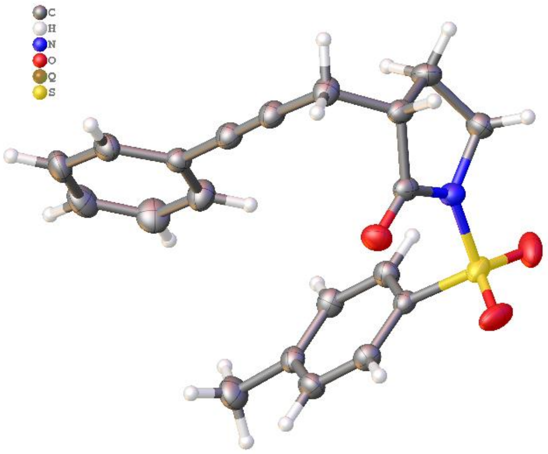

Figure S2: Ellipsoid plot (probability level 50%) of 5n.

Experimental. Single clear pale colourless prism-shaped crystals of **5n** were used as supplied. A suitable crystal with dimensions 0.75 × 0.39 × 0.36 mm^3 was selected and mounted on a SuperNova, Dual, Cu at home/near, AtlasS2 diffractometer. The crystal was kept at a steady T = 140.00(10) K during data collection. The structure was solved with the **ShelXT** (Sheldrick, 2015) solution program using dual methods and by using **Olex2** 1.5 (Dolomanov et al., 2009) as the graphical interface. The model was refined with **ShelXL** 2019/3 (Sheldrick, 2015) using full matrix least squares minimisation on $\boldsymbol{F^2}$.

Crystal Data. $C_{20}H_{19}NO_3S$, M_r = 353.42, monoclinic, $P2_1/c$ (No. 14), a = 8.0846(3) Å, b = 21.2355(7) Å, c = 10.2862(4) Å, β = 93.826(4)°, $\alpha = \gamma$ = 90°, V = 1762.01(11) Å^3, T = 140.00(10) K, Z = 4, Z' = 1, μ(Mo K_α) = 0.202, 19643 reflections measured, 5961 unique (R_{int} = 0.0277) which were used in all calculations. The final wR_2 was 0.1205 (all data) and R_1 was 0.0466 (I≥2 σ(I)).

**Compound**	**5n**
Formula	$C_{20}H_{19}NO_3S$
$D_{calc.}$/ g cm^{-3}	1.332
μ/mm^{-1}	0.202
Formula Weight	353.42
Colour	clear pale colourless
Shape	prism-shaped
Size/mm^3	0.75×0.39×0.36
T/K	140.00(10)
Crystal System	monoclinic
Space Group	$P2_1/c$
a/Å	8.0846(3)
b/Å	21.2355(7)
c/Å	10.2862(4)
α/°	90
β/°	93.826(4)
γ/°	90
V/Å^3	1762.01(11)
Z	4
Z'	1
Wavelength/Å	0.71073
Radiation type	Mo K_α
Θ_{min}/°	2.760
Θ_{max}/°	32.883
Measured Refl's.	19643
Indep't Refl's	5961
Refl's I≥2 σ(I)	4686
R_{int}	0.0277
Parameters	302
Restraints	0
Largest Peak	0.407
Deepest Hole	-0.379
GooF	1.045
wR_2 (all data)	0.1205
wR_2	0.1108
R_1 (all data)	0.0633
R_1	0.0466

Table 1: Fractional Atomic Coordinates (×10^4) and Equivalent Isotropic Displacement Parameters (Å^2×10^3) for cav-05-216_mo. U_{eq} is defined as 1/3 of the trace of the orthogonalised U_{ij}.

**Atom**	**x**	**y**	**z**	U_{eq}
S1	3253.1(4)	5733.6(2)	2070.6(4)	28.92(10)
O1	4854.3(13)	5820.5(5)	4832.1(10)	33.4(2)
O2	2081.7(13)	5472.9(5)	2897.8(13)	41.6(3)
O3	3233.2(15)	5542.4(5)	738.5(11)	42.7(3)
N1	5145.4(14)	5558.3(5)	2683.1(11)	26.0(2)
C1	5670.1(16)	5614.2(6)	3993.2(13)	24.9(2)
C2	7437.4(16)	5365.5(6)	4138.5(13)	25.9(3)
C3	7998.5(18)	5390.6(7)	2756.4(14)	31.9(3)
C4	6422.4(18)	5288.5(7)	1884.2(15)	31.1(3)
C5	8506.7(19)	5732.3(7)	5159.4(15)	31.8(3)
C6	8430.6(17)	6414.2(6)	4946.5(14)	29.8(3)
C7	8297.6(17)	6966.6(6)	4767.6(13)	28.9(3)
C8	8155.1(16)	7630.3(6)	4500.0(13)	25.8(3)
C9	7171(2)	7839.3(7)	3427.9(15)	35.0(3)
C10	7096(2)	8475.6(8)	3125.8(17)	41.5(4)
C11	8008(2)	8905.0(7)	3878.3(17)	39.5(4)

Atom	x	y	z	U_{eq}
C12	8989(2)	8700.8(7)	4951.0(15)	36.9(3)
C13	9045.1(18)	8070.0(7)	5279.3(14)	30.7(3)
C14	3121.5(15)	6556.5(6)	2136.0(12)	22.8(2)
C15	2228.8(17)	6835.9(6)	3082.3(13)	27.4(3)
C16	2049.5(18)	7484.4(7)	3083.2(14)	29.4(3)
C17	2757.2(17)	7857.9(6)	2155.4(13)	26.5(3)
C18	3681.6(18)	7566.9(6)	1230.8(13)	27.9(3)
C19	3862.5(17)	6918.3(6)	1206.4(13)	27.0(3)
C20	2493(3)	8557.6(7)	2164(2)	41.0(4)

Table 2: Anisotropic Displacement Parameters (×10[4]) for cav-05-216_mo. The anisotropic displacement factor exponent takes the form: $-2\pi^2[h^2a^{*2} \times U_{11} + ... +2hka^* \times b^* \times U_{12}]$

Atom	U_{11}	U_{22}	U_{33}	U_{23}	U_{13}	U_{12}
S1	23.08(16)	21.35(16)	41.2(2)	0.23(12)	-6.34(13)	-1.51(11)
O1	30.5(5)	35.6(5)	35.1(5)	0.6(4)	8.7(4)	1.9(4)
O2	22.6(5)	29.2(5)	72.8(8)	12.8(5)	0.3(5)	-5.1(4)
O3	46.7(7)	32.5(6)	45.9(6)	-12.5(5)	-18.4(5)	4.6(5)
N1	21.8(5)	25.6(5)	30.5(5)	0.4(4)	-0.1(4)	3.0(4)
C1	22.3(6)	21.2(6)	31.4(6)	4.2(5)	2.9(5)	-0.7(4)
C2	22.9(6)	22.1(6)	32.6(6)	4.5(5)	1.1(5)	1.1(4)
C3	23.8(6)	36.8(8)	35.6(7)	1.0(6)	6.2(5)	3.0(5)
C4	29.7(7)	31.6(7)	32.1(7)	-3.5(5)	3.5(5)	3.4(5)
C5	32.4(7)	24.9(7)	36.9(7)	4.6(5)	-7.8(6)	1.4(5)
C6	27.1(6)	28.3(7)	33.1(7)	2.8(5)	-3.9(5)	-0.6(5)
C7	26.4(6)	29.5(7)	30.2(6)	1.4(5)	-1.7(5)	-0.2(5)
C8	24.7(6)	24.6(6)	28.1(6)	1.5(5)	2.3(5)	1.3(5)
C9	36.6(8)	31.5(7)	35.5(7)	1.6(6)	-7.0(6)	-0.8(6)
C10	48.3(9)	35.8(8)	39.2(8)	10.0(6)	-5.6(7)	6.5(7)
C11	51.3(10)	24.1(7)	43.9(8)	3.4(6)	9.9(7)	6.3(6)
C12	44.8(9)	27.8(7)	38.2(8)	-10.3(6)	3.7(6)	0.9(6)
C13	32.8(7)	30.3(7)	28.5(7)	-4.1(5)	-2.0(5)	3.7(5)
C14	20.7(5)	21.6(6)	25.4(6)	1.7(4)	-3.0(4)	-0.6(4)
C15	27.6(6)	29.0(7)	25.8(6)	3.6(5)	3.3(5)	-1.9(5)
C16	29.3(7)	31.3(7)	28.0(6)	-3.5(5)	4.0(5)	1.8(5)
C17	26.3(6)	24.0(6)	28.3(6)	0.4(5)	-5.1(5)	0.6(5)
C18	31.7(7)	27.8(7)	24.0(6)	5.0(5)	0.7(5)	-3.2(5)
C19	28.7(6)	28.4(7)	24.1(6)	-0.6(5)	3.8(5)	-0.5(5)
C20	46.8(10)	24.3(7)	51.5(10)	1.1(6)	-0.8(8)	4.0(6)

Table 3: Bond Lengths in Å for cav-05-216_mo.

Atom	Atom	Length/Å
S1	O2	1.4264(12)
S1	O3	1.4282(12)
S1	N1	1.6575(11)
S1	C14	1.7524(13)
O1	C1	1.2031(17)
N1	C1	1.3906(17)
N1	C4	1.4773(18)
C1	C2	1.5213(18)
C2	C3	1.522(2)
C2	C5	1.5283(19)
C3	C4	1.524(2)
C5	C6	1.4651(19)

Atom	Atom	Length/Å
C6	C7	1.1912(19)
C7	C8	1.4393(18)
C8	C9	1.3888(19)
C8	C13	1.3982(19)
C9	C10	1.387(2)
C10	C11	1.378(2)
C11	C12	1.385(2)
C12	C13	1.381(2)
C14	C15	1.3836(18)
C14	C19	1.3929(18)
C15	C16	1.385(2)
C16	C17	1.393(2)

Atom	Atom	Length/Å
C17	C18	1.393(2)
C17	C20	1.501(2)
C18	C19	1.385(2)

Table 4: Bond Angles in ° for cav-05-216_mo.

Atom	Atom	Atom	Angle/°
O2	S1	O3	119.77(7)
O2	S1	N1	108.59(6)
O2	S1	C14	108.64(7)
O3	S1	N1	104.59(7)
O3	S1	C14	108.87(6)
N1	S1	C14	105.44(6)
C1	N1	S1	124.28(9)
C1	N1	C4	113.13(11)
C4	N1	S1	122.49(9)
O1	C1	N1	125.43(12)
O1	C1	C2	127.75(12)
N1	C1	C2	106.82(11)
C1	C2	C3	103.46(11)
C1	C2	C5	112.03(11)
C3	C2	C5	115.71(12)
C2	C3	C4	104.79(11)
N1	C4	C3	101.85(11)
C6	C5	C2	112.63(11)
C7	C6	C5	177.23(15)
C6	C7	C8	177.75(15)
C9	C8	C7	119.94(12)
C9	C8	C13	119.16(13)
C13	C8	C7	120.85(12)
C10	C9	C8	120.24(14)
C11	C10	C9	120.32(15)
C10	C11	C12	119.81(14)
C13	C12	C11	120.40(14)
C12	C13	C8	120.02(13)
C15	C14	S1	119.38(10)
C15	C14	C19	120.99(12)
C19	C14	S1	119.59(10)
C14	C15	C16	119.11(12)
C15	C16	C17	121.20(13)
C16	C17	C20	119.63(14)
C18	C17	C16	118.60(12)
C18	C17	C20	121.77(14)
C19	C18	C17	121.04(13)
C18	C19	C14	119.03(12)

Table 5: Torsion Angles in ° for cav-05-216_mo.

Atom	Atom	Atom	Atom	Angle/°
S1	N1	C1	O1	-3.46(19)
S1	N1	C1	C2	176.40(9)
S1	N1	C4	C3	164.57(10)
S1	C14	C15	C16	-176.33(10)
S1	C14	C19	C18	176.91(10)
O1	C1	C2	C3	-160.91(14)
O1	C1	C2	C5	-35.60(19)
O2	S1	N1	C1	-45.10(12)
O2	S1	N1	C4	131.19(11)
O2	S1	C14	C15	11.78(12)
O2	S1	C14	C19	-165.80(10)
O3	S1	N1	C1	-174.07(11)
O3	S1	N1	C4	2.22(13)
O3	S1	C14	C15	143.78(11)
O3	S1	C14	C19	-33.80(12)
N1	S1	C14	C15	-104.47(11)
N1	S1	C14	C19	77.95(11)
N1	C1	C2	C3	19.24(13)
N1	C1	C2	C5	144.55(11)
C1	N1	C4	C3	-18.77(15)
C1	C2	C3	C4	-30.44(14)
C1	C2	C5	C6	-51.56(17)
C2	C3	C4	N1	29.77(14)
C3	C2	C5	C6	66.70(17)
C4	N1	C1	O1	179.94(13)
C4	N1	C1	C2	-0.20(15)
C5	C2	C3	C4	-153.35(12)

Atom	Atom	Atom	Atom	Angle/°
C7	C8	C9	C10	-176.67(15)
C7	C8	C13	C12	175.16(14)
C8	C9	C10	C11	0.7(3)
C9	C8	C13	C12	-2.5(2)
C9	C10	C11	C12	-0.8(3)
C10	C11	C12	C13	-0.7(3)
C11	C12	C13	C8	2.3(2)
C13	C8	C9	C10	1.0(2)
C14	S1	N1	C1	71.18(12)
C14	S1	N1	C4	-112.53(11)
C14	C15	C16	C17	-0.3(2)
C15	C14	C19	C18	-0.63(19)
C15	C16	C17	C18	-1.1(2)
C15	C16	C17	C20	178.18(14)
C16	C17	C18	C19	1.7(2)
C17	C18	C19	C14	-0.8(2)
C19	C14	C15	C16	1.21(19)
C20	C17	C18	C19	-177.57(14)

Table 6: Hydrogen Fractional Atomic Coordinates (×10^4) and Equivalent Isotropic Displacement Parameters (Å^2×10^3) for cav-05-216_mo. U_{eq} is defined as 1/3 of the trace of the orthogonalised U_{ij}.

Atom	x	y	z	U_{eq}
H3	7330(20)	4927(8)	4436(17)	40(5)
H5	8850(20)	5073(8)	2590(17)	38(5)
H13	8400(20)	5819(9)	2614(18)	41(5)
H1	6420(30)	5507(9)	1080(20)	48(5)
H15	6170(20)	4843(9)	1683(17)	40(5)
H14	9690(20)	5594(8)	5169(17)	38(5)
H20	8120(20)	5652(8)	6018(19)	44(5)
H17	6570(20)	7542(9)	2904(18)	43(5)
H6	6400(30)	8613(10)	2400(20)	61(6)
H4	8020(30)	9350(9)	3660(20)	55(6)
H7	9640(20)	8991(9)	5508(19)	46(5)
H16	9690(20)	7948(8)	6022(17)	37(5)
H11	1730(20)	6583(8)	3742(17)	40(5)
H19	1400(20)	7681(8)	3741(16)	34(4)
H2	4210(20)	7825(8)	597(18)	41(5)
H18	4510(20)	6717(8)	545(18)	42(5)
H8	2820(30)	8720(12)	3030(30)	82(8)
H9	1330(40)	8668(12)	2070(30)	89(8)
H12	3050(30)	8745(12)	1560(20)	73(7)

8. References

1) Amos, S. G. E.; Cavalli, D.; Le Vaillant, F.; Waser, J. Direct Photoexcitation of Ethynylbenziodoxolones: An Alternative to Photocatalysis for Alkynylation Reactions. *Angew. Chem. Int. Ed.* **2021**, *60* (44), 23827–23834.
2) Liu, B.; Lim, C.-H.; Miyake, G. M. Light-Driven Intermolecular Charge Transfer Induced Reactivity of Ethynylbenziodoxol(on)e and Phenols. *J. Am. Chem. Soc.* **2018**, *140* (40), 12829–12835.
3) Weires, N. A.; Slutskyy, Y.; Overman, L. E. Facile Preparation of Spirolactones by an Alkoxycarbonyl Radical Cyclization–Cross-coupling Cascade. *Angew. Chem. Int. Ed*. **2019**, *58* (25), 8561–8565.
4) Freerksen, R. W.; Pabst, W. E.; Raggio, M. L.; Sherman, S. A.; Wroble, R. R.; Watt, D. S. Photolysis of α-Peracetoxynitriles. 2. A Comparison of Two Synthetic Approaches to 18-Cyano-20-Ketosteroids. *J. Am. Chem. Soc*. **1977**, *99* (5), 1536–1542.
5) Wang, T.; Hao, X.-Q.; Huang, J.-J.; Niu, J.-L.; Gong, J.-F.; Song, M.-P. Chiral Bis(Imidazolinyl)Phenyl NCN Pincer Rhodium(III) Catalysts for Enantioselective Allylation of Aldehydes and Carbonyl–Ene Reaction of Trifluoropyruvates*. J. Org. Chem.* **2013**, *78* (17), 8712–8721.
6) Tobia, D.; Rickborn, B. Kinetics and Stereochemistry of LiNR2-Induced 1,2-Elimination of Homoallylic Ethers. *J. Org. Chem.* **1989**, *54* (4), 777–782.
7) Launay, G. G.; Slawin, A. M. Z.; O'Hagan, D. Prins Fluorination Cyclisations: Preparation of 4-Fluoro-Pyran and -Piperidine Heterocycles. *Beilstein J. Org. Chem.* **2010**, *6*. https://doi.org/10.3762/bjoc.6.41.
8) Schelwies, M.; Paciello, R.; Pelzer, R.; Siegel, W.; Breuer, M. Palladium-catalyzed Low Pressure Carbonylation of Allylic Alcohols by Catalytic Anhydride Activation. *Chemistry* **2021**, *27* (36), 9263–9266.
9) Fanourakis, A.; Hodson, N. J.; Lit, A. R.; Phipps, R. J. Substrate-Directed Enantioselective Aziridination of Alkenyl Alcohols Controlled by a Chiral Cation*. J. Am. Chem. Soc*. **2023**, *145* (13), 7516–7527.
10) Lucas, E. L.; Hewitt, K. A.; Chen, P.-P.; Castro, A. J.; Hong, X.; Jarvo, E. R. Engaging Sulfonamides: Intramolecular Cross-Electrophile Coupling Reaction of Sulfonamides with Alkyl Chlorides. *J. Org. Chem.* **2020**, *85* (4), 1775–1793.
11) Wang, J.-W.; Liu, D.-G.; Chang, Z.; Li, Z.; Fu, Y.; Lu, X. Nickel-catalyzed Switchable Site-selective Alkene Hydroalkylation by Temperature Regulation. *Angew. Chem. Int. Ed*. **2022**, *61* (31), e202205537
12) Asensio, G.; Mello, R.; Boix-Bernardini, C.; Gonzalez-Nunez, M. E.; Castellano, G. Epoxidation of Primary and Secondary Alkenylammonium Salts with Dimethyldioxirane, Methyl(Trifluoromethyl)Dioxirane, and m-Chloroperbenzoic Acid. A General Synthetic Route to Epoxyalkylamines. *J. Org. Chem*. **1995**, *60* (12), 3692–3699
13) Jones, A. D.; Knight, D. W.; Hibbs, D. E. A Stereochemically Flexible Approach to Pyrrolidines Based on 5-Endo-Trig Iodocyclisations of Homoallylic Sulfonamides. *J Chem Soc Perkin Trans 1* **2001**, No. 10, 1182–1203.
14) Nguyen, T. M.; Nicewicz, D. A. Anti-Markovnikov Hydroamination of Alkenes Catalyzed by an Organic Photoredox System. *J. Am. Chem. Soc*. **2013**, *135* (26), 9588–9591
15) Su, J. Y.; Grünenfelder, D. C.; Takeuchi, K.; Reisman, S. E. Radical Deoxychlorination of Cesium Oxalates for the Synthesis of Alkyl Chlorides. *Org. Lett.* **2018**, *20* (16), 4912–4916.
16) Yang, D.; Chen, F.; Dong, Z.-M.; Zhang, D.-W. Ruthenium-Catalyzed Oxidative Cleavage of Alkynes to Carboxylic Acids*. J. Org. Chem*. **2004**, *69* (6), 2221–2223.
17) Gérardy, R.; Winter, M.; Horn, C. R.; Vizza, A.; Van Hecke, K.; Monbaliu, J.-C. M. Continuous-Flow Preparation of γ-Butyrolactone Scaffolds from Renewable Fumaric and Itaconic Acids under Photosensitized Conditions. *Org. Process Res. Dev.* **2017**, *21* (12), 2012–2017.

9. NMR Spectra of New Compounds

^{1}H NMR Spectrum (400 MHz, $CDCl_3$) of **S7g**

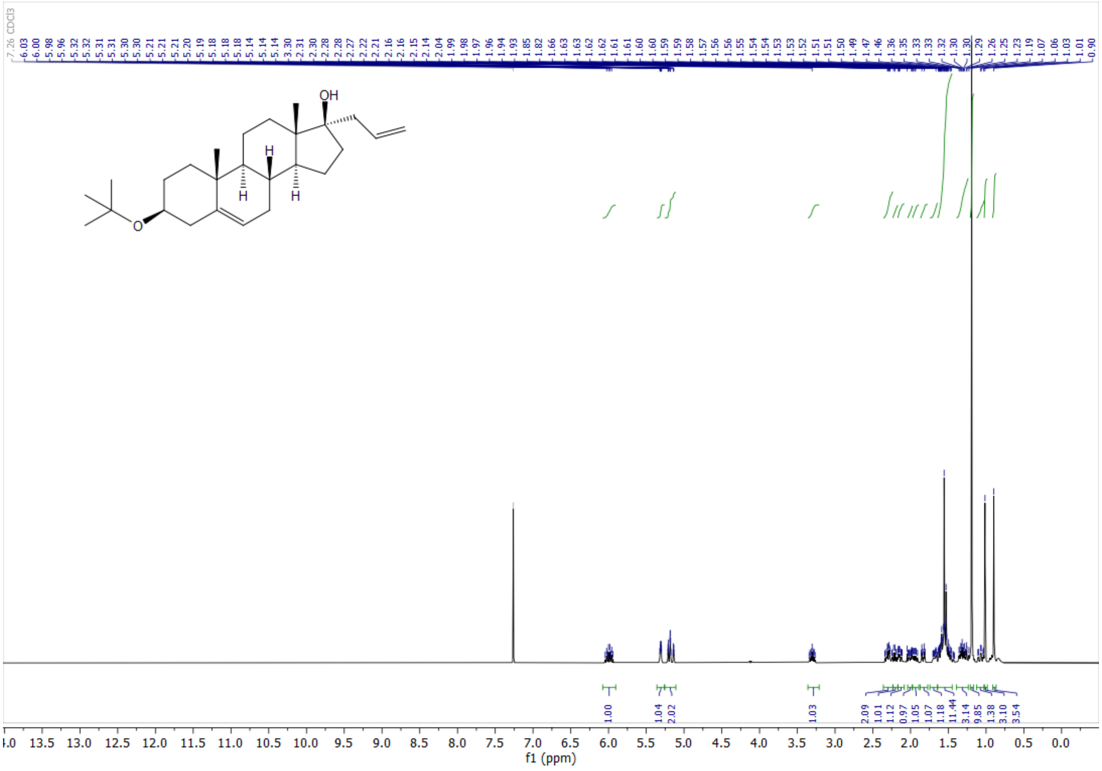

^{13}C {^{1}H} NMR Spectrum (101 MHz, $CDCl_3$) of **S7g**

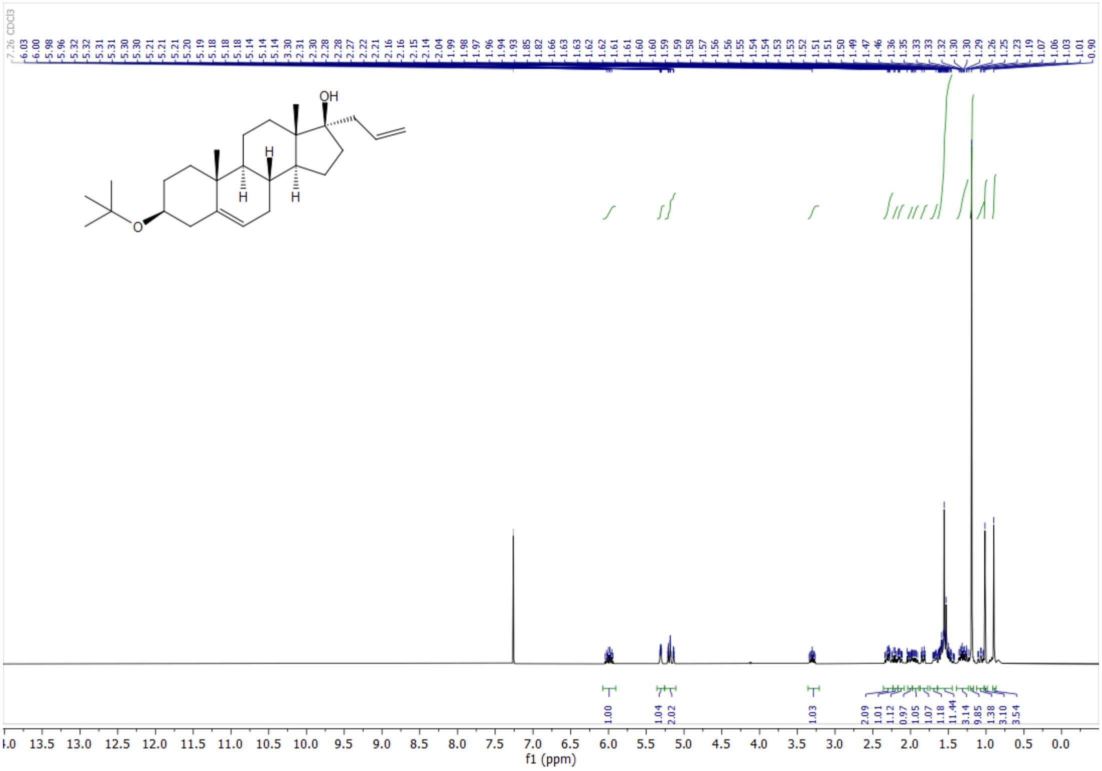

^{1}H NMR Spectrum (400 MHz, $CDCl_3$) of **S7q**

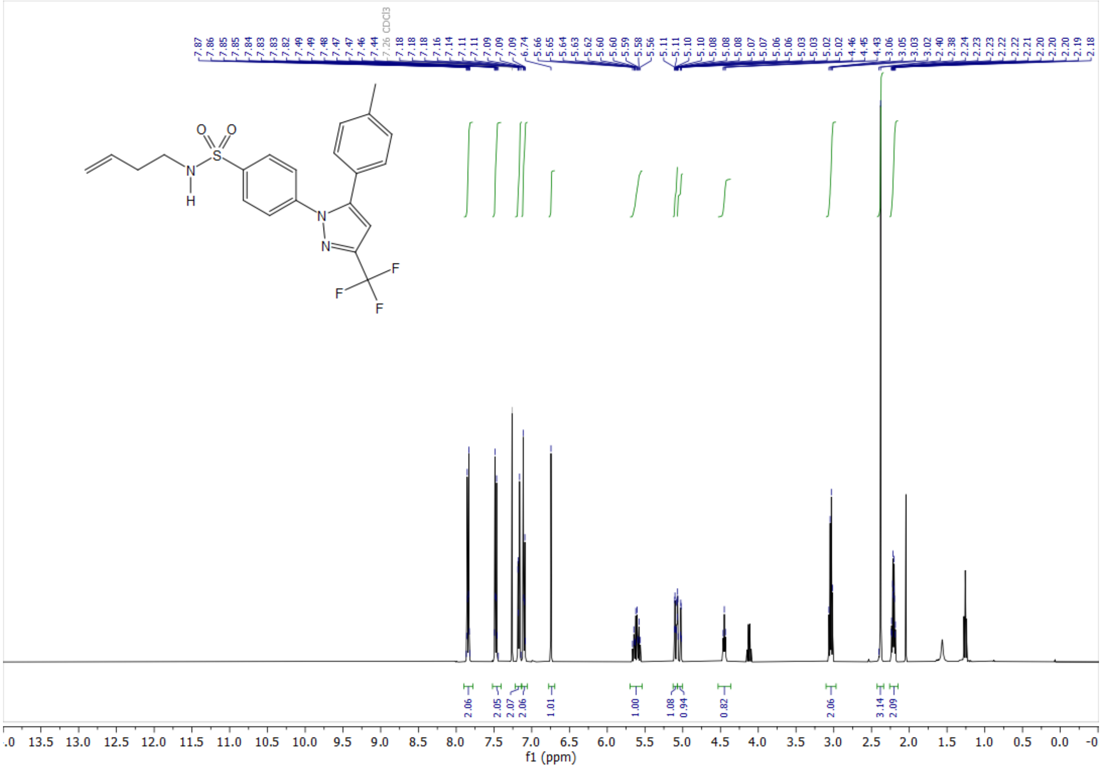

^{13}C {^{1}H} NMR Spectrum (101 MHz, $CDCl_3$) of **S7q**

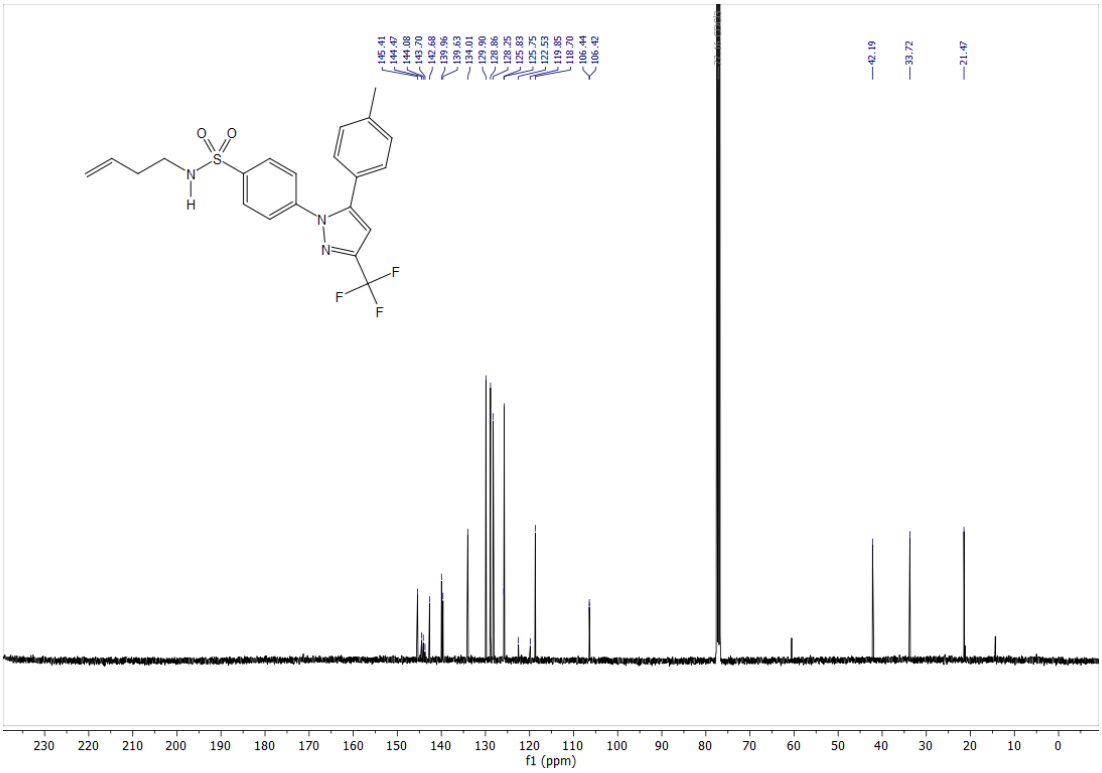

^{19}F NMR Spectrum (376 MHz, $CDCl_3$) of **S7q**

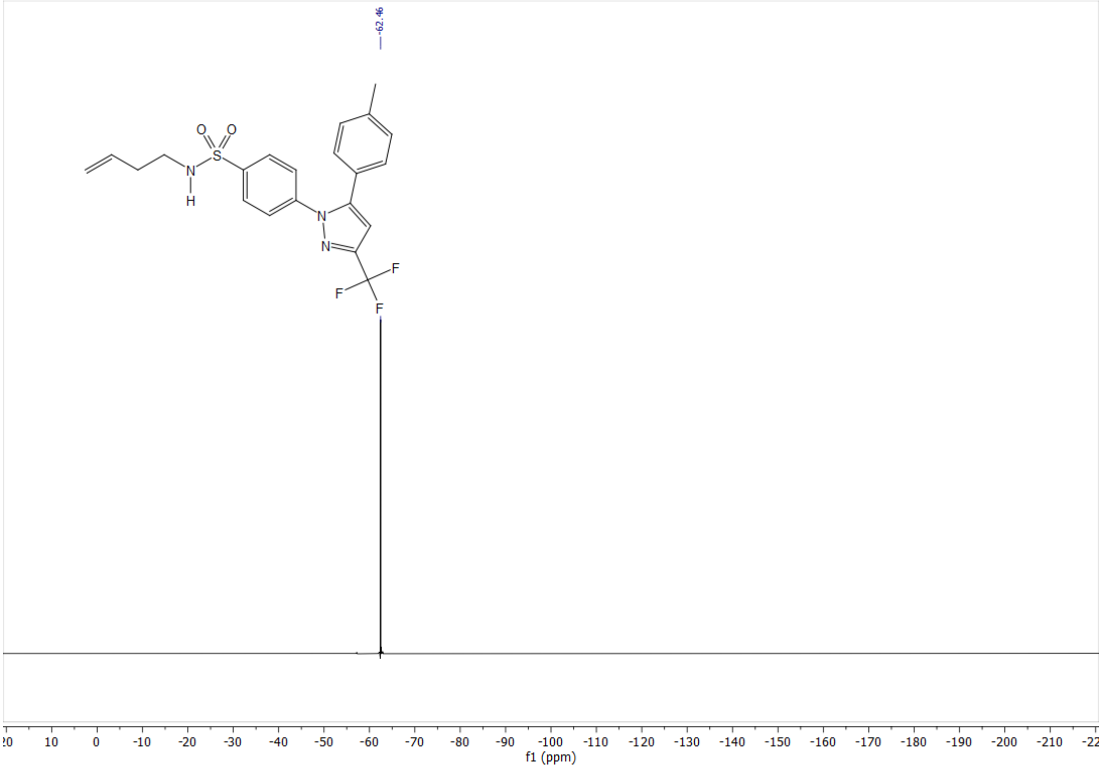

^{1}H NMR Spectrum (400 MHz, $CDCl_3$) of **S17a**

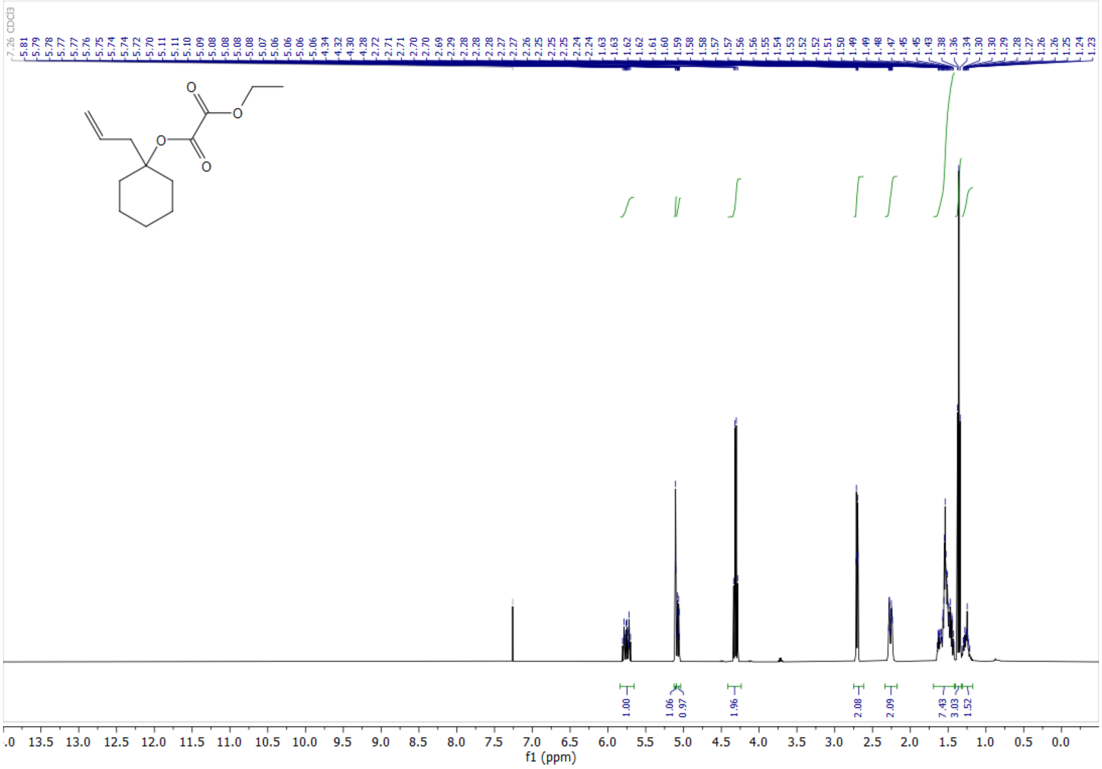

^{13}C {^{1}H} NMR Spectrum (101 MHz, $CDCl_3$) of **S17a**

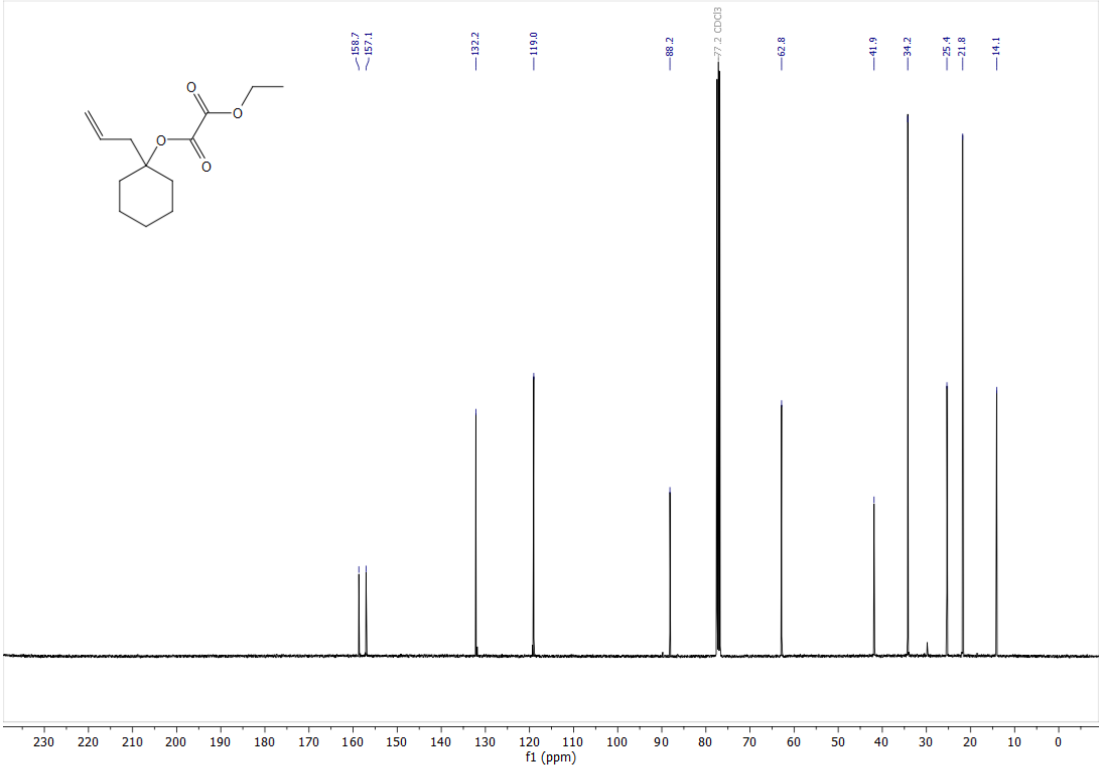

^{1}H NMR Spectrum (400 MHz, $CDCl_3$) of **S17b**

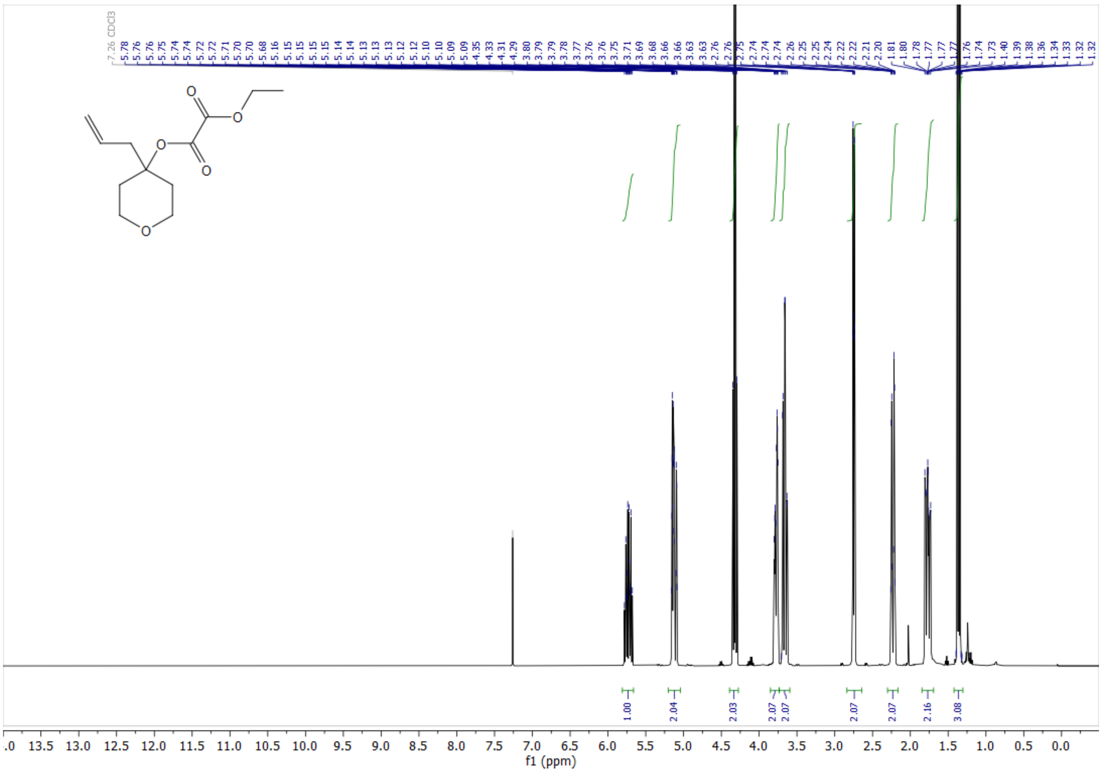

^{13}C {^{1}H} NMR Spectrum (101 MHz, $CDCl_3$) of **S17b**

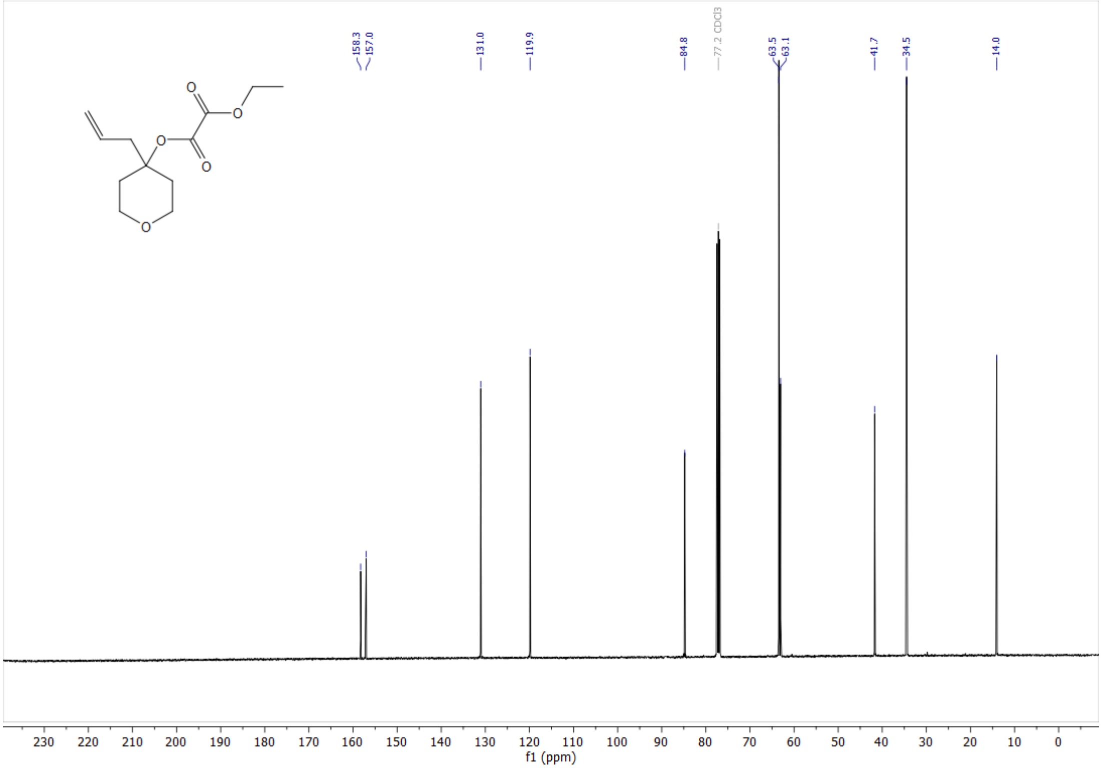

^{1}H NMR Spectrum (400 MHz, $CDCl_3$) of **S17c**

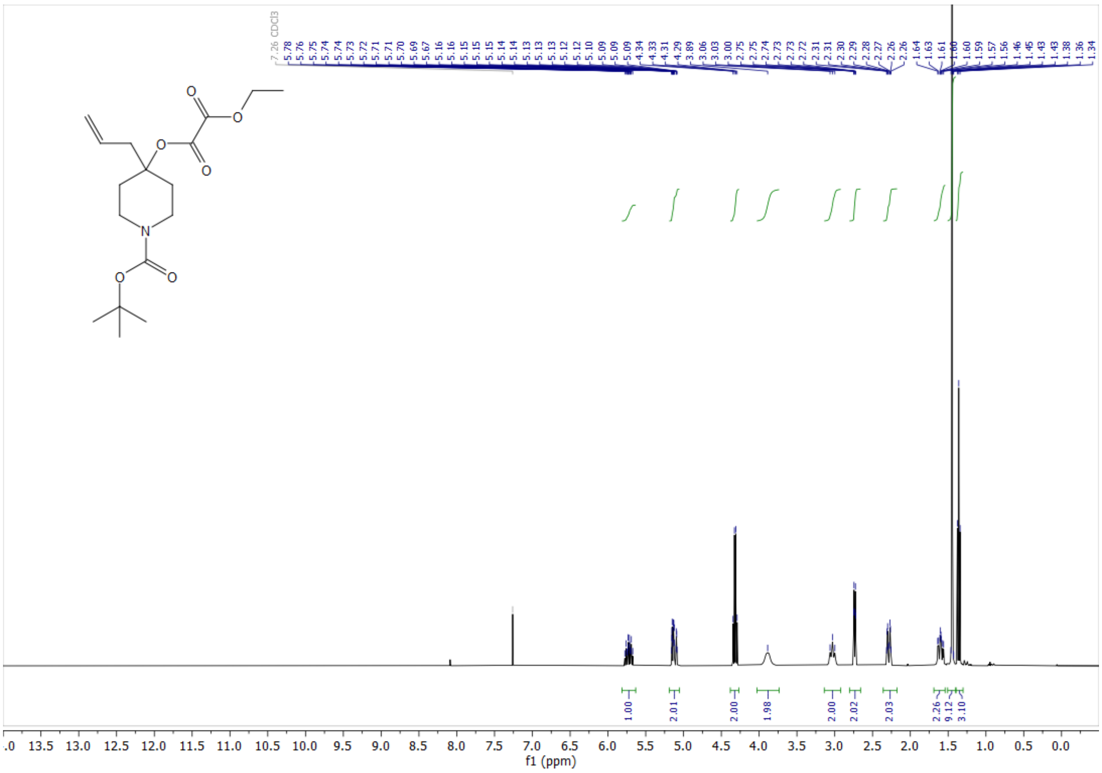

^{13}C {^{1}H} NMR Spectrum (101 MHz, $CDCl_3$) of **S17c**

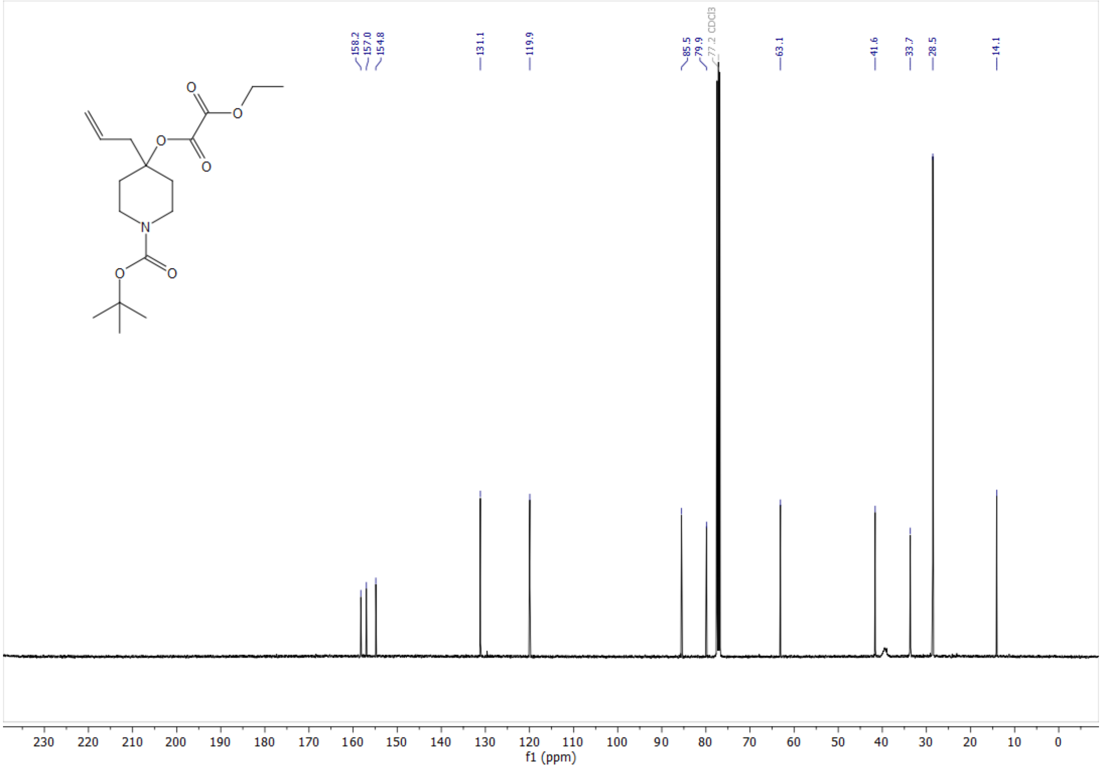

^{1}H NMR Spectrum (400 MHz, $CDCl_3$) of **S17d**

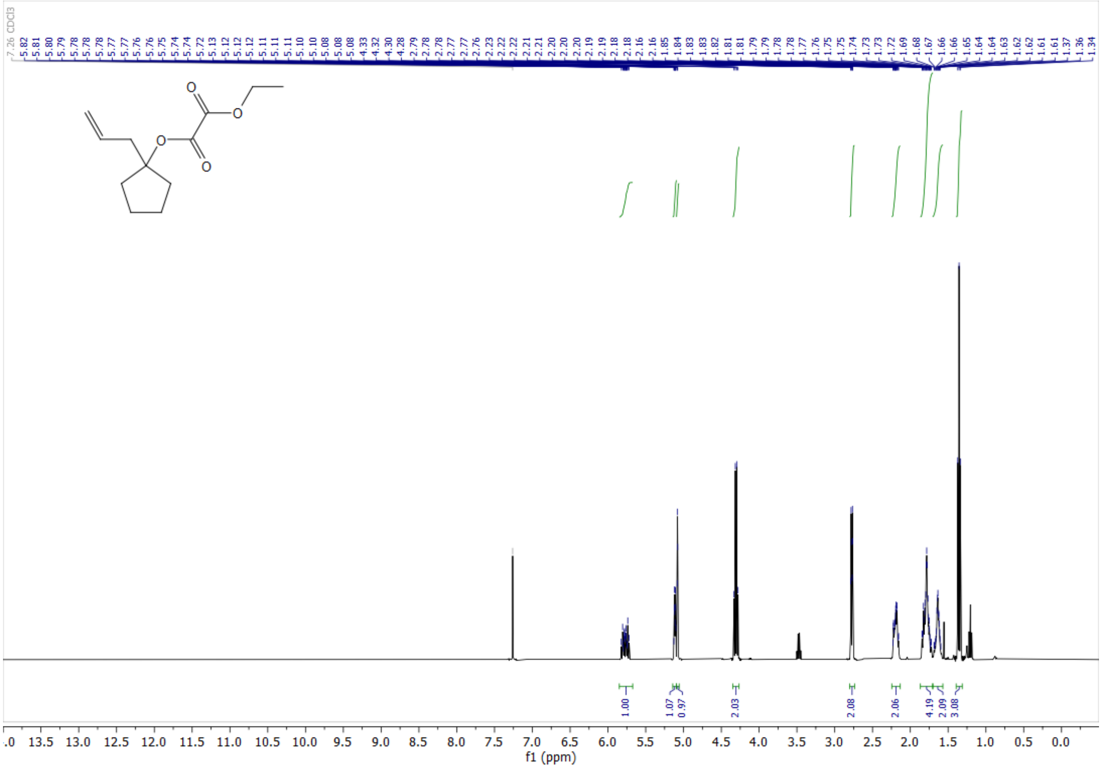

^{13}C {^{1}H} NMR Spectrum (101 MHz, $CDCl_3$) of **S17d**

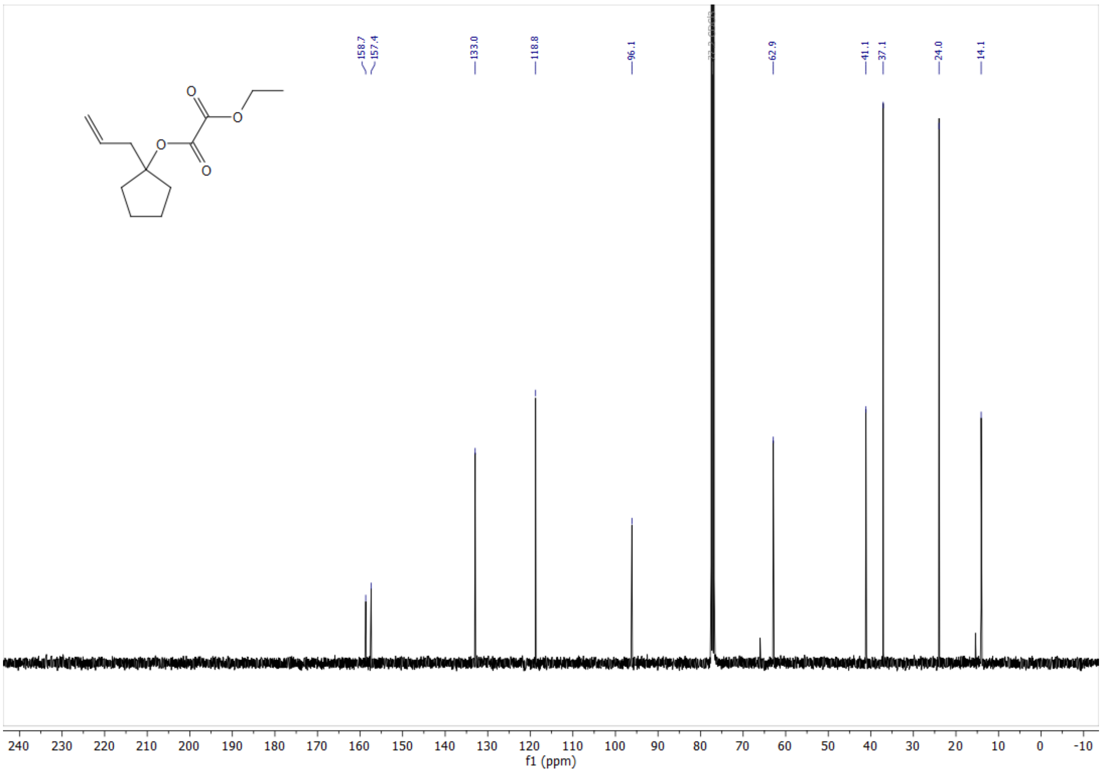

^{1}H NMR Spectrum (400 MHz, $CDCl_3$) of **S17e**

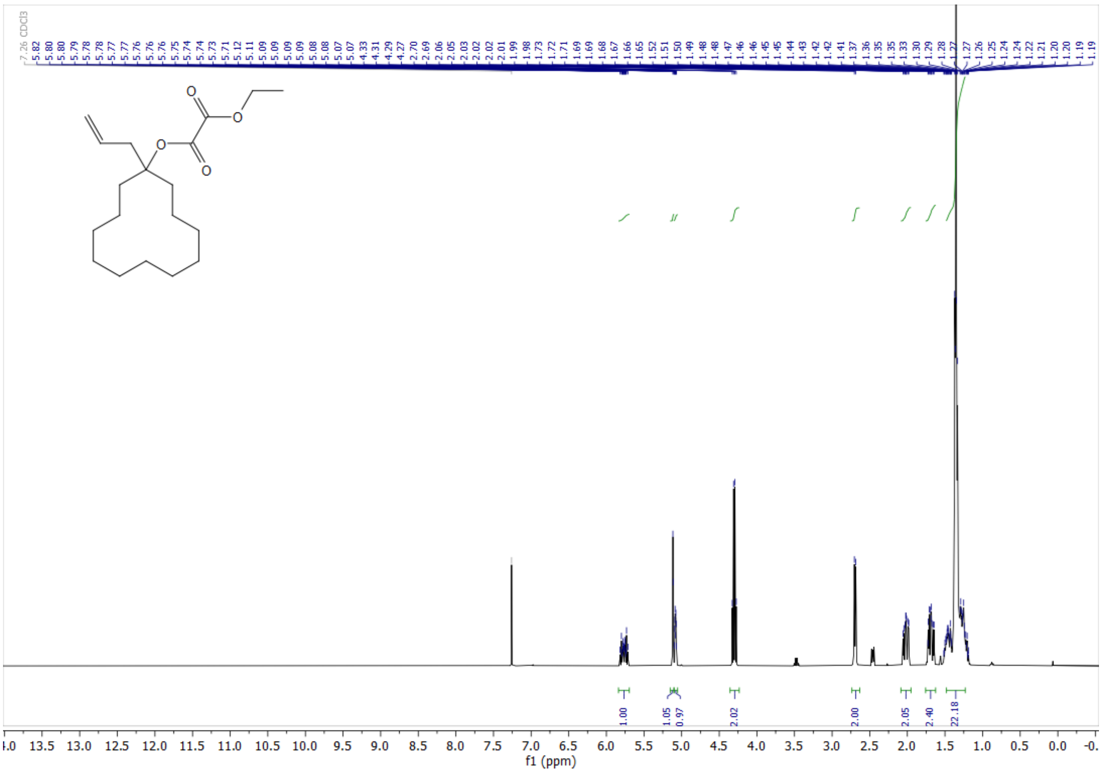

^{13}C {^{1}H} NMR Spectrum (101 MHz, $CDCl_3$) of **S17e**

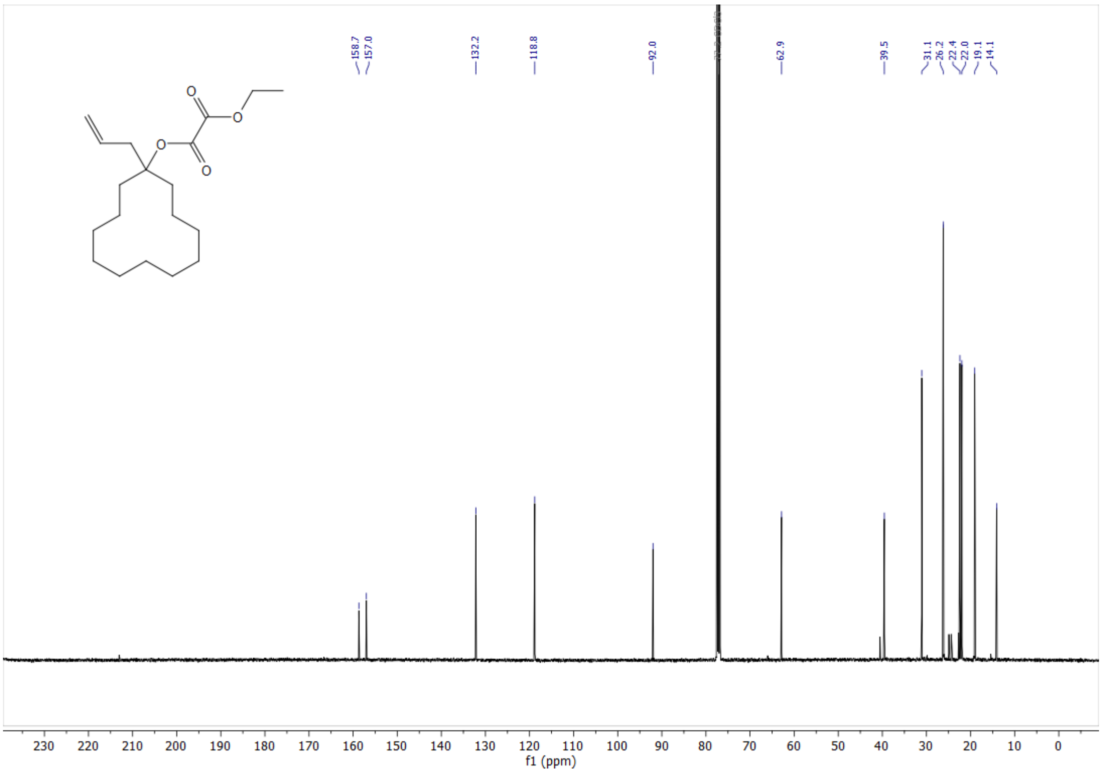

^{1}H NMR Spectrum (400 MHz, $CDCl_3$) of **S17f**

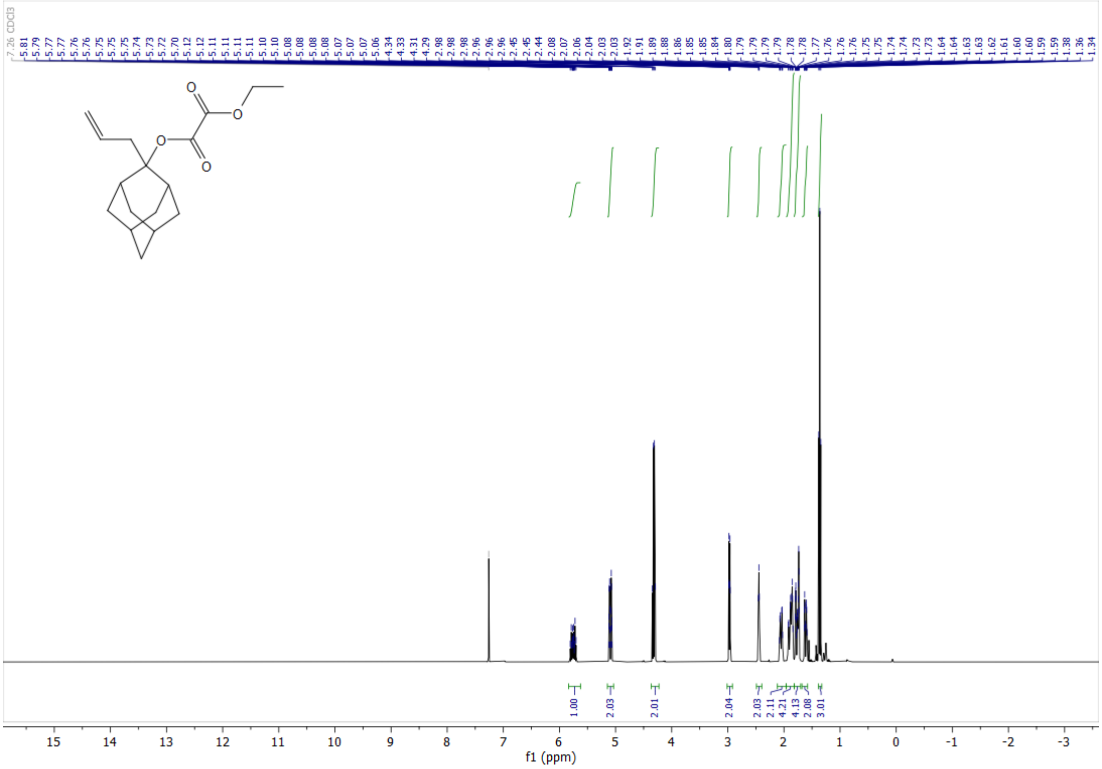

[13] C {[1] H} NMR Spectrum (101 MHz, $CDCl_3$) of **S17f**

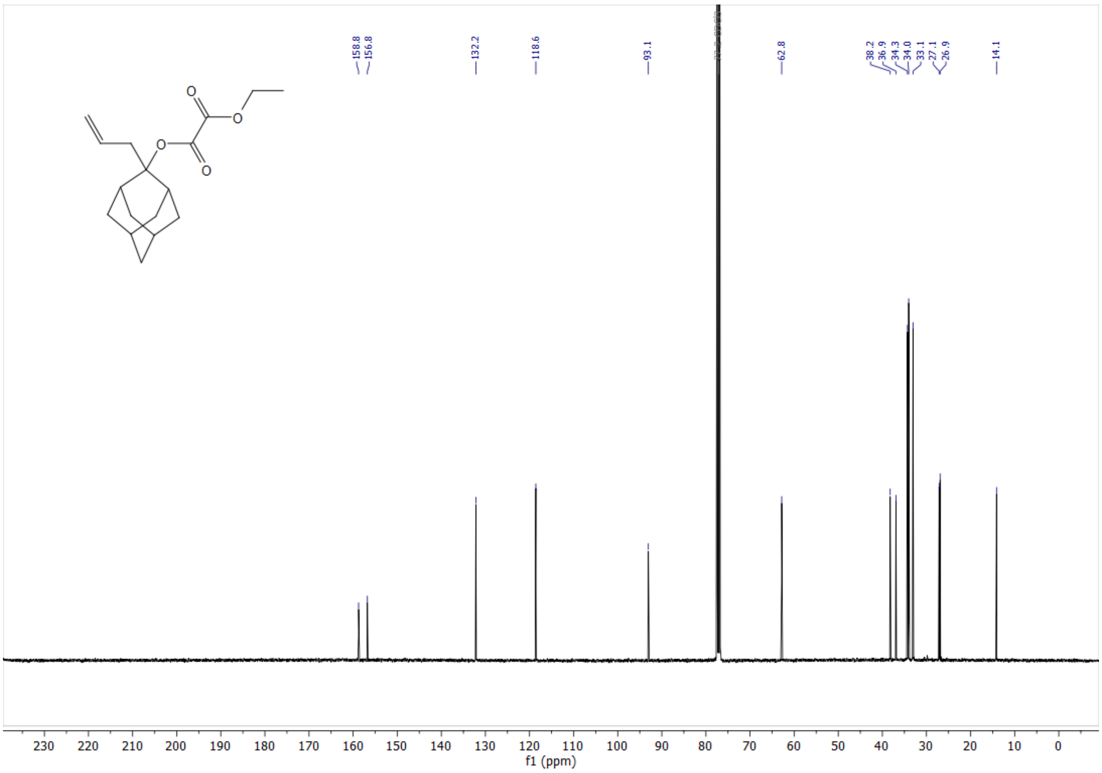

[1] H NMR Spectrum (400 MHz, $CDCl_3$) of **S17g**

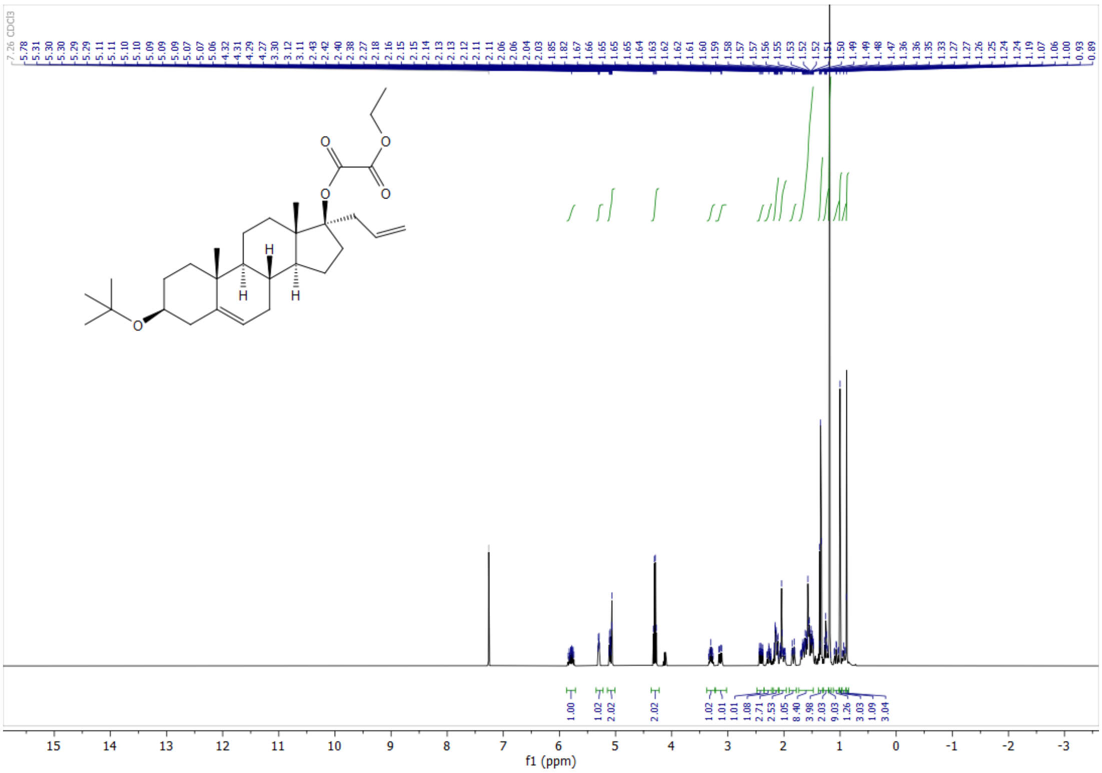

^{13}C {^{1}H} NMR Spectrum (101 MHz, $CDCl_3$) of **S17g**

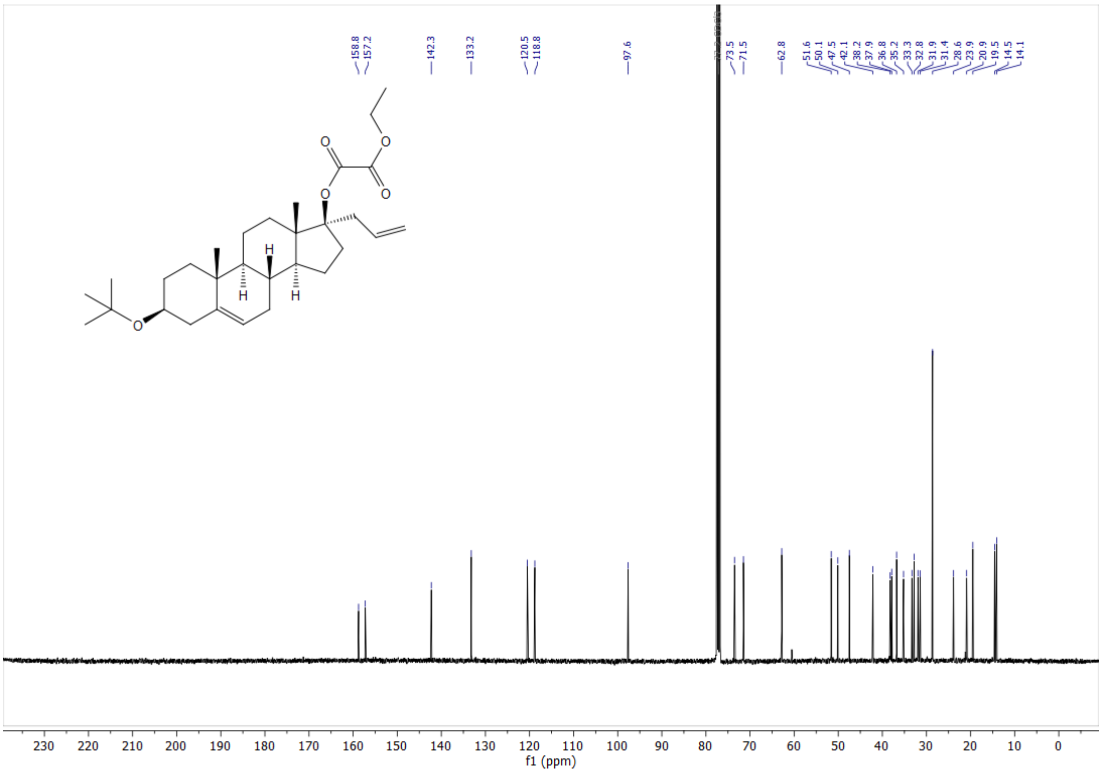

^{1}H NMR Spectrum (400 MHz, DMSO-d_6) of **2g**

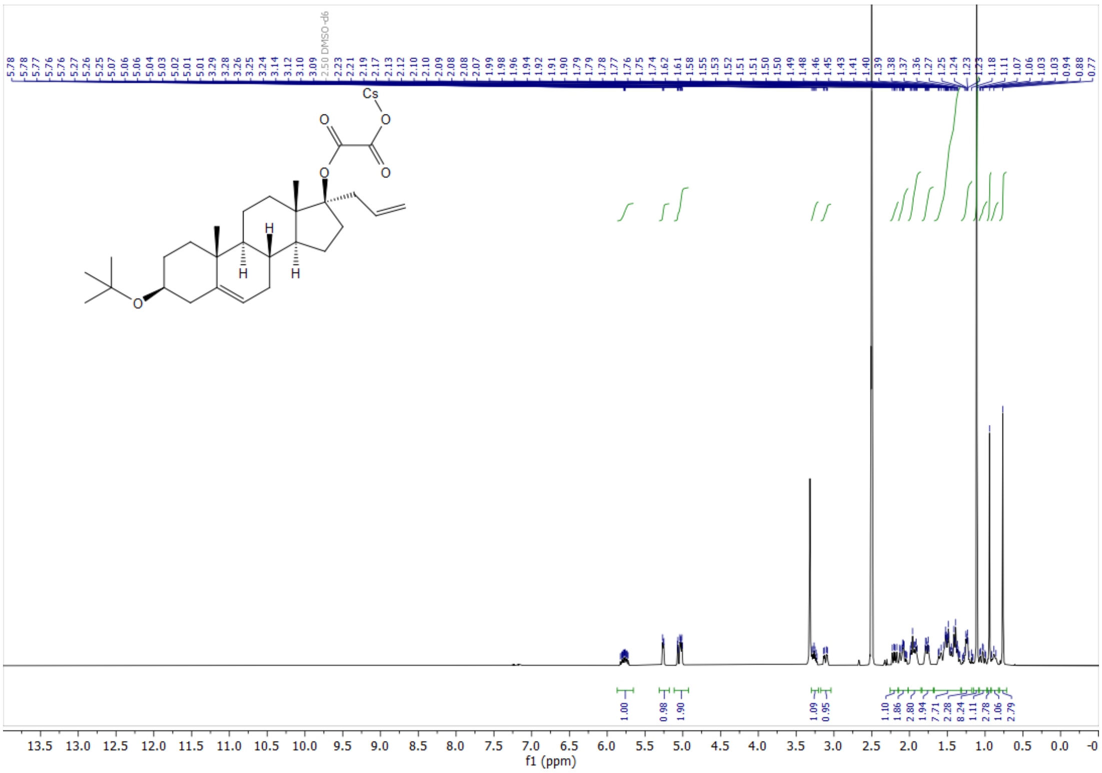

^{13}C {^{1}H} NMR Spectrum (101 MHz, DMSO-*d*$_6$) of **2g**

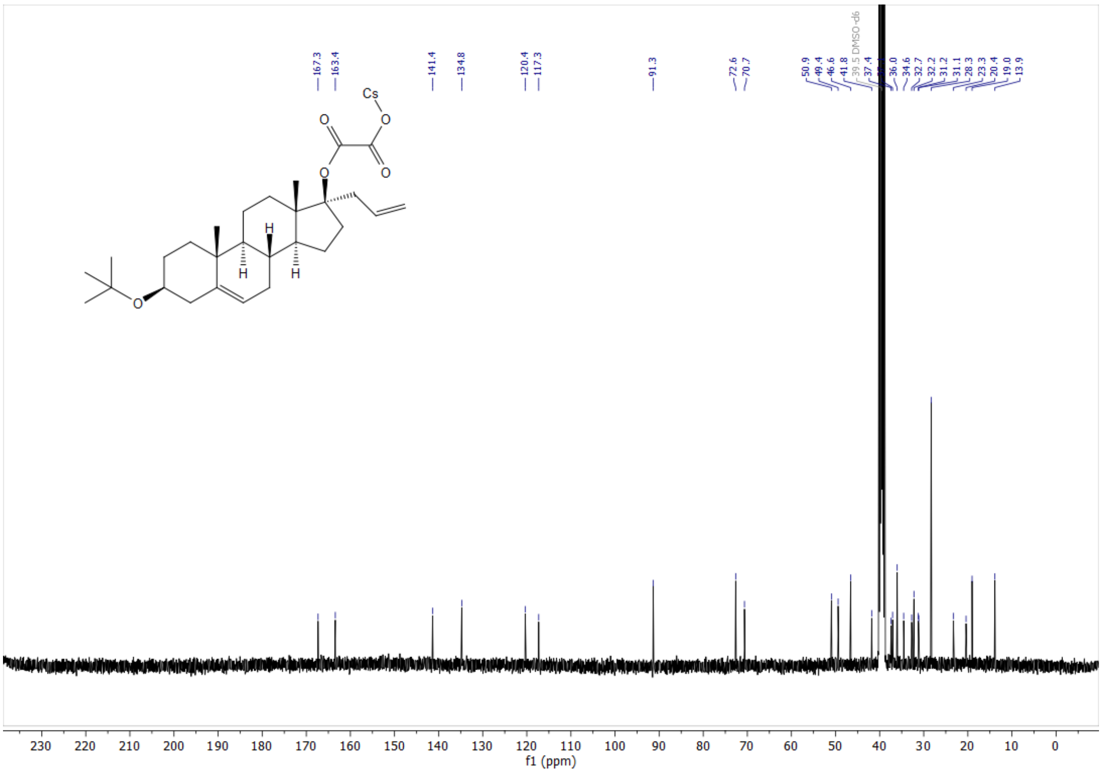

^{1}H NMR Spectrum (400 MHz, DMSO-*d*$_6$) of **2h**

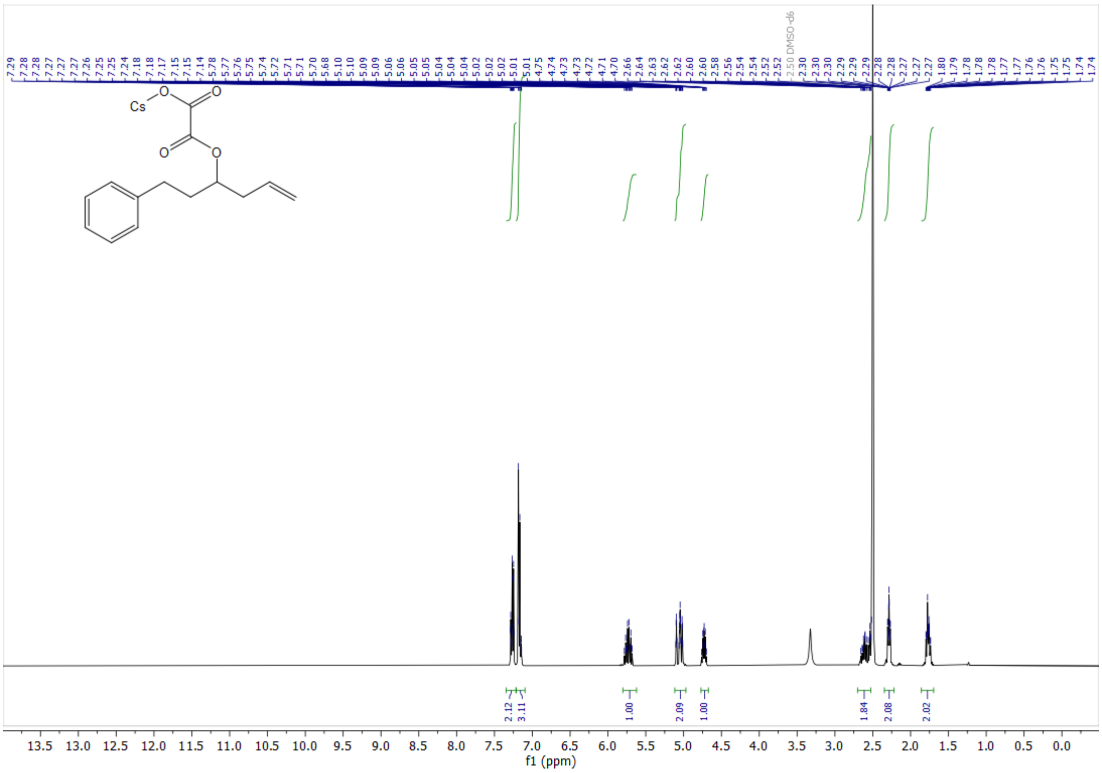

^{13}C {^{1}H} NMR Spectrum (101 MHz, DMSO-d_6) of **2h**

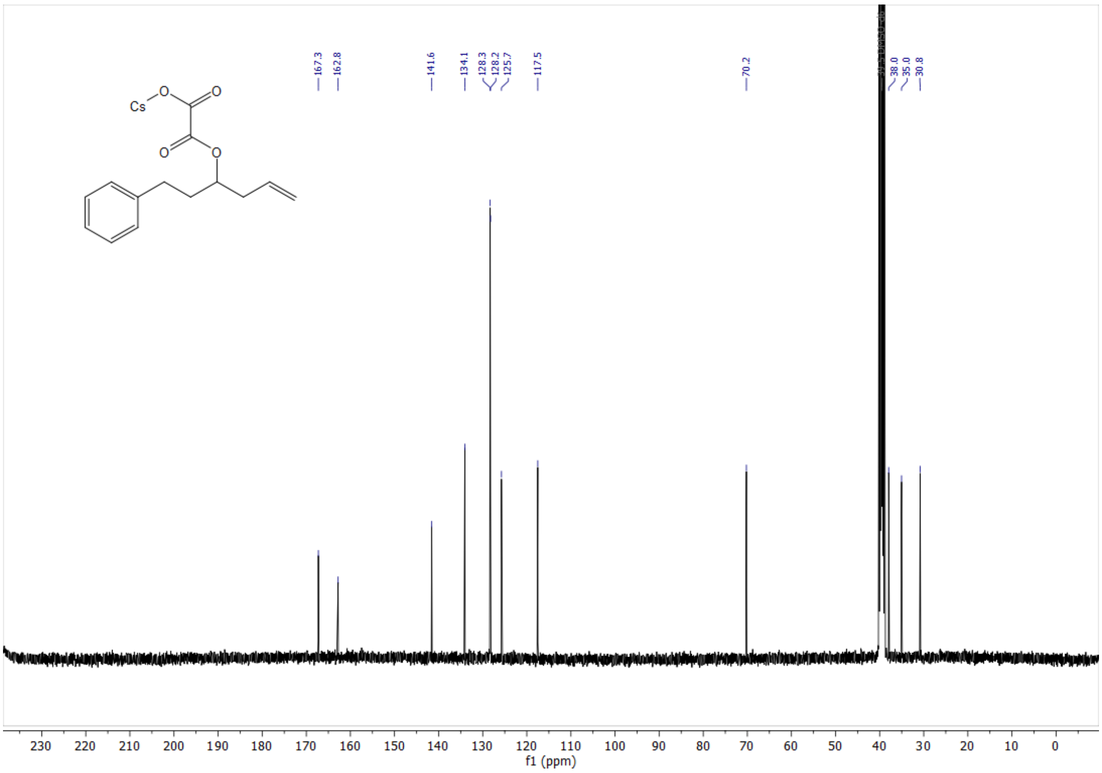

^{1}H NMR Spectrum (400 MHz, DMSO-d_6) of **2i**

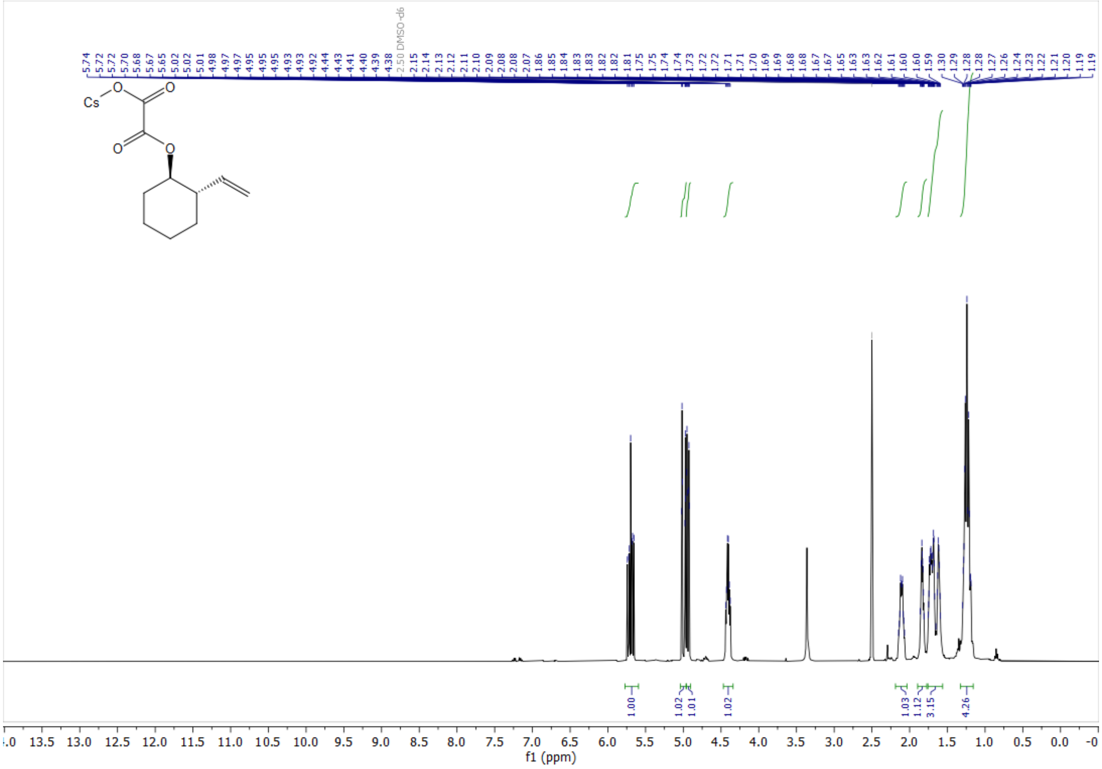

^{13}C {^{1}H} NMR Spectrum (101 MHz, DMSO-d_6) of **2i**

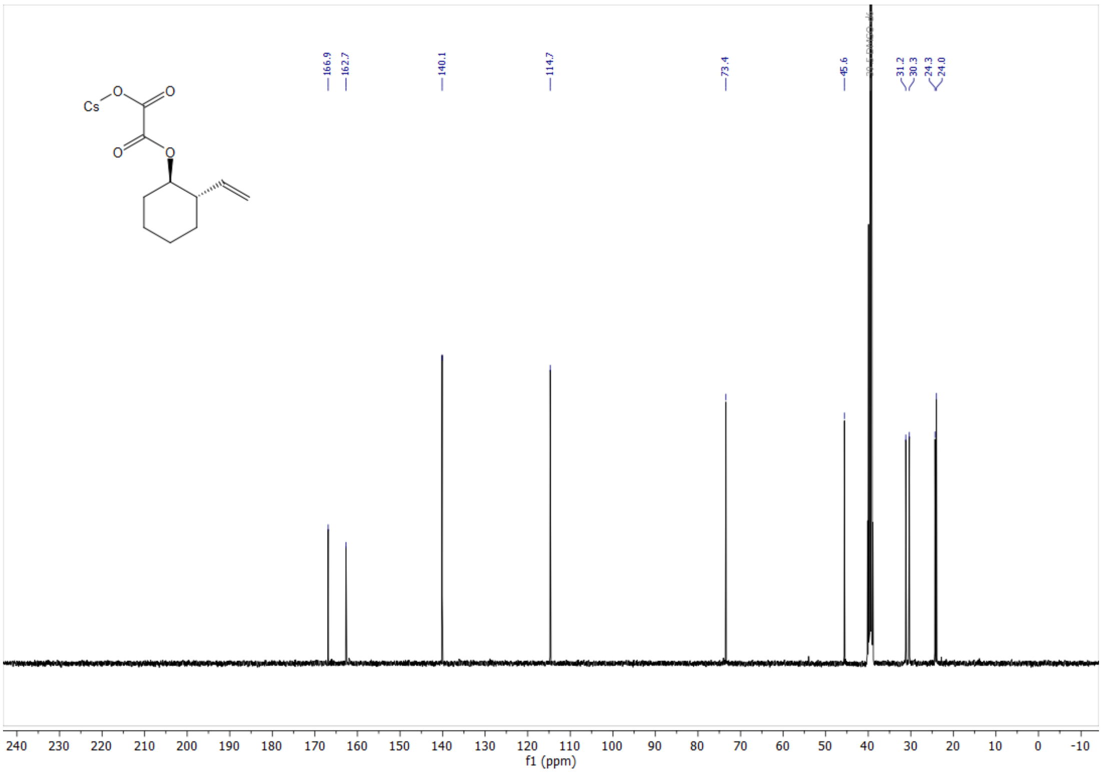

^{1}H NMR Spectrum (400 MHz, DMSO-d_6) of **2j**

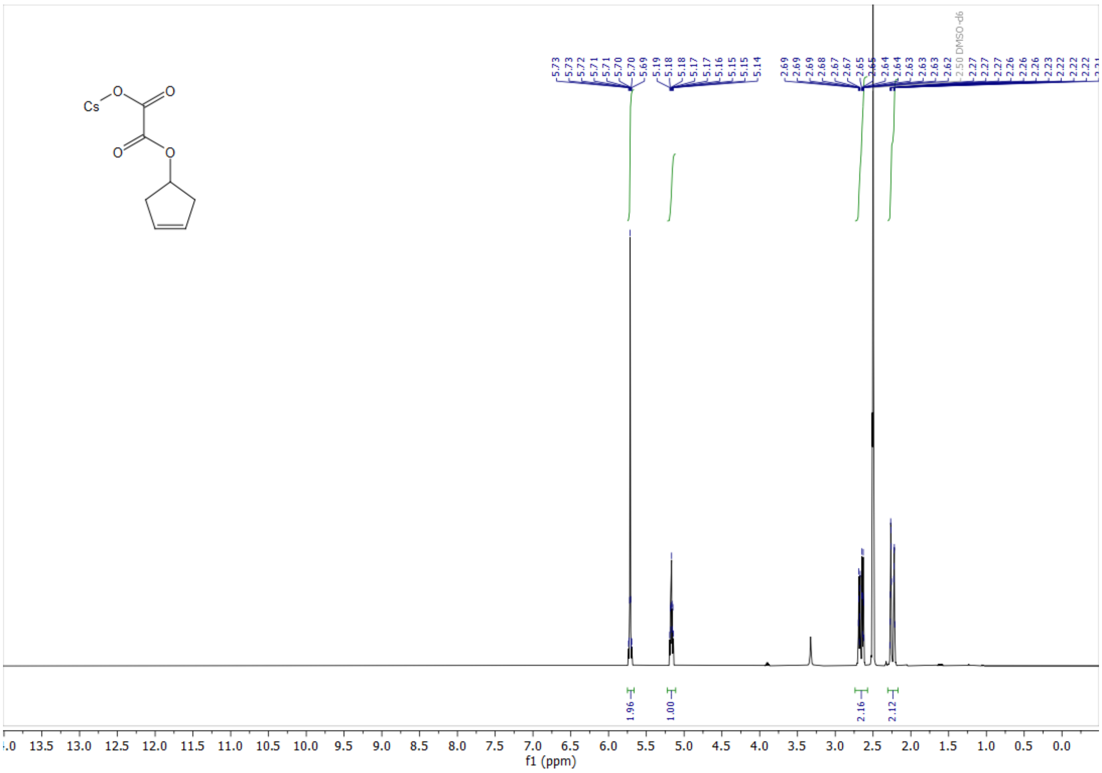

^{13}C {^{1}H} NMR Spectrum (101 MHz, DMSO-d_6) of **2j**

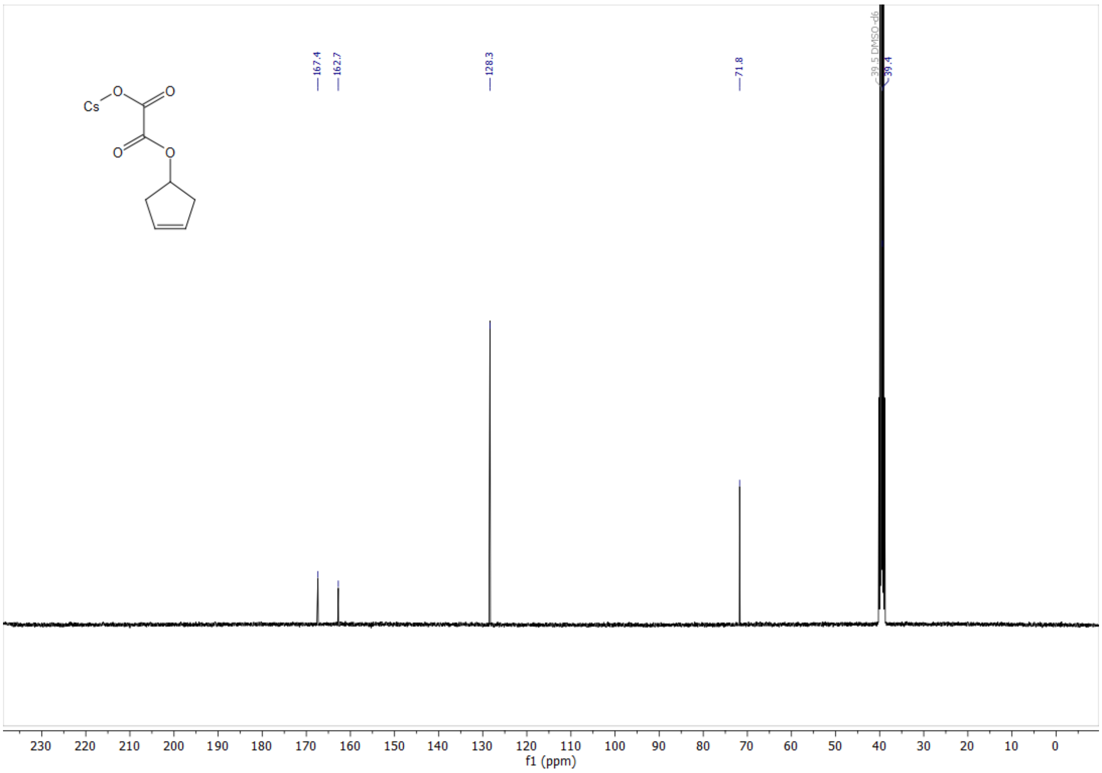

^{1}H NMR Spectrum (400 MHz, DMSO-d_6) of **2l**

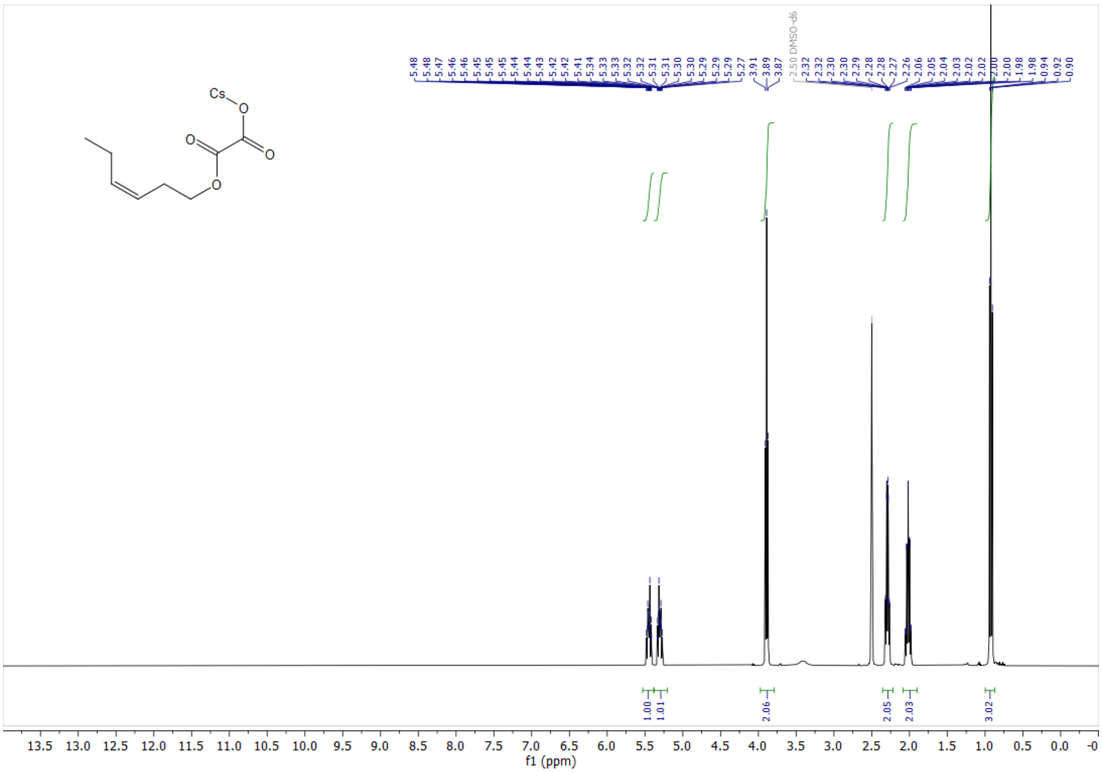

^{13}C {^{1}H} NMR Spectrum (101 MHz, DMSO-d_6) of **2l**

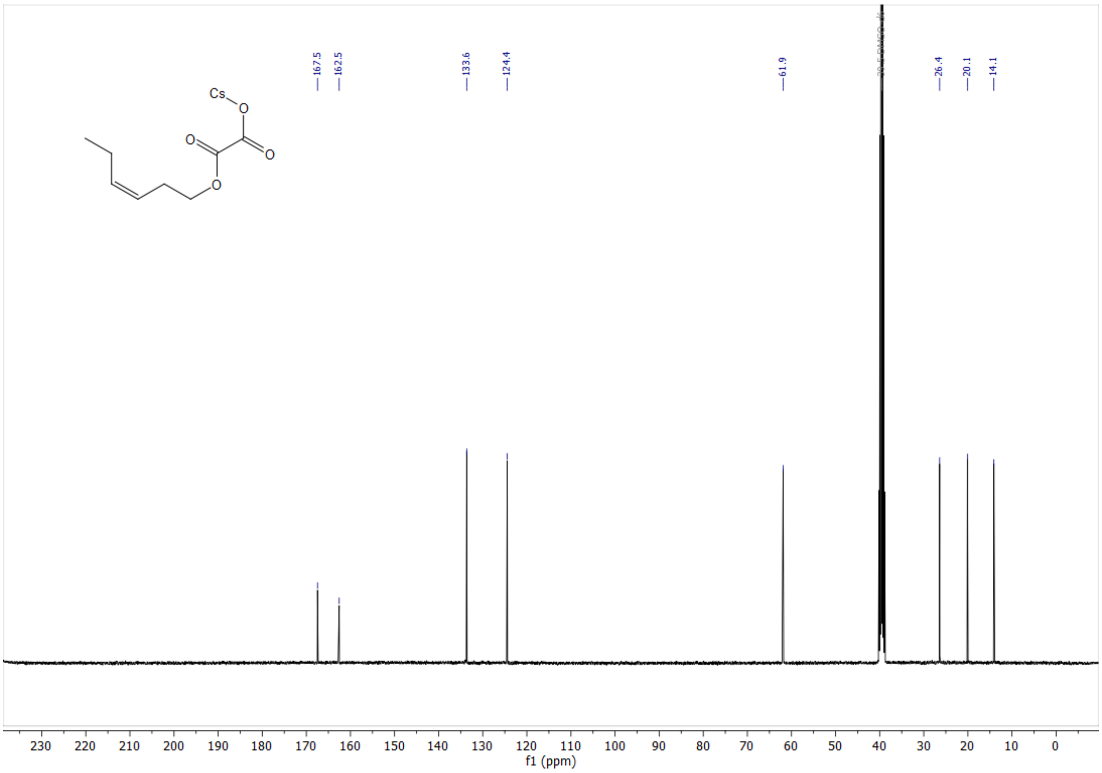

^{1}H NMR Spectrum (400 MHz, DMSO-d_6) of **2m**

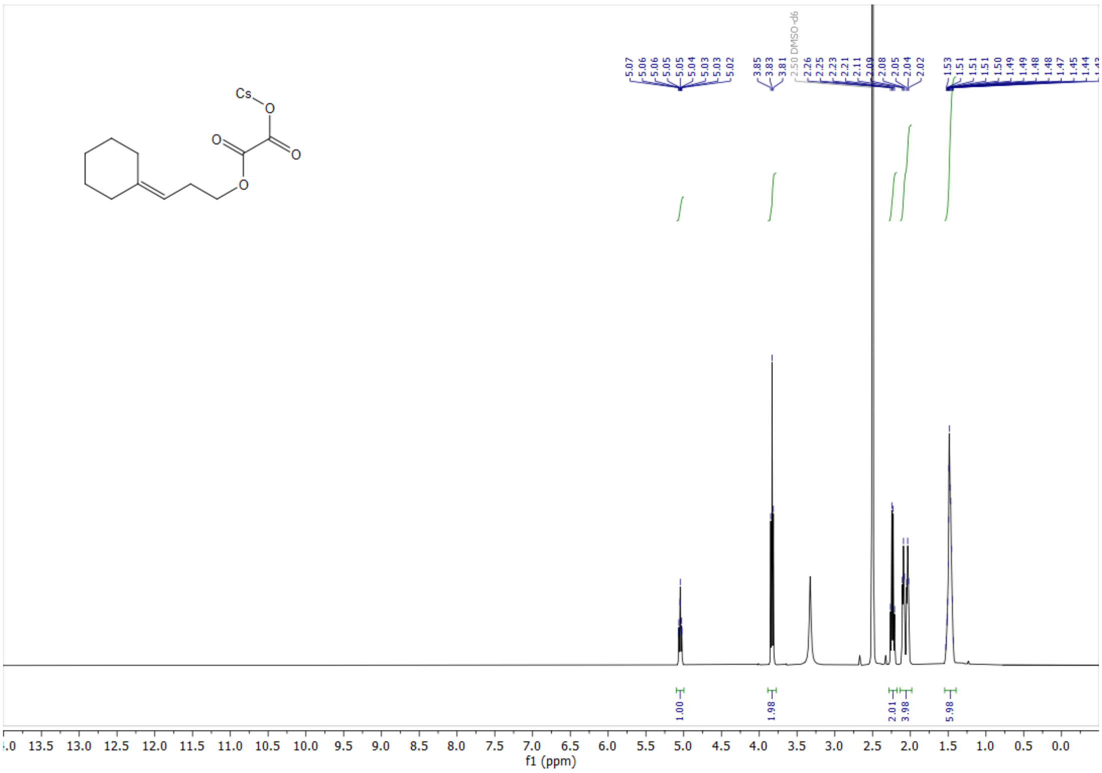

^{13}C {^{1}H} NMR Spectrum (101 MHz, DMSO-d_6) of **2m**

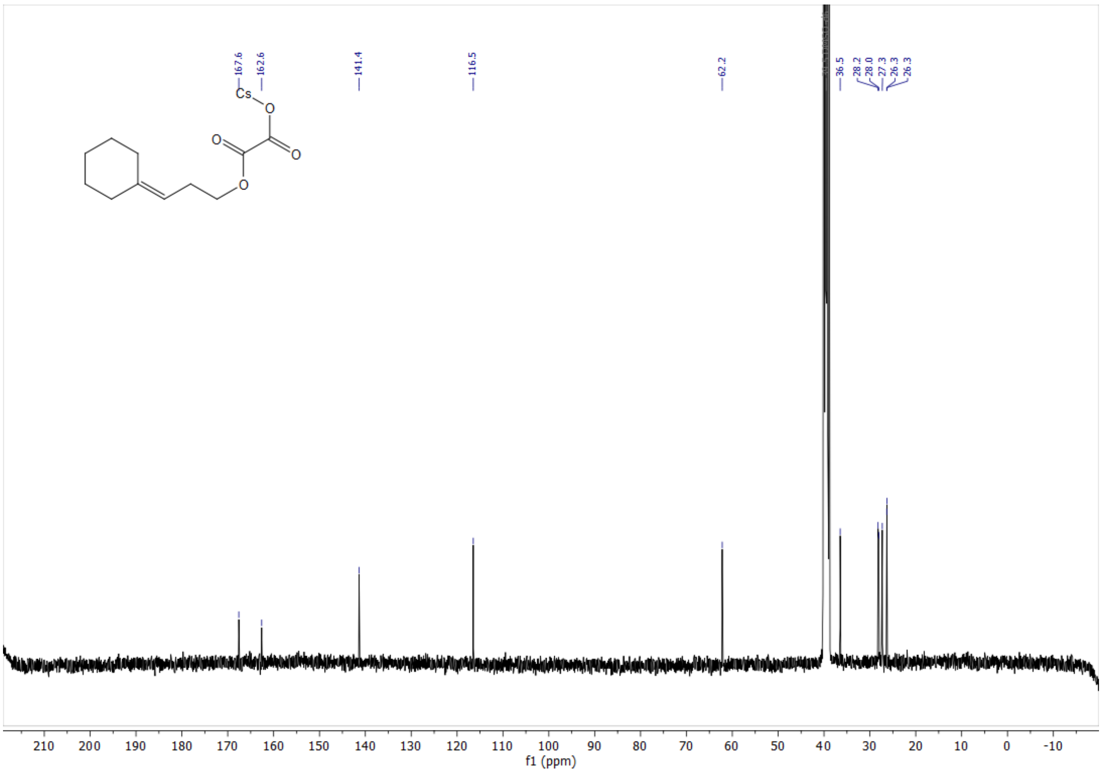

^{1}H NMR Spectrum (400 MHz, DMSO-d_6) of **2o**

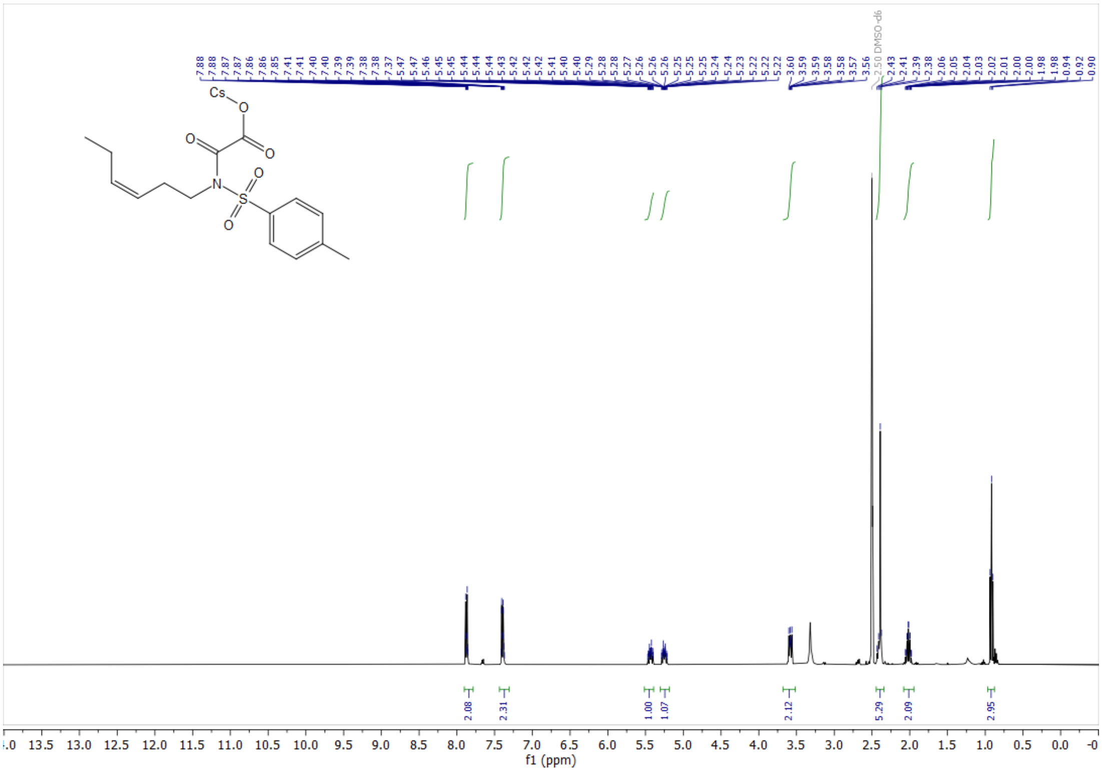

^{13}C {^{1}H} NMR Spectrum (101 MHz, DMSO-d_6) of **2o**

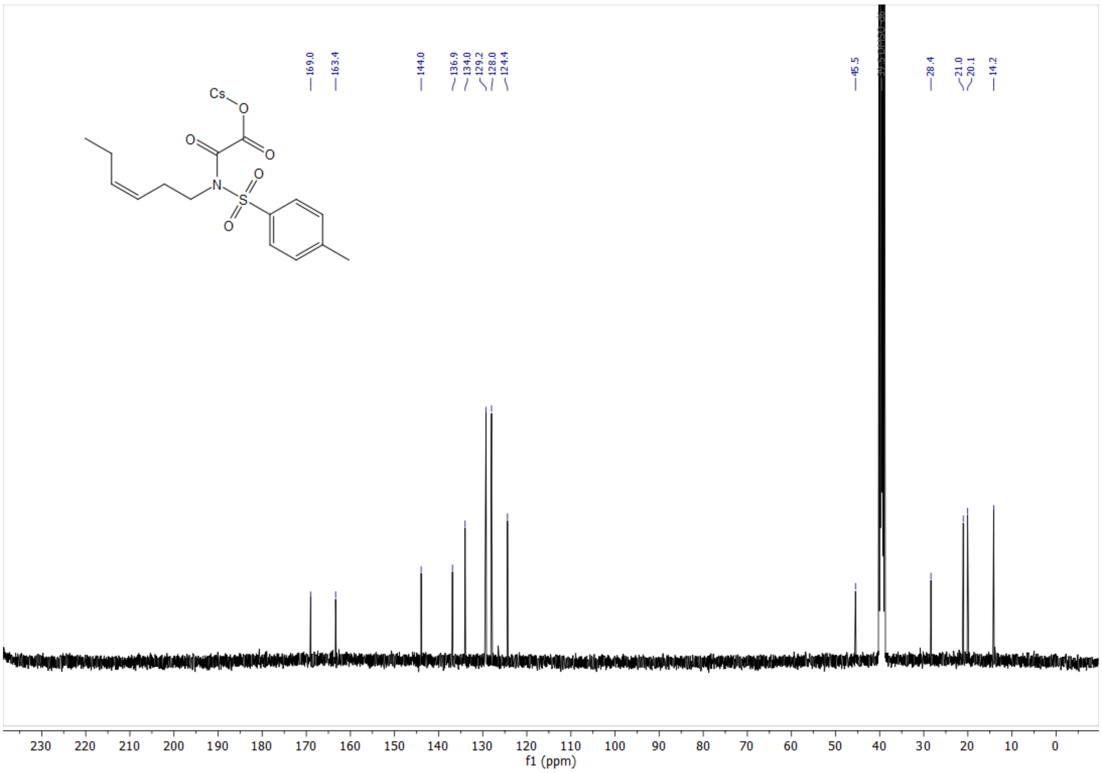

^{1}H NMR Spectrum (400 MHz, DMSO-d_6) of **2p**

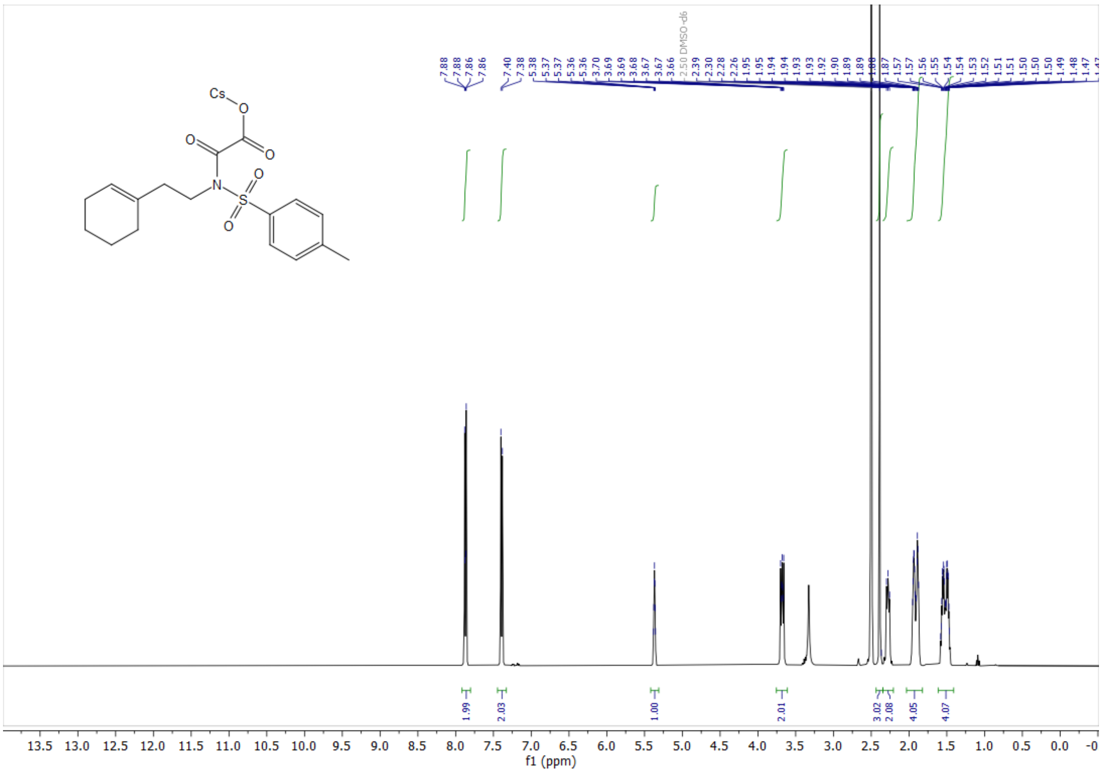

^{13}C {^{1}H} NMR Spectrum (101 MHz, DMSO-d_6) of **2p**

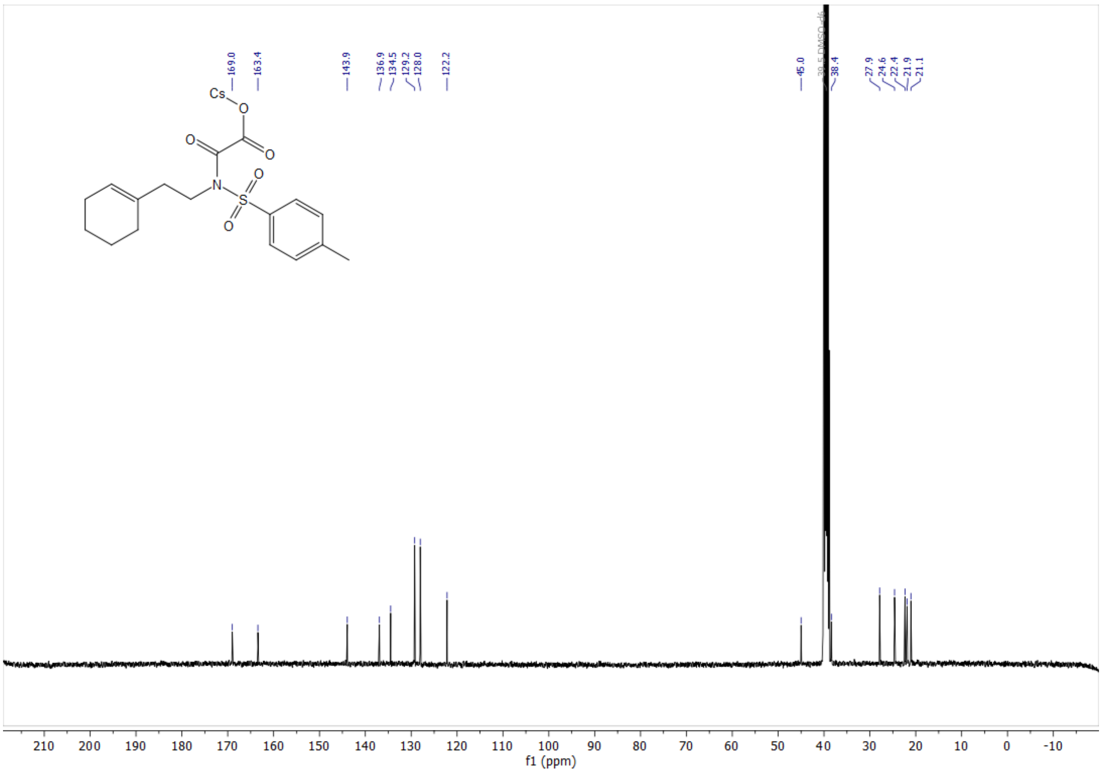

^{1}H NMR Spectrum (400 MHz, DMSO-d_6) of **2q**

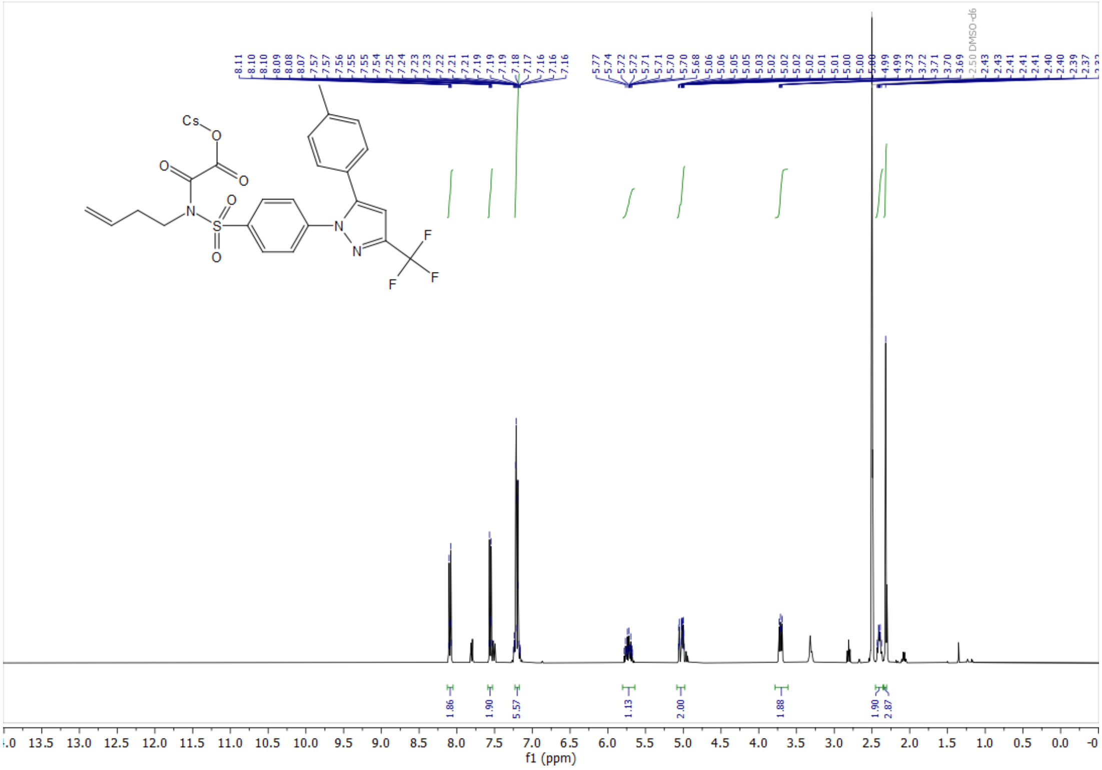

^{13}C {^{1}H} NMR Spectrum (101 MHz, DMSO-d_6) of **2q**

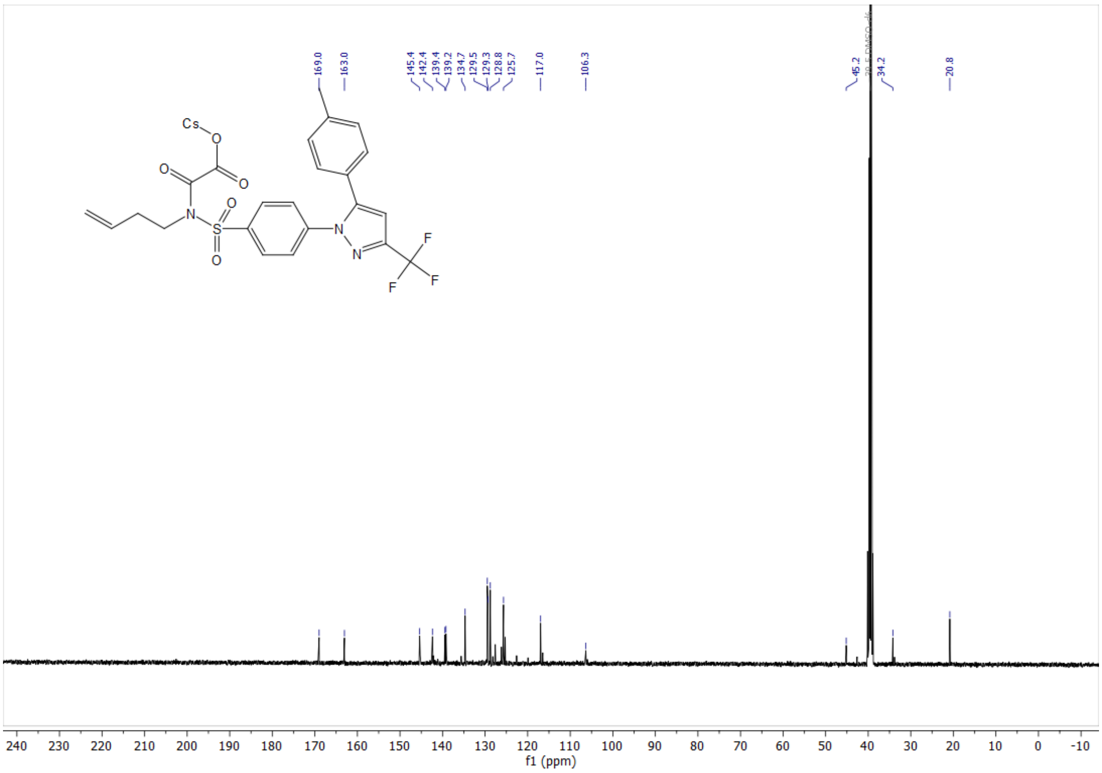

^{19}F {^{1}H} NMR Spectrum (376 MHz, DMSO-d_6) of **2q**

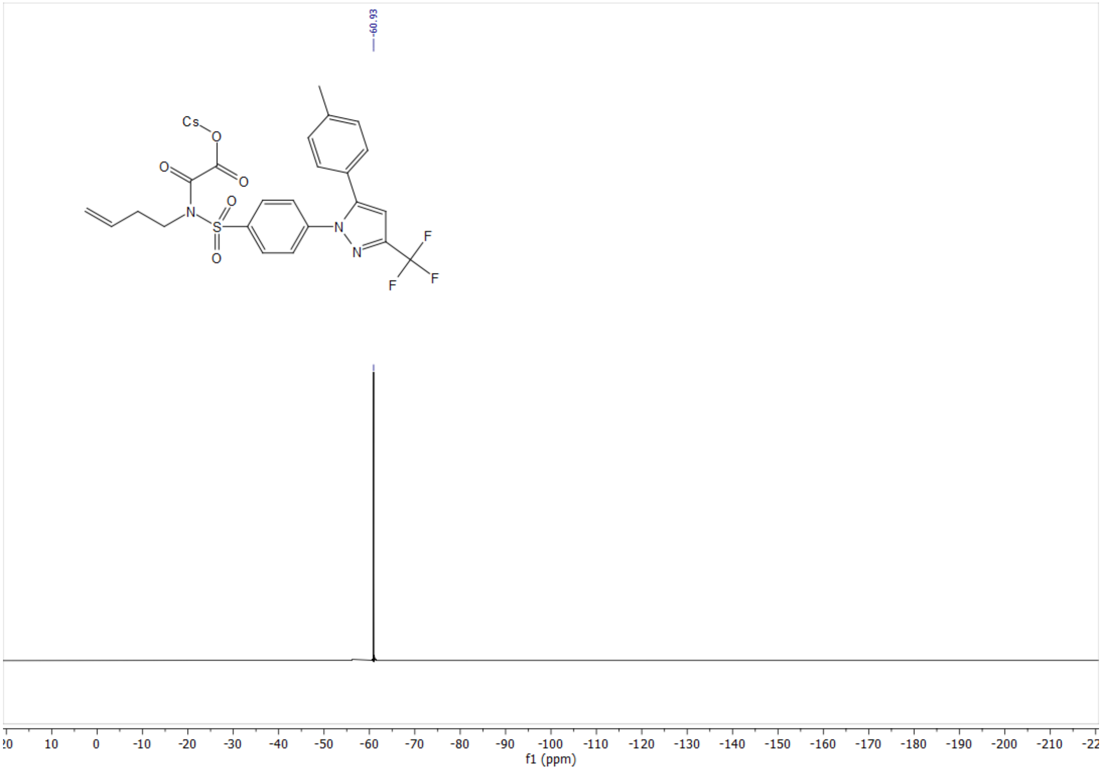

^{1}H NMR Spectrum (400 MHz, $CDCl_3$) of **5a**

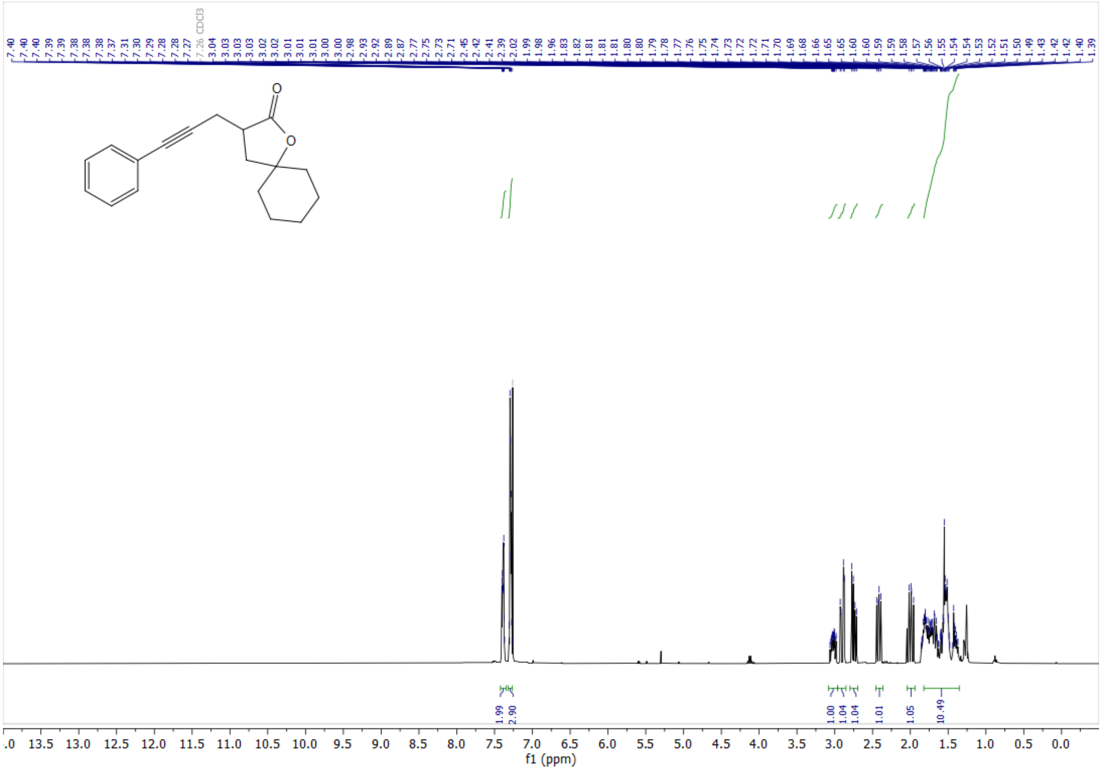

^{13}C {^{1}H} NMR Spectrum (101 MHz, $CDCl_3$) of **5a**

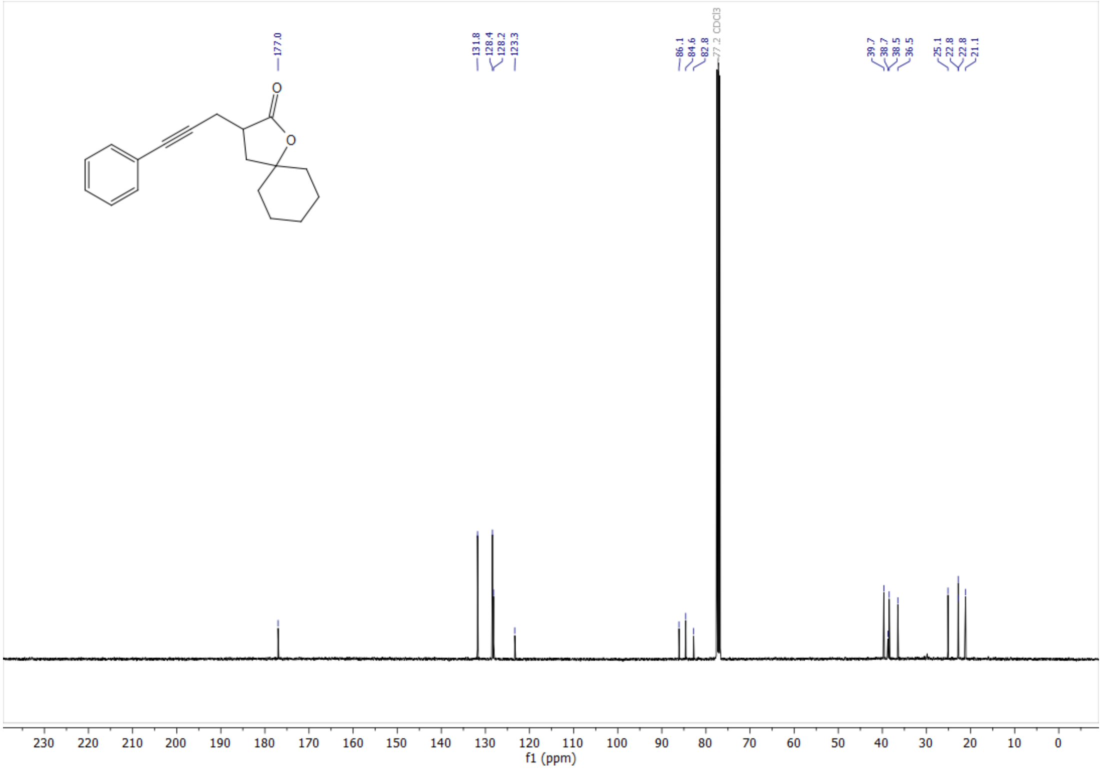

^{1}H NMR Spectrum (400 MHz, $CDCl_3$) of **5b**

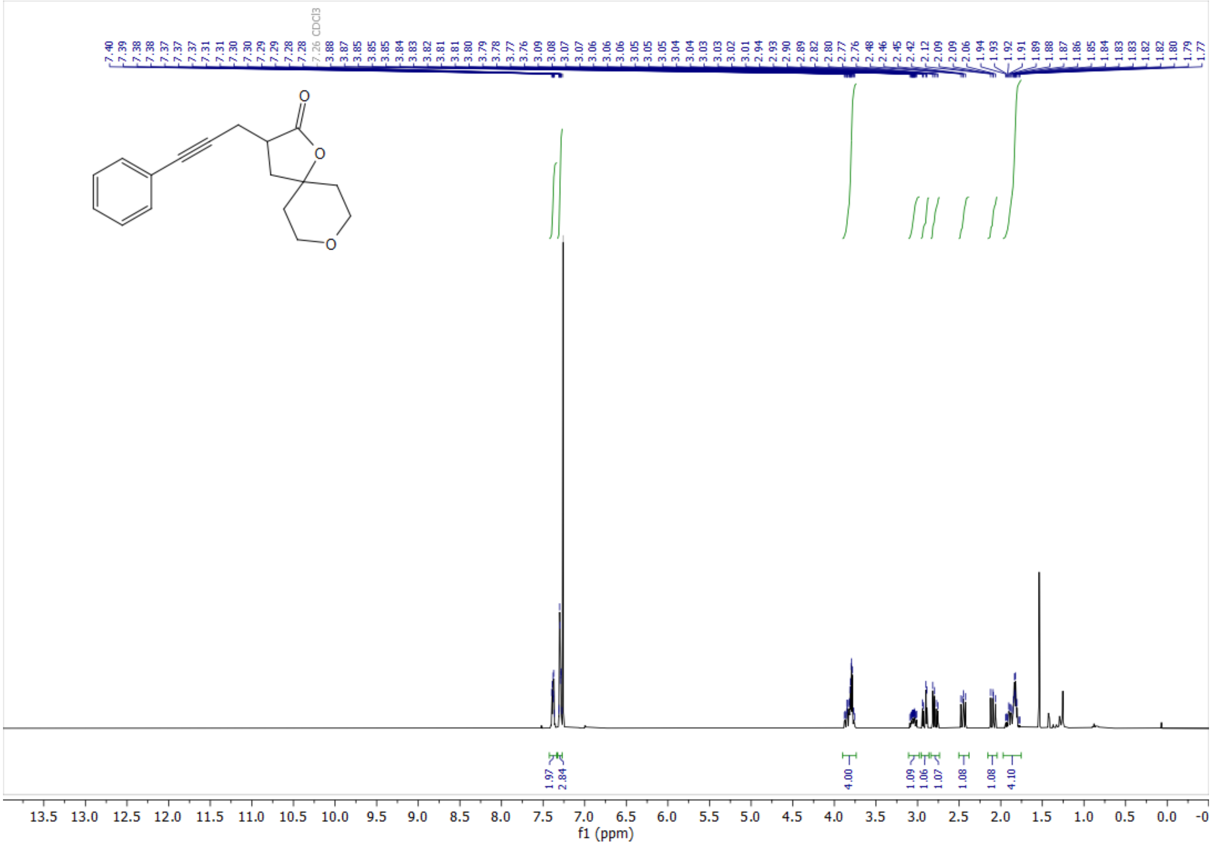

^{13}C {^{1}H} NMR Spectrum (101 MHz, $CDCl_3$) of **5b**

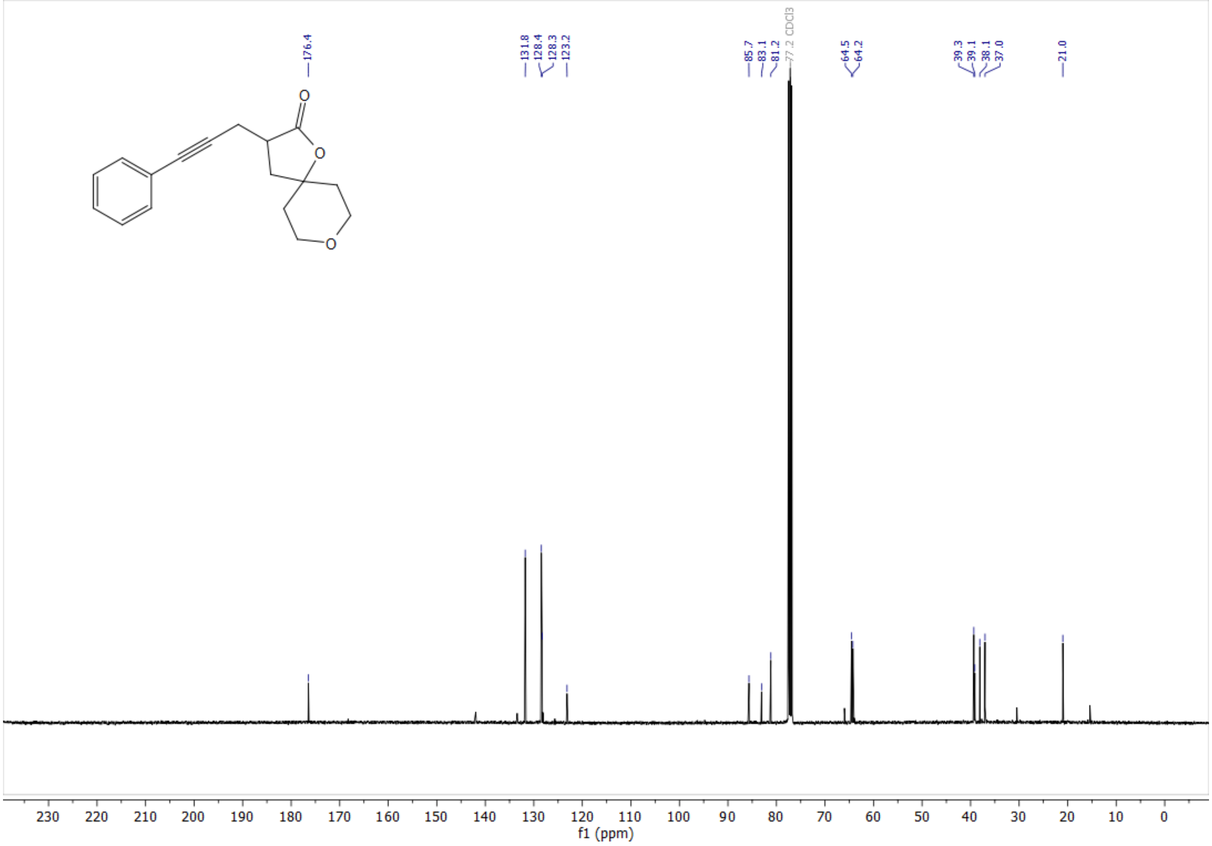

^{1}H NMR Spectrum (400 MHz, $CDCl_3$) of **5c**

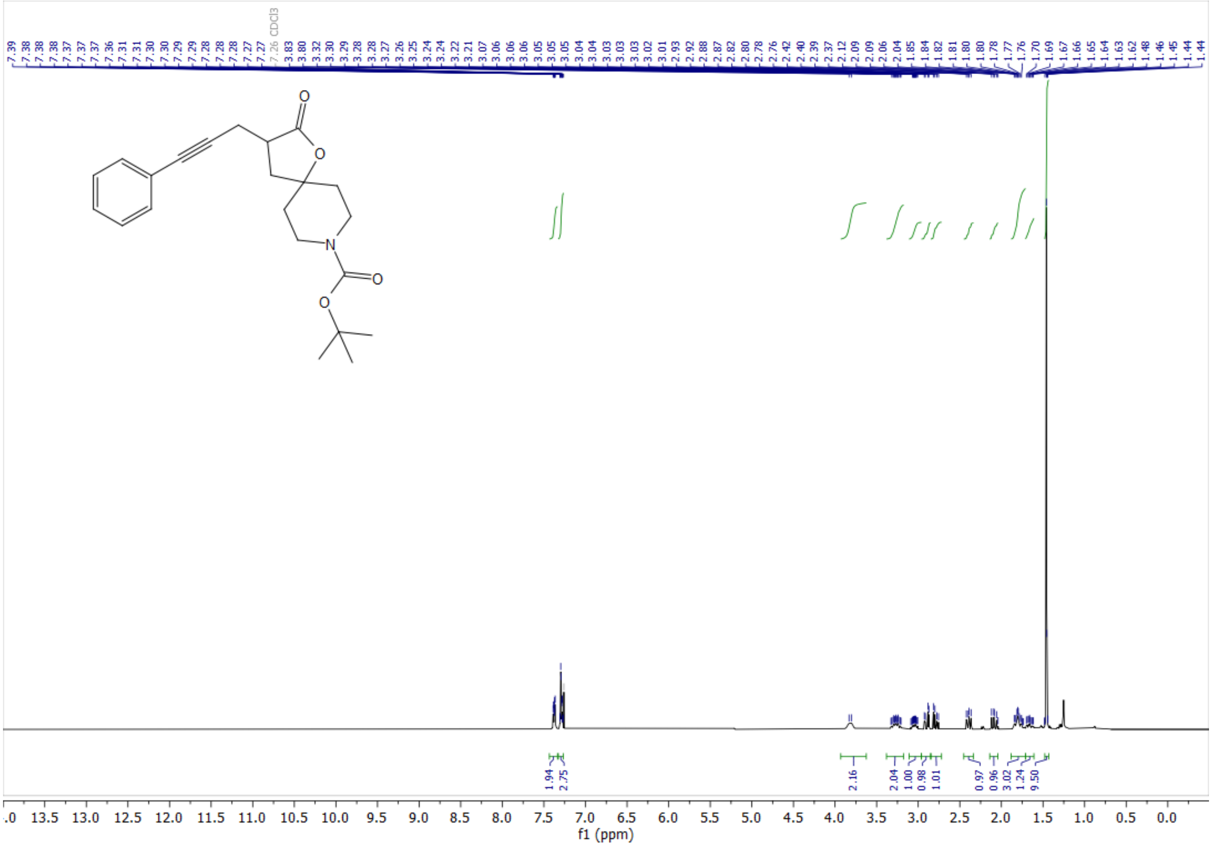

$^{13}C\ \{^{1}H\}$ NMR Spectrum (101 MHz, $CDCl_3$) of **5c**

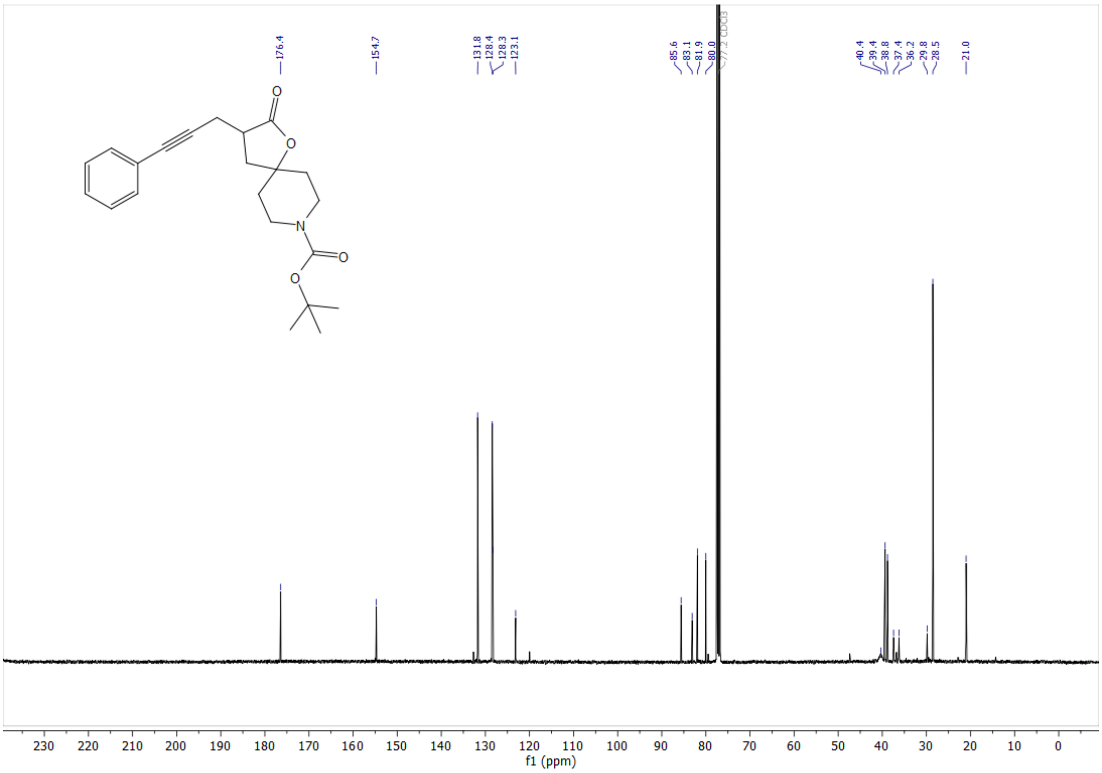

^{1}H NMR Spectrum (400 MHz, $CDCl_3$) of **5d**

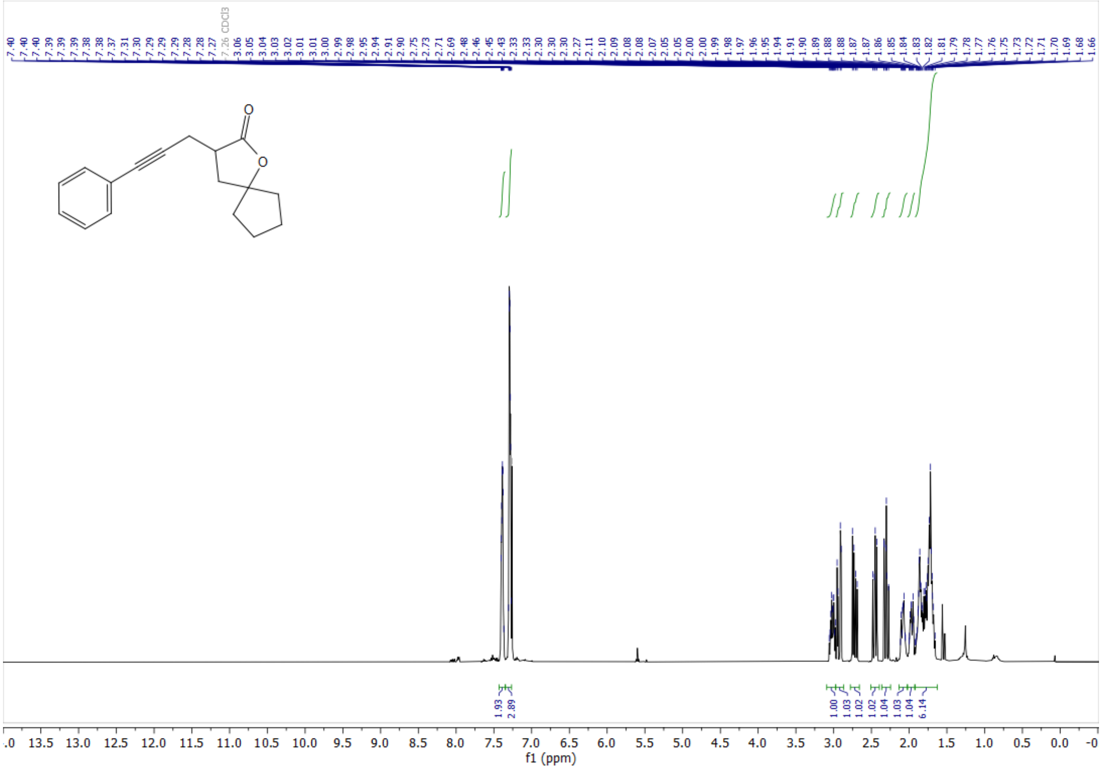

^{13}C {^{1}H} NMR Spectrum (101 MHz, $CDCl_3$) of **5d**

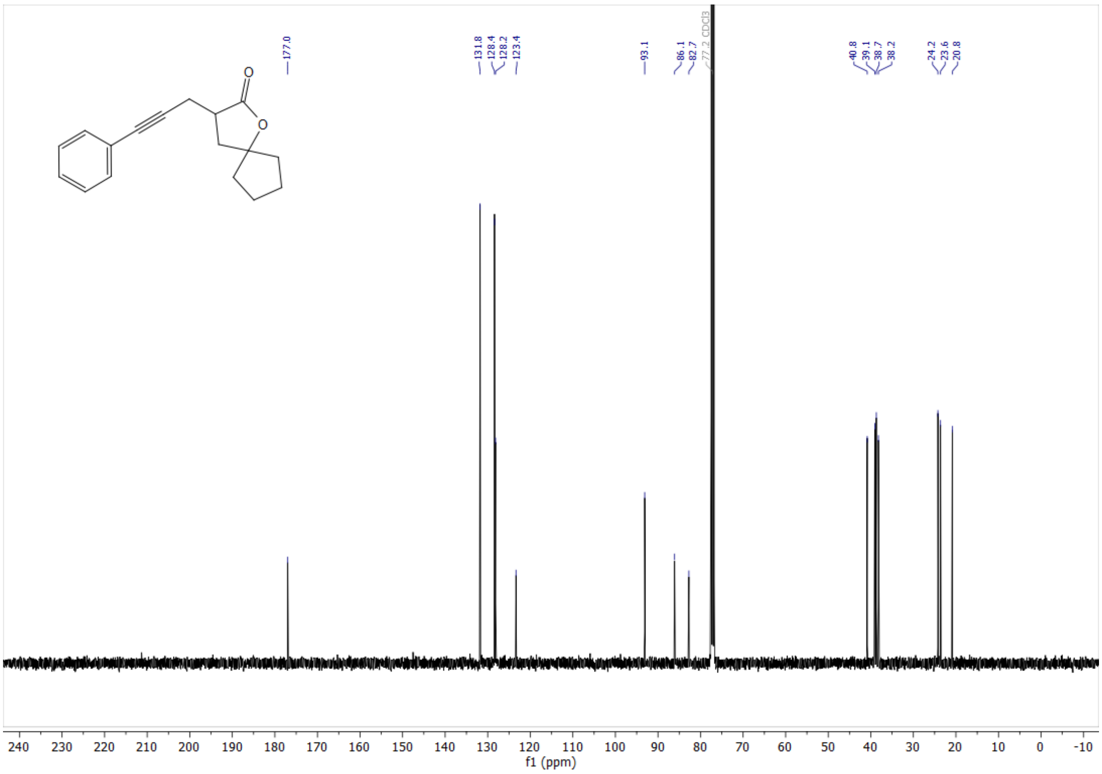

^{1}H NMR Spectrum (400 MHz, $CDCl_3$) of **5e**

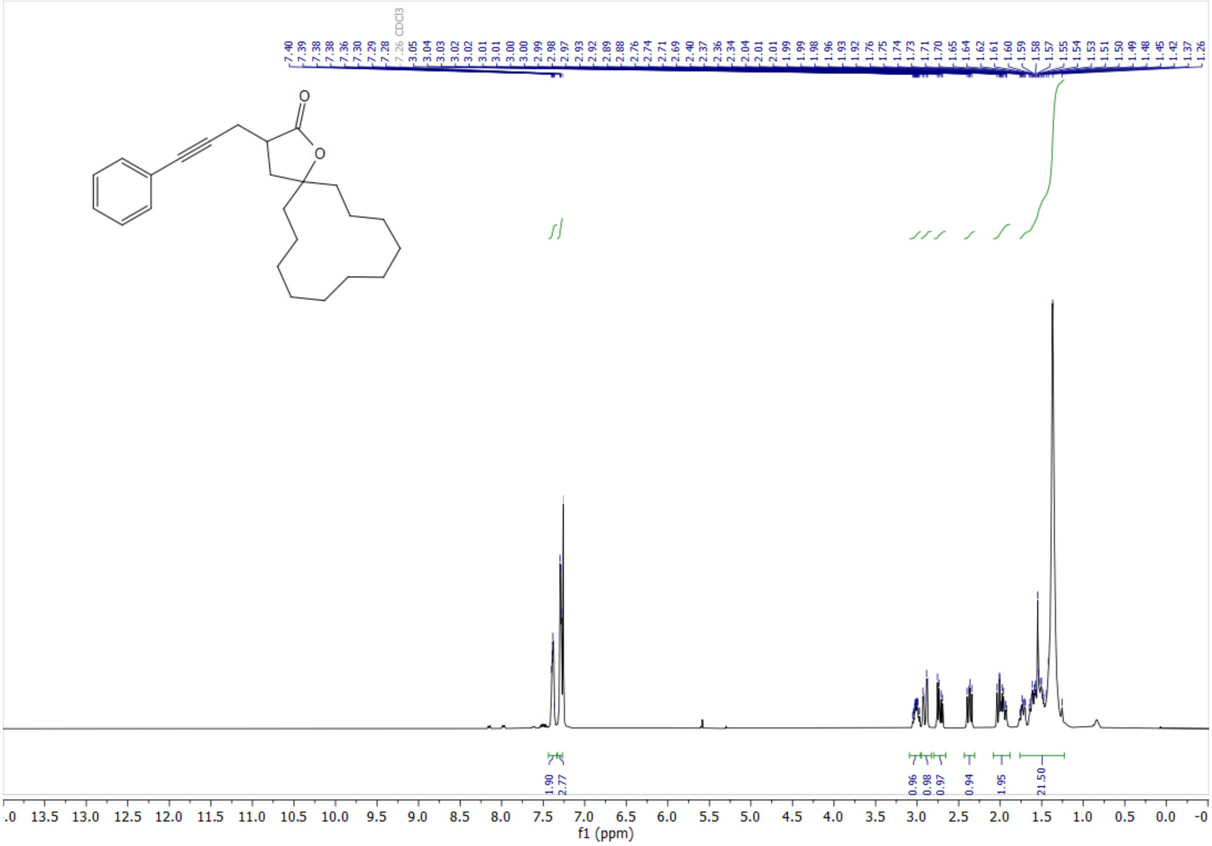

^{13}C {^{1}H} NMR Spectrum (101 MHz, $CDCl_3$) of **5e**

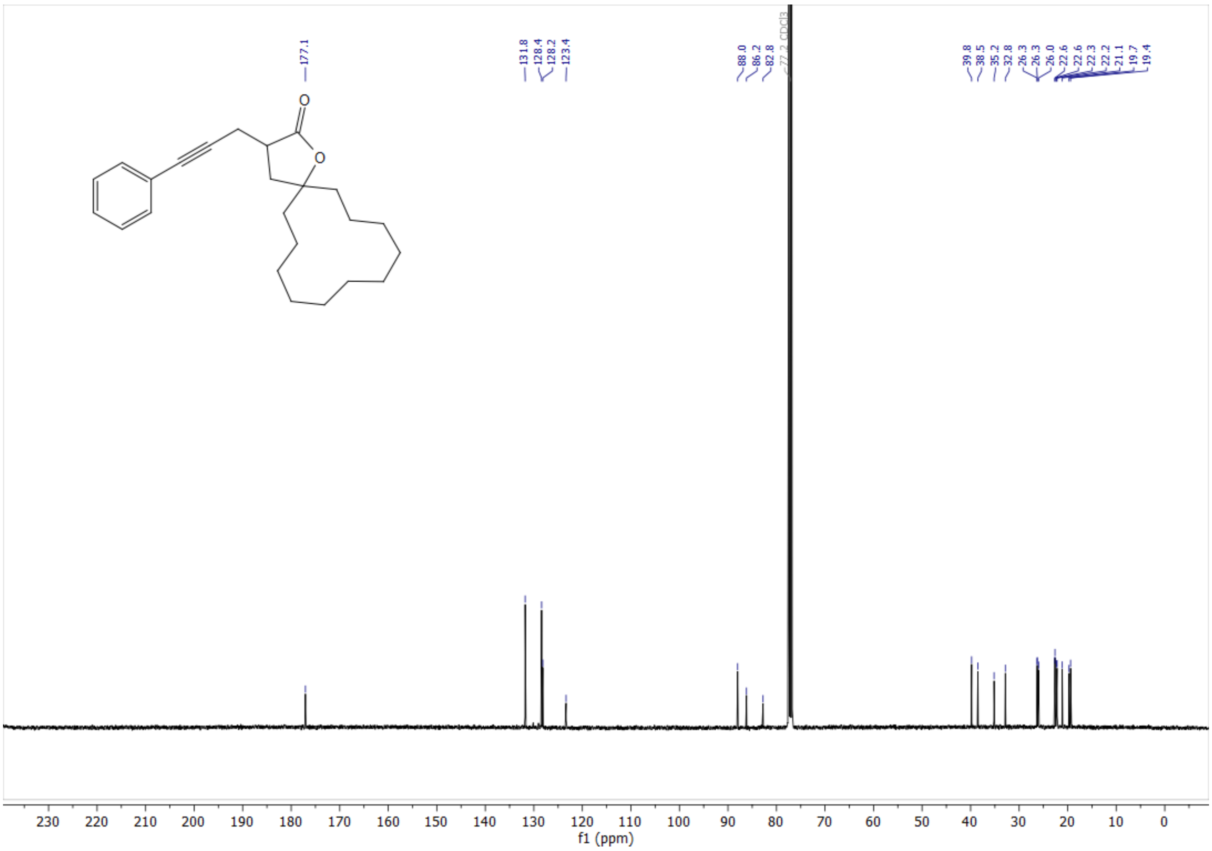

^{1}H NMR Spectrum (400 MHz, $CDCl_3$) of **5f**

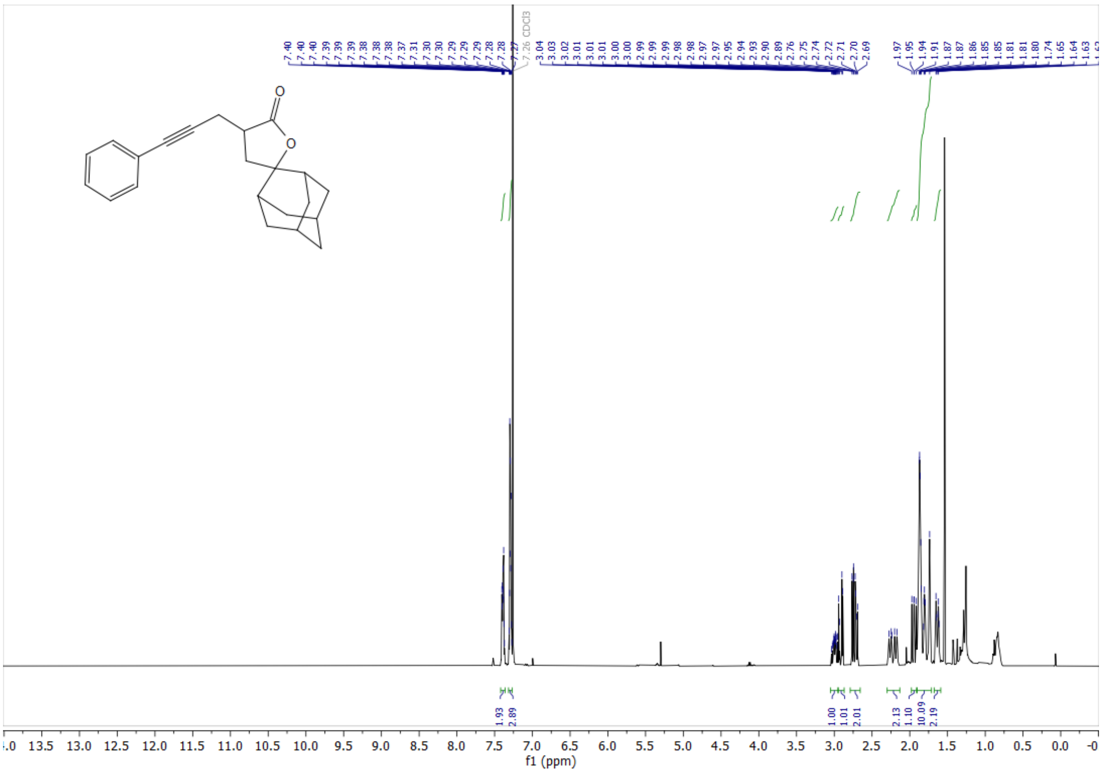

^{13}C {^{1}H} NMR Spectrum (101 MHz, $CDCl_3$) of **5f**

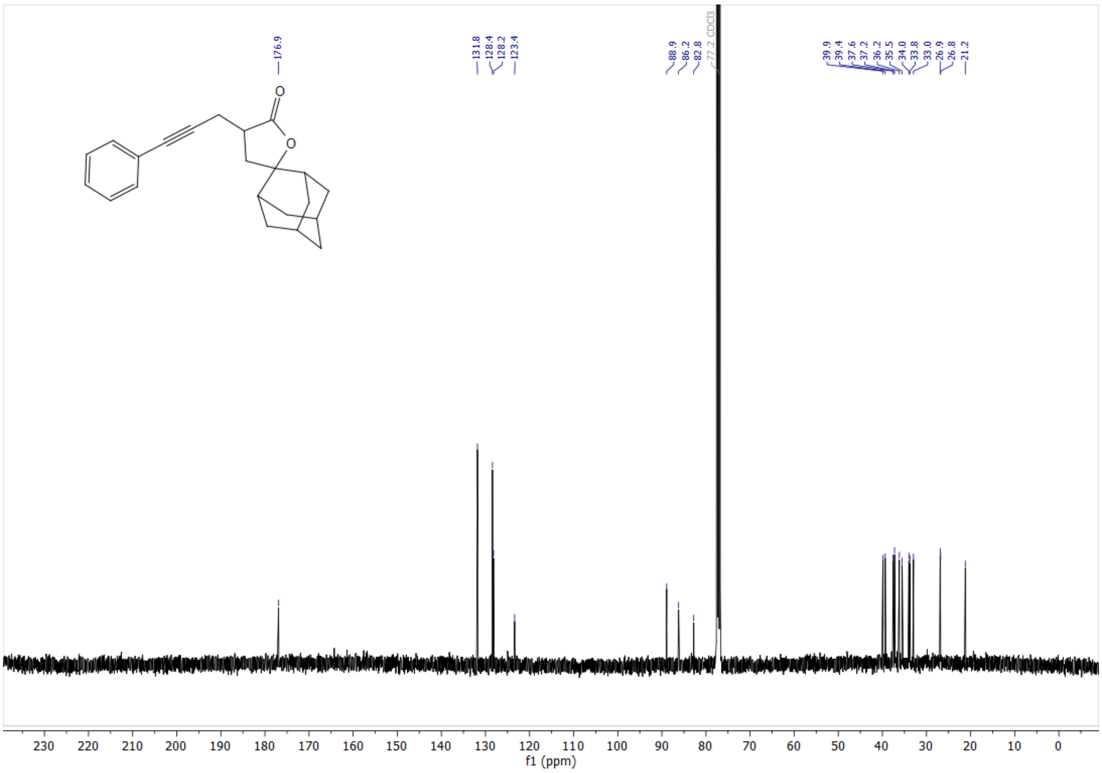

^{1}H NMR Spectrum (400 MHz, $CDCl_3$) of **5g** Major

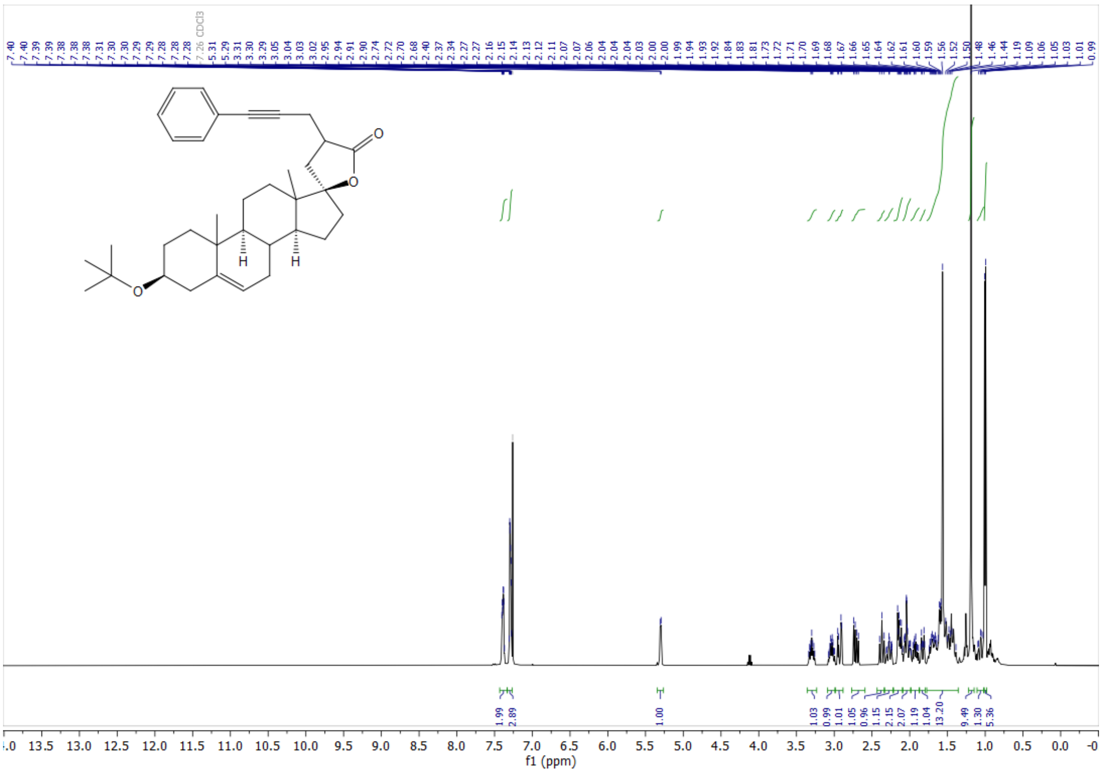

^{13}C {^{1}H} NMR Spectrum (101 MHz, $CDCl_3$) of **5g** Major

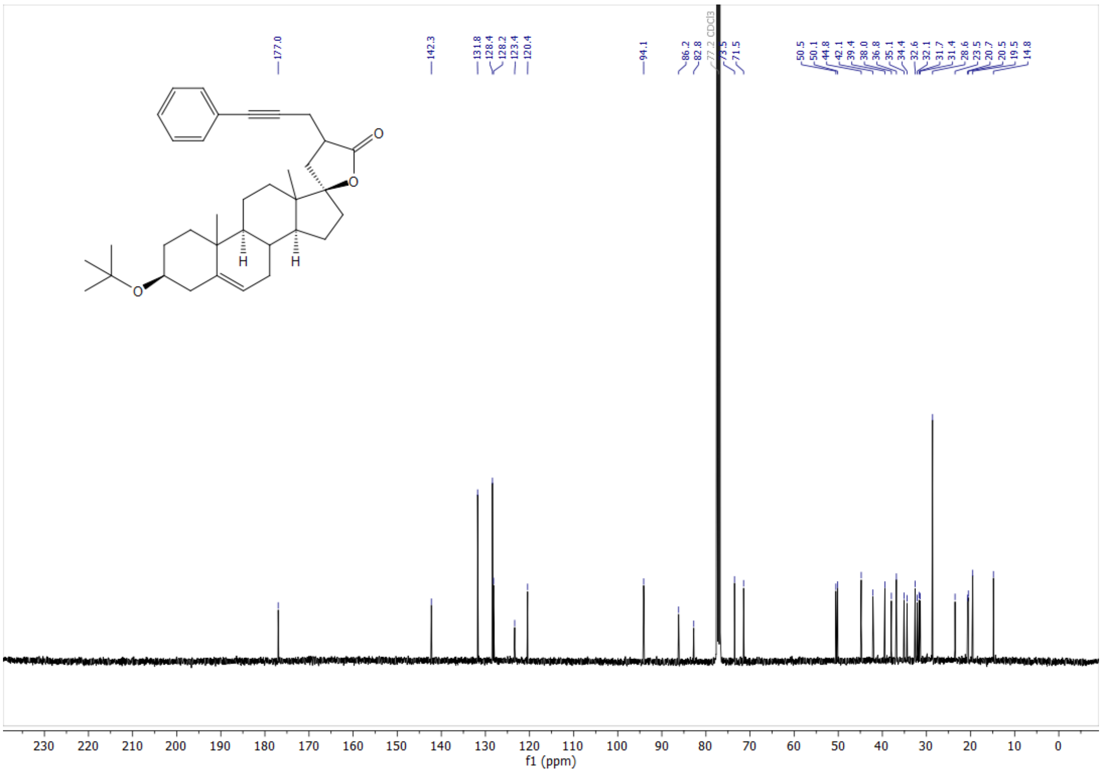

^{1}H NMR Spectrum (400 MHz, $CDCl_3$) of **5g** Minor

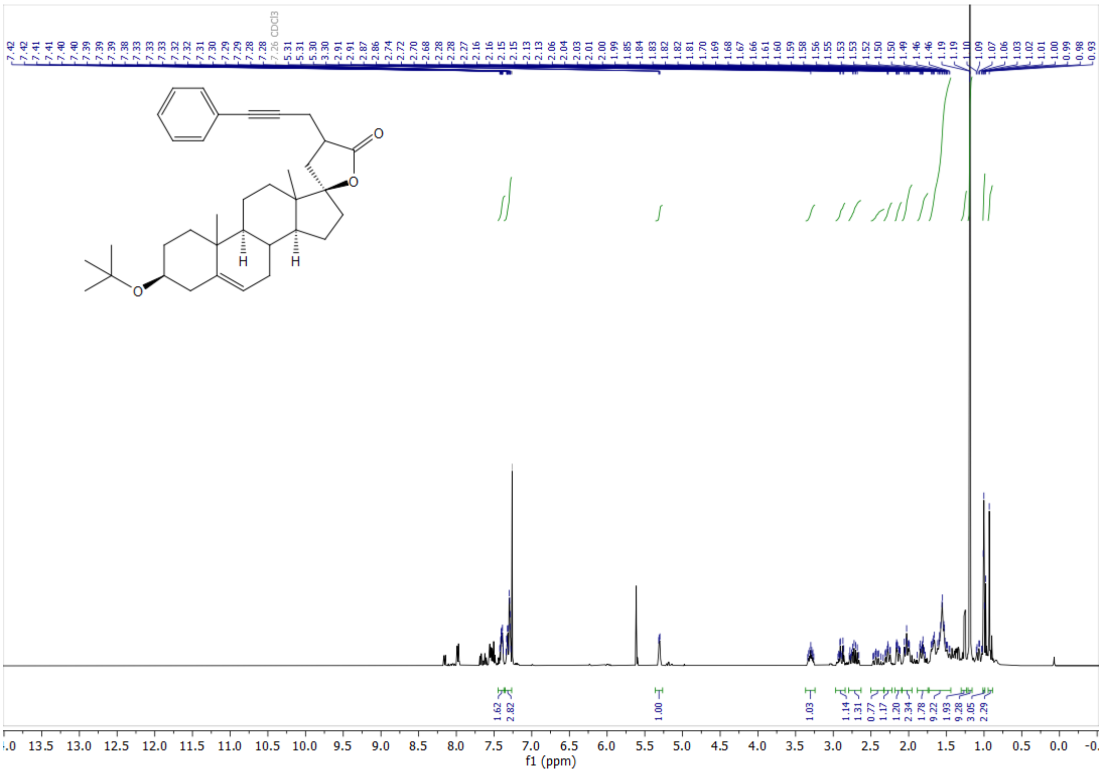

^{13}C {^{1}H} NMR Spectrum (101 MHz, $CDCl_3$) of **5g** Minor

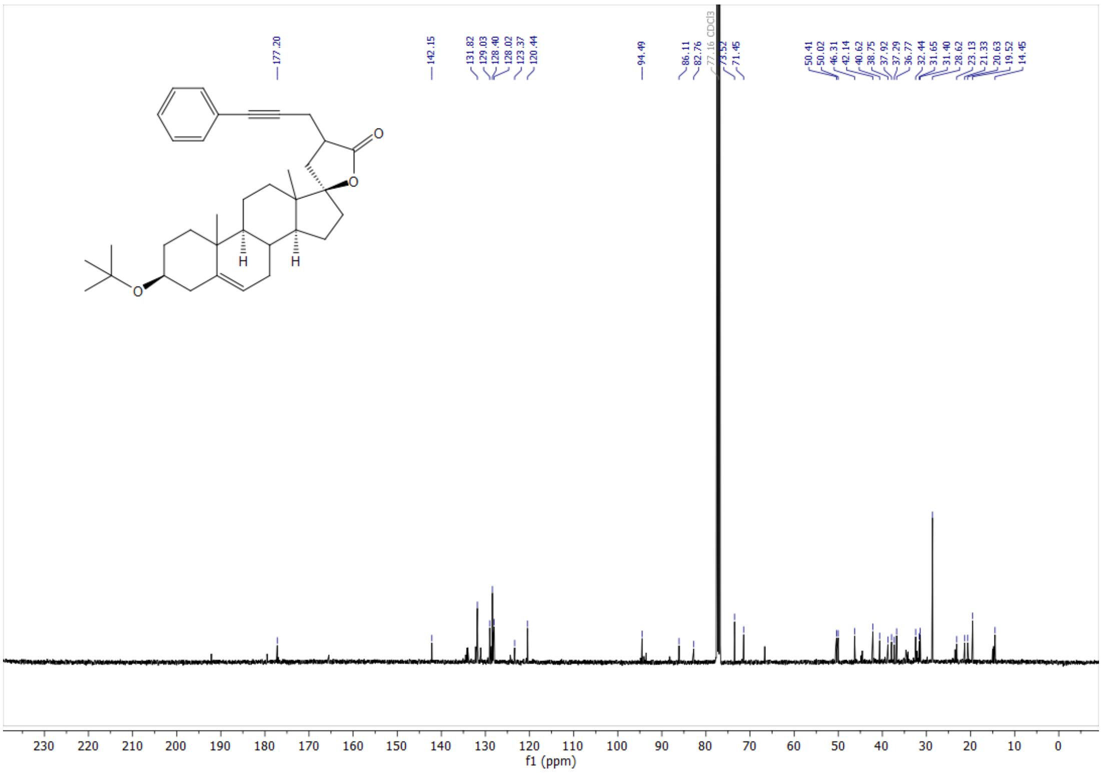

^{1}H NMR Spectrum (400 MHz, $CDCl_3$) of **5h** Major

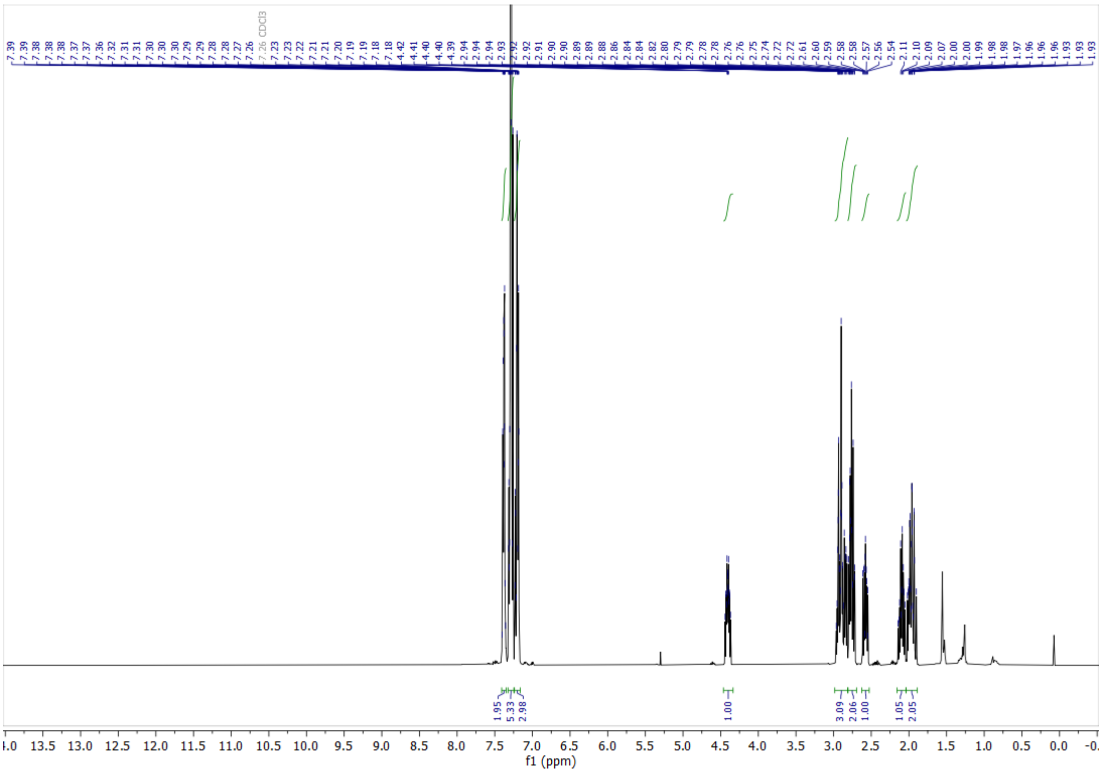

^{13}C {^{1}H} NMR Spectrum (101 MHz, $CDCl_3$) of **5h** Major

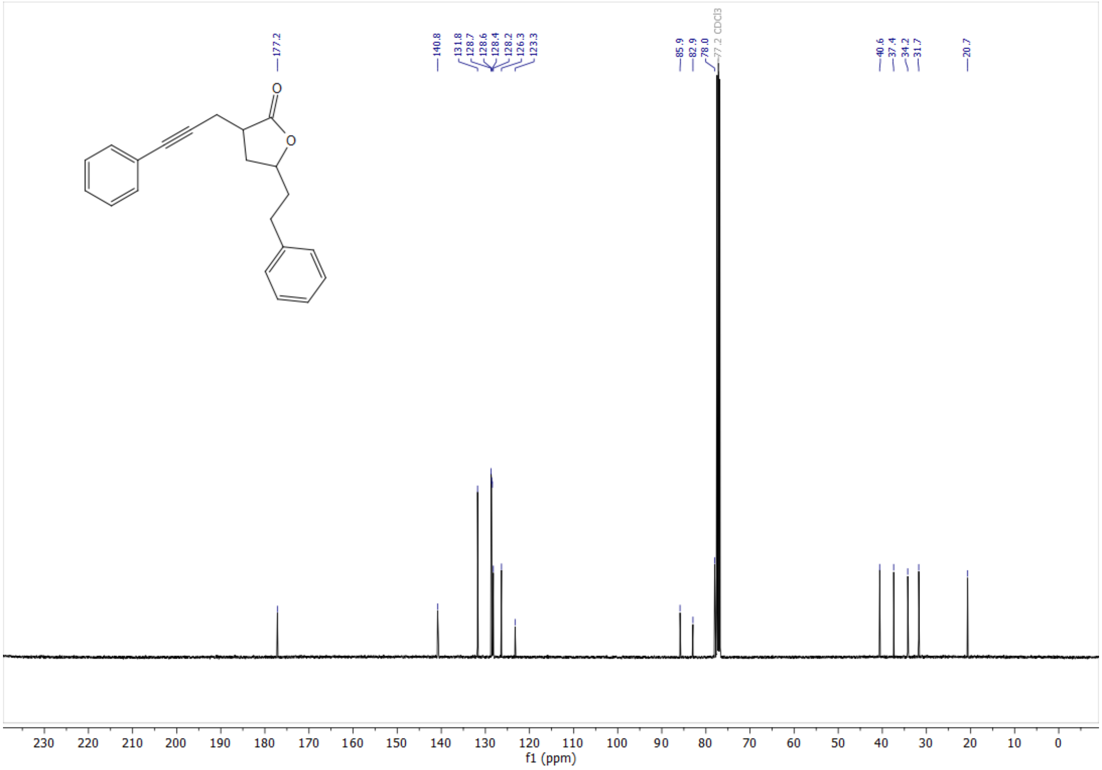

^{1}H NMR Spectrum (400 MHz, $CDCl_3$) of **5h** Minor

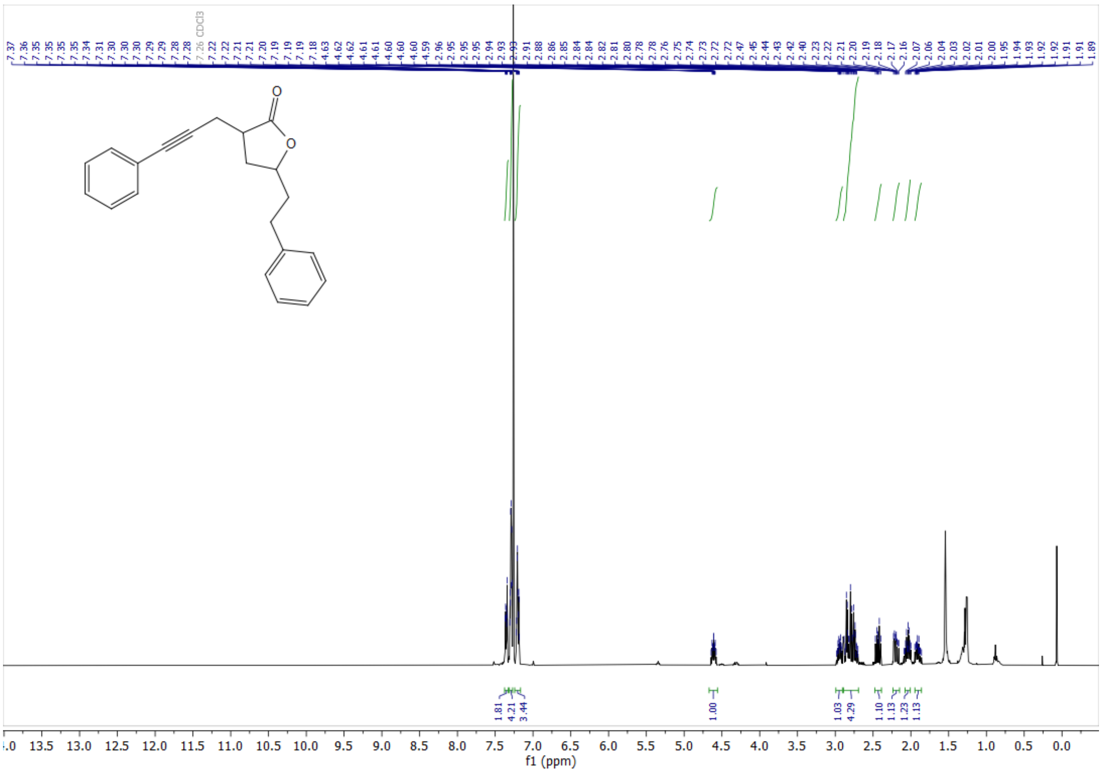

^{13}C {^{1}H} NMR Spectrum (400 MHz, $CDCl_3$) of **5h** Minor

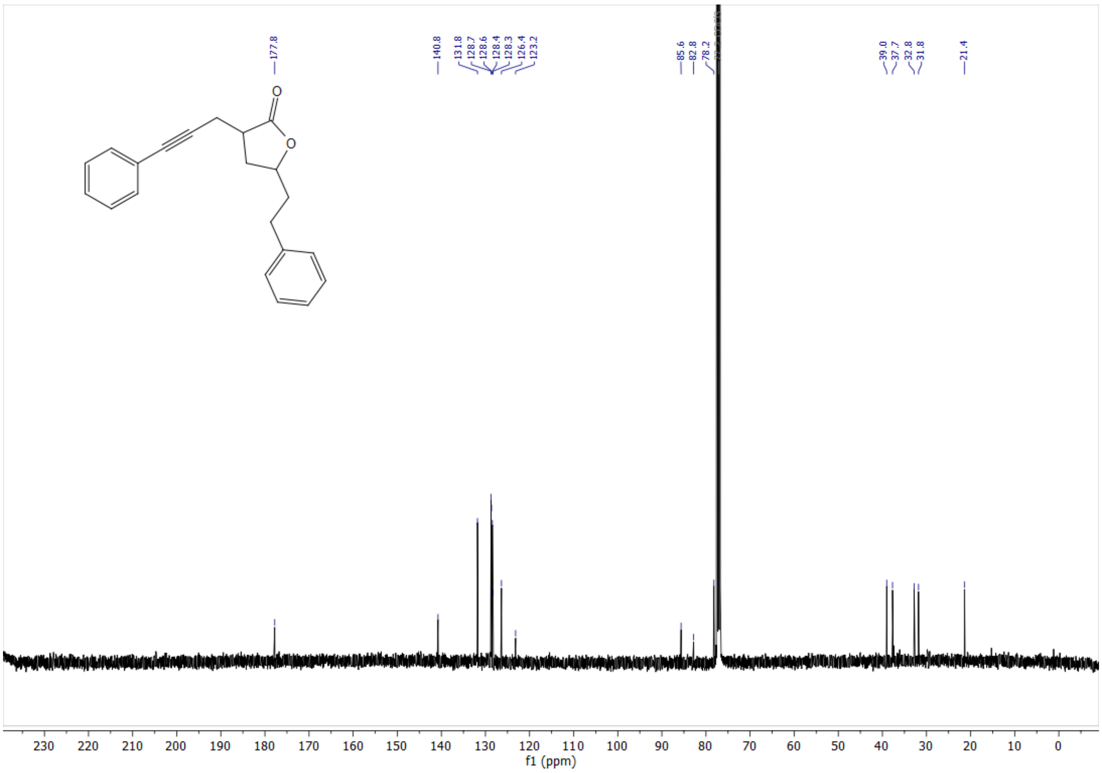

^{1}H NMR Spectrum (400 MHz, $CDCl_3$) of **5i**

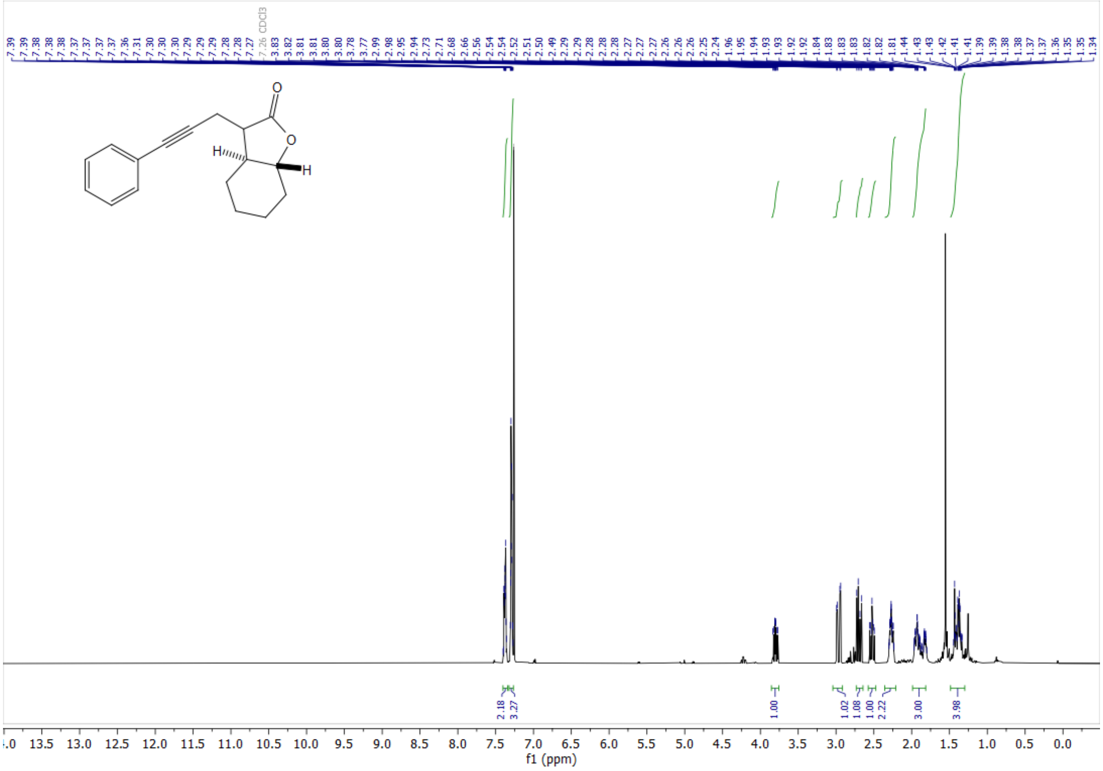

^{13}C {^{1}H} NMR Spectrum (400 MHz, $CDCl_3$) of **5i**

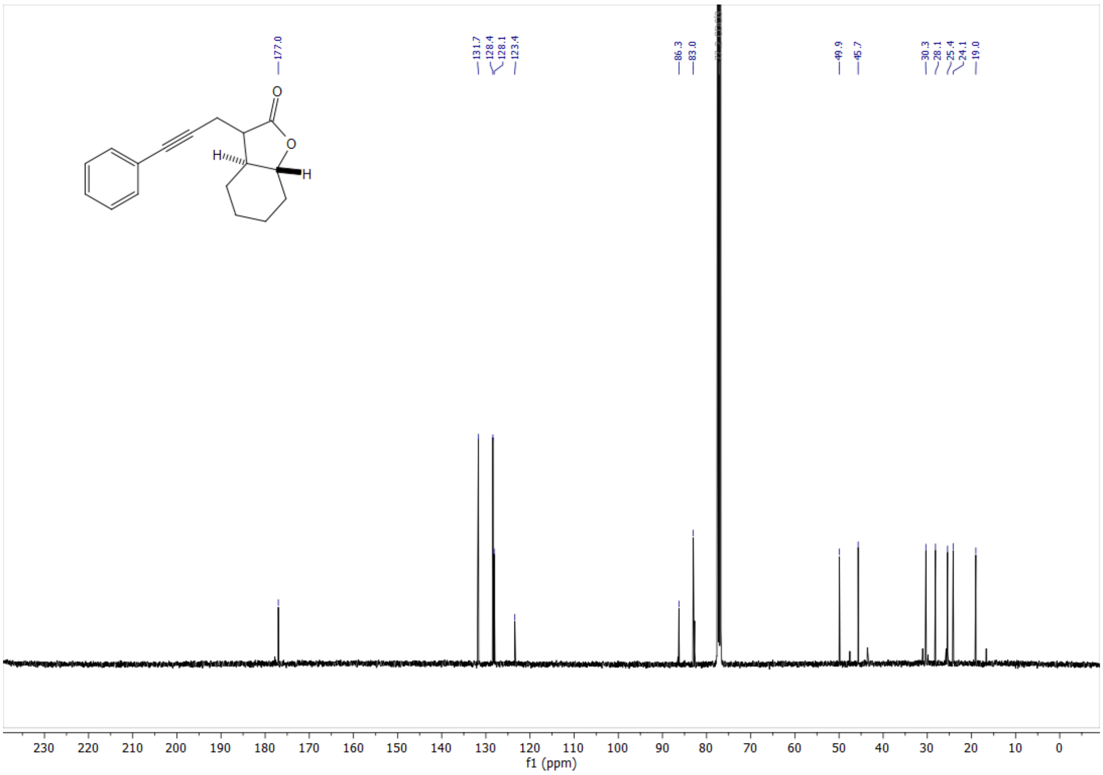

^1H NMR Spectrum (400 MHz, $CDCl_3$) of **5j**

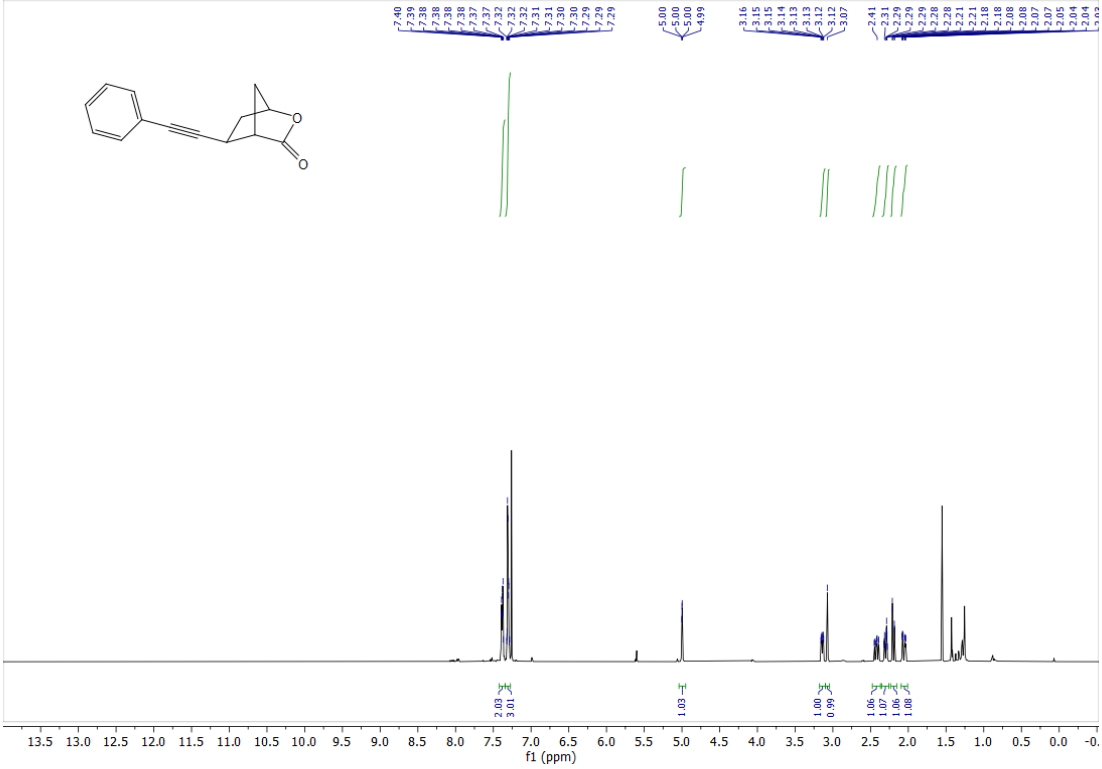

^{13}C {^1H} NMR Spectrum (101 MHz, $CDCl_3$) of **5j**

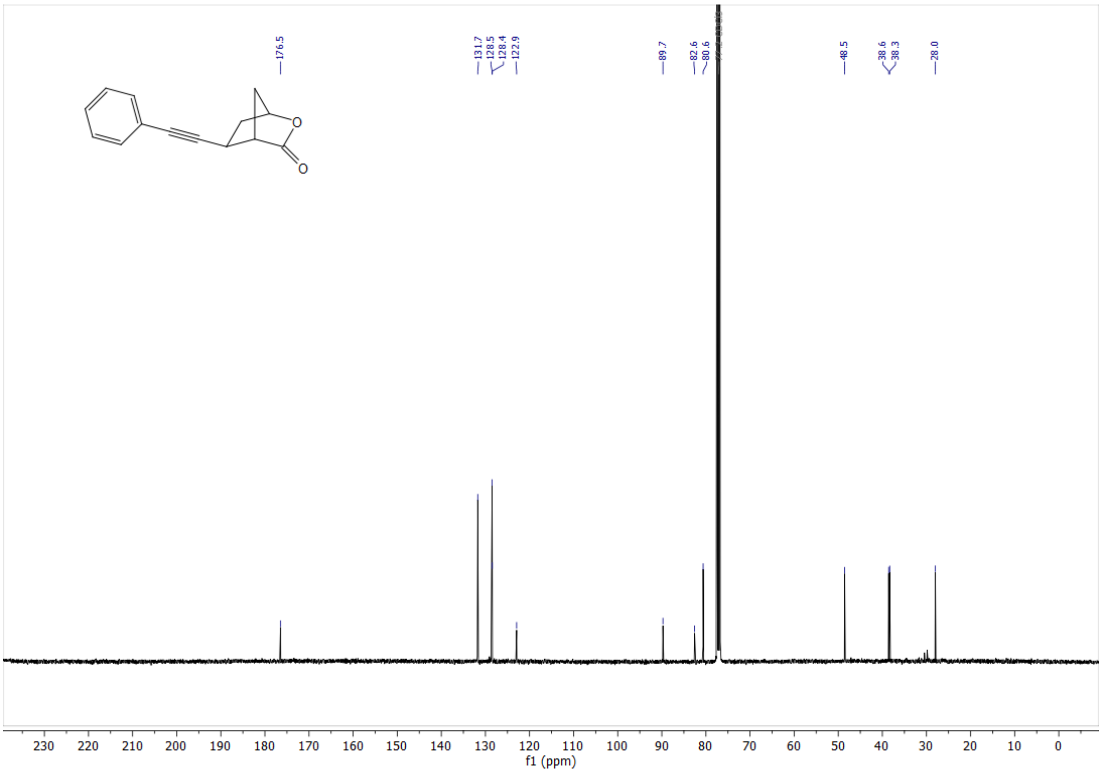

^{1}H NMR Spectrum (400 MHz, $CDCl_3$) of **5k**

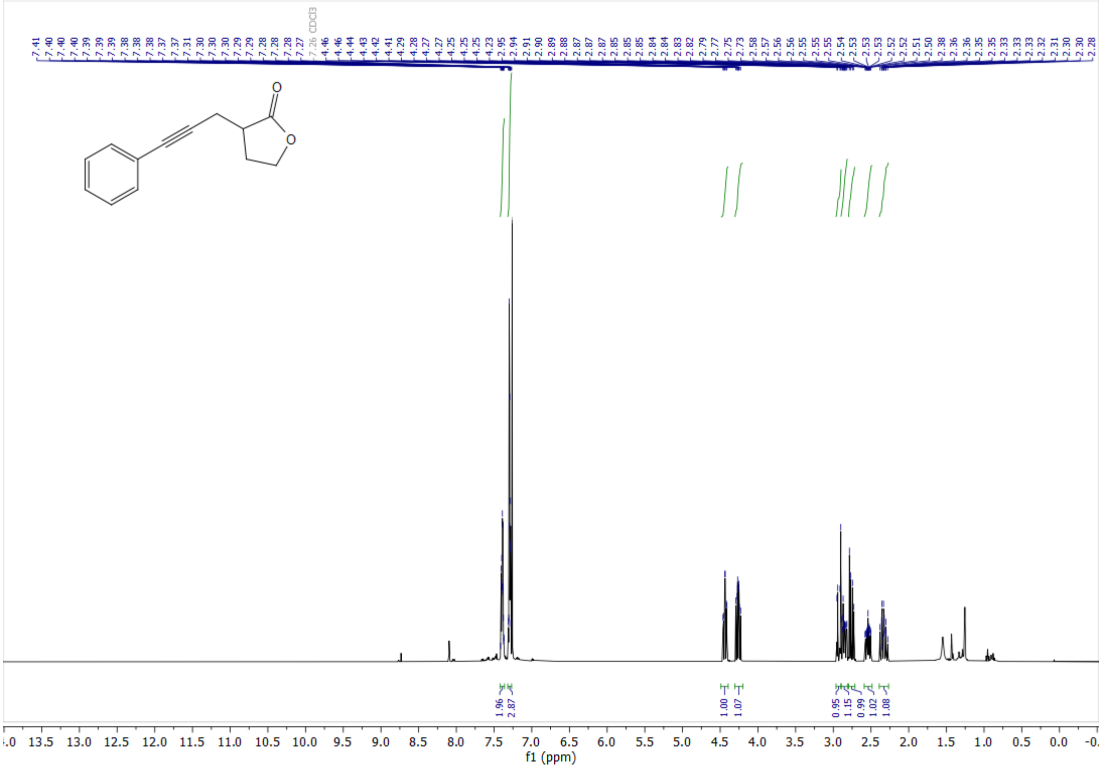

^{13}C {^{1}H} NMR Spectrum (101 MHz, $CDCl_3$) of **5k**

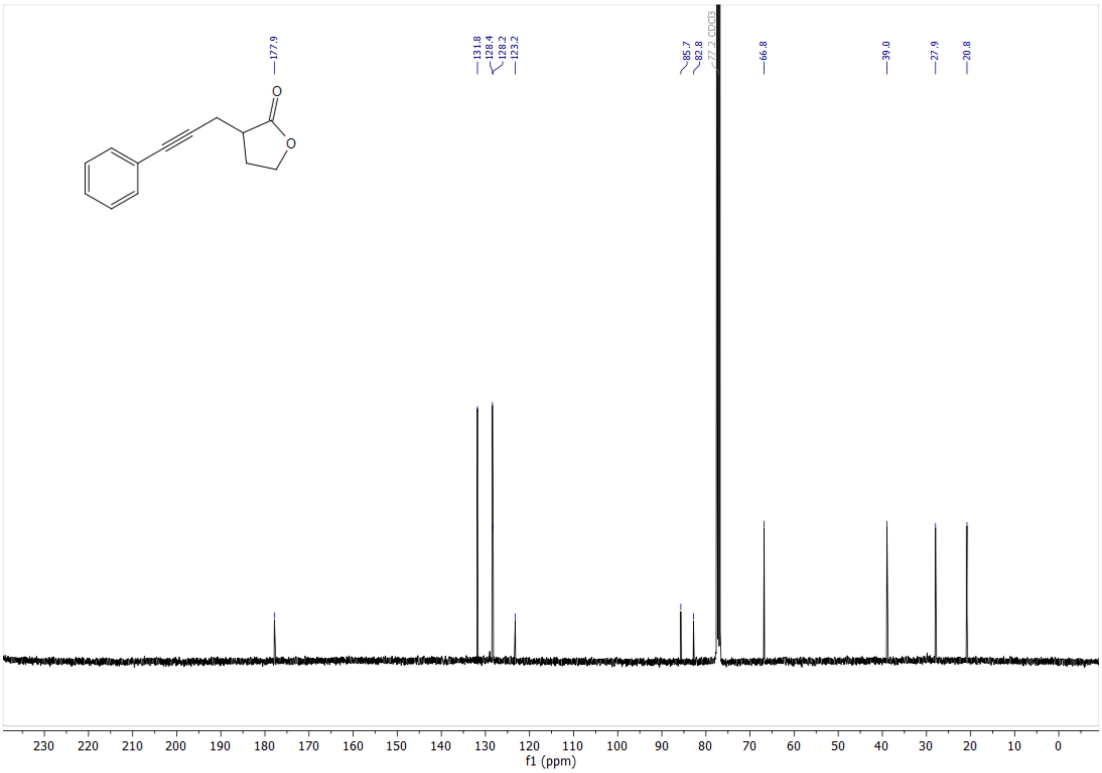

^{1}H NMR Spectrum (400 MHz, $CDCl_3$) of **5l** Major

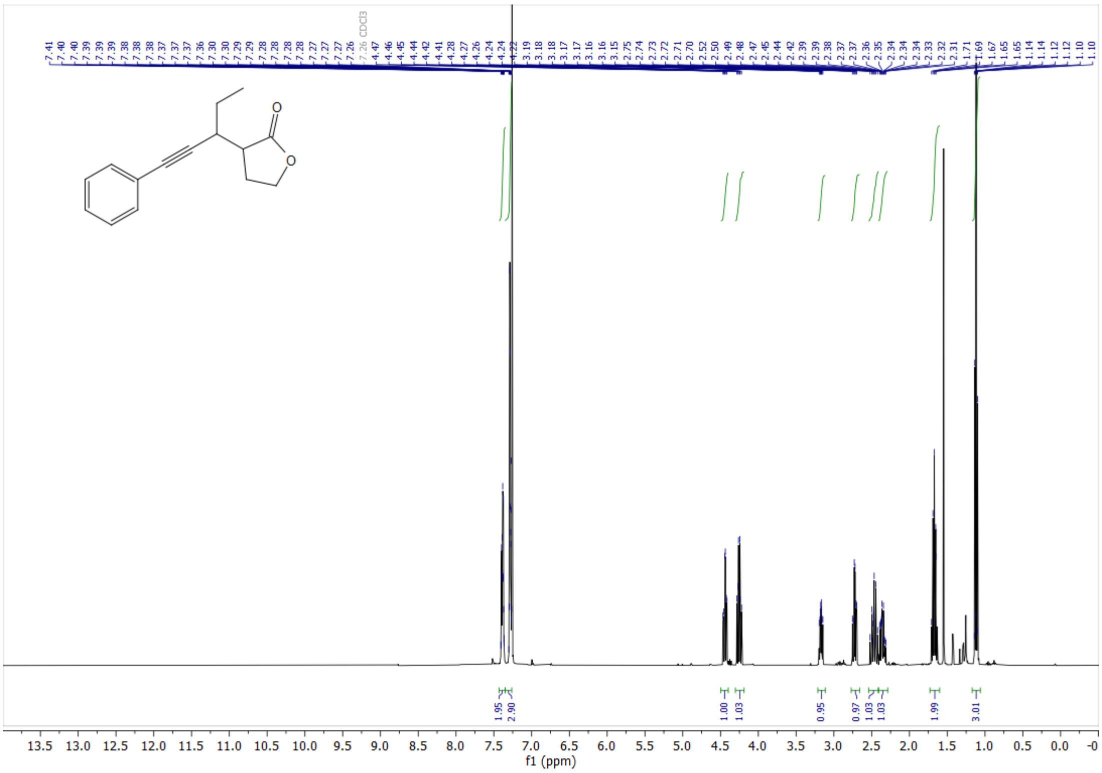

^{13}C {^{1}H} NMR Spectrum (101 MHz, $CDCl_3$) of **5l** Minor

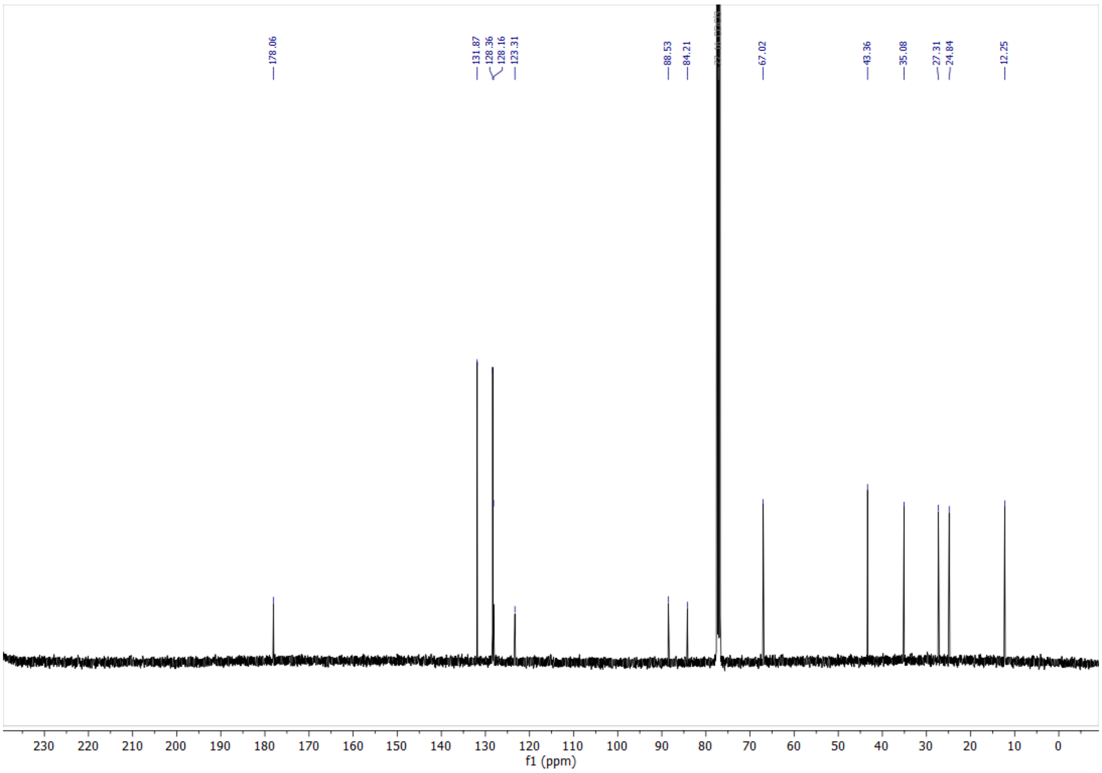

^{1}H NMR Spectrum (400 MHz, $CDCl_3$) of **5l** Minor

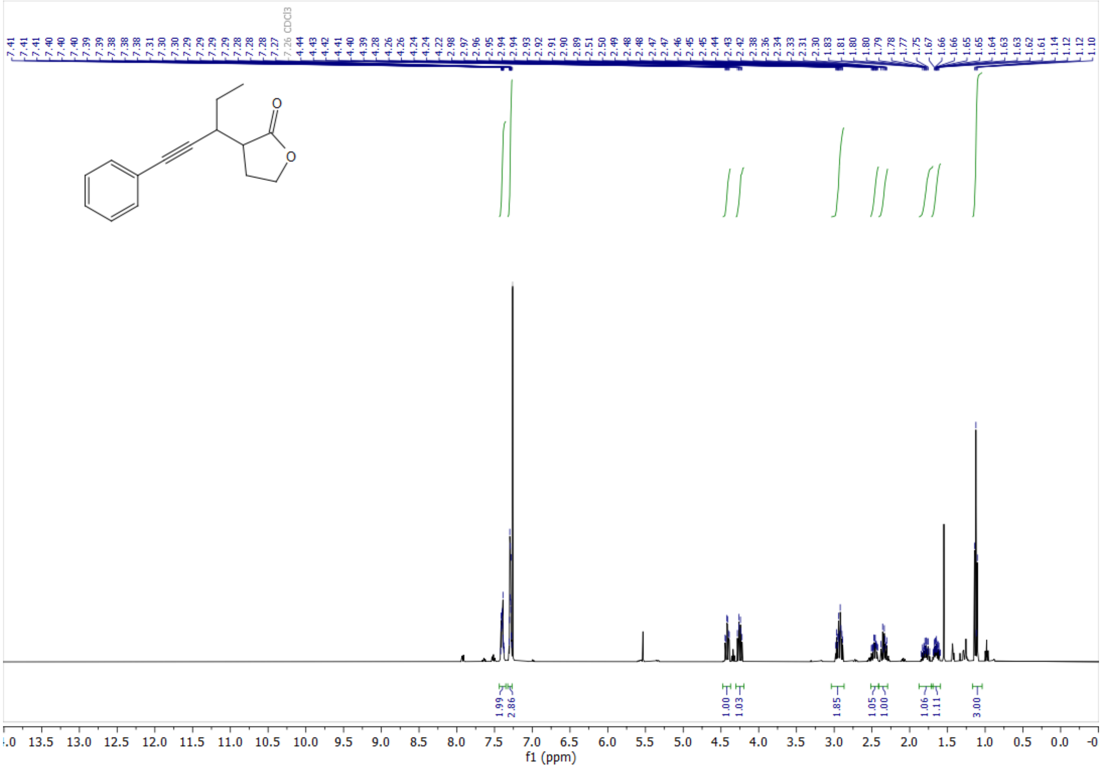

^{13}C {^{1}H} NMR Spectrum (101 MHz, $CDCl_3$) of **5l** Minor

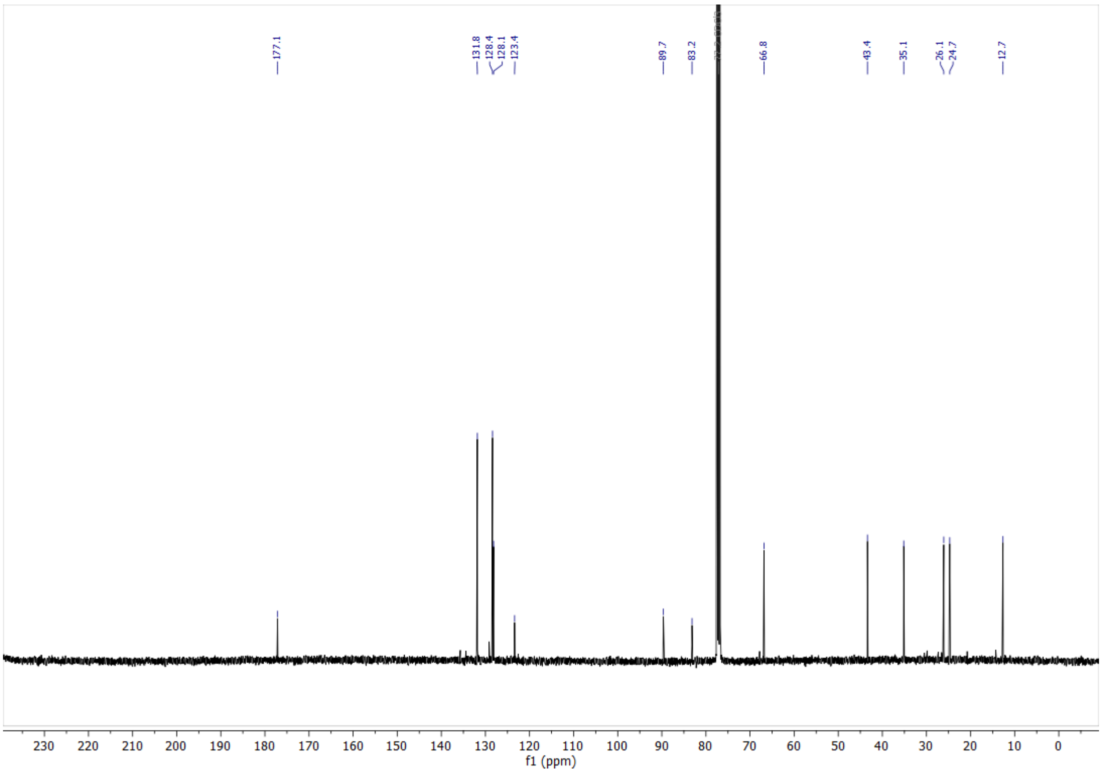

^{1}H NMR Spectrum (400 MHz, $CDCl_3$) of **5m**

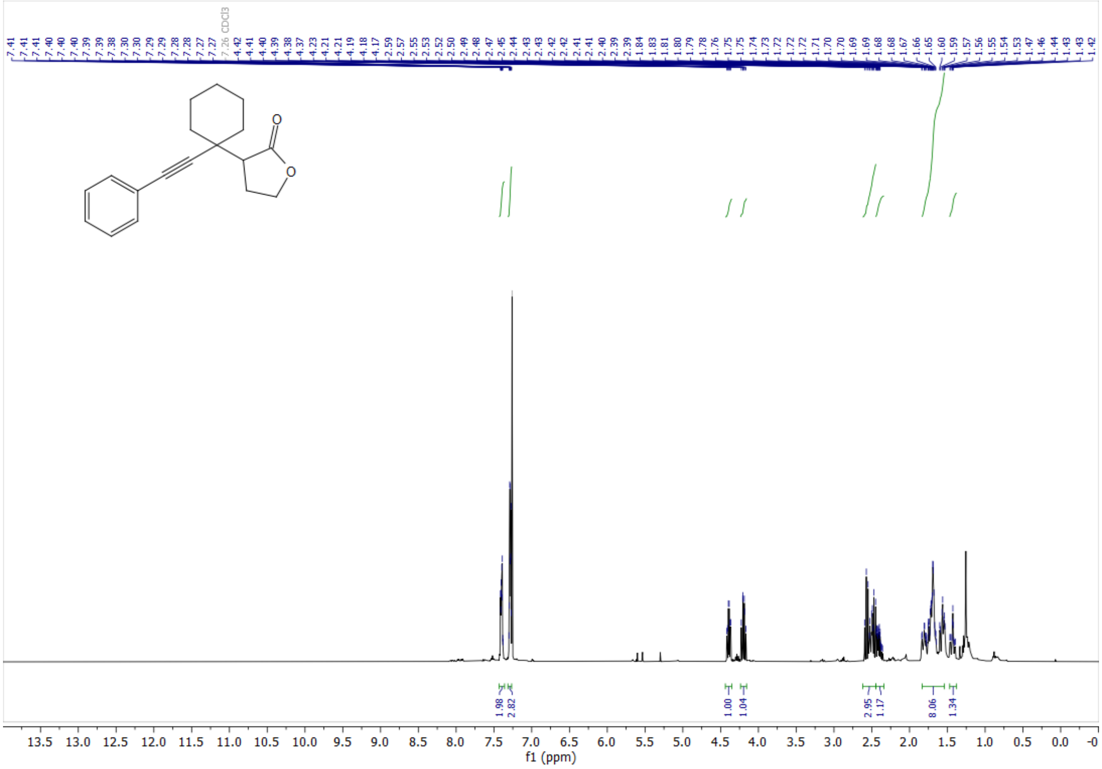

^{13}C {^{1}H} NMR Spectrum (101 MHz, $CDCl_3$) of **5m**

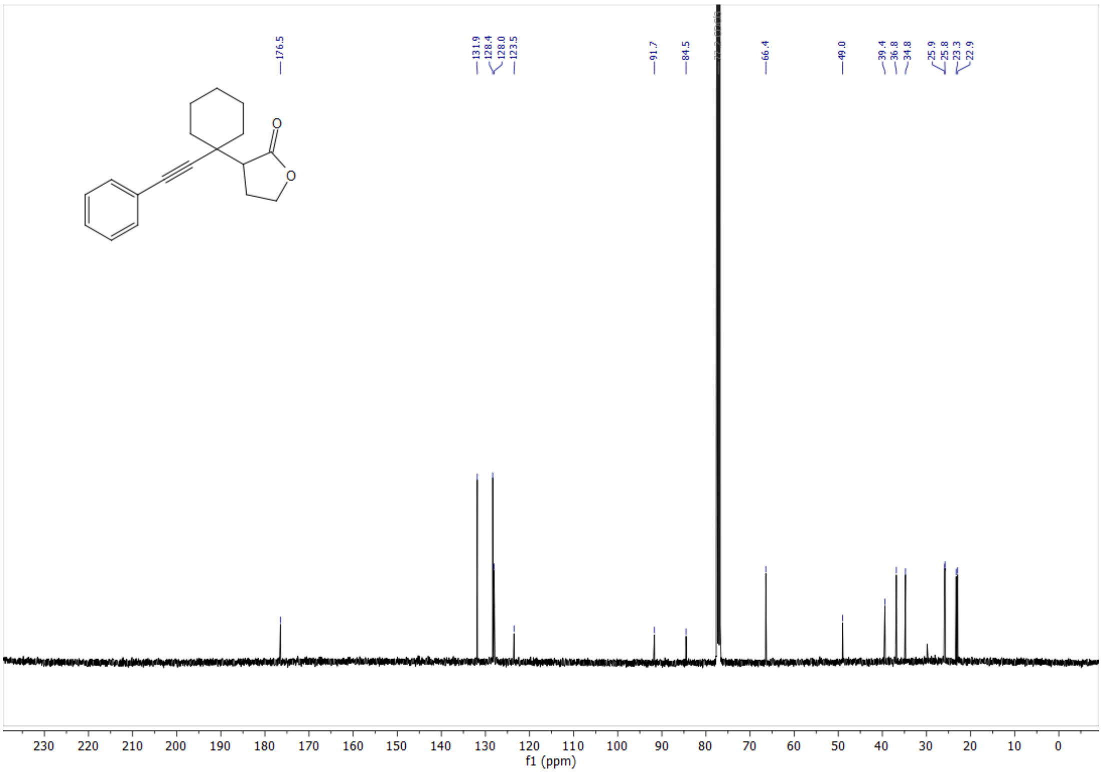

^{1}H NMR Spectrum (400 MHz, $CDCl_3$) of **5n**

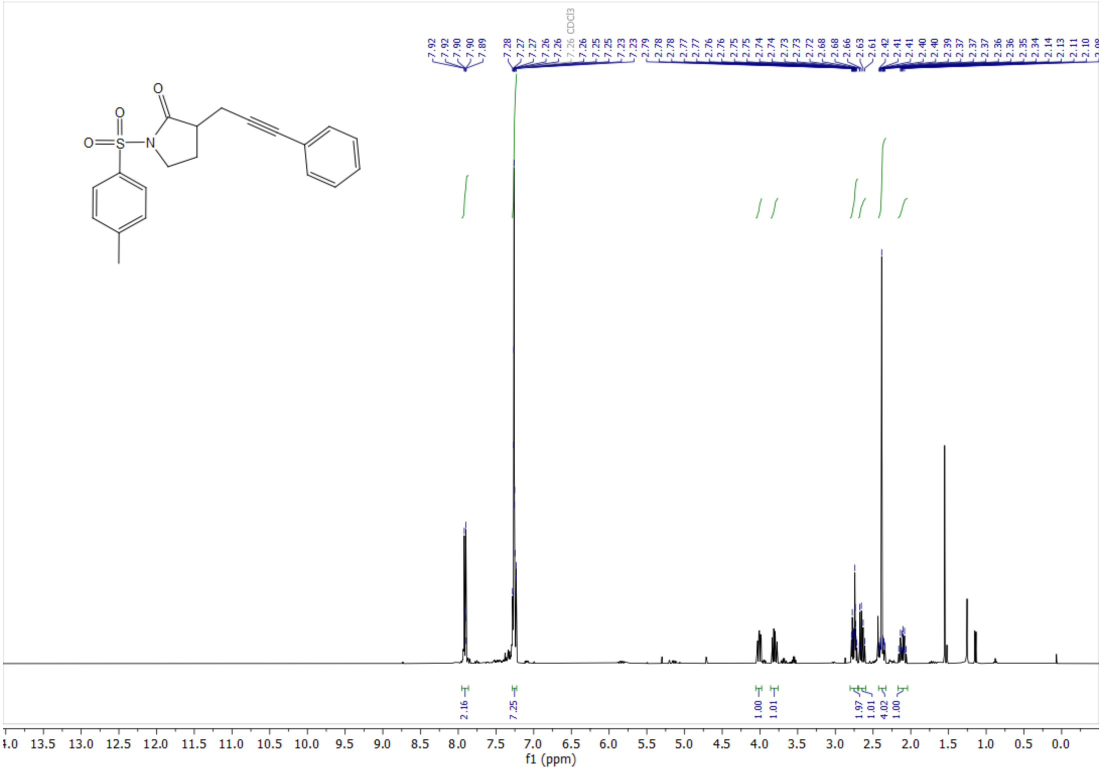

^{13}C {^{1}H} NMR Spectrum (101 MHz, $CDCl_3$) of **5n**

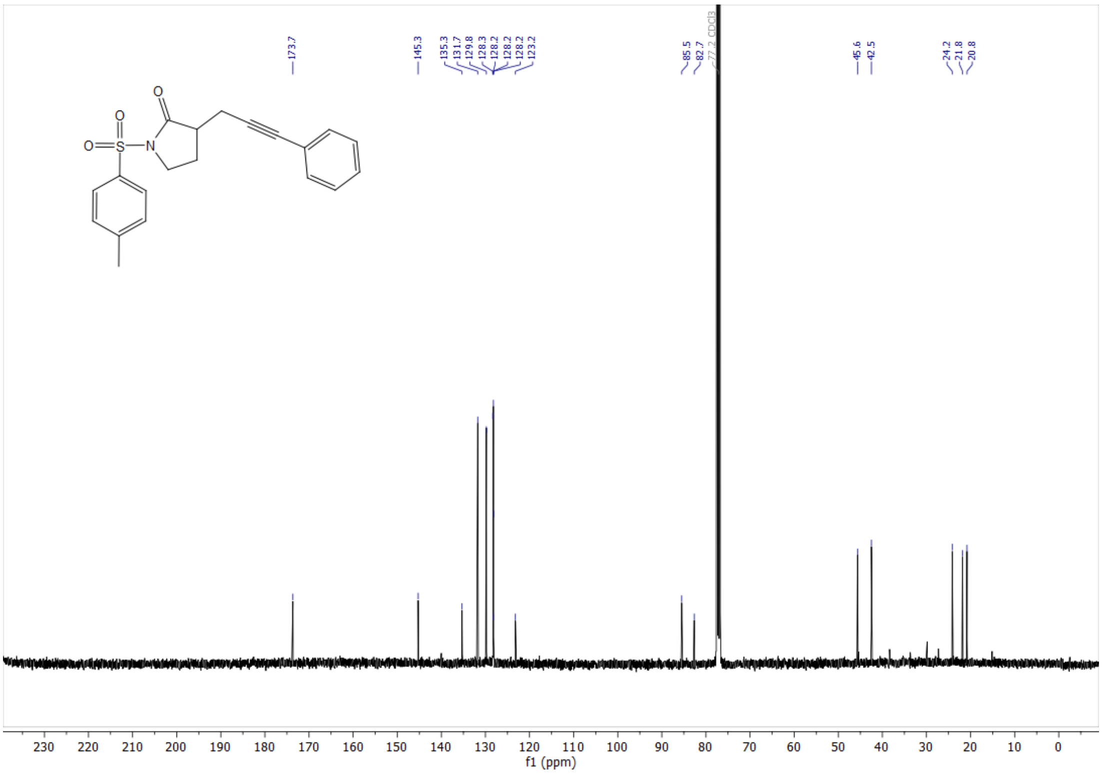

^{1}H NMR Spectrum (400 MHz, $CDCl_3$) of **5o** Major

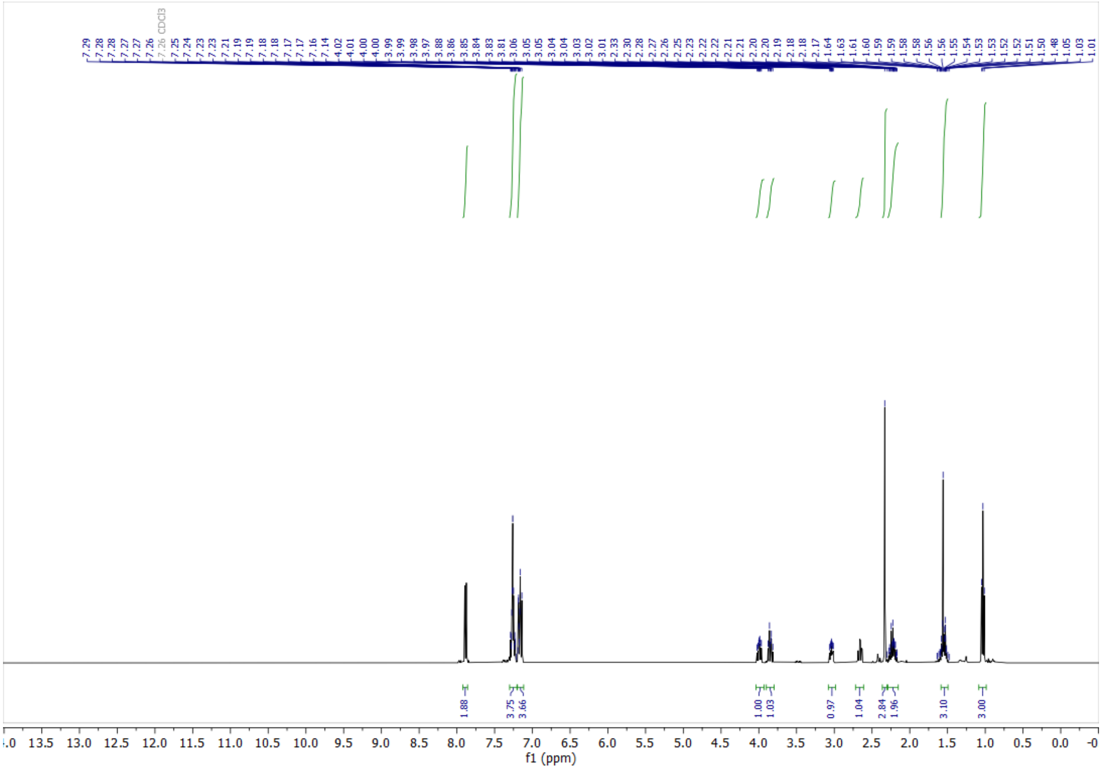

^{13}C {^{1}H} NMR Spectrum (101 MHz, $CDCl_3$) of **5o** Major

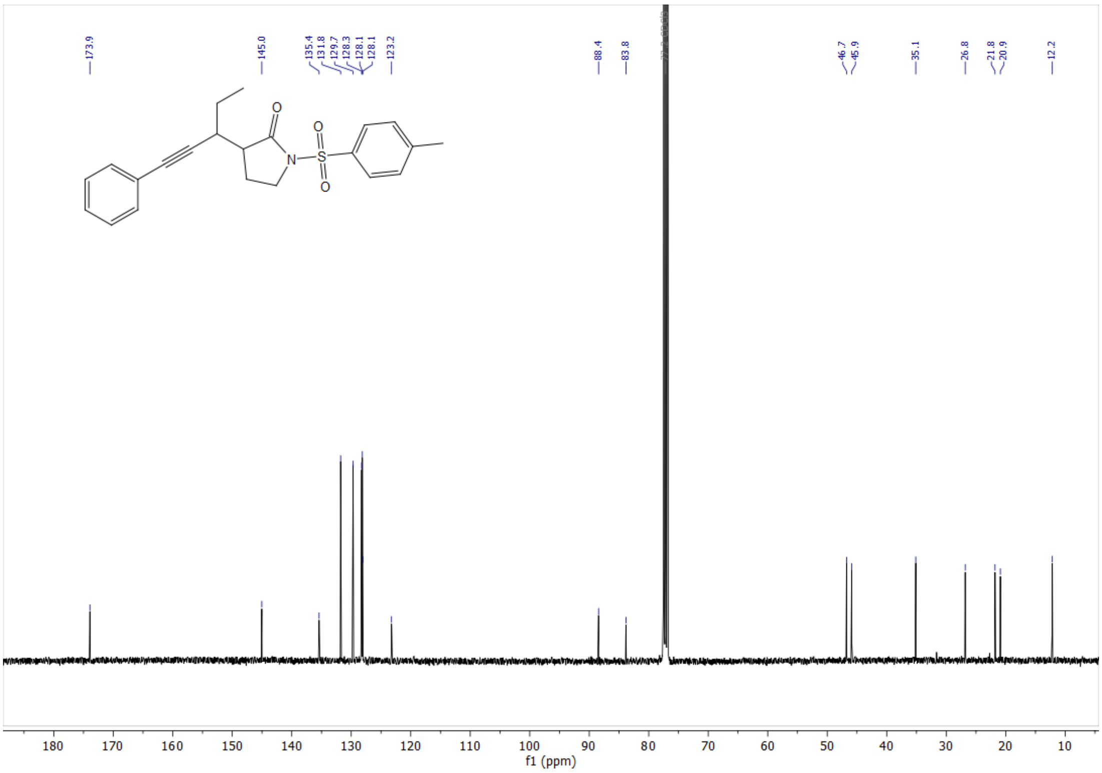

^{1}H NMR Spectrum (400 MHz, $CDCl_3$) of **5o** Minor

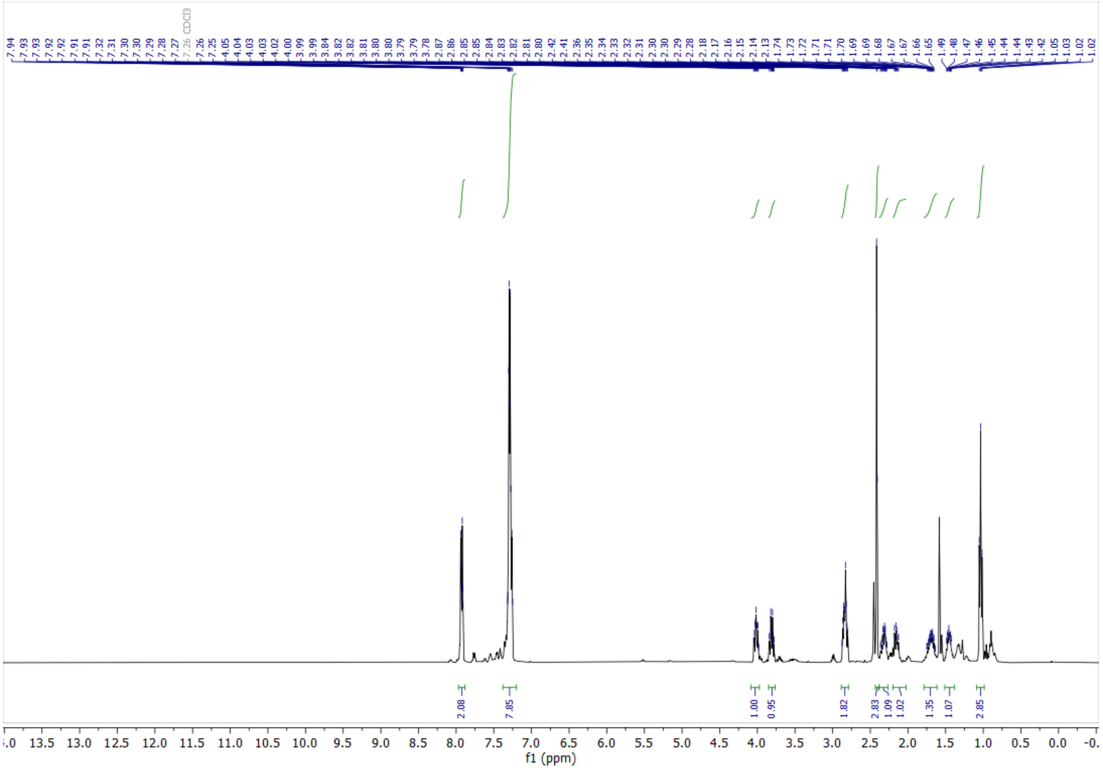

^{13}C {^{1}H} NMR Spectrum (101 MHz, $CDCl_3$) of **5o** Minor

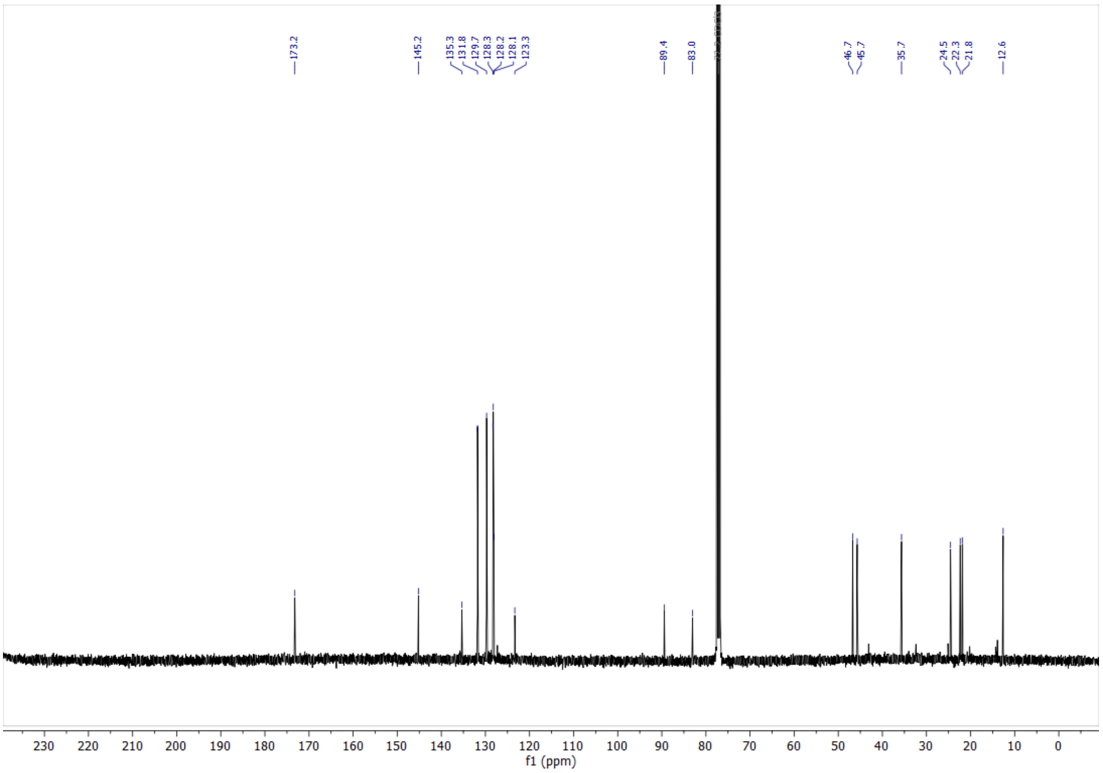

^{1}H NMR Spectrum (400 MHz, $CDCl_3$) of **5p**

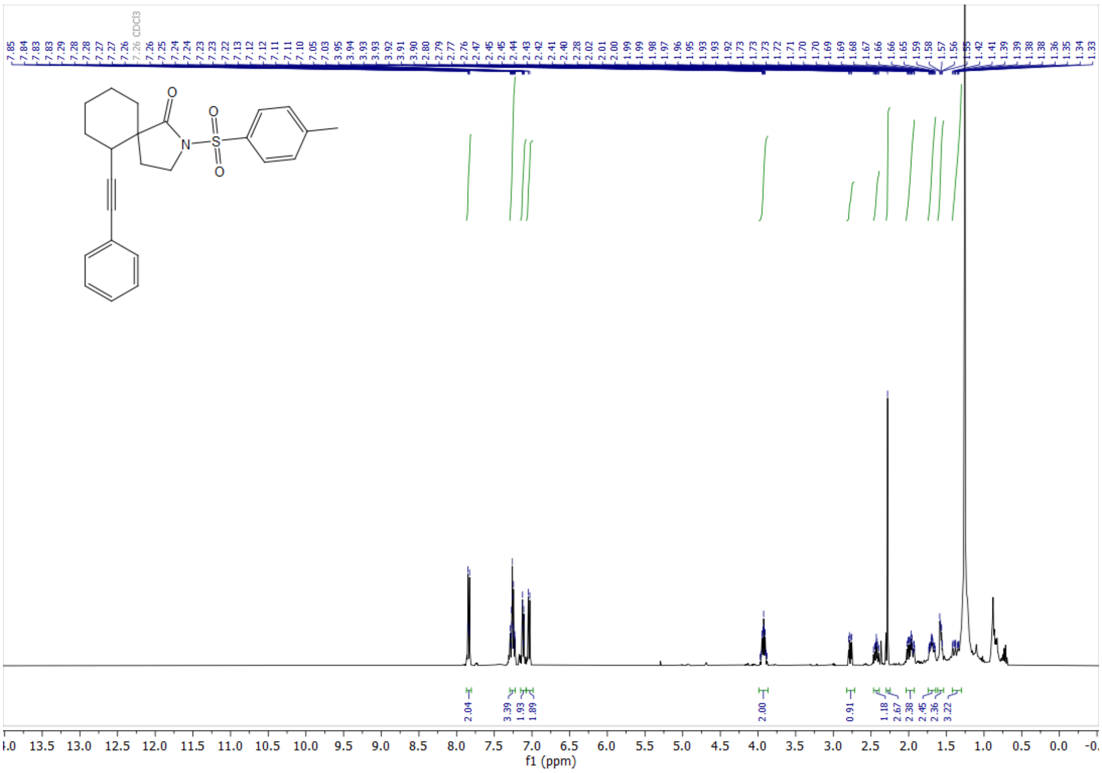

^{13}C {^{1}H} NMR Spectrum (101 MHz, $CDCl_3$) of **5p**

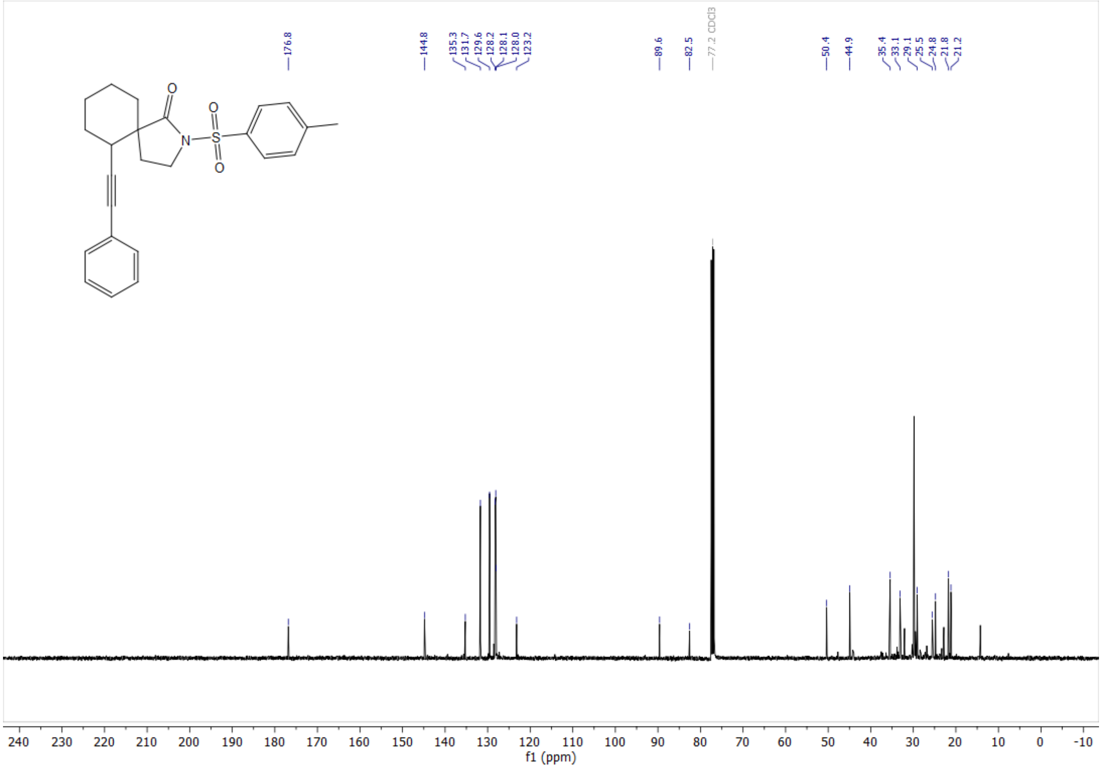

^{1}H NMR Spectrum (400 MHz, $CDCl_3$) of **5q**

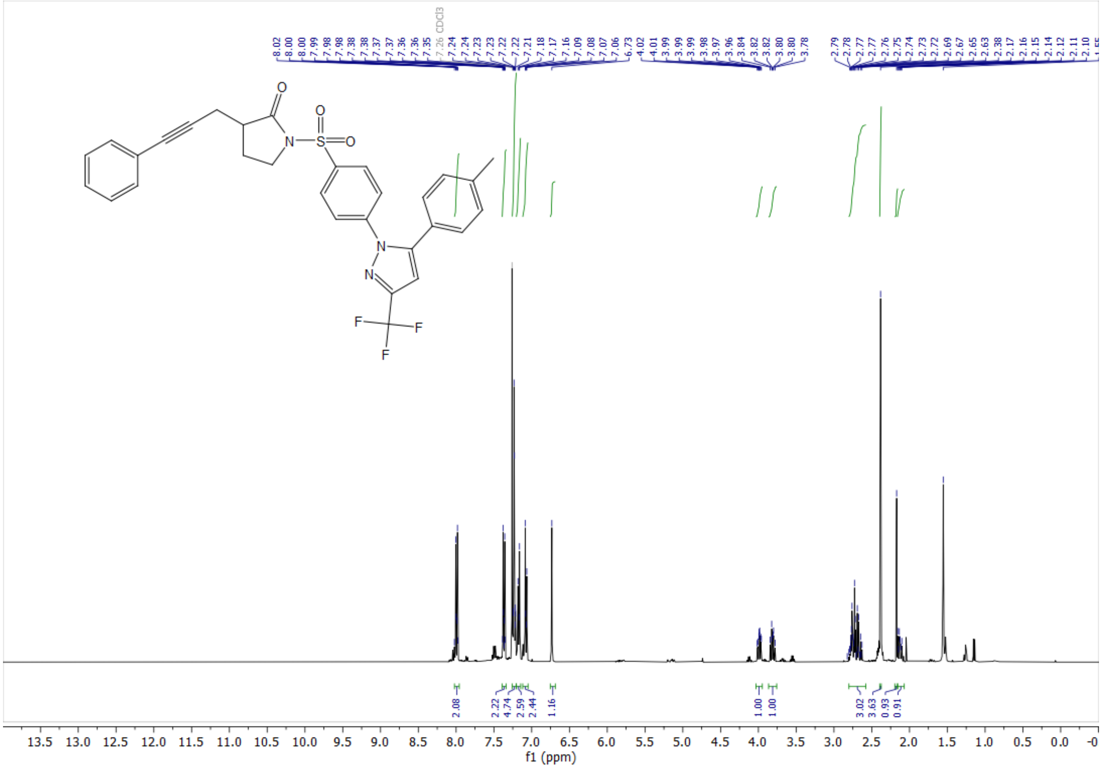

^{13}C {^{1}H} NMR Spectrum (101 MHz, $CDCl_3$) of **5q**

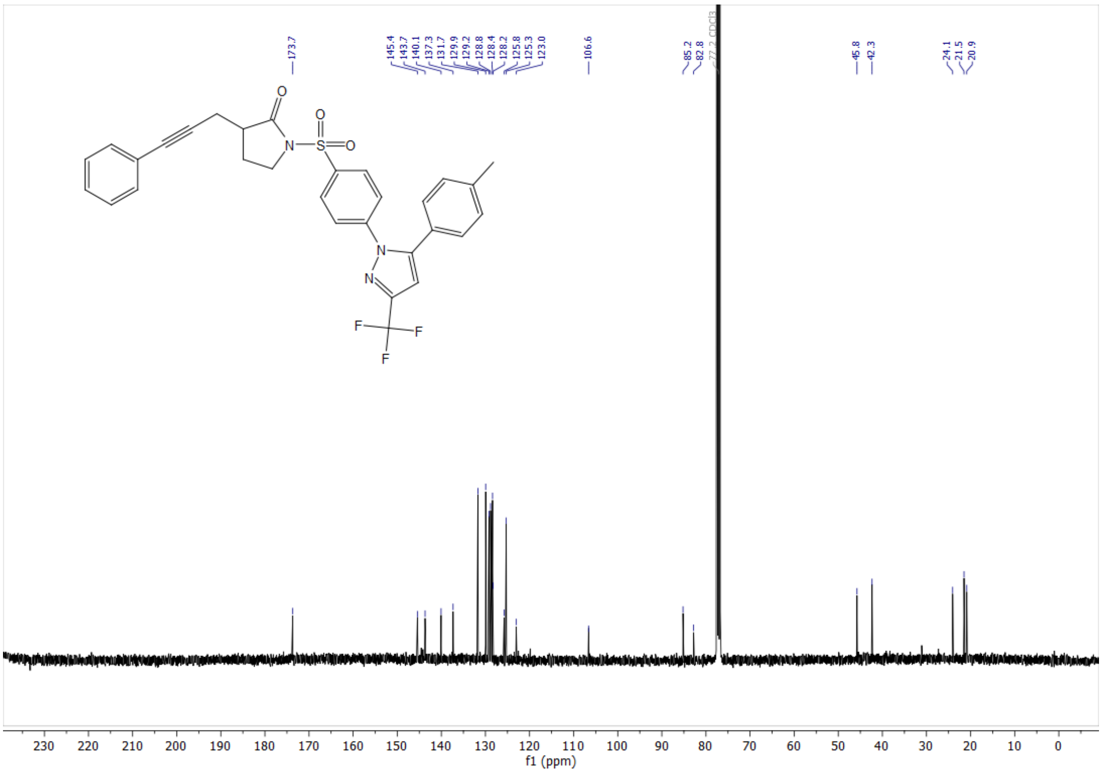

^{19}F {^{1}H} NMR Spectrum (376 MHz, $CDCl_3$) of **5q**

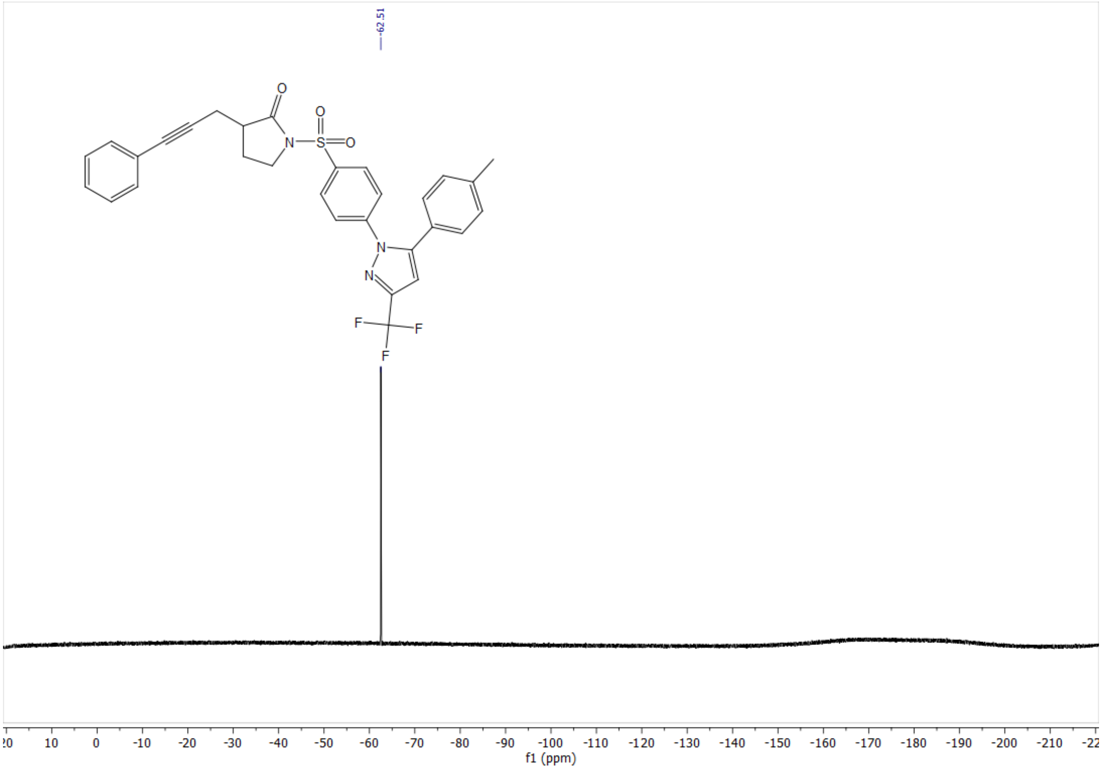

^{1}H NMR Spectrum (400 MHz, $CDCl_3$) of **5r**

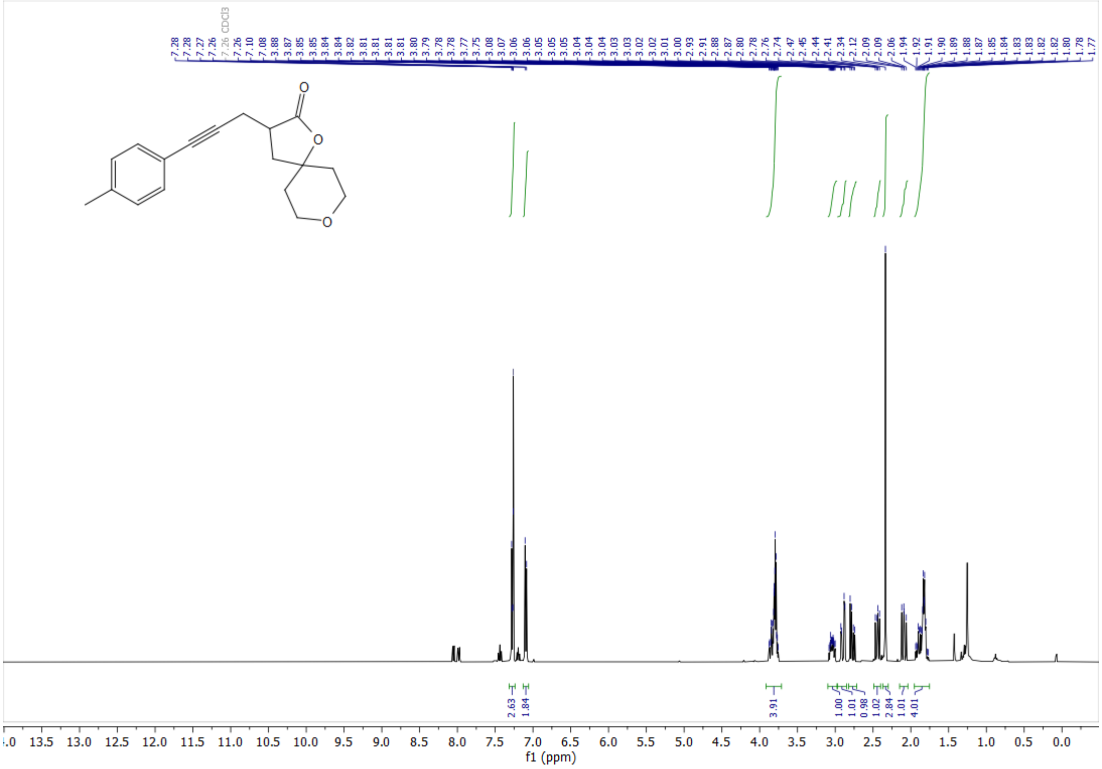

^{13}C {^1H} NMR Spectrum (101 MHz, $CDCl_3$) of **5r**

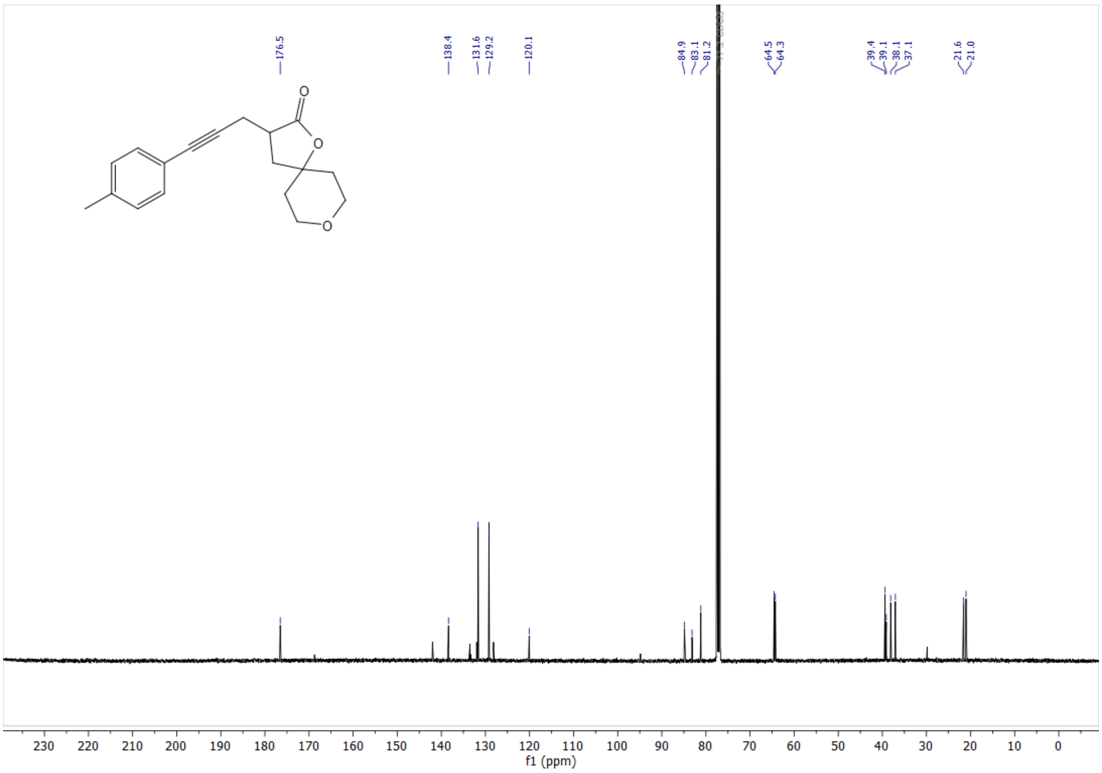

^1H NMR Spectrum (400 MHz, $CDCl_3$) of **5s**

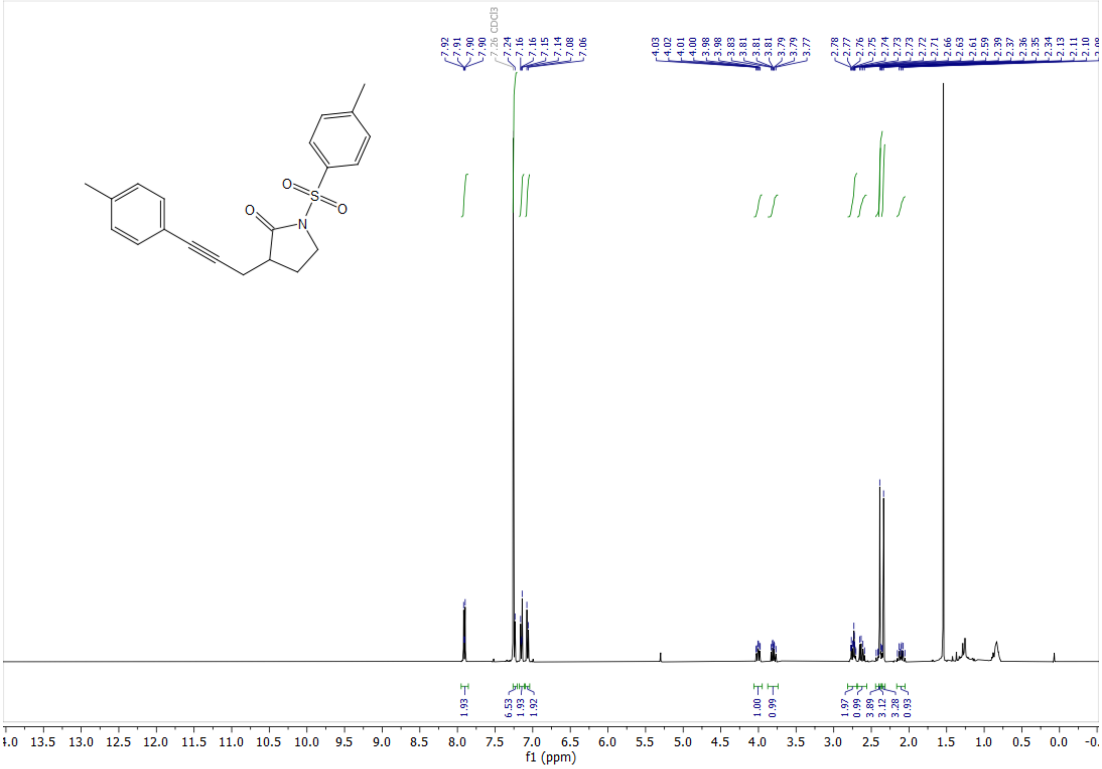

$^{13}C\ \{^1H\}$ NMR Spectrum (101 MHz, $CDCl_3$) of **5s**

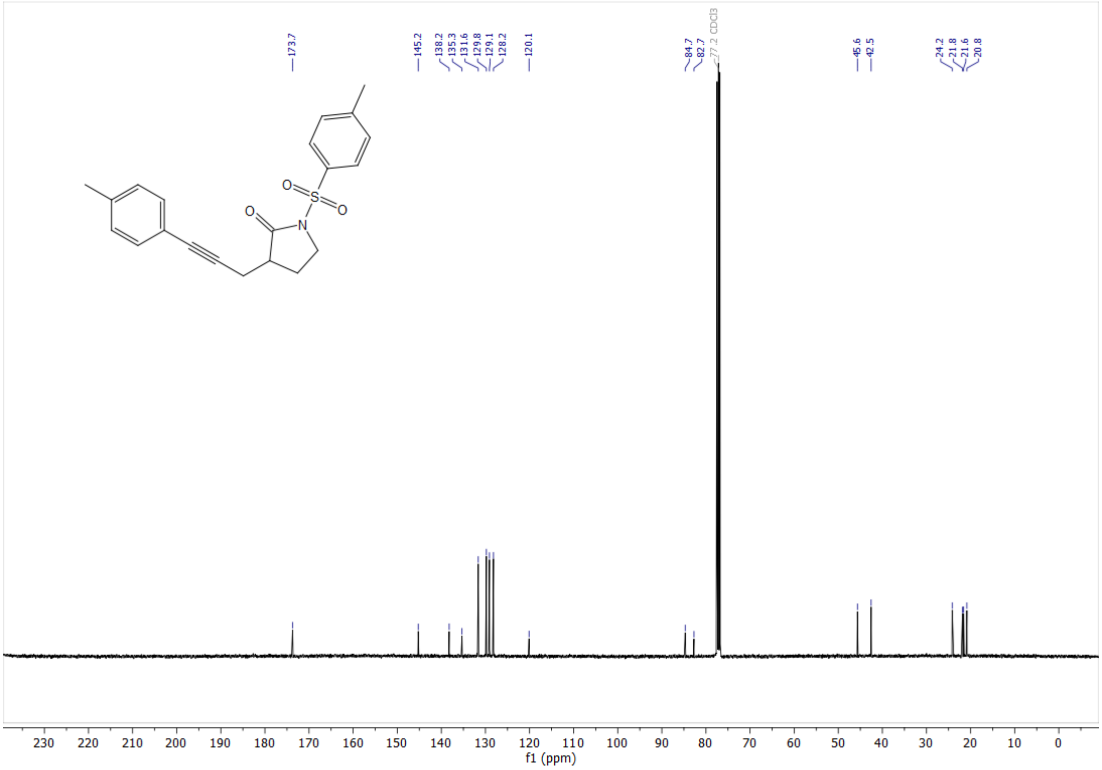

^1H NMR Spectrum (400 MHz, $CDCl_3$) of **5t**

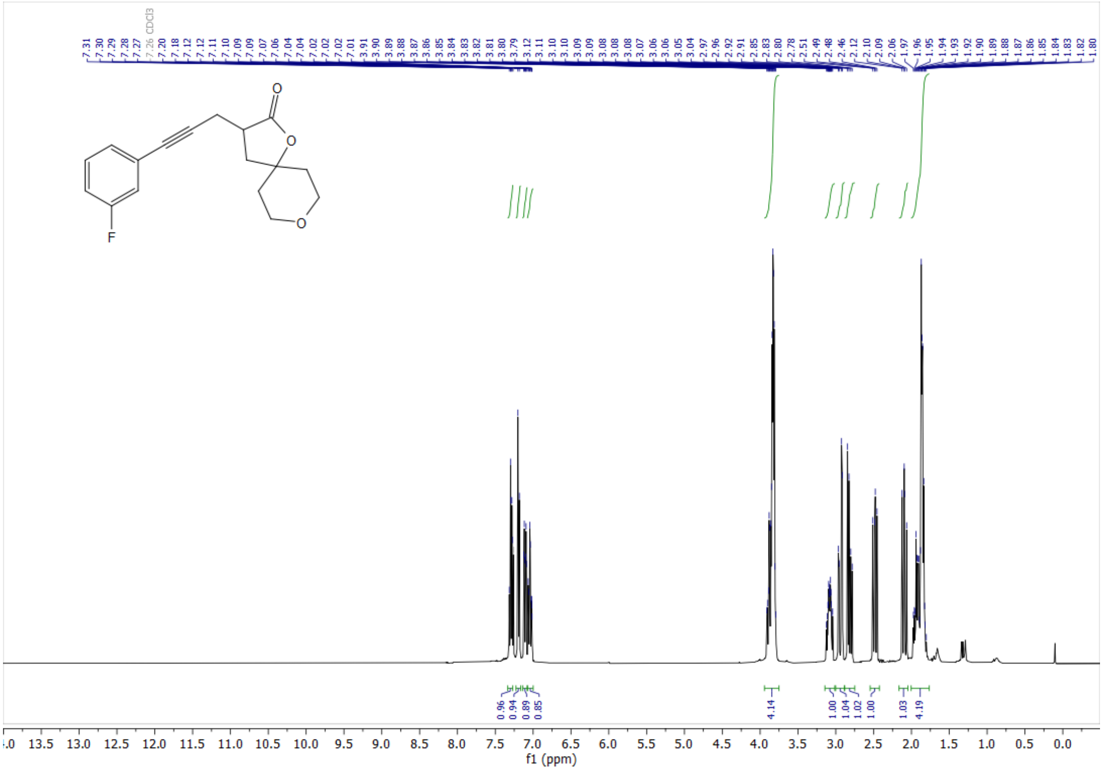

^{13}C {^{1}H} NMR Spectrum (101 MHz, $CDCl_3$) of **5t**

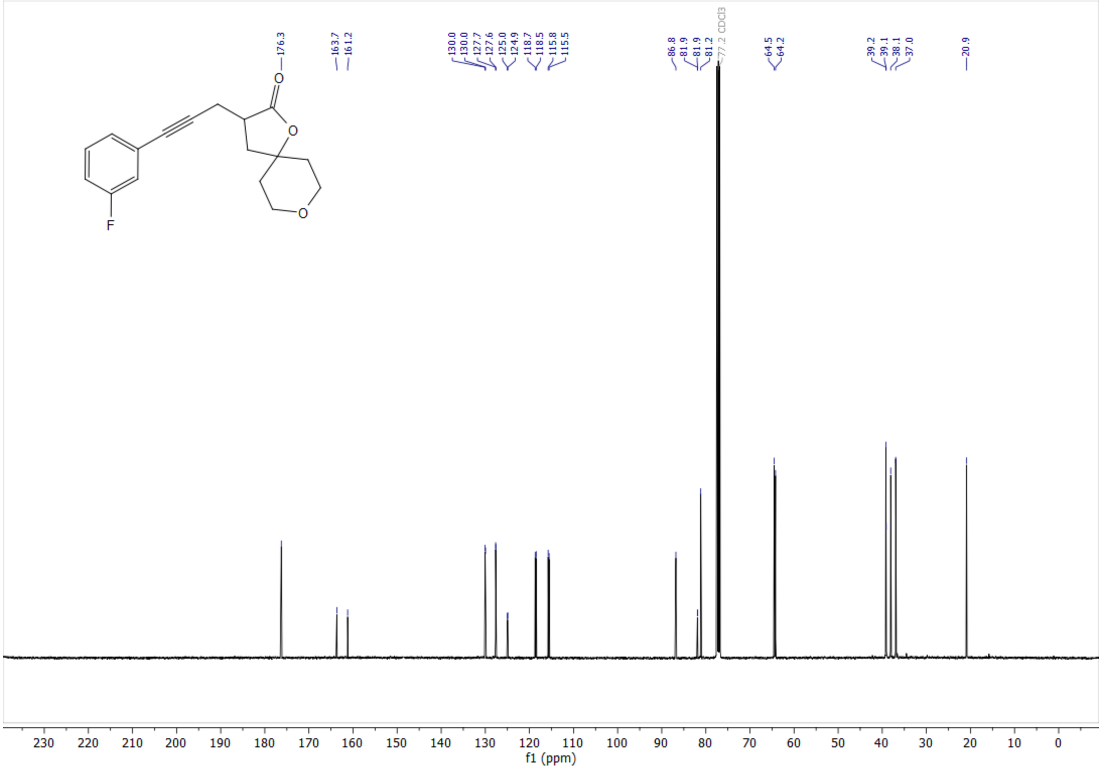

^{19}F {^{1}H} NMR Spectrum (376 MHz, $CDCl_3$) of **5t**

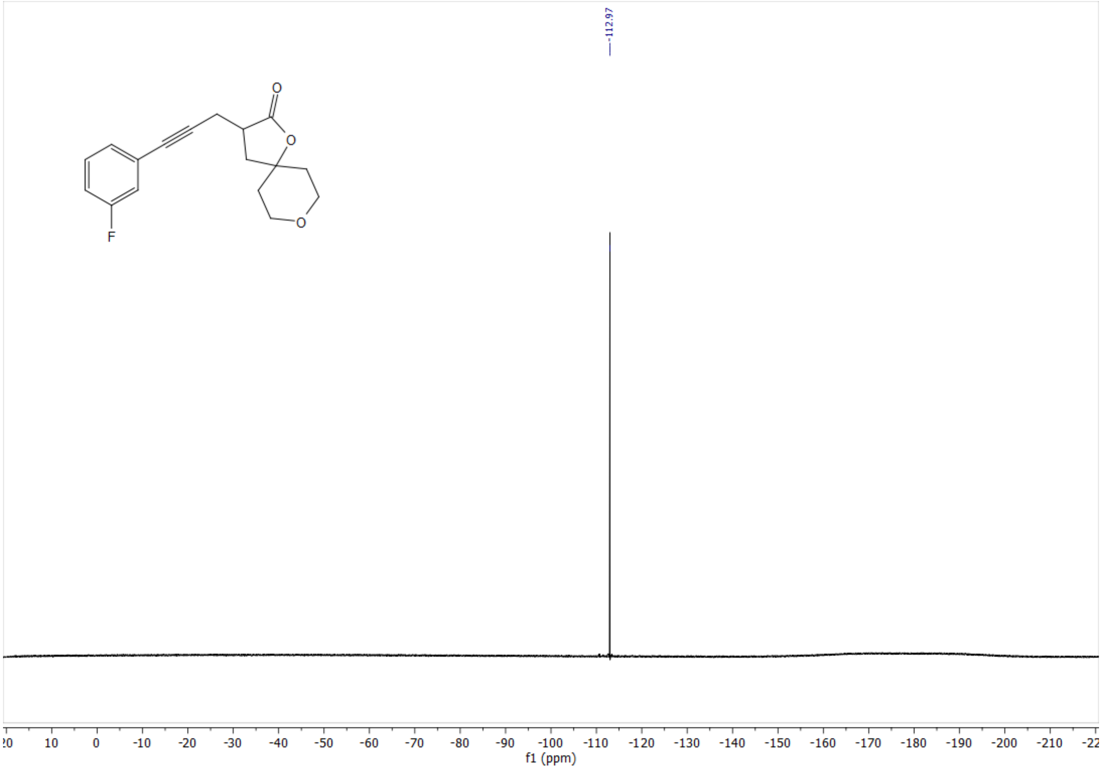

^{1}H NMR Spectrum (400 MHz, $CDCl_3$) of **5u**

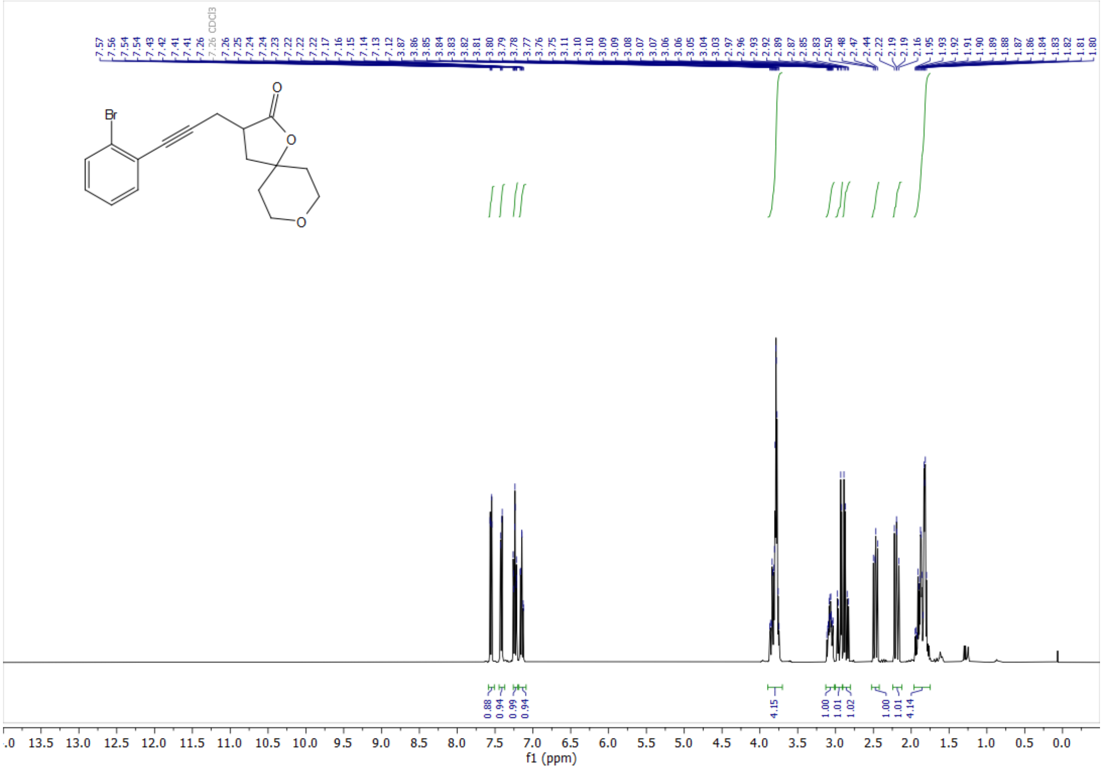

^{13}C {^{1}H} NMR Spectrum (101 MHz, $CDCl_3$) of **5u**

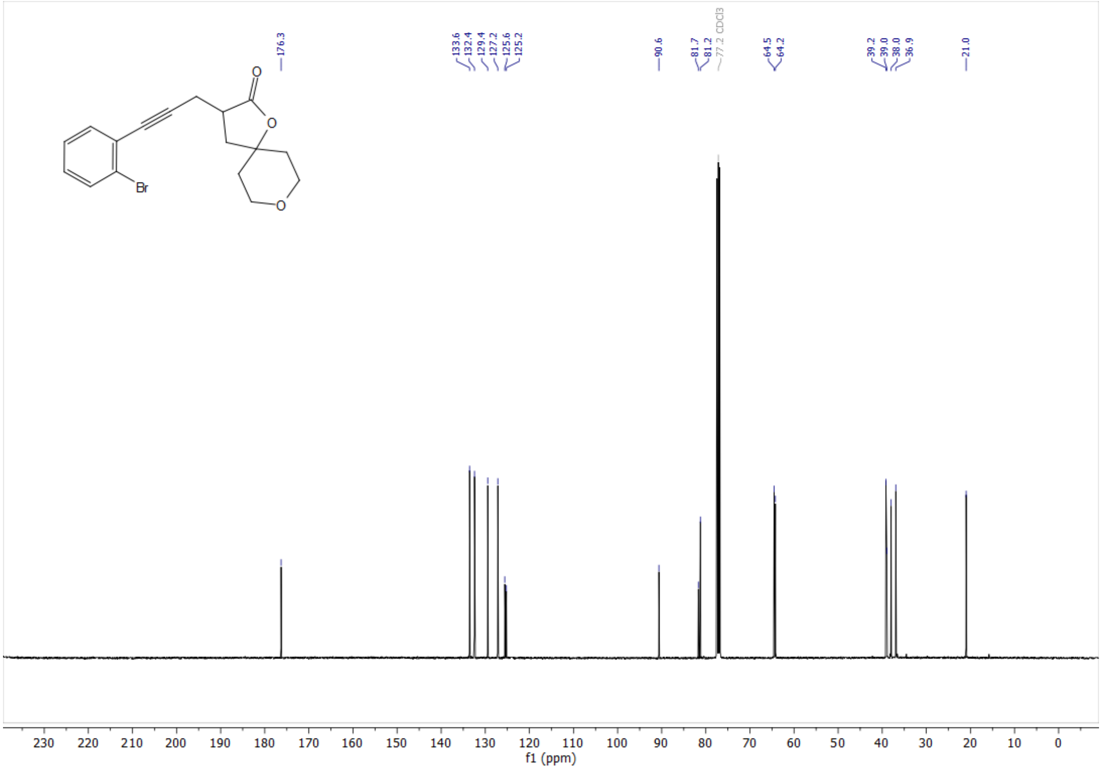

^1H NMR Spectrum (400 MHz, $CDCl_3$) of **5v**

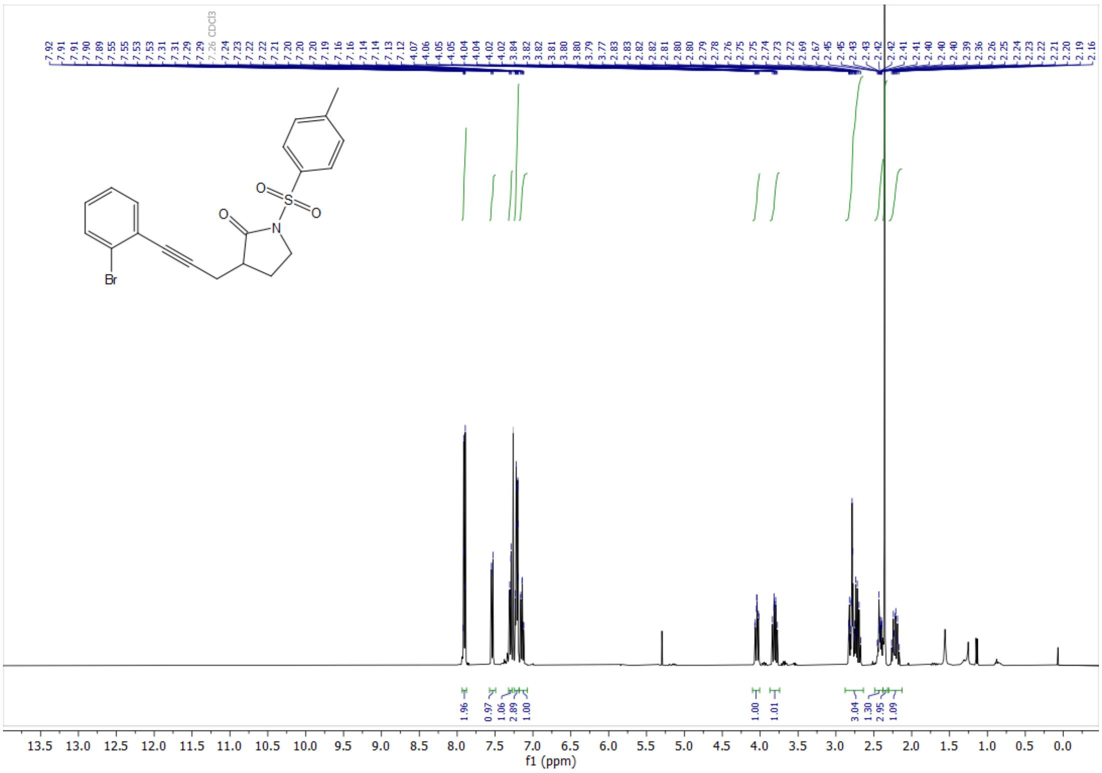

^{13}C {^1H} NMR Spectrum (101 MHz, $CDCl_3$) of **5v**

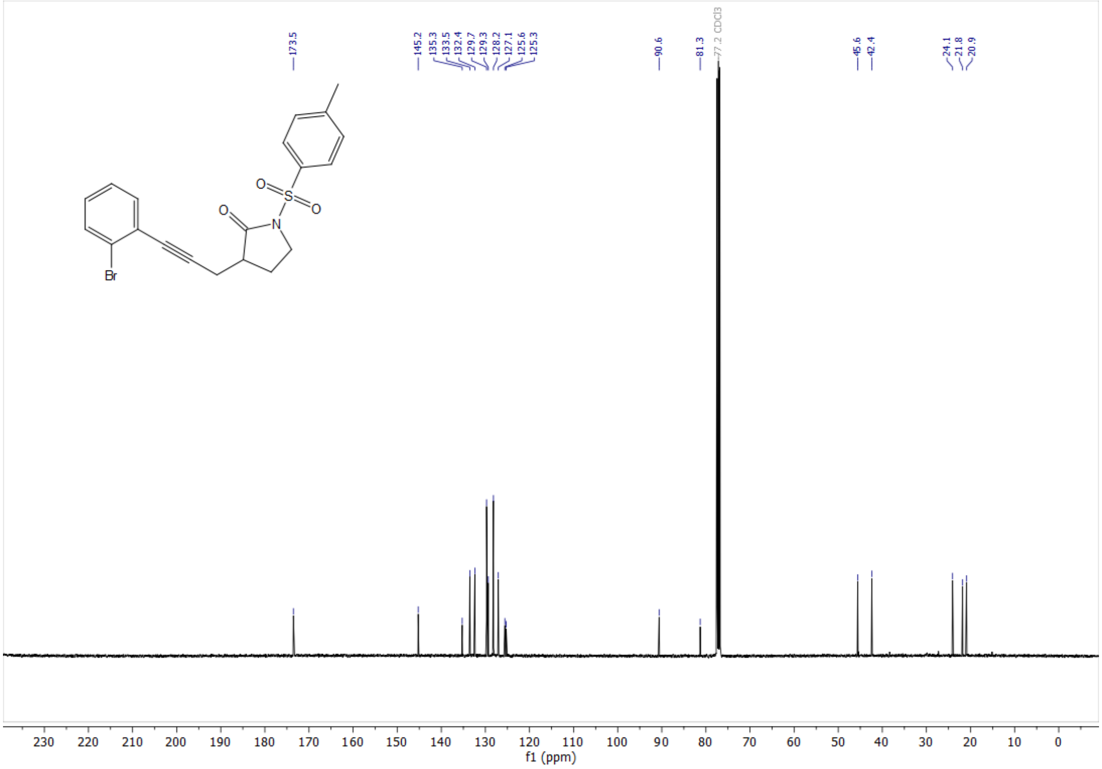

^{1}H NMR Spectrum (400 MHz, $CDCl_3$) of **5w**

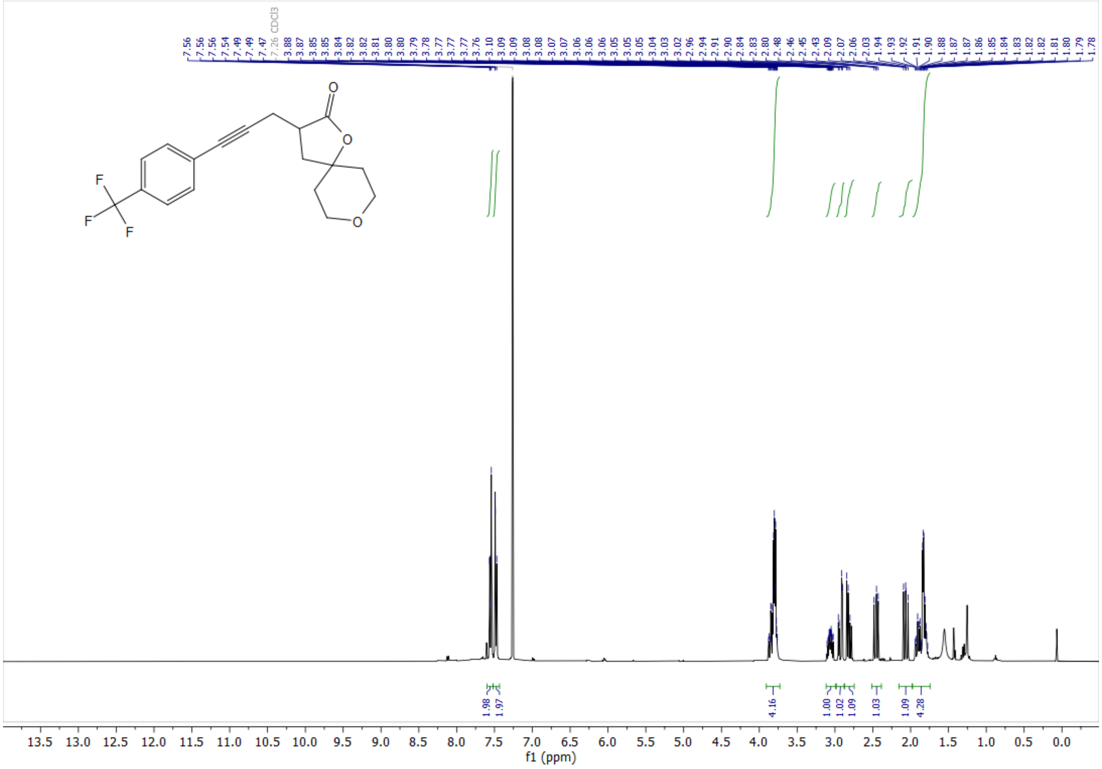

^{13}C {^{1}H} NMR Spectrum (101 MHz, $CDCl_3$) of **5w**

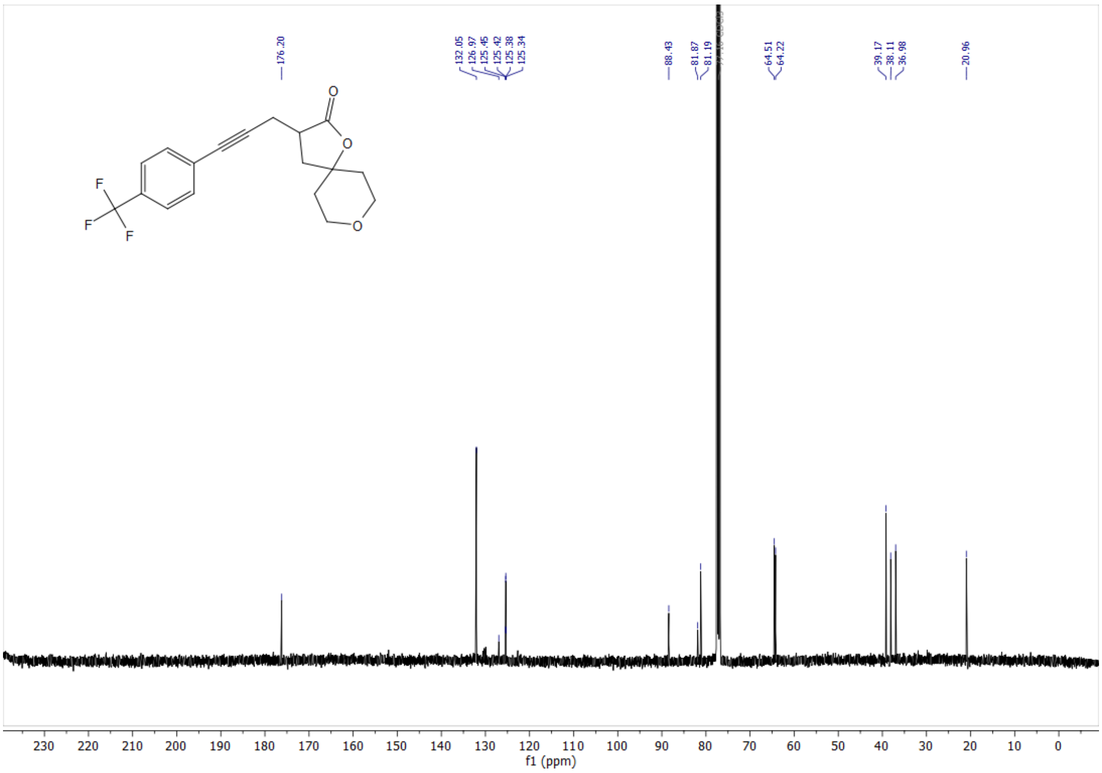

^{19}F {^{1}H} NMR Spectrum (376 MHz, $CDCl_3$) of **5w**

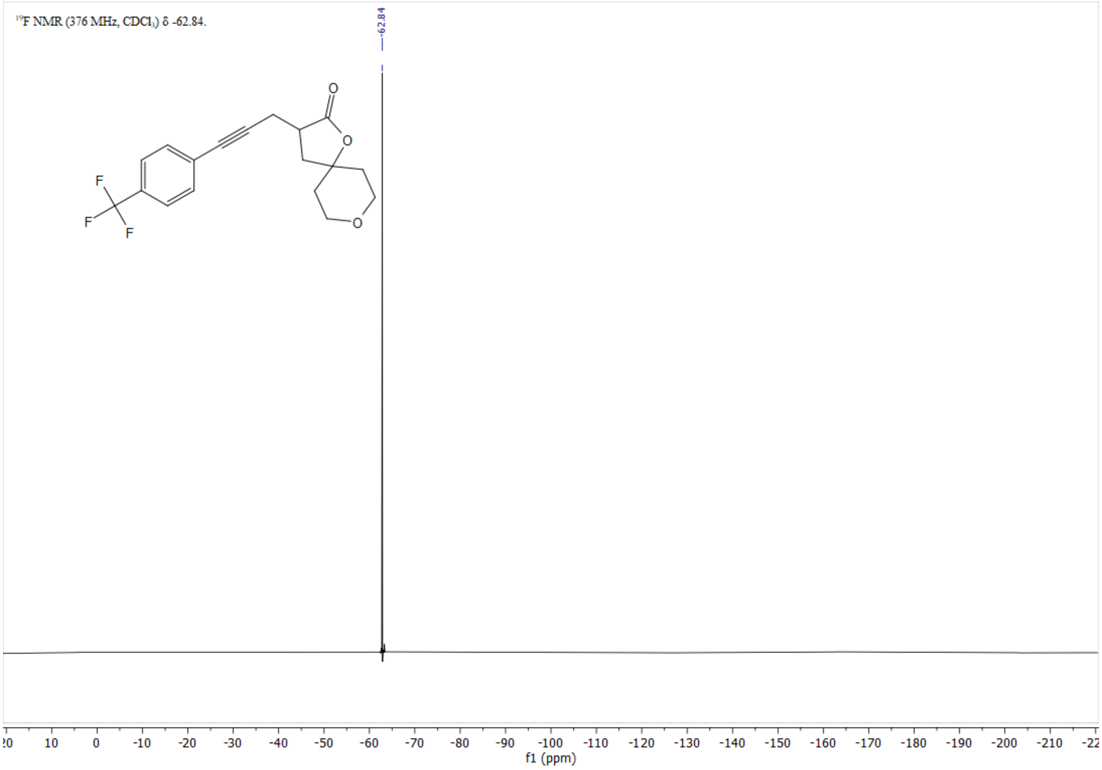

^{1}H NMR Spectrum (400 MHz, $CDCl_3$) of **5x**

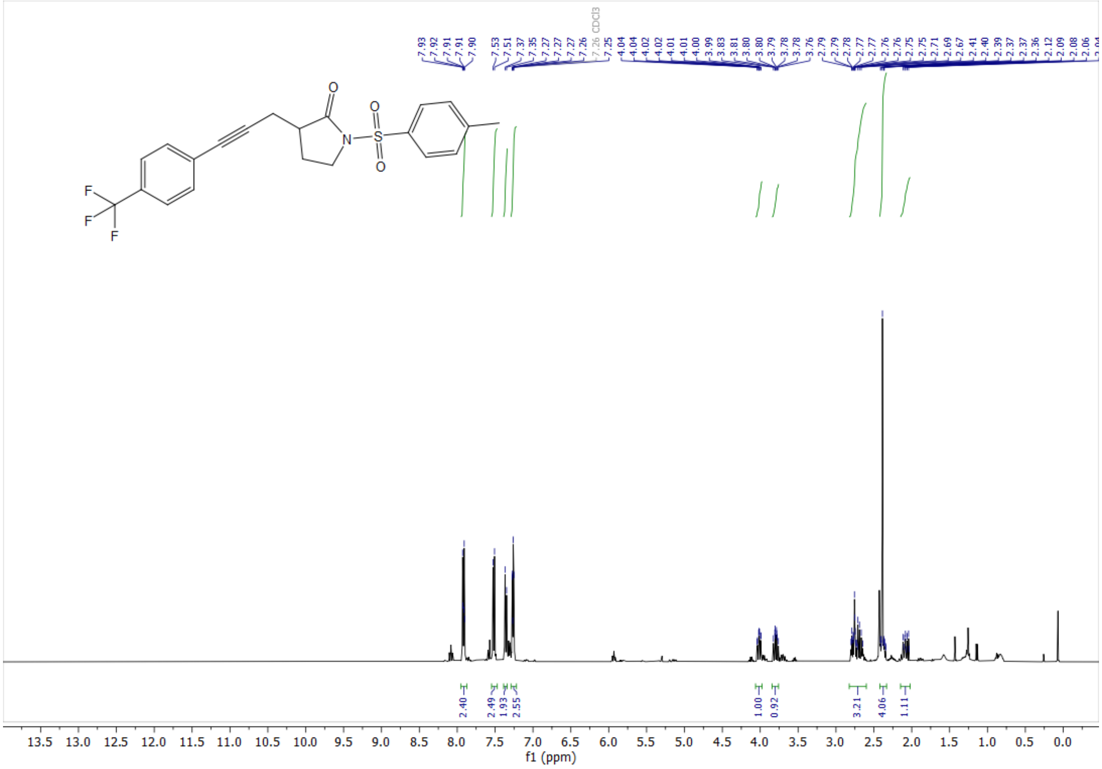

^{13}C {^{1}H} NMR Spectrum (101 MHz, $CDCl_3$) of **5x**

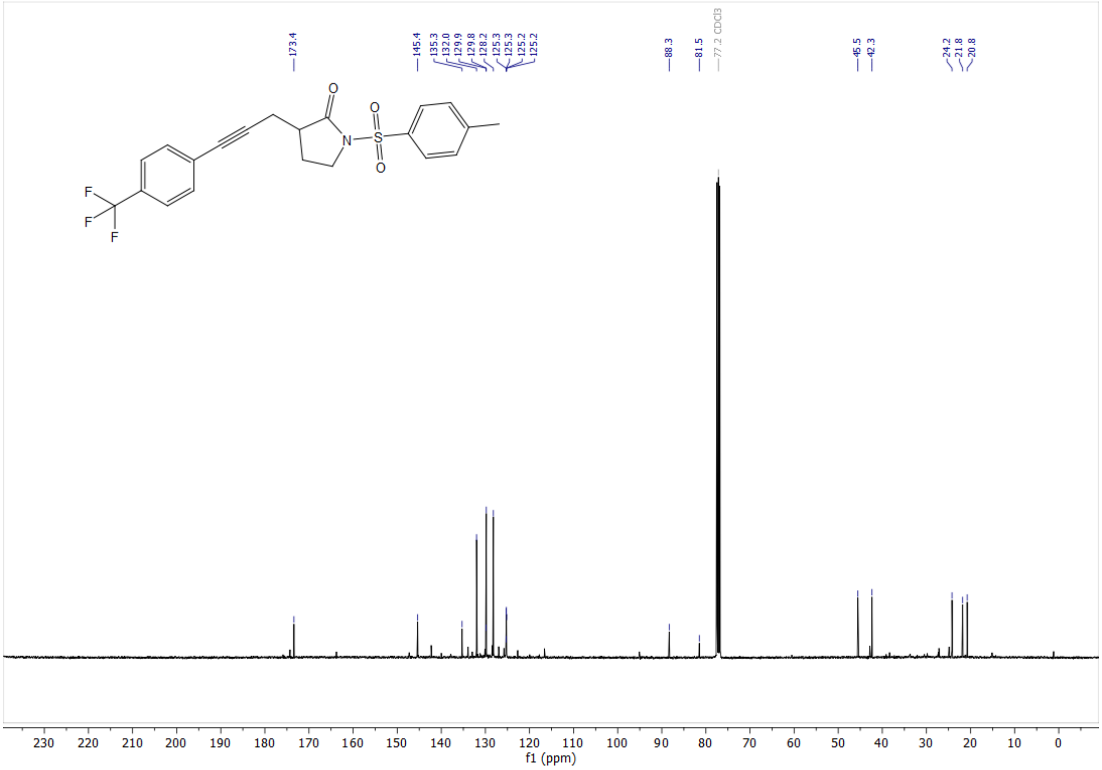

^{19}F {^{1}H} NMR Spectrum (376 MHz, $CDCl_3$) of **5x**

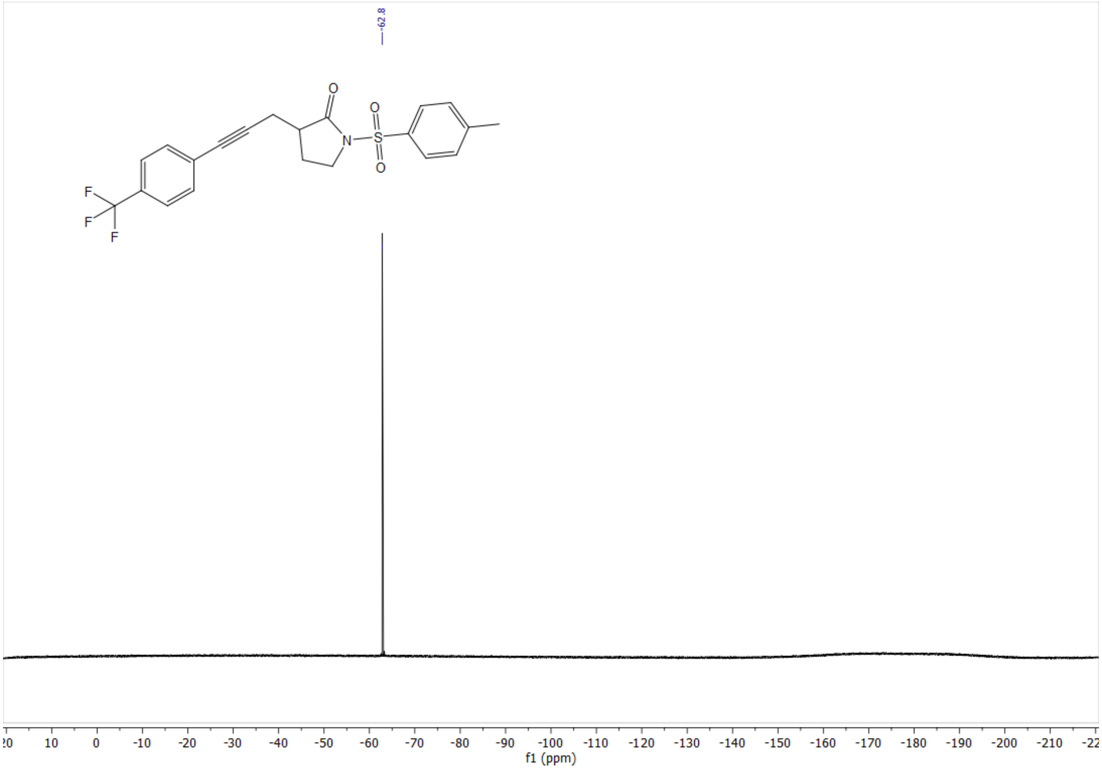

^{1}H NMR Spectrum (400 MHz, $CDCl_3$) of **5y**

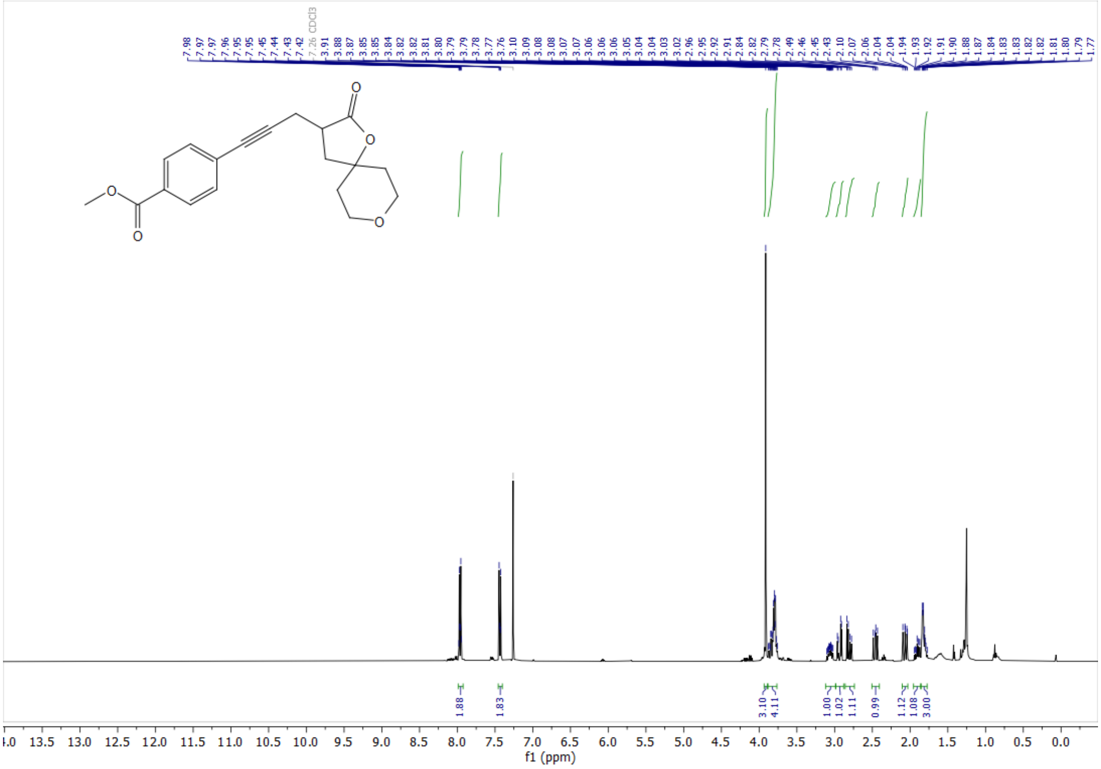

^{13}C {^{1}H} NMR Spectrum (101 MHz, $CDCl_3$) of **5y**

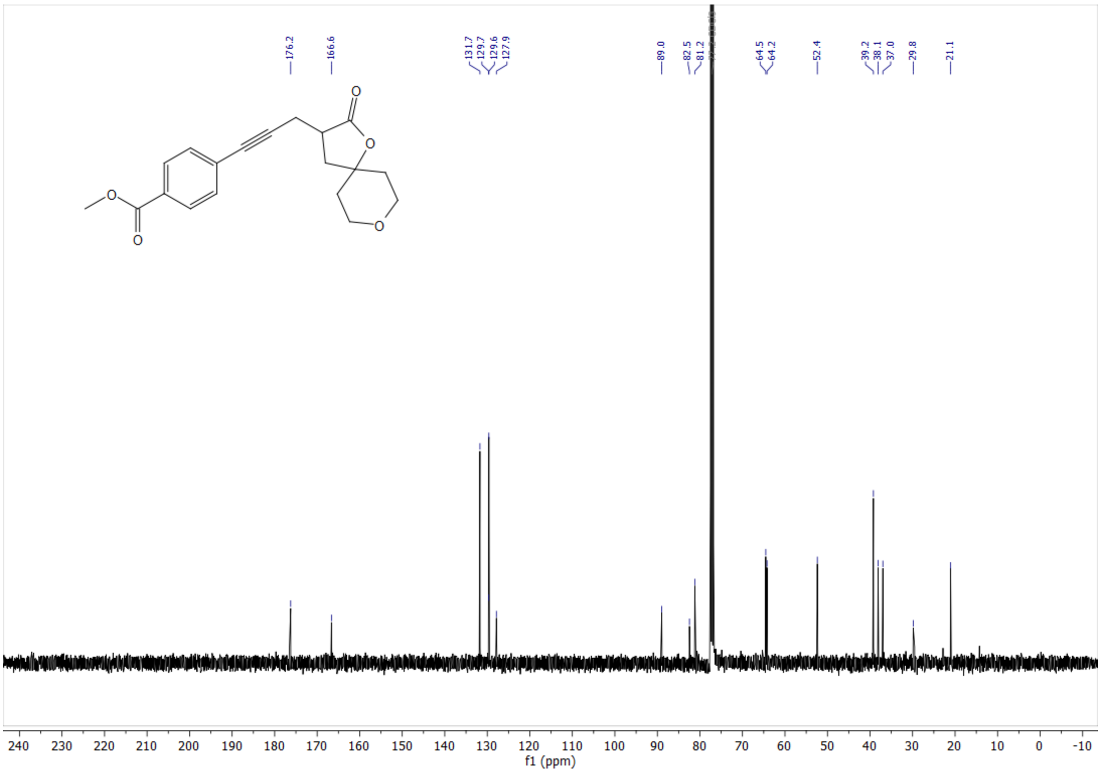

^{1}H NMR Spectrum (400 MHz, MeOD) of **5z**

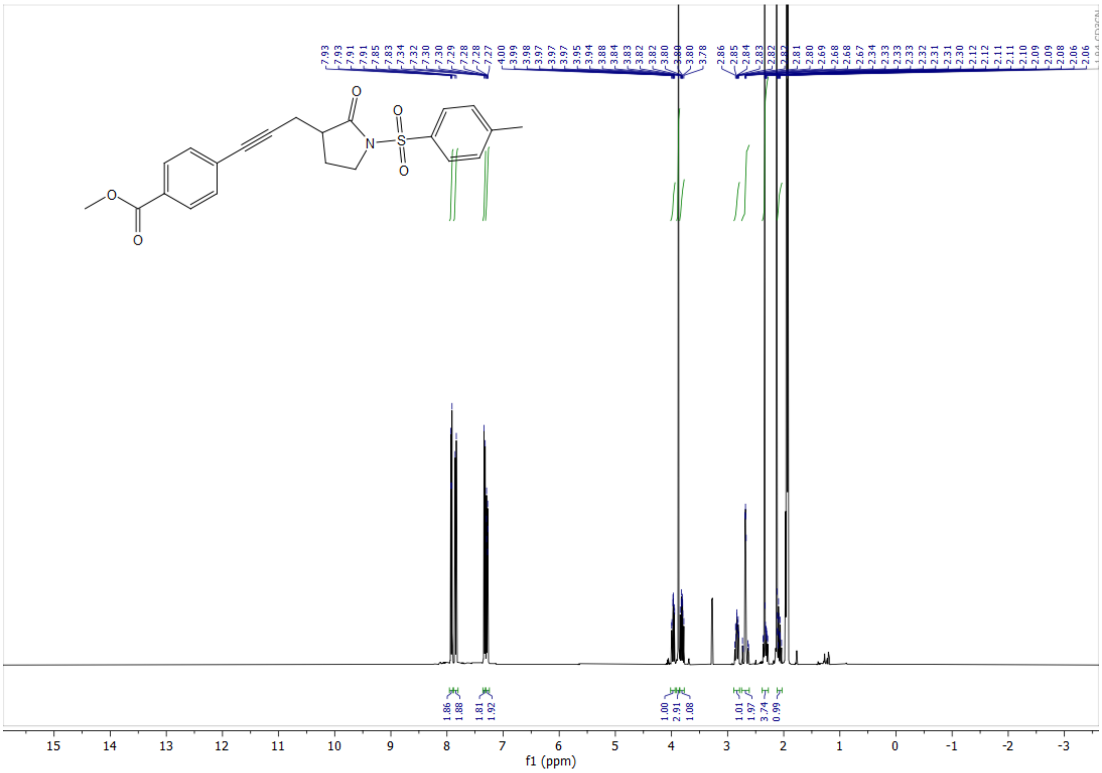

^{13}C {^{1}H} NMR Spectrum (101 MHz, MeOD) of **5z**

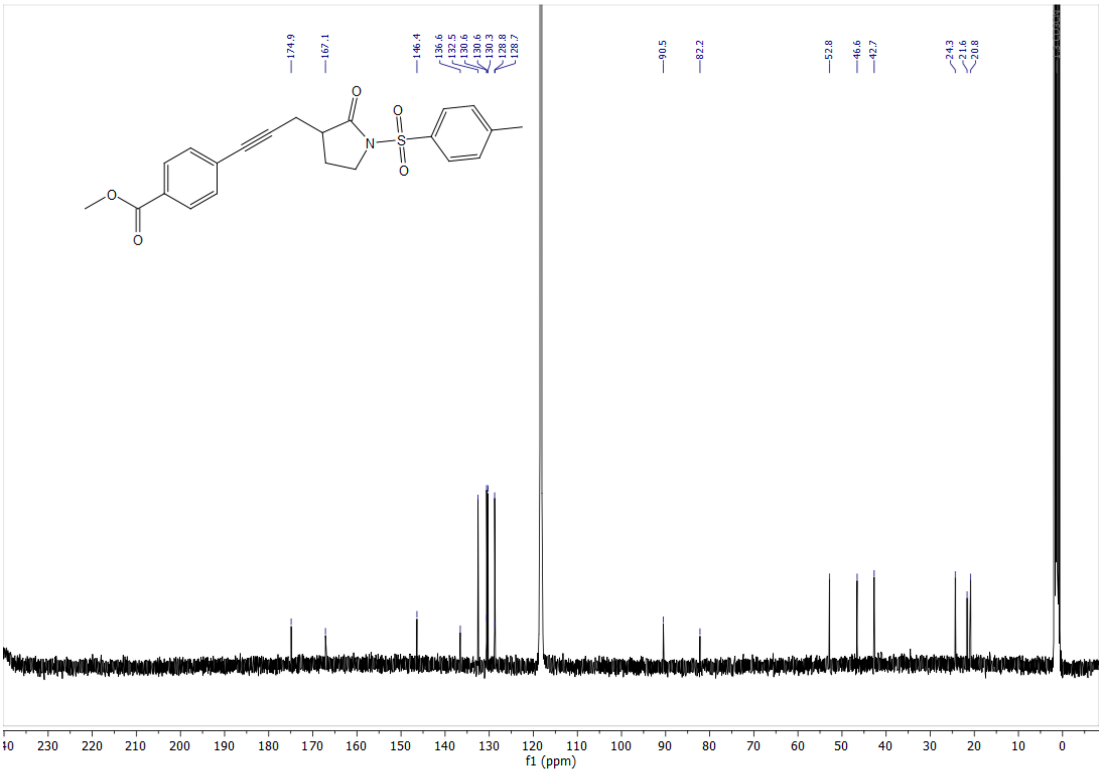

^{1}H NMR Spectrum (400 MHz, CD_3Cl) of **7**

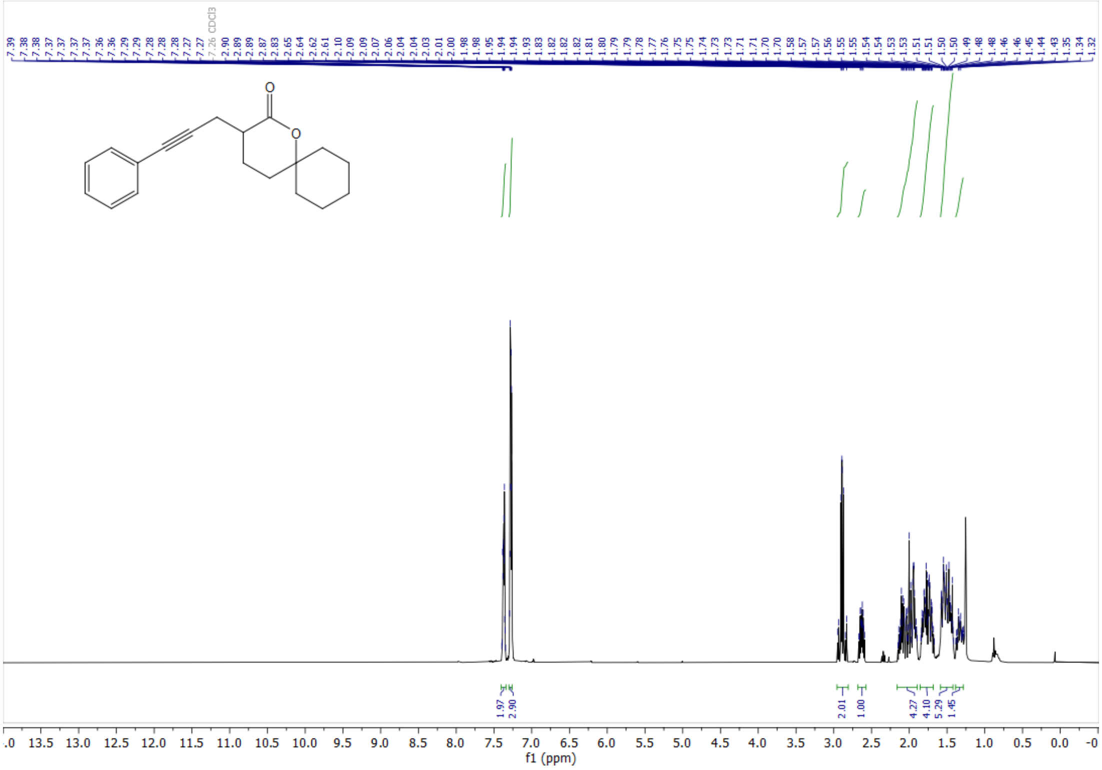

^{13}C {^{1}H} NMR Spectrum (101 MHz, CD_3Cl) of **7**

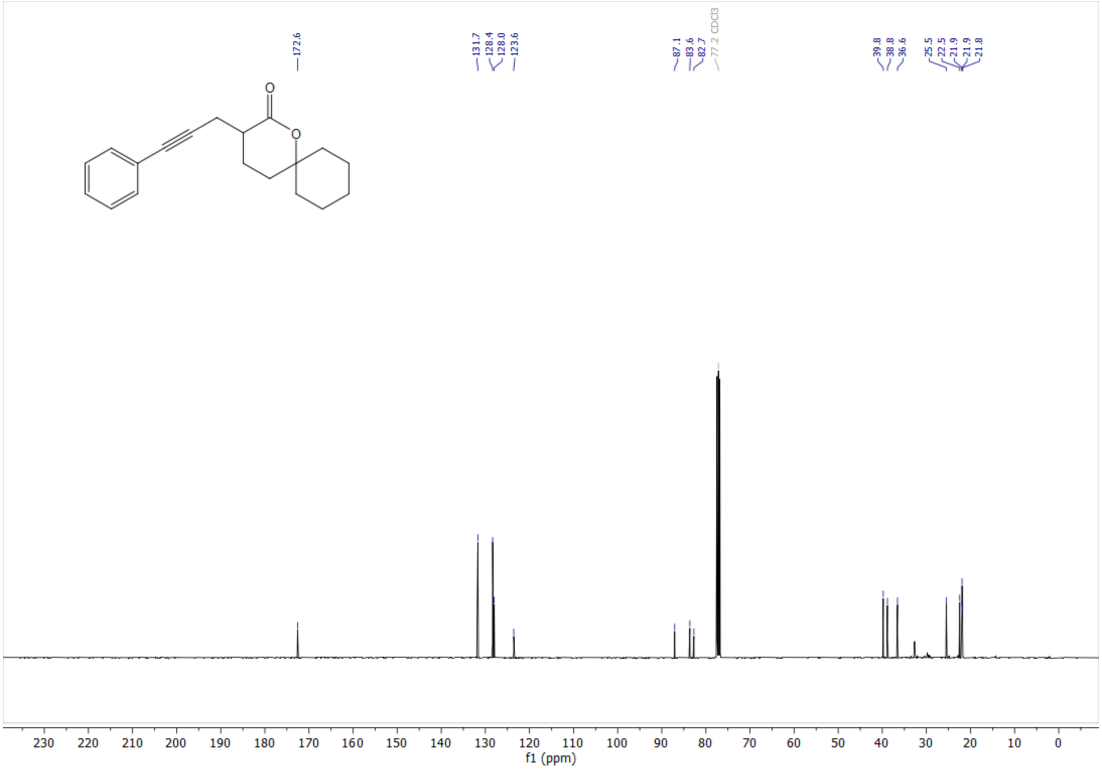

^{1}H NMR Spectrum (400 MHz, $CDCl_3$) of **9**

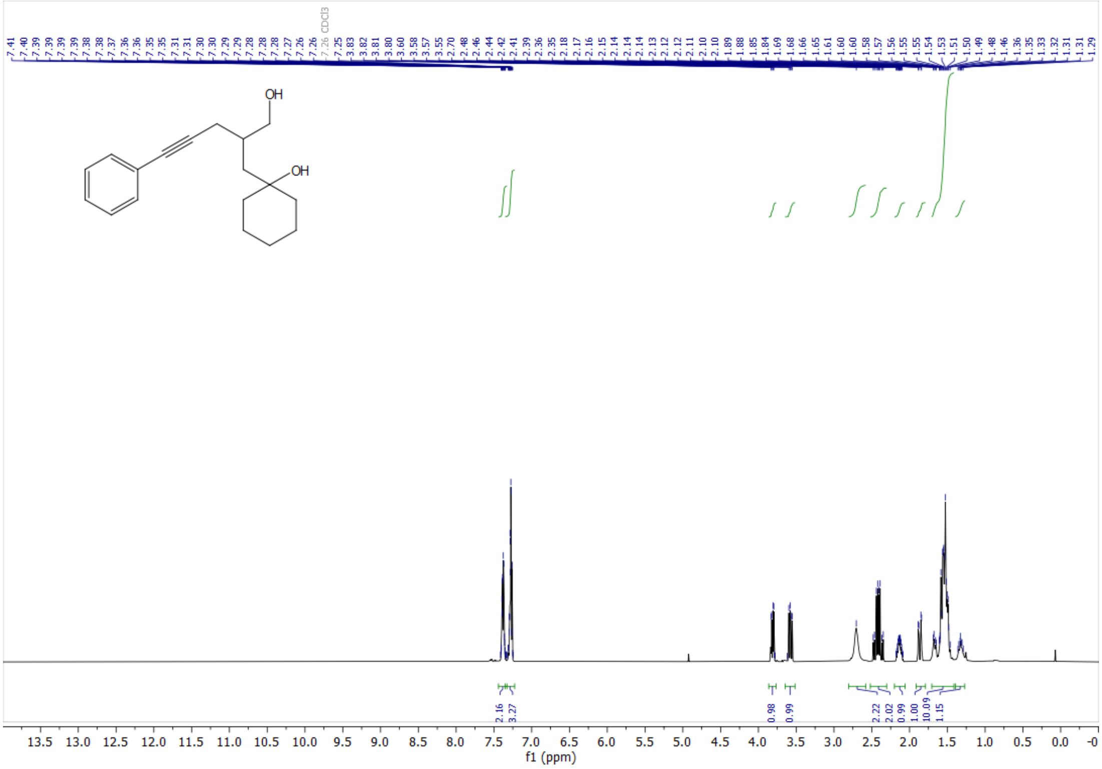

^{13}C {^{1}H} NMR Spectrum (400 MHz, $CDCl_3$) of **9**

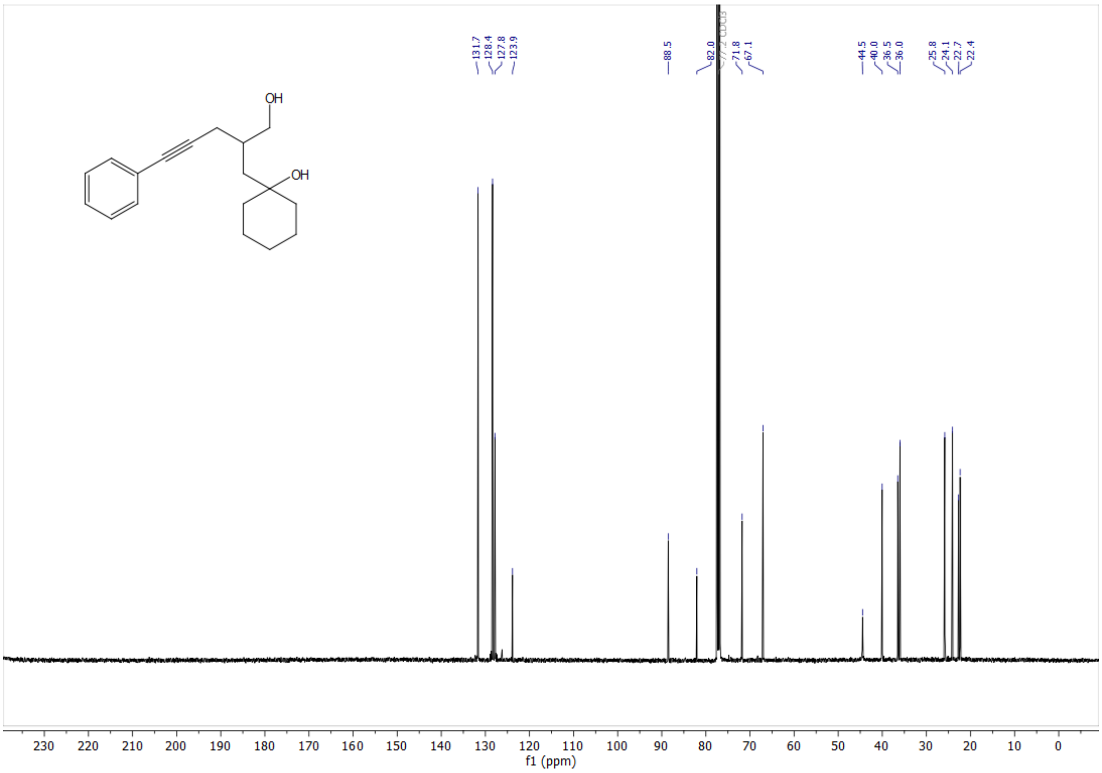

^{1}H NMR Spectrum (400 MHz, $CDCl_3$) of **10**

With * are shown the overreduction product.

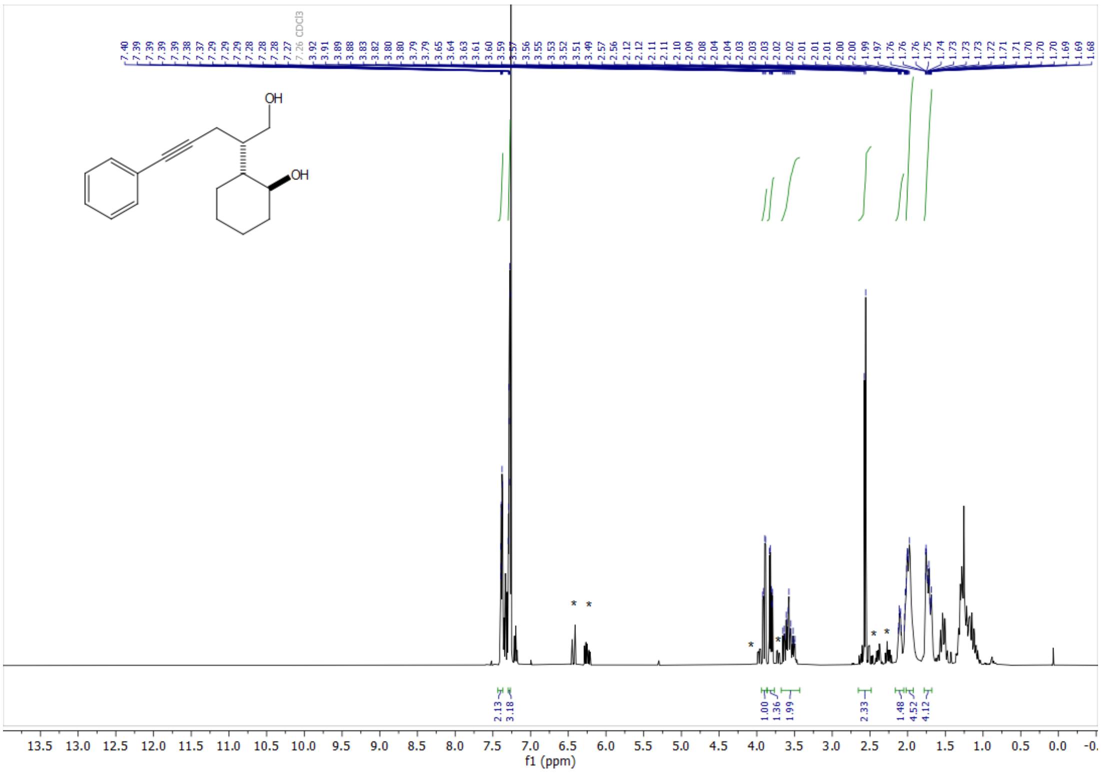

^{13}C {^{1}H} NMR Spectrum (101 MHz, $CDCl_3$) of **10**

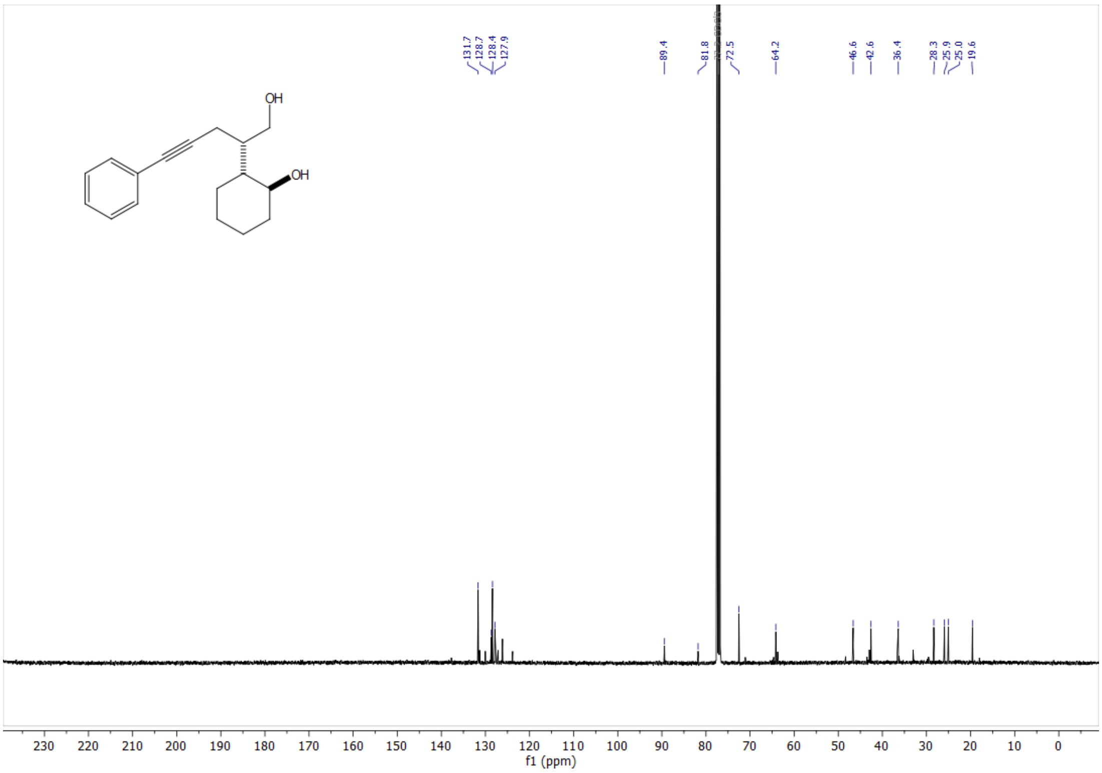

Supplement: Supplementary file 1 — ol4c01078_si_001.pdf [file ol4c01078_si_001.pdf]
